# Strong and fragile topological Dirac semimetals with higher-order Fermi arcs

**DOI:** 10.1038/s41467-020-14443-5

**Published:** 2020-01-31

**Authors:** Benjamin J. Wieder, Zhijun Wang, Jennifer Cano, Xi Dai, Leslie M. Schoop, Barry Bradlyn, B. Andrei Bernevig

**Affiliations:** 1 Department of Physics, Princeton University, Princeton, NJ 08544 USA; 2 Beijing National Laboratory for Condensed Matter Physics and Institute of Physics, Chinese Academy of Sciences, Beijing, 100190 China; 3 University of Chinese Academy of Sciences, 100049 Beijing, China; 4 Department of Physics and Astronomy, Stony Brook University, Stony Brook, NY 11974 USA; 5 Center for Computational Quantum Physics, The Flatiron Institute, New York, NY 10010 USA; 6 Physics Department, Hong Kong University of Science and Technology, Clear Water Bay, Hong Kong, Hong Kong; 7 Department of Chemistry, Princeton University, Princeton, NJ 08544 USA; 8 Department of Physics and Institute for Condensed Matter Theory, University of Illinois at Urbana-Champaign, Urbana, IL 61801-3080 USA; 9 Donostia International Physics Center, P. Manuel de Lardizabal 4, 20018 Donostia-San Sebastián, Spain; 10 Dahlem Center for Complex Quantum Systems and Fachbereich Physik, Freie Universität Berlin, Arnimallee 14, 14195 Berlin, Germany; 11 Max Planck Institute of Microstructure Physics, 06120 Halle, Germany

**Keywords:** Electronic properties and materials, Topological insulators

## Abstract

Dirac and Weyl semimetals both exhibit arc-like surface states. However, whereas the surface Fermi arcs in Weyl semimetals are topological consequences of the Weyl points themselves, the surface Fermi arcs in Dirac semimetals are not directly related to the bulk Dirac points, raising the question of whether there exists a topological bulk-boundary correspondence for Dirac semimetals. In this work, we discover that strong and fragile topological Dirac semimetals exhibit one-dimensional (1D) higher-order hinge Fermi arcs (HOFAs) as universal, direct consequences of their bulk 3D Dirac points. To predict HOFAs coexisting with topological surface states in solid-state Dirac semimetals, we introduce and layer a spinful model of an *s*–*d*-hybridized quadrupole insulator (QI). We develop a rigorous nested Jackiw–Rebbi formulation of QIs and HOFA states. Employing ab initio calculations, we demonstrate HOFAs in both the room- (*α*) and intermediate-temperature (*α″*) phases of Cd_3_As_2_, KMgBi, and rutile-structure ($\beta ^{\prime}$-) PtO_2_.

## Abstract (web summary)

The existence of a topological bulk-boundary correspondence for Dirac semimetals has remained an open question. Here, Wieder et al. predict one-dimensional hinge states originating from bulk three-dimensional Dirac points in solid-state Dirac semimetals, revealing condensed matter Dirac fermions to be higher-order topological.

## Introduction

Since the realization that the Fermi surface of graphene is characterized not only by its bulk 2D Dirac cones, but also by 1D arc-like states along zigzag edges^1^, there has been an ongoing effort to identify bulk-gapless systems with topological boundary modes. This effort has yielded a wide variety of 3D nodal semimetals with topological states on their 2D faces, including systems with bulk Weyl^2–4^ and unconventional^5–9^ fermions. Despite the presence of bulk gapless points in these semimetals, bands are still generically gapped in momentum space away from the nodal points, allowing for topological invariants to be defined along closed surfaces in the Brillouin zone (BZ)^2,10,11^. Nontrivial values of these invariants necessitate the presence of topological surface bands. Examples include the surface Fermi arcs in Weyl^2–4^ and unconventional chiral semimetals^6–9,12–15^, and topological boundary polarization modes, such as the solitons in the Su–Schrieffer–Heeger (SSH) and Rice–Mele chains^16,17^, the aforementioned Fermi arcs in graphene^1^, and the drumhead surface states in centrosymmetric nodal-line semimetals^11^. Researchers have also identified 3D Dirac semimetals with arc-like surface states that resemble the Fermi arcs of Weyl semimetals^10,18–20^. However, unlike the surface states of Weyl, nodal-line, and unconventional chiral semimetals, the surface Fermi arcs in Dirac semimetals can be disconnected and removed without breaking a symmetry or closing a gap^21^, and therefore are not topological consequences of the bulk Dirac points themselves. It has thus remained an open question as to whether 3D Dirac points can actually exhibit robust, nontrivial topology with spectroscopic consequences.

In this work, we exploit the theory of topological quantum chemistry (TQC)^22^ and recent advances in higher-order^23–32^ and fragile^33–37^ topology to discover a large family of 3D Dirac semimetals that exhibit intrinsic, polarization- (quadrupole-) nontrivial higher-order Fermi-arc (HOFA) states on their 1D hinges as direct, topological consequences of their bulk Dirac points, definitively diagnosing condensed matter Dirac fermions as higher-order topological. The HOFA states introduced in this work therefore represent a robust manifestation of a topological bulk-hinge correspondence in experimentally established 3D solid-state semimetals, and may be observable through experimental probes such as scanning tunneling microscopy (STM) and nonlocal quantum oscillation measurements. We support our findings with extensive analytic, tight-binding, and first-principles calculations.

## Results

### Boundary modes in topological tuning cycles

**Fig. 1:**
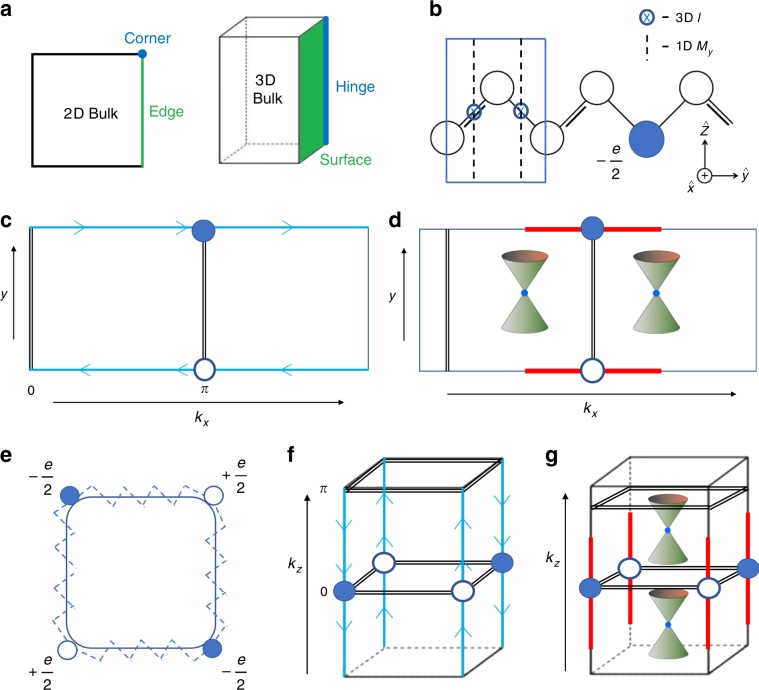
Tuning cycles of 1D and 2D insulators with 0D boundary states. **a** Terminology for the bulk and boundary of 2D and 3D systems. **b** A *y*-directed 1D SSH chain with quantized polarization, enforced by either 3D inversion ${\mathcal{I}}$ or 1D mirror symmetry along the chain (e.g., the operation *M*_*y*_, which takes *y* → −*y*)^16,17^. **c** In a 2D crystal with ${\mathcal{I}}$ symmetry, *k*_*x*_ can be treated as a parameter that periodically tunes between a *y*-directed SSH chain (double black lines in **c** and **d**) with zero polarization (*k*_*x*_ = 0) and another with *e*∕2 polarization (*k*_*x*_ = *π*), yielding a Chern insulator^17,40^ with chiral edge modes (blue lines). **d** In a 2D crystal with *M*_*y*_ symmetry instead of ${\mathcal{I}}$, the Hamiltonian at each value of *k*_*x*_ is equivalent to that of a *y*-directed SSH chain with a quantized polarization of 0 or *e*∕2; because the polarization cannot change continuously, a periodic tuning cycle indexed by *k*_*x*_ between SSH polarizations 0 and *e*∕2 must pass through a pair of gapless points. This yields a 2D band-inverted semimetal with topological polarization modes (red lines) analogous to those in zigzag-terminated graphene^1,44^. **e** A ${{\mathbb{Z}}}_{2}$ quantized quadrupole insulator (QI)^23^ invariant under wallpaper group *p*4*m*. **f** A *C*_4*z*_-broken, mirror-preserving pumping cycle of a QI (double black lines in **f** and **g**) is equivalent to a 3D 2nd-order Chern insulator^24–27^ with chiral hinge modes (blue lines), whereas **g** a *p*4*m*-preserving cycle is equivalent to a 3D Dirac semimetal with higher-order Fermi arcs (HOFAs) on its 1D hinges (red lines).

To provide context for the analysis performed in this work, we first review the crucial distinctions between topological polarization boundary modes and the surface states of topological insulators (TIs). Whereas in topological (crystalline) insulators^38,39^ the Bloch wavefunctions do not admit a description in terms of symmetric, exponentially localized Wannier functions^22,40,41^, insulating phases with only quantized electric polarization conversely do admit a Wannier description; the quantized polarization leads to a nontrivial Berry phase indicating the positions of the electronic Wannier centers relative to the ionic positions^22,42^. In these insulators, such as the SSH chain^16,17^, the Berry phase is quantized by the presence of a crystal symmetry, typically mirror reflection *M* or spatial inversion ${\mathcal{I}}$ (Fig. 1b). Correspondingly, the boundary between insulators with differing polarizations forms a domain wall that binds a topological soliton of fractional charge^16,17^, though the energy of this mode may float away from zero if particle-hole symmetry is broken. Nevertheless, as observed in polyacetylene^16,43^, zigzag-terminated graphene^44^, and nodal-line semimetals^11,45,46^, topological polarization boundary modes can still frequently lie near the Fermi energy in real materials.

By reinterpreting one of the momenta as an external parameter, a subset of topological semimetals and (crystalline) insulators, can be reexpressed as the adiabatic, cyclic tuning of an insulator with quantized electric polarization in one fewer dimension^40^. For example, the *y*-directed (hybrid) Wannier centers of a Chern insulator exhibit spectral flow as a function of the momentum *k*_*x*_, which can be indicated by the product of parity eigenvalues if $$ {\mathcal{I}} $$ symmetry is present^40,47^. Reinterpreting *k*_*x*_ as an external tuning parameter, we can recast the Chern number, *C*, as a nontrivial tuning cycle (Thouless pump) of a 1D SSH chain; as *k*_*x*_ is tuned from 0 to 2*π*, charge *eC* is pumped across the unit cell of the crystal. If the cycle is $$ {\mathcal{I}} $$-symmetric, then *C* mod 2 can be detected by (twice) the change in quantized polarization between effective *y*-directed SSH chains at *k*_*x*_ = 0 and^17,47^ *k*_*x*_ = *π*. We show this schematically in Fig. 1(c). In a crystalline semimetal, the presence of additional symmetries in the tuning cycle can force the gap to close at certain values of *k*_*x*_. For instance, adding (spinless) time-reversal ($$ {\mathcal{T}} $$) symmetry to the $$ {\mathcal{I}} $$-symmetric Thouless pump obstructs the presence of a nonzero Chern number; in order for the polarization to change by *e*∕2 from *k*_*x*_ = 0 to *k*_*x*_ = *π*, there must be a gapless point^11^ at some $$ {k}_{x}^{* }\in (0,\pi ) $$, with a time-reversed partner at $$ -{k}_{x}^{* }$$. A similar gapless point occurs when the polarization of an SSH chain in line group *pm* is periodically tuned (Fig. 1d). There, taking the mirror to be *M*_*y*_, each value in parameter space indexed by the periodic tuning parameter *k*_*x*_ corresponds to a *y*-directed SSH chain with a quantized polarization indicated by the mirror eigenvalues of the occupied bands^34^; each time the polarization jumps between 0 and *π*, a robust gapless point forms because the crossing bands carry different mirror eigenvalues.

Recently these arguments were generalized to higher electric multipole moments. In ref. ^23^, the authors demonstrated the theoretical existence of spinless insulators with threaded flux that exhibit trivial dipole moments, but topologically quantized electric quadrupole and octupole moments, and which host boundary (corner) modes in two and three dimensions fewer than the bulk, respectively (Fig. 1a, e). Many of these corner-mode phases^23,24,26^ can be identified by their bulk symmetry eigenvalues, exploiting the theory of band representations^22,35,36,48–50^. As shown in recent independent proposals, imposing combinations of rotational, rotoinversion, and ${\mathcal{T}}$ symmetries allows for 3D topological insulating crystals that are equivalent to nontrivial pumping cycles of quantized quadrupole insulators (QIs)^24–27,51^, or other 2D phases with corner modes^29,30,32,37,49^. These 3D higher-order TIs^24–32^ host chiral or helical modes not on their 2D faces, but instead on their 1D hinges (Fig. 1a, f).

### Summary of results

In this work, we present the discovery of higher-order (polarization) topology and HOFA states in a large family of previously identified Dirac semimetals, completing the set of interrelated (higher-order) TIs and semimetals shown in Fig. 1. We demonstrate the intrinsic, topological nature of the HOFA states by performing several extensive calculations that bridge the significant gap between previously established theoretical concepts and the candidate real-material HOFA Dirac semimetals identified in this work. First, we use TQC^22^ to formulate a new, spinful model of a QI derived from *s*–*d*-orbital hybridization in a magnetic layer group, and show that it is topologically equivalent to the spinless model with staggered magnetic flux proposed in ref. ^23^ (see Supplementary Note  2). This puts the QI (*s*–*d* hybridization) on the same physical foundation as previous dipole insulators, such as the SSH chain^16,17^ (*s*–*p* hybridization). We then prove using band representations that the QI is an obstructed atomic limit with localizable Wannier functions^22^ (Supplementary Note 3). Next, we use crystal symmetry to develop an extensive, angular-momentum-based, nested Jackiw–Rebbi^52^ formulation of intrinsic corner modes in order to analytically obtain the bound states of the *s*–*d*-hybridized QI (Supplementary Note 6) and to relate them to SSH (anti)solitons (Fig. 1(e) and Supplementary Note 6). Because our construction employs an isotropic (i.e., cylindrical) boundary, it uniquely represents an analytic formulation of the QI in which the presence of intrinsic 0D boundary modes can be separated from the extrinsic effects of the singular curvature of sharp corners. Furthermore, because our construction is explicit, general, and rigorous, it can also be employed to predict and analyze other corner-mode phases^32,37^. Through our TQC-based model of a QI and our Jackiw–Rebbi analysis, we discover a fragile topological phase^33–37^ that exhibits the same corner charges as a QI modulo *e*; because these charges are a property of the fragile bands closest to the Fermi energy, they persist even when the valence manifold of the fragile phase is trivialized by additional (trivial) bands (Supplementary Notes 4 and 9), as is expected to occur in real materials. Stacking our spinful, TQC-based model of a QI in 3D, we construct both ${\mathcal{T}}$-symmetric and ${\mathcal{T}}$-breaking realizations of Dirac semimetals with higher-order Fermi arcs (HOFAs) on their 1D hinges (Fig. 1a, g), i.e., in two fewer dimensions than their bulk. Furthermore, unlike the surface Fermi arcs in Dirac semimetals, which can be removed by symmetry- and bulk-band-order-preserving potentials^21^, HOFA states represent a direct, topological boundary consequence of the bulk Dirac points.

Crucially, because our analysis is derived from TQC, atomic orbitals, and symmetry-based (nested) Jackiw–Rebbi domain walls, it allows the immediate connection to real materials, unlike recent toy models with HOFA states (i.e., the flux-lattice and particle-hole symmetric semimetallic models in refs. ^53,54^, respectively) that appeared while we were expanding our material search to fragile and experimentally favorable structural phases of established topological Dirac semimetals. Specifically, while one can naively stack the spinless QI and obtain a toy-model HOFA state, without the careful symmetry- and orbital-based analysis developed in this work, the resulting HOFA states bear no clear connection to 2D TIs (Supplementary Notes 7 and 8), topological crystalline insulator (TCIs) (Supplementary Note 9), fragile phases (Supplementary Notes 4 and 9), or to 3D Dirac points in real materials (Supplementary Notes 12 and 13). Furthermore, in this work, we explicitly relax particle-hole symmetry, which numerous other works, such as ref. ^54^, centrally exploit. Because particle-hole is not generically a symmetry of real materials, it can protect corner (and thus HOFA) states that appear in toy models, but which are not observable in real materials. This can be understood by making an analogy to the SSH model of polyacetylene^16,17^. Specifically, while real polyacetylene exhibits only ${{\mathbb{Z}}}_{2}$ polarization topology^16,17^, the particle-hole symmetric toy-model SSH chain exhibits strong, ${\mathbb{Z}}$-valued topology (Class AIII in the nomenclature of ref. ^55^).

We predict previously unidentified HOFAs and related fragile-phase corner charges (Supplementary Note 9) in established candidate Dirac semimetals. We present ab initio and tight-binding calculations demonstrating the presence of HOFAs in the intermediate-temperature (*α″*) phase of the well-studied Dirac semimetal Cd_3_As_2_ in space group (SG) 137 ($P{4}_{2}/nmc1^{\prime}$)^10,18,56,57^ and in the candidate Dirac semimetals KMgBi in SG 129 ($P4/nmm1^{\prime}$)^58,59^ and rutile-structure ($\beta ^{\prime}$-) PtO_2_ in SG 136 ($P{4}_{2}/mnm1^{\prime}$)^60,61^ (here and throughout this work, we follow ref. ^62^ in using primes to denote antiunitary group elements). We also use symmetry arguments to predict that the archetypal room-temperature (*α*) phase of Cd_3_As_2_ in SG 142 ($I{4}_{1}/acd1^{\prime}$) exhibits a related variant of HOFA states that derive from relaxing the reflection symmetries of the QI phase (Supplementary Note 12). Finally, we also demonstrate that, in the presence of an external electric field, the topological Dirac semimetal phase of $\beta ^{\prime}$-PtO_2_ can be converted into a previously uncharacterized variant of fragile topological Dirac semimetal that displays HOFA states coexisting with fractionally charged corner (hinge) state.

### 2D TCIs, fragile TIs, and QIs in *p*4*m*

**Fig. 2:**
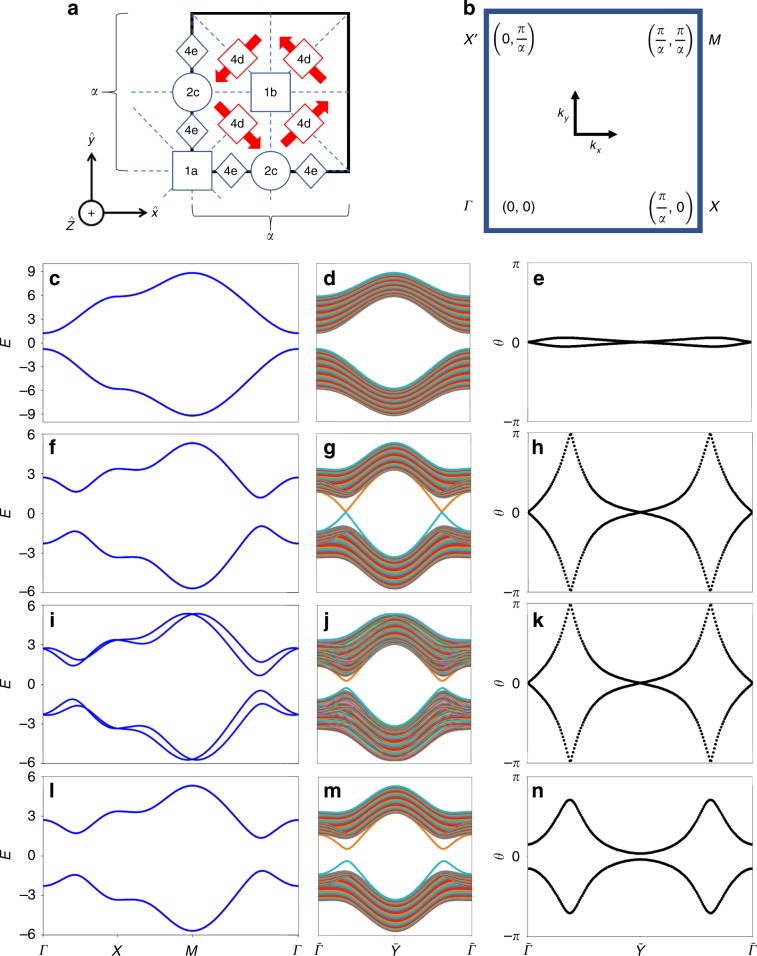
TCI, fragile, and QI phases in 2D insulators with *p*4*m* symmetry. **a** ${\mathcal{T}}$-symmetric layer group $p4/mmm1^{\prime}$ reduces to type-III magnetic layer group^62^ $p4/m^{\prime} mm$ under the application of a magnetic potential with no net magnetic moment in each unit cell (Supplementary Note 3); this can be achieved by placing spin-1∕2 magnetic moments (red arrows in **a**) at the 4*d* Wyckoff position with orientations related by *C*_4*z*_ and *M*_*x*,*y*_. **b** BZ and **c** bulk bands for a model (Eq. (1)) that respects $p4/mmm1^{\prime}$ (Table 1), which has *M*_*z*_ and ${\mathcal{T}}$ symmetries, as well as the symmetries of $p4/m^{\prime} mm$ in **a**; this model is constructed from *s* and ${d}_{{x}^{2}-{y}^{2}}$ orbitals at the 1*a* Wyckoff position. Eq. (1) can be tuned between a trivial and a mirror TCI phase (**f**), distinguished by their (**d**, **g**) ribbon edge spectra and (**e**, **h**) *x*-directed Wilson loops plotted as functions of *k*_*y*_ (Eq. (2)). **i** Relaxing *M*_*z*_ while preserving *C*_2*z*_ and ${\mathcal{T}}$ by introducing Eq. (3) to Eq. (1), (**k**) we realize a four-band model with the same Wilson loop winding as the 2D TCI phase in **f**–**h**, but (**j**) without topological edge states. The Wilson loop in **k** can either be trivialized by the addition of more orbitals to the model (Supplementary Note 4), or gapped by the magnetic potential depicted in **a**. **l** Upon gapping (**m**) the surface and (**n**) Wilson bands with magnetism that breaks *M*_*z*_ while preserving magnetic wallpaper group^28,62^ *p*4*m*, which we accomplish by adding the potential in Eq. (4), the Wannier centers of the topological phases in **f**–**k** localize at the 1*b* position (Supplementary Note 3), realizing an insulator topologically equivalent to the QI introduced in ref. ^23^ (Supplementary Note 2).

We begin by providing a more physical formulation of the 2D QI introduced in ref. ^23^ using atomic orbitals, which clarifies the connection with the SSH chain. We place spin-1 ∕ 2*s* and ${d}_{{x}^{2}-{y}^{2}}$ orbitals at the center (1*a* Wyckoff position) of a square unit cell in 2D (Fig. 2(a)) and then, following ref. ^23^, impose the symmetries of wallpaper group *p*4*m*, which is generated by *M*_*x*_ and *C*_4*z*_ about the 1*a* position in Fig. 2, as well as 2D square lattice translations (for the distinctions between wallpaper and layer groups and their relationship to topological semimetals and insulators, see ref. ^28^). In addition to the symmetries of *p*4*m*, we will first additionally impose *M*_*z*_ and ${\mathcal{T}}$ symmetries to explore 2D phases with spin-orbit coupling (SOC), and then subsequently relax *M*_*z*_ and ${\mathcal{T}}$ with magnetism to induce the QI. Eliminating all nonessential symmetries and degeneracies, we form the Hamiltonian:

1 $$ {\mathcal{H}}({\bf{k}})=	\, {t}_{1}{\tau }^{z}[\cos ({k}_{x})+\cos ({k}_{y})]+{t}_{2}{\tau }^{x}[\cos ({k}_{x})-\cos ({k}_{y})]\\ 	 \!+{v}_{m}{\tau }^{z}+{t}_{PH}{\mathbb{1}}_{\tau \sigma }[\cos ({k}_{x})+\cos ({k}_{y})]\\ 	\! + {v}_{s}{\tau }^{y}{\sigma }^{z}\sin ({k}_{x})\sin ({k}_{y}), $$

where *τ* (*σ*) indexes the *s*, *d*-orbital (spin) degree of freedom and ${\mathbb{1}}$_*τσ*_ is the 4 × 4 identity. Here, *v*_*m*_ produces on-site orbital splitting, *t*_1_ (*t*_2_) is first-neighbor hopping between the same (opposite) orbital, *t*_*PH*_ is spin- and orbital-independent first-neighbor hopping that explicitly breaks particle-hole symmetry, and *v*_*s*_ represents second-neighbor SOC (Supplementary Note 1). Equation (1) is invariant under the symmetries of layer group $p4/mmm1^{\prime}$ (Table 1). Since ${\mathcal{I}}={M}_{x}{M}_{y}{M}_{z}$ is given by the identity matrix and {*M*_*x*_, *M*_*y*_} = 0 in the representation in Table 1, our model with four spinful orbitals (Eq. (1)) exhibits the same bulk symmetry eigenvalues and symmetry algebra as the original, spinless QI model in ref. ^23^. The bulk bands of Eq. (1), due to the presence of ${\mathcal{I}}\times {\mathcal{T}}$ symmetry, are twofold degenerate (Fig. 2b, c). In Supplementary Note 5 and 6, we additionally introduce and analyze models of QIs and HOFA semimetals with *p*–*d* hybridization.

**Table 1:** The symmetry representation of the 2D and 3D *s*–*d*-hybridized models in the main text (Eqs. (1), (3), (4), (5), and (6)).

Symmetries of 2D Hamiltonians ${\mathcal{H}}({k}_{x},{k}_{y})$	
*g*	$g{\mathcal{H}}(g{k}_{x},g{k}_{y}){g}^{-1}$
*M*_*x*_	${\sigma }^{x}{\mathcal{H}}(-{k}_{x},{k}_{y}){\sigma }^{x}$
*M*_*y*_	${\sigma }^{y}{\mathcal{H}}({k}_{x},-{k}_{y}){\sigma }^{y}$
*C*_4*z*_	${\tau }^{z}\left(\frac{{{\mathbb{1}}}_{\sigma }-i{\sigma }^{z}}{\sqrt{2}}\right){\mathcal{H}}({k}_{y},-{k}_{x}){\tau }^{z}\left(\frac{{{\mathbb{1}}}_{\sigma }+i{\sigma }^{z}}{\sqrt{2}}\right)$
*M*_*z*_	${\sigma }^{z}{\mathcal{H}}({k}_{x},{k}_{y}){\sigma }^{z}$
${\mathcal{I}}$	${\mathcal{H}}(-{k}_{x},-{k}_{y})$
${\mathcal{T}}$	${\sigma }^{y}{{\mathcal{H}}}^{* }(-{k}_{x},-{k}_{y}){\sigma }^{y}$

Symmetries of 3D Hamiltonians ${\mathcal{H}}({k}_{x},{k}_{y},{k}_{z})$	
*g*	$g{\mathcal{H}}(g{k}_{x},g{k}_{y},g{k}_{z}){g}^{-1}$
*M*_*x*_	${\sigma }^{x}{\mathcal{H}}(-{k}_{x},{k}_{y},{k}_{z}){\sigma }^{x}$
*M*_*y*_	${\sigma }^{y}{\mathcal{H}}({k}_{x},-{k}_{y},{k}_{z}){\sigma }^{y}$
*C*_4*z*_	${\tau }^{z}\left(\frac{{{\mathbb{1}}}_{\sigma }-i{\sigma }^{z}}{\sqrt{2}}\right){\mathcal{H}}({k}_{y},-{k}_{x},{k}_{z}){\tau }^{z}\left(\frac{{\mathbb{1}}_{\sigma }+i{\sigma }^{z}}{\sqrt{2}}\right)$
*M*_*z*_	${\sigma }^{z}{\mathcal{H}}({k}_{x},{k}_{y},-{k}_{z}){\sigma }^{z}$
${\mathcal{I}}$	${\mathcal{H}}(-{k}_{x},-{k}_{y},-{k}_{z})$
${\mathcal{T}}$	${\sigma }^{y}{{\mathcal{H}}}^{* }(-{k}_{x},-{k}_{y},-{k}_{z}){\sigma }^{y}$

The models in Eqs. (3–6) derive from Eq. (1), which contains the symmetries (wallpaper group^28^ *p*4*m*) of a QI^23,24^ (Supplementary Note 3), as well as *M*_*z*_, ${\mathcal{I}}={M}_{x}{M}_{y}{M}_{z}$, and ${\mathcal{T}}$.

To diagnose the topology of Eq. (1), we examine the *x*-directed Wilson loop (holonomy) matrix^34,36,63^, a bulk quantity defined by:

2 $$ {{\mathcal{W}}}_{({k}_{x0},{k}_{y})}\equiv P{e}^{i\int_{{k}_{x0}}^{{k}_{x0}+2\pi }d{k}_{x}{A}_{x}({k}_{x},{k}_{y})}, $$

where *P* indicates that the integral is path-ordered and ${A}_{x}{(k)}_{ij}\equiv i\langle {u}^{i}(k)| {\partial }_{{k}_{x}}{u}^{j}(k)\rangle$ is the matrix-valued Berry connection. The eigenvalues *θ*(*k*_*y*_) of ${\mathcal{W}}$ are gauge invariant and form bands in one fewer dimension than that of the bulk, with connectivity and degeneracy constrained by the symmetries of the *x*-projected edge symmetry group^28^, as well as by the representations of bulk symmetries^34^. At half filling, Eq. (1) exhibits two topologically distinct insulating phases (Fig. 2c, f), indicated by the relative ordering of the Kramers pairs of *C*_4*z*_ eigenvalues of the occupied bands at Γ and *M* (Fig. 2b)^23^. In Fig. 2, we show the Wilson spectra computed from the lower two bands of Eq. (1) in the uninverted (trivial) phase (e) and in the inverted (nontrivial) phase (h). As we will detail below, we then also calculate the Wilson spectrum of Eq. (1) in the presence of potentials that break *M*_*z*_ symmetry while either preserving (k) or breaking (n) ${\mathcal{T}}$ symmetry; we also compare the Wilson loop spectra to the surface states of tight-binding Hamiltonians calculated in a ribbon geometry (d,g,j,m).

Using Eq. (2), we identify the nontrivial phase of Eq. (1) as a TCI^28,64,65^ with mirror Chern number ${C}_{{M}_{z}}=2$ (Fig. 2g, h). By introducing a term that breaks *M*_*z*_ and ${\mathcal{I}}$ while preserving the symmetries of wallpaper group $p4m1^{\prime}$ (generated by *M*_*x*,*y*_, *C*_4*z*_,  and ${\mathcal{T}}$)^28,62^:

3 $$ {V}_{{M}_{z}}({\bf{k}})={v}_{{M}_{z}}\left[{\tau }^{z}{\sigma }^{y}\sin ({k}_{x})-{\tau }^{z}{\sigma }^{x}\sin ({k}_{y})\right], $$

we can gap the edge states of this TCI (Fig. 2j). However, its two-band *x*-directed Wilson loop still winds (Fig. 2k). This phenomenon is related to recently identified fragile topological phases^33–37^, whose Wilson loops can be rendered topologically trivial by the introduction of trivial bands. In Supplementary Note 4, we show how the topological Wilson connectivity of this four-band model is unstable to the addition of spinful *s* orbitals at the 2*c* position of $p4m1^{\prime}$. In both the TCI (Eq. (1)) and fragile (Eqs. (1) and (3)) phases, ${\mathcal{T}}$ symmetry obstructs the presence of singly degenerate corner modes; however, we found that the *M*_*z*_-broken fragile phase, when the overall system is kept at a constant half filling, still exhibits three-quarters-filled Kramers pairs of corner modes that can float into the bulk gap (Supplementary Notes 4 and 9). We specifically show in Supplementary Note 9 that, as this fragile phase can be connected to a QI by restoring ${\mathcal{T}}$ symmetry without closing a bulk or edge gap, its corner modes still exhibit the same charges as the QI modulo *e*. ${C}_{{M}_{z}}=2$ TCI phases in layer group $p4/mmm1^{\prime}$ have been proposed in XY (X=Sn, Te; Y=S, Se, Te) monolayers^66^. However because band inversion in these XY monolayers occurs at the *X* and $X^{\prime}$ points (Fig. 2b) between bands with different ${\mathcal{I}}$ eigenvalues, rather than at the Γ or *M* points between bands with different pairs of *C*_4*z*_ eigenvalues, XY monolayers will realize a different insulating phase than the fragile phase of Eqs. (1) and (3) when *M*_*z*_ is broken with a substrate or an external field (Supplementary Note 9).

To induce the QI phase (Fig. 2l), we first set ${v}_{{M}_{z}}=0$ in Eq. (3); this restores *M*_*z*_ and ${\mathcal{I}}$ symmetries. We then instead add to Eq. (1) a term that anticommutes with ${\mathcal{H}}({\bf{k}})$ in its particle-hole symmetric limit (*t*_*PH*_ → 0):

4 $$ U({\bf{k}})=u[{\tau }^{y}{\sigma }^{y}\sin ({k}_{x})+{\tau }^{y}{\sigma }^{x}\sin ({k}_{y})]. $$

Equation (4) breaks *M*_*z*_, $$ {\mathcal{I}} $$, and $$ {\mathcal{T}} $$ while preserving the unitary symmetries of *p*4*m* and the magnetic antiunitary symmetries $$ {M}_{z}\times {\mathcal{T}} $$ and $$ {\mathcal{I}}\times {\mathcal{T}} $$, the latter of which continues to enforce a twofold band degeneracy (Fig. 2l). The new Hamiltonian $$ {\mathcal{H}}({\bf{k}})+U({\bf{k}}) $$ (Eqs. (1) and (4)) therefore has the symmetry of magnetic layer group $$ p4/m^{\prime} mm $$, a supergroup of *p*4*m*. We note that because *U*(**k**) preserves two orthogonal mirrors, *M*_*x*,*y*_, it cannot be induced by a constant Zeeman field alone, and must instead come from several internal magnetic moments or applied quadrupolar magnetism. An example of a configuration of spin-1/2 magnetic moments is shown in Fig. 2a, that like *U*(**k**), lowers the symmetry of $$ p4/mmm1^{\prime} $$ to $$ p4/m^{\prime} mm $$. When Eq. (4) is added to Eq. (1), the surface states and Wilson spectrum gap (Fig. 2m, n), but gapped, SSH-like states remain bound to the 1D edges^23,24^ (Supplementary Note 11). By projecting onto one of the eigenstates of $$ {\mathcal{W}} $$ (for example the lower Wilson band in Fig. 2n), a second, nested Wilson loop can be computed in the *y* direction, and displays a nested Berry phase *θ*_2_ of 0 (*π*) if this magnetic insulator is in a trivial (quadrupole) phase^23^. For all nonzero values of *u* in Eq. (4), transitions between QI and trivial phases occur when the bulk gap closes at Γ(*M*) for 2*t*_1_ = −(+)*v*_*m*_, with $$ | \frac{{v}_{m}}{{t}_{1}}| <(> )2 $$ characterizing the QI (trivial) phase. As we show in Supplementary Note 2, the Hamiltonian $$ {\mathcal{H}}({\bf{k}})+U({\bf{k}}) $$ (Eqs. (1) and (4)) is topologically equivalent to the quadrupole model introduced in ref. ^23^. We can also choose to reintroduce $$ {V}_{{M}_{z}}({\bf{k}}) $$ (Eq. (3)) to Eqs. (1) and (4), which, as it is invariant under *p*4*m*, will preserve the QI phase if it does not close a bulk or edge gap, even though it breaks the combined magnetic symmetries $$ {M}_{z}\times {\mathcal{T}} $$ and $$ {\mathcal{I}}\times {\mathcal{T}} $$ in $$ p4/m^{\prime} mm $$, the (magnetic) layer group of Eqs. (1) and (4). For weak $$ {v}_{{M}_{z}}$$ this therefore results in a QI phase in *p*4*m* with singly degenerate bands, and for stronger values, it can induce a crystalline semimetallic phase (Supplementary Note 1). It also follows from the theory of band representations^22,35,36^ that the QI phase of Eqs. (1) and (4) with (without) Eq. (3) is an obstructed atomic limit^22^ with the two occupied Wannier orbitals shifted to the 1*b* Wyckoff position of *p*4*m* ($$ p4/m^{\prime} mm $$) (Supplementary Note 3).

In Supplementary Notes 6–10, we construct a microscopic picture of the phase transitions between the TCI, fragile, and QI phases of the tight-binding Hamiltonians given by Eq. (1) with the potentials in Eqs. (3) and (4). We also analytically examine the phase transition between a tight-binding model of a ${p}_{z}-{d}_{{x}^{2}-{y}^{2}}$-hybridized 2D TI (Supplementary Note 5) and an additional model of QI in $p4/m^{\prime} mm$ that is distinct from (but topologically equivalent to) Eqs. (1) and (4). Specifically in Supplementary Notes 7 and 8, we derive the low-energy *k* ⋅ *p* theories of ${p}_{z}-{d}_{{x}^{2}-{y}^{2}}$- and *s*–*p*_*z*_-hybridized 2D TIs whose atoms lie at the 1*a* position of $p4/mmm1^{\prime}$, and analytically solve for the bound states on their corners when their edge states are gapped with *p*4*m*-symmetric magnetism. We find that the *p*–*d*- (*s*–*p*-) hybridized TI evolves into a QI (trivial insulator) when *p*4*m*-symmetric magnetism is introduced, precisely because the inverted bands exhibit different (the same) Kramers pairs of *C*_4*z*_ eigenvalues, such that the symmetry eigenvalues of the occupied bands (do not) match those of a QI in *p*4*m* (Supplementary Note 3). We then show in Supplementary Note 9 that the edge states of a ${C}_{{M}_{z}}=2$ TCI (such as the $s-{d}_{{x}^{2}-{y}^{2}}$-hybridized TCI phase of Eq. (1)) can gap under an *M*_*z*_-breaking, ${\mathcal{T}}$-symmetric potential (such as Eq. (3)) into four Kramers pairs of corner modes that, if the total system filling is fixed at 1∕2, are quarter- (three-quarters-), half-, or fully filled depending on the *C*_4*z*_ eigenvalues of the inverted bulk bands. We then demonstrate that the quarter-filled and three-quarters-filled cases evolve into QIs under *p*4*m*-preserving magnetism, also indicating that the *s*–*d*-hybridized TCI phase of Eq. (1), like the *p*–*d*-hybridized 2D TI in Supplementary Notes 5 and 7, can transition into a QI when its edge states are gapped with *p*4*m*-symmetric, *M*_*z*_-breaking magnetism. In Supplementary Note 9, we explain this by using TQC^22,35,36^ to show that the *s*–*d*-hybridized TCI phase of Eq. (1) exhibits the same quadrupole moment (modulo *e*) as a ${p}_{z}-{d}_{{x}^{2}-{y}^{2}}$-hybridized 2D TI (when their edge states are gapped by breaking *M*_*z*_ and ${\mathcal{T}}$).

### 3D Dirac semimetals with HOFA states

We now stack the previous 2D models into 3D to create physically motivated Hamiltonians modeling solid state materials that are equivalent to tuning cycles of Eqs. (1), (3), and (4) (Fig. 1f, g). In this work, we restrict focus to gapless tuning cycles, which are equivalent 3D topological semimetals. We begin constructing 3D models by stacking Eq. (1) in the *z* direction, adding a term (${t}_{H}{\tau }^{z}\cos ({k}_{z})$) that varies the gaps at *k*_*x*_ = *k*_*y*_ = 0, *π* as functions of *k*_*z*_, and adding Eq. (4) with a modulation governed by one of two distinct interlayer coupling terms:

5 $$ {{\mathcal{H}}}_{H1}({\bf{k}})={\mathcal{H}}({\bf{k}})+U({\bf{k}})+{t}_{H}{\tau }^{z}\cos ({k}_{z}), $$

6 $$ {{\mathcal{H}}}_{H2}({\bf{k}})={\mathcal{H}}({\bf{k}})+U({\bf{k}})\sin ({k}_{z})+{t}_{H}{\tau }^{z}\cos ({k}_{z}). $$

**Fig. 3:**
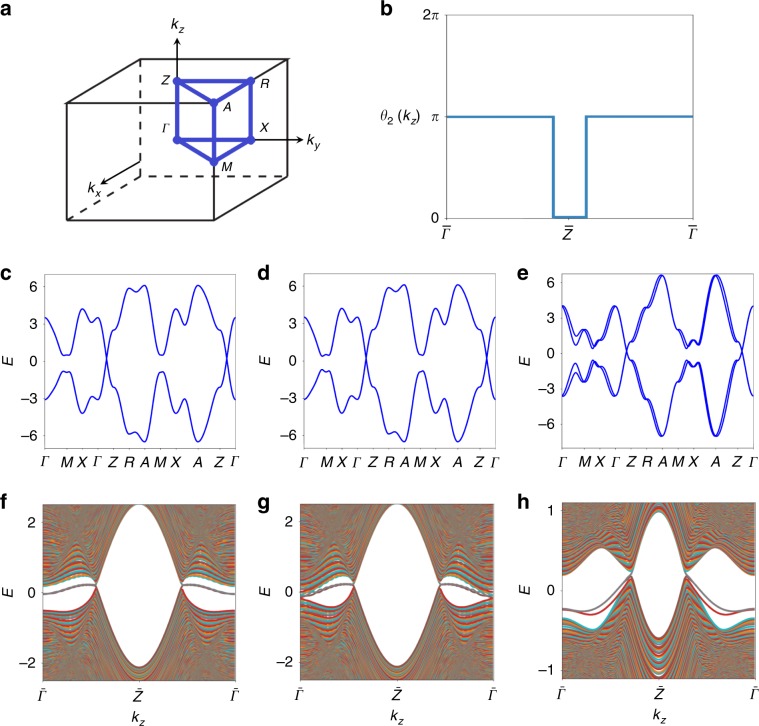
HOFA states in magnetic, nonmagnetic, and fragile topological Dirac semimetals. **a** The BZ, **c** bulk bands, and **f** hinge bands of a *z*-directed rod of a ${\mathcal{T}}$-broken 3D Dirac semimetal with HOFAs in magnetic SG $P4/m^{\prime} mm$ (Eq. (5)). The Hamiltonian of each *k*_*z*_ slice describes either a trivial insulator or a QI, and the bulk Dirac points occur at the quantum critical points between the two phases. **d** HOFAs can also be realized in a closely related ${\mathcal{T}}$-symmetric Dirac semimetal in SG 123 $P4/mmm1^{\prime}$ (Eq. (6)). **g** Here, the Hamiltonian of the *k*_*z*_ = 0 plane is in the 2D TCI phase^10^ shown in Fig. 2f, g, and therefore there is no gap in the hinge-projected surface states at $\bar{\Gamma }$. **e** Upon breaking *M*_*z*_ and ${\mathcal{I}}$ symmetries by adding Eqs. (3)–(6), which reduces the symmetry to SG 99 ($P4mm1^{\prime}$), **h** the face TCI cones gap as they did previously in Fig. 2i–k, resulting in a noncentrosymmetric Dirac semimetal without surface states^21^ whose only topological boundary modes are HOFA states. **h** Two sets of weakly split HOFAs meet in Kramers pairs at $\bar{\Gamma }$; if the system filling is fixed to 1∕2 (i.e., to the filling of the Dirac points), then one set of HOFA states in **h** is half-filled and carries a topological quadrupole moment, and the other set is fully filled, and is topologically trivial. At $\bar{\Gamma }$, this implies that the Kramers pairs of hinge states are three-quarters filled, and that they exhibit the same quadrupole moment (modulo *e*) as a QI (Supplementary Note 9). The Hamiltonian of the *k*_*z*_ = 0 plane in **e**, **h** exhibits the same fragile topology as the 2D model in Fig. 2i–k (Supplementary Note 4), implying that the hinge states at $\bar{\Gamma }$ are equivalent to the fractionally charged corner modes of a 2D fragile TI. The model (Eqs. (6) and (3)) shown in **e**, **h** therefore represents a previously uncharacterized variant of topological semimetal that carries observable signatures of fragile topology. **b** The 3D semimetallic phases of Eqs. (5) and (6), whether or not Eq. (3) is additionally present, exhibit the same quadrupole-quantized nested Berry phase^23^ *θ*_2_(*k*_*z*_).

In addition to respecting the symmetries of magnetic SG *P*4*mm* (number 99.163 in the BNS notation^62^), the space group generated by adding translations in the *z* direction to^62^*p*4*m*, *H*_*H*1_(**k**) respects the antiunitary symmetries ${M}_{z}\times {\mathcal{T}}$ and ${\mathcal{I}}\times {\mathcal{T}}$, whereas *H*_*H*2_(**k**) individually respects *M*_*z*_, ${\mathcal{I}}$, and ${\mathcal{T}}$ (Table 1). To tune ${{\mathcal{H}}}_{H1,2}({\bf{k}})$ into 3D Dirac semimetal phases, we invert bands by setting *v*_*m*_ < 0, *t*_1_ > 0, and tuning *t*_*H*_. When ∣*t*_*H*_∣ > 2*t*_1_ + *v*_*m*_, a pair of Dirac points forms along the Γ*Z* line (Fig. 3c). Viewing ${\mathcal{H}}({k}_{x},{k}_{y})$ on each constant-*k*_*z*_ slice of the 3D BZ as a 2D system, these Dirac points are equivalent to the critical point between trivial and QI phases (Supplementary Note 5). To see this, note that the Dirac points are formed by inverting bands with different pairs of *C*_4*z*_ eigenvalues in a 3D BZ for which slices indexed by *k*_*z*_ are invariant under magnetic supergroups of *p*4*m* (Supplementary Note 5). For both Eqs. (5) and (6), the QI-nontrivial BZ slices are identified by the bulk nested Wilson loop^23^ parameterized as a function of *k*_*z*_ (Fig. 3b). When ∣*t*_*H*_∣ is further increased beyond 2*t*_1_ − *v*_*m*_, an additional pair of Dirac points forms along *MA*; we analyze the HOFA-state structure of this semimetal in Supplementary Note 5. We note that similar results were obtained in ref. ^53^ in toy models of magnetic Dirac semimetals. However, in this work, we also uniquely discover HOFA states in ${\mathcal{T}}$-symmetric Dirac and Weyl semimetals, allowing their prediction in real materials, which we will address shortly.

We first search for HOFA states in the 3D Dirac semimetal phase of ${{\mathcal{H}}}_{H1}({\bf{k}})$ (Eq. (5)) that only exhibits a pair of Dirac points along Γ*Z* (specific parameters are listed in Supplementary Note 1). In Eq. (5), ${\mathcal{T}}$ symmetry is broken, and therefore the 2D Hamiltonians of all *k*_*z*_-indexed BZ planes (including *k*_*z*_ = 0, *π*) describe either trivial insulators or QIs. Calculating the hinge and surface states of the Dirac semimetal phase of Eq. (5) in a rod geometry that is finite in the *x* and *y* directions (Fig. 3f), we observe the absence of 2D surface states and the presence of HOFAs spanning the projections of the bulk 3D Dirac points along the 1D hinges. If the bulk Dirac points are gapped by breaking *C*_4*z*_ while preserving mirror symmetry, the HOFA states can evolve into the chiral hinge modes of a 3D (magnetic) higher-order TI (axion insulator)^24,32,37^. Though ${{\mathcal{H}}}_{H1}({\bf{k}})$ provides the simplest realization of a HOFA Dirac semimetal without surface states, it also requires the complicated mirror-preserving magnetism of magnetic SG $P4/m^{\prime} mm$ (123.341 in the BNS notation^62^), which cannot be realized in a constant external field or with ferromagnetism. As the number of known magnetic structures is small compared with the number of known materials^67^, it is difficult to identify material candidates for the magnetic HOFA Dirac semimetal phase of ${{\mathcal{H}}}_{H1}({\bf{k}})$. However, we do find that the antiferromagnetic phase of the Dirac semimetal CeSbTe in magnetic space group *P*_*c*_4 ∕ *ncc* (130.432 in the BNS notation^62^) is closely related^68^, and may exhibit HOFA states (Supplementary Note 12).

Fortunately, we discover that topological HOFA states are also present in ${\mathcal{T}}$-symmetric Dirac semimetals. To see this, we tune ${{\mathcal{H}}}_{H2}({\bf{k}})$ (Eq. (6)) into the parameter regime 2*t*_1_ + *v*_*m*_ < ∣*t*_*H*_∣ < 2*t*_1_ − *v*_*m*_ (specific parameters for Fig. 3d, g are detailed in Supplementary Note 1) to realize a ${\mathcal{T}}$-symmetric Dirac semimetal in SG 123 ($P4/mmm1^{\prime}$) with a time-reversed pair of Dirac points along Γ*Z* and with mirror Chern number ${C}_{{M}_{z}}=2$ (0) at *k*_*z*_ = 0 (*π*). As with the magnetic Dirac semimetal phase of ${{\mathcal{H}}}_{H1}({\bf{k}})$ (Eq. (5)), the bulk bands of ${{\mathcal{H}}}_{H2}({\bf{k}})$ (Fig. 3d) are twofold degenerate throughout the BZ as a consequence of ${\mathcal{I}}\times {\mathcal{T}}$ symmetry (Table 1). Crucially, while this 3D model (${{\mathcal{H}}}_{H2}({\bf{k}})$ in Eq. (6)) is ${\mathcal{T}}$-symmetric, 2D planes of the BZ indexed by *k*_*z*_ ≠ 0, *π* are still invariant under magnetic layer group $p4/m^{\prime} mm$, and thus can still be topologically equivalent to QIs. In the Dirac semimetal phase of ${{\mathcal{H}}}_{H2}({\bf{k}})$, there are two kinds of topological boundary modes: mirror TCI cones on *M*_*z*_-preserving 2D faces at *k*_*z*_ = 0, and singly-degenerate HOFAs on each of the four 1D hinges connecting the projections of the TCI cones to those of the bulk Dirac points (Fig. 3g). Furthermore, we recognize that $U({\bf{k}})\sin ({k}_{z})$ in Eq. (6), which acts in each *k*_*z*_ ≠ 0, *π* BZ slice like the *p*4*m*-symmetric magnetism (Eq. (4)) depicted in Fig. 2a, is also equivalent to the bulk spin-orbit term previously introduced in ref. ^21^ to destabilize the surface Fermi arcs of a Dirac semimetal.

As with the 2D TCI in Fig. 2j, the (100)-surface states of the TCI-nontrivial plane at *k*_*z*_ = 0 of the 3D Dirac semimetal phase of ${{\mathcal{H}}}_{H2}({\bf{k}})$ can be gapped by breaking *M*_*z*_ while preserving ${\mathcal{T}}$. We accomplish this by adding ${V}_{{M}_{z}}({\bf{k}})$ in Eq. (3) to Eq. (6); this breaks *M*_*z*_ and ${\mathcal{I}}$ while preserving *p*4*m*, ${\mathcal{T}}$, and *z*-direction lattice translations, lowering the overall symmetry to SG 99 $P4mm1^{\prime}$ (Table 1). In Fig. 3e, h, we respectively plot the bulk and hinge bands of the noncentrosymmetric Dirac semimetal phase resulting from adding Eq. (3) to Eq. (6). We observe that the previous mirror TCI surface states of Eq. (6) have become split and, instead, there are four hinge-localized Kramers pairs of states at $\bar{\Gamma }$ in Fig. 3h. These eight states become weakly split into two sets of HOFA states at *k*_*z*_ ≠ 0; as described in Supplementary Note 9, if we fix the overall system filling to 1/2 (i.e., to the filling of the Dirac points), then one of the sets of four HOFA states in Fig. 3h is half-filled and carries a topological quadrupole moment, and the other set is fully filled, and is topologically trivial. This implies that the Kramers pairs of hinge states at $\bar{\Gamma }$ in Fig. 3h are three-quarters-filled and exhibit the same quadrupole moment (modulo *e*) as a QI (Supplementary Note 9). In this noncentrosymmetric Dirac semimetal phase (Eqs. (3) and (6)), the Hamiltonian of the *k*_*z*_ = 0 plane exhibits the same fragile topology as the 2D insulator in Fig. 2l–n, and the anomalous, fractionally charged Kramers pairs of states on each hinge at *k*_*z*_ = 0 represent an observable signature of the fragile bands (or of an obstructed atomic limit that can be decomposed into the sum of fragile bands and unobstructed atomic limits^37^) (Supplementary Note 9). Therefore, the noncentrosymmetric Dirac semimetal phase of Eqs. (3) and (6) represents a previously uncharacterized fragile topological variant of Dirac semimetal. Like the 3D HOTIs (axion insulators) analyzed in ref. ^37^, we refer to this variant of Dirac semimetal (Eqs. (3) and (6)) as fragile because its minimal realization is equivalent to a tuning cycle between a 2D fragile TI with anomalous corner modes and a 2D insulator with a trivial Wilson and corner spectrum. Specifically, because the 3D Dirac semimetal phase of Eqs. (3) and (6) respects fourfold rotation and ${\mathcal{T}}$ symmetries, then the appearance of quarter-empty (or -filled) Kramers pairs of hinge states at $\bar{\Gamma }$ (where the overall system filling is fixed to the filling of the Dirac points) indicates that the occupied bands at *k*_*z*_ = 0 contain the fragile valence bands of the 2D model described by Eqs. (1) and (3). This occurs because the band inversion that creates the Dirac points along Γ*Z* in Eqs. (3) and (6) also drives the Hamiltonian of the *k*_*z*_ = 0 plane to exhibit the same *C*_4*z*_ eigenvalues as a QI in *p*4*m* (Supplementary Note 9). While not every fragile phase exhibits intrinsic (anomalous) corner modes (for example, two superposed copies of the ${\mathcal{I}}$-symmetric fragile TIs examined in refs. ^32,37^ combine to form an insulator that is also fragile, but one without anomalous corner states), our results further imply that specific corner states (or state counting imbalances, as discussed in Supplementary Note 9) can still represent a robust signature of a valence manifold that can be decomposed into the sum of unobstructed (trivial) atomic limits and fragile bands, when crystal symmetries and band connectivity are taken into account.

Alternatively, we can formulate a model of a 3D Dirac semimetal from hybridized layers of *p*_*z*_ and ${d}_{{x}^{2}-{y}^{2}}$ orbitals in which the Hamiltonian of the *k*_*z*_ = 0 plane instead characterizes a 2D TI^38^, as occurs in the experimentally confirmed Dirac semimetals^10,18,20,56,57^ Cd_3_As_2_ and^19^ Na_3_Bi. To realize HOFAs as the only boundary (surface and hinge) modes in a semimetal with TI surface cones, unlike with the Dirac semimetal phase of ${{\mathcal{H}}}_{H2}({\bf{k}})$ (Eq. (6)), one must break ${\mathcal{T}}$ symmetry (Supplementary Note 7), or apply strain to drive additional band inversions (Supplementary Note 5). In Supplementary Note 5, we also present a model of a *p*–*d*-hybridized Dirac semimetal with coexisting TI surface states and HOFA hinge states. We also note that the three ${\mathcal{T}}$-symmetric semimetal models presented in this work—the TCI-nontrivial^10^ Dirac semimetal phase of Eq. (6), the fragile topological Dirac semimetal phase of Eqs. (3) and (6), and the *p*–*d*-hybridized Dirac semimetal in Supplementary Note 5—all exhibit the same number of half-filled HOFA states at *k*_*z*_ ≠ 0 (where the system filling is fixed to the filling of the bulk Dirac points), despite displaying differing numbers of gapped surface states at *k*_*z*_ ≠ 0, *π*. This reinforces the notion that the surface states of Dirac semimetals are not themselves a topological consequence of the bulk Dirac points, but rather only appear due to the topology of high-symmetry planes, and are not required to connect to the surface projections of the bulk Dirac points^21^.

### Material realizations

**Fig. 4:**
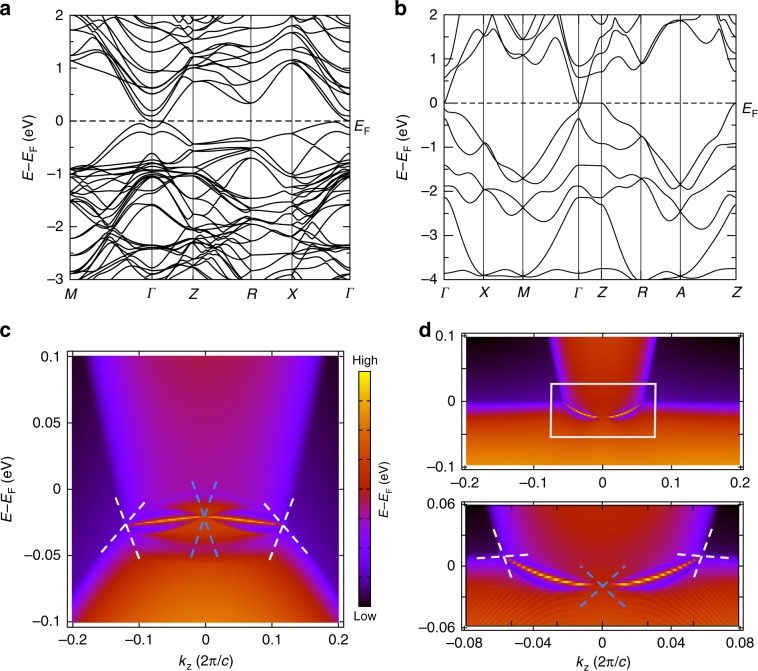
HOFA states in *α″*-Cd_3_As_2_ and KMgBi. **a** Bulk bands incorporating the effects of SOC of *α″*-Cd_3_As_2_ in SG 137 ($P{4}_{2}/nmc1^{\prime}$)^10,18,20,56,57^. This semimetal exhibits ${\mathcal{T}}$-reversed pairs of bulk 3D Dirac cones as well as 2D TI cones on its faces as a consequence of the nontrivial ${{\mathbb{Z}}}_{2}$ topology of the *k*_*z*_ = 0 plane (Supplementary Note 13)^10,18–20^. **c** The hinge spectrum of the *k* ⋅ *p* model of *α″*-Cd_3_As_2_ introduced in ref. ^18^ exhibits previously undetected HOFA states connecting the projections of the bulk Dirac cones (white) to the hinge projection of the topological face cones (blue). **b** Bulk bands incorporating the effects of SOC and (**d**) hinge states of the candidate tilted Dirac semimetal KMgBi in SG 129 ($P4/nmm1^{\prime}$)^58,59^. **d** Zooming into the green boxed region, HOFAs are clearly visible connecting the projections of the bulk, tilted 3D Dirac points (white) to the projections of surface 2D TI cones (blue). The bulk band structures in (**a**) and (**b**) were obtained from first-principles, and then used to fit tight-binding models whose hinge Green's functions are shown in (**c**) and (**d**), respectively (Supplementary Note 13).

Most surprisingly, the Dirac points in Fig. 3 display the same *k* ⋅ *p* Hamiltonian as the bulk nodes^10,18^ in the centrosymmetric structural (*α* and *α″*) phases of the experimentally established Dirac semimetal^18,20,56^ Cd_3_As_2_ (Fig. 4a). This is because ${{\mathcal{H}}}_{H2}({\bf{k}})$ in Eq. (6), which respects symmorphic SG 123 $P4/mmm1^{\prime}$, and Cd_3_As_2_ in its room- (high-) temperature *α* (*α″*) phase, which respects nonsymmorphic SG 137 $P{4}_{2}/nmc1^{\prime}$ (SG 142 $I{4}_{1}/acd1^{\prime}$), have little groups along their respective *k*_*x*_ = *k*_*y*_ = 0 lines with isomorphic unitary subgroups (Fig. 3a and Supplementary Notes 5 and 12). Both the *α* and *α″* structural phases of Cd_3_As_2_ exhibit the same bulk topology—they both host a time-reversed pair of Dirac points along *k*_*x*_ = *k*_*y*_ = 0, and are equivalent at *k*_*z*_ = 0 to 2D TIs due to a band inversion between the 5*s* orbitals of Cd and the *m*_*j*_  = ±3 ∕ 2 subset of the 4*p*_*x*,*y*_ orbitals of As^18^. In terms of the *s*–*p*- and *s*–*d*-hybridized semimetals and TIs analyzed in Supplementary Notes 5–9, the topology of the *α* and *α″* structural phases of Cd_3_As_2_ can be understood by noting that the *m*_*j*_ = ±3 ∕ 2 subset of spinful *p*_*x*,*y*_ orbitals exhibits the same parity eigenvalues as spinful *p*_*z*_ orbitals and the same fourfold rotation eigenvalues as spinful ${d}_{{x}^{2}-{y}^{2}}$ orbitals^22^. This implies that the bulk topology of Cd_3_As_2_ (Supplementary Note 13) is equivalent to the superposition of an *s*–*p*_*z*_-hybridized 3D TI and an $s-{d}_{{x}^{2}-{y}^{2}}$-hybridized topological Dirac semimetal with HOFA states (or equivalently, to the ${p}_{z}-{d}_{{x}^{2}-{y}^{2}}$ HOFA Dirac semimetal in Supplementary Note 5). Using an analytic formulation of topological (intrinsic) HOFA states derived in Supplementary Notes 6–10, we find that the *k* ⋅ *p* theory and symmetries of *α*-Cd_3_As_2_ imply the presence of HOFA states on the hinges of (001)- (*z*-) axis-directed samples, which have recently been synthesized in experiment^69^. Though the *α* phase is body-centered and respects *x*- and *y*-normal glide reflections, instead of *M*_*x*,*y*_ like Eq. (6), we provide proofs in Supplementary Note 12 demonstrating that body-centered and glide-symmetric Dirac semimetals also exhibit topological HOFA states like those in Fig. 3f–h.

The symmetries and *k* ⋅ *p* theory of the Dirac points of Eq. (6) additionally imply that the primitive tetragonal (*α″*) phase of Cd_3_As_2_ should also exhibit topological HOFA states. Although the *α″* phase naturally occurs at high temperatures (475–600 ^∘^C)^56^, it can be stabilized in single crystalline form at room temperature and below by 2% zinc doping^57^; as Zn is isoelectronic to Cd, this doping will not affect the Fermi level. Calculating the hinge spectrum of the original *k* ⋅ *p* model introduced in ref. ^18^ for *α*″-Cd_3_As_2_, we confirm our prediction of previously overlooked HOFAs (Fig. 4c and Supplementary Note 13). This suggests a clear route towards predicting additional candidate Dirac semimetals with HOFA states: using the low-energy theory of the QI (Supplementary Note 6), we determine that strong-SOC Dirac semimetals with SGs that contain point group 4*mm* (*C*_4*v*_) will exhibit HOFA states when they are cut into nanorods or exhibit step edge configurations that preserve fourfold axes (Table 2). This is analogous to the helical hinge modes in the HOTI bismuth, which are only observable in samples that are cut into nanowires (or terminated with step edge configurations) that preserve bulk rotation and ${\mathcal{I}}$ symmetries^31^. A number of candidate Dirac semimetals have already been identified in the SGs in Table 2, including the aforementioned *α* and *α″* phases of Cd_3_As_2_, the rutile-structure ($\beta ^{\prime}$-) phase of PtO_2_ in SG 136 ($P{4}_{2}/mnm1^{\prime}$)^60,61^, and families of tilted Dirac semimetals related to VAl_3_ in SG 139 ($I4/mmm1^{\prime}$)^70^, YPd_2_Sn in SG 225 ($Fm\bar{3}m1^{\prime}$)^71^, and KMgBi in SG 129 ($P4/nmm1^{\prime}$)^58,59^.

**Table 2:** Space groups (SGs) that admit Dirac points with HOFA states derived from the QI introduced in ref. ^23^.

Space groups admitting dirac points with HOFA states		
Point group name	Point group symbol	SG numbers
*C*_4*v*_	$4mm1^{\prime}$	99–110
*D*_4*h*_	$4/mmm1^{\prime}$	123–142
*O*_*h*_	$m\bar{3}m1^{\prime}$	221–230

All of these SGs have point groups that contain C4*v*. We obtain this list of SGs by combining the nested Jackiw–Rebbi formulation of the QI in Supplementary Note 6 with an analysis of the crystallographic rod groups in Supplementary Note 12. In all of these SGs, semimetals with Dirac points along lines with 4 *mm* or $4/m^{\prime} mm$ symmetry will exhibit intrinsic HOFA states when cut into nanorods that preserve fourfold axes and are thick compared with the in-plane lattice spacing. This list is a complete enumeration of the SGs that permit Dirac semimetals with HOFA states directly related to the QI introduced in ref. ^23^; alternative realizations of Dirac and Weyl semimetals with HOFA states derived from other 2D magnetic insulators with corner states are also possible (refs. ^32,37^ and Supplementary Note 12).

Of the candidate HOFA semimetals that we identified, we highlight KMgBi and *β*′-PtO_2_ because of their simple geometries. KMgBi has recently been identified as a topological semimetal with critically tilted bulk Dirac cones^58^, and its electronic properties have been examined in experiment^59^. In Fig. 4, we plot the bulk bands (b) calculated from first principles, and the hinge spectrum (d) of a lattice tight-binding model of KMgBi fit to the bands in (b) (Supplementary Note 13). We find that the *k*_*z*_ = 0 plane of KMgBi exhibits the topology of a 2D TI (Supplementary Note 13), in agreement with the surface-state calculation in ref. ^58^. In the vicinity of *k*_*z*_ = 0 (Fig. 4c), HOFAs are clearly visible connecting the hinge projections of the bulk 3D Dirac points (white) to the projections of 2D surface TI cones at *k*_*z*_ = 0 (blue). This boundary mode structure is captured by the model of a *p*–*d*-hybridized Dirac semimetal in Supplementary Note 5.

**Fig. 5:**
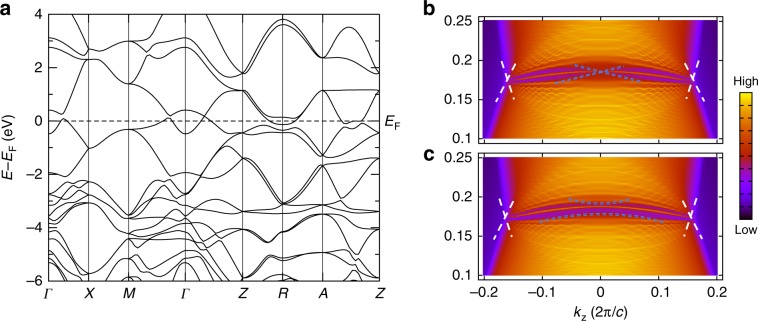
HOFA states and fragile Dirac semimetal phase in *β*′-PtO_2_. **a** Bulk bands incorporating the effects of SOC of the candidate Dirac semimetal *β*′-PtO_2_ in SG 136 ($P{4}_{2}/mnm1^{\prime}$)^60,61^. Unlike in the Dirac semimetals *α″*-Cd_3_As_2_ and KMgBi examined in Fig. 4, the *k*_*z*_ = 0 plane of *β*′-PtO_2_ is equivalent to a 2D TCI with mirror Chern number ${C}_{{M}_{z}}=2$ (ref. ^61^ and Supplementary Note  13). **b** The hinge spectrum of *β*′-PtO_2_ exhibits two narrowly split HOFA states connecting the hinge projections of the bulk 3D Dirac points (white) to the projections of two surface TCI cones at *k*_*z*_ = 0 (blue). Fixing the system filling to that of the bulk Dirac points, we find that the lower HOFA state in energy in (**b**) is half-filled, and therefore exhibits a topological quadrupole moment, and the higher state is unoccupied, and is thus topologically trivial, as discussed in Supplementary Notes 9 and 13. **c** In the presence of a *z*-directed external electric field, the surface TCI cones in *β*′-PtO_2_ become gapped (blue), allowing for the two HOFA states to meet at *k*_*z*_ = 0 in a quarter-filled Kramers pair of corner modes that is characteristic of the fragile phase introduced in this work (Figs. 2i–k, 3e, h, and Supplementary Notes 4, 9, and 13). The bulk band structure in (**a**) was obtained from first-principles, and then used to fit a tight-binding model whose hinge Green's functions in the absence and presence of an external electric field are shown in (**b**) and (**c**), respectively (Supplementary Note 13).

In Fig. 5, we also examine the bulk and hinge spectra of the candidate Dirac semimetal *β*′-PtO_2_. Single crystals of PtO_2_ in its rutile-structure ($\beta ^{\prime}$) phase have previously been prepared in experiment^60^, and its bulk and surface electronic structure were examined in a previous theoretical work^61^. In *β*′-PtO_2_, the Hamiltonian of the *k*_*z*_ = 0 plane is equivalent to a 2D TCI with mirror Chern number ${C}_{{M}_{z}}=2$; therefore *β*′-PtO_2_ is more closely related to the *s*–*d*-hybridized HOFA semimetal model introduced in this work (Eq. (6)) than it is to *α″*-Cd_3_As_2_ and KMgBi, which at *k*_*z*_ = 0 are instead equivalent to 2D TIs (Fig. 4 and Supplementary Note 13). In Fig. 5, we plot the bulk bands (a) and hinge spectrum (b) of *β*′-PtO_2_ calculated from first principles, as detailed in Supplementary Note 13. In the spectrum of a single hinge (Fig. 5b), we observe two narrowly split HOFA states connecting the hinge projections of the bulk 3D Dirac points to the projections of the surface 2D TCI cones. Fixing the system filling to that of the bulk Dirac points, we observe that, similar to the hinge spectrum of the fragile topological Dirac semimetal in Fig. 3h, only one of the HOFA states on each hinge of *β*′-PtO_2_ is half-filled. Specifically, we find that the lower HOFA state in energy in Fig. 5b is half filled, and therefore carries a topological quadrupole moment (Supplementary Note 9), and that the other HOFA state is unoccupied, and is therefore topologically trivial.

Because the TCI surface cones in *β*′-PtO_2_ are only protected by *M*_*z*_ symmetry, they can be gapped without breaking ${\mathcal{T}}$ symmetry, unlike the 2D TI surface cones in *α″*-Cd_3_As_2_ and KMgBi (Supplementary Note 7). To preserve the bulk Dirac points and intrinsic HOFA states in *β*′-PtO_2_ while gapping the surface TCI cones, we must break *M*_*z*_ (and hence ${\mathcal{I}}$) symmetry while preserving the 4_2_ screw, *x*-directed *n*-glide reflection, ${\mathcal{T}}$, and lattice translation symmetries of SG 136 ($P{4}_{2}/mnm1^{\prime}$). Though this cannot be accomplished by uniaxial strain, which either manifests as symmetry-preserving stretching in the *z* direction or as a translation-breaking strain gradient, these symmetry requirements can be satisfied in experiment by applying an external electric field that is spatially constant (or slowly varying on the scale of the lattice spacing) along the *z*- (*c*-) axis of a fourfold-symmetric *β*′-PtO_2_ sample. Implementing the effects of an external electric field into our Green’s function calculation of the hinge states in *β*′-PtO_2_ (Fig. 5c), we observe that the TCI cones have become gapped, and that the HOFA states instead meet in a Kramers pair of quarter-filled corner modes at *k*_*z*_ = 0.

Furthermore, because the *k*_*z*_ = 0 plane of *β*′-PtO_2_ both exhibits the topology of a ${C}_{{M}_{z}}=2$ TCI and carries the same bulk fourfold rotation eigenvalues as a QI in *p*4*m* (Supplementary Note 3), then the quarter-filled corner modes that appear at *k*_*z*_ = 0 in the hinge spectrum of *β*′-PtO_2_ when its TCI surface states are gapped with an external electric field (Fig. 5c) indicate that the valence manifold at *k*_*z*_ = 0 can be separated into trivial bands and fragile bands with the same topology as the 2D fragile phase introduced in this work (Eqs. (1) and (3)). Specifically, *β*′-PtO_2_ only differs from a trivial (unobstructed) atomic limit without Dirac points or hinge states by a single inversion at the Γ point between bands with the same parity eigenvalues and different fourfold rotation eigenvalues (Fig. 5a and Supplementary Note 13). Therefore, the band inversion in *β*′-PtO_2_ drives the *k*_*z*_ = 0 plane into the same ${C}_{{M}_{z}}=2$ TCI phase as that of Eq. (1), which necessarily gaps into an insulator with fragile bands and fractionally charged Kramers pairs of corner modes when *M*_*z*_ is relaxed while preserving fourfold rotation and ${\mathcal{T}}$ (Supplementary Note 9). Whether the entire valence manifold at *k*_*z*_ = 0 is fragile or an obstructed atomic limit depends on the precise details of the bands below the Fermi energy, and for the case of the fragile phase introduced in this work (Eqs. (1) and (3)), uniquely cannot be inferred from the symmetry eigenvalues of the occupied bands (Supplementary Note 4), unlike the fragile phases examined in previous works^33–37^. Nevertheless, like the ${\mathcal{I}}$-symmetric fragile phases with corner modes introduced in refs. ^32,37^, the fragile phase of Eqs. (1) and (3) still exhibits anomalous (intrinsic) corner modes when trivial bands (i.e., unobstructed atomic limits without corner states) are introduced below the Fermi energy. Therefore, because the *k*_*z*_ = 0 plane of *β*′-PtO_2_ can be decomposed into a set of trivial bands without corner states and the inverted bands at the Fermi energy, it still exhibits the fractionally charged corner states shown in Fig. 5c when *M*_*z*_ is relaxed while preserving ${\mathcal{T}}$ and fourfold rotation, whether or not the entire valence manifold at *k*_*z*_ = 0 is fragile or an obstructed atomic limit. We draw further connection between *β*′-PtO_2_ and the model of an *s*–*d*-hybridized, noncentrosymmetric, fragile topological Dirac semimetal introduced in this work (Eqs. (3) and (6) and Fig. 3e, h) by noting that the quarter-filled corner modes at *k*_*z*_ = 0 in Fig. 5c represent the particle-hole conjugates of the three-quarters-filled fragile-phase corner modes observable at *k*_*z*_ = 0 in Fig. 3h (Supplementary Notes 9 and 13).

## Discussion

The HOFA states introduced in this work may be detectable through transport and STM experiments^31^. Though our analysis has focused on nanowire geometries, HOFA states may also be observable through momentum-resolved probes of fourfold-symmetric arrangements of step edges on the surfaces of Dirac semimetals with the SGs in Table 2. Nonlocal quantum oscillation experiments^72^ and SQUID measurements^31^ performed on materials with HOFA states are likely to show interesting signatures reflecting the reduced dimensionality of the hinge modes. By generalizing the analysis performed in this work, further examples of topological semimetals with HOFA states should be readily discoverable, including HOFA Dirac semimetals with sixfold symmetries and, as discussed in Supplementary Notes 10 and 12, high-fold-rotation Weyl semimetals with coexisting surface Fermi arcs and HOFA states. In addition, our atomic-orbital description of QIs with *s*–*d* hybridization suggests the possibility of quadrupolar generalizations of polyacetylene^16,43^. Finally, because the analytic expression that we obtain for the bound (corner) states of the QI in Supplementary Note 7, when the reflection symmetries of *p*4*m* are relaxed, can be expressed as the superposition of 1 + 2*n* (i.e., an odd number) of quadrupole moments whose direction is a free parameter but whose magnitude is fixed to *e*/2 (Supplementary Note 10), then it bears similarities with recent gauge-theory descriptions of fractons with anomalous tensor charges^73^.

## Methods

All tight-binding, surface state, hinge state, and Wilson loop calculations were performed using the standard implementation of the open-source PYTHTB Python package^74^. Nested Wilson loop calculations were performed using an extension of PYTHTB that is documented in ref. ^37^.

First-principles calculations were performed using the projector augmented wave^75^ method as implemented in the Vienna Ab initio Simulation Package^76,77^. The hinge states of *α″*-Cd_3_As_2_, KMgBi, and *β*′-PtO_2_ were obtained by mapping the bands closest to the Fermi energy to tight-binding models and then calculating the Green’s function along a single 1D hinge of a slab that was infinite along the crystal axis parallel to the hinge and respectively finite and semi-infinite along the two perpendicular axes. Further details of our first-principles and hinge Green’s function calculations are provided in Supplementary Note 13.

## Supplementary information

Supplementary Information

Peer Review File

## Data Availability

The data supporting the findings of this study are available within the paper and other findings of this study are available from the corresponding authors upon reasonable request. All first-principles calculations were performed using CIF structure files with the experimental lattice parameters, which can be obtained from the Inorganic Crystal Structure Database (ICSD)^78^ using the accession numbers provided in Supplementary Note  13.
