# Supplementary material for: Strong and fragile topological Dirac semimetals with higher-order Fermi arcs
Source: Nat Commun. 2020 Jan 31;11:627. doi: 10.1038/s41467-020-14443-5 (PMC6994491; doi:10.1038/s41467-020-14443-5)
Supplement: Supplementary file 1 — Supplementary Information [file 41467_2020_14443_MOESM1_ESM.pdf]

Supplementary Information for "Strong and Fragile Topological Dirac Semimetals with Higher-Order Fermi Arcs"

CONTENTS

Supplementary Figures 1

Supplementary Tables 18

Supplementary Notes 21
1. Tight-Binding Parameters for Figures 2 and 3 of the Main Text 21
2. Topological Equivalence of the Spinful s-d Hybridized Model and Spinless Flux-Threaded Model of Quadrupole Insulators 22
3. Quadrupole Insulators in Spinful Magnetic Wallpaper Group $p4m$ as Obstructed Atomic Limits 24
4. Fragile Topology in Wallpaper Group $p4m1'$ 29
5. Alternative Realizations of $\mathcal{T}$-Symmetric HOFA Semimetals 32
6. Summary of Supplementary Notes Detailing the Evolution of the 1D Edge States of 2D TIs to the Corner Modes of QIs and Fragile Topological Phases 37
7. Gapping the Edge Modes of a 2D Topological Insulator with Quadrupolar Magnetism 37
8. Gapping the Edge Modes of a 2D TI into an Magnetic Insulator with Zero Quadrupole Moment 47
9. Gapping the Edge Modes of a 2D TCI with $C_{M_z} = 2$ with Quadrupolar Magnetism 53
10. Relaxation of $M_{x,y}$ Symmetry in QIs and Related Fragile Phases 58
11. Numerical Investigations of the Surface States of HOFA Dirac Points 59
12. Space Groups Supporting Dirac Semimetals with Quadrupolar HOFA States 60
13. First-Principles Calculation Details 66

Supplementary References 72

SUPPLEMENTARY FIGURES

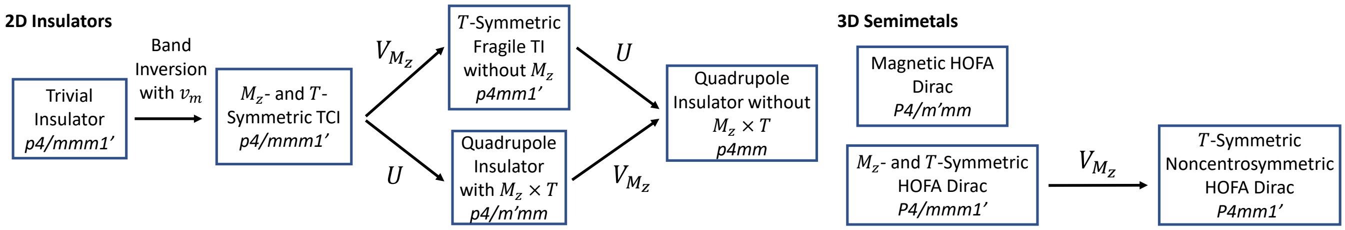

Supplementary Figure 1. Relationship between the phases of the 2D and 3D models introduced in this work. The 2D $\mathcal{T}$-symmetric Hamiltonian in SEq. (1) can be tuned between trivial and and topological crystalline insulating (TCI) phases. From the TCI phase, breaking M_z and keeping time-reversal ($\mathcal{T}$), accomplished by the introduction of $V_{M_z}(\mathbf{k})$ in Supplementary Equation (SEq.) (3), results in a phase with "fragile" topology[1–18]. Conversely, starting in the TCI phase and breaking $\mathcal{T}$ while keeping the product $M_z \times \mathcal{T}$, accomplished through the introduction of $U(\mathbf{k})$ in SEq. (4), results in a quadrupole insulating (QI) phase. By introducing the magnetic potential $U(\mathbf{k})$ into the fragile topological phase, or by introducing the $M_z \times \mathcal{T}$-breaking potential $V_{M_z}(\mathbf{k})$ into the QI in $p4/m'mm$, a QI can be realized in the type-I magnetic group[19,20] $p4mm$. In this work, this $M_z \times \mathcal{T}$-broken QI is realized in 2D Brillouin zone (BZ) planes indexed by $k_z \neq 0, \pi$ in Fig. 3(e,h) of the main text. In 3D, an $M_z \times \mathcal{T}$-symmetric magnetic Dirac semimetal with higher-order Fermi arcs (HOFAs) is realized by $\mathcal{H}_{H1}(\mathbf{k})$ (SEq. (5)), and a $\mathcal{T}$- and M_z-symmetric centro- ($\mathcal{I}$-) symmetric Dirac semimetal with HOFAs is realized by $\mathcal{H}_{H2}(\mathbf{k})$ (SEq. (6)). Finally, the $\mathcal{T}$- and M_z-symmetric HOFA semimetal phase of $\mathcal{H}_{H2}(\mathbf{k})$ (SEq. (6)) can be reduced to a noncentrosymmetric HOFA Dirac semimetal without surface states through the introduction of $V_{M_z}(\mathbf{k})$ in SEq. (3). For both the 2D and 3D phases, the layer and space groups are listed, respectively, using the expanded Shubnikov magnetic group notation[19,20]. The specific parameters used to realize the 2D and 3D phases highlighted in this work are listed in Supplementary Tables 1 and 2, respectively.

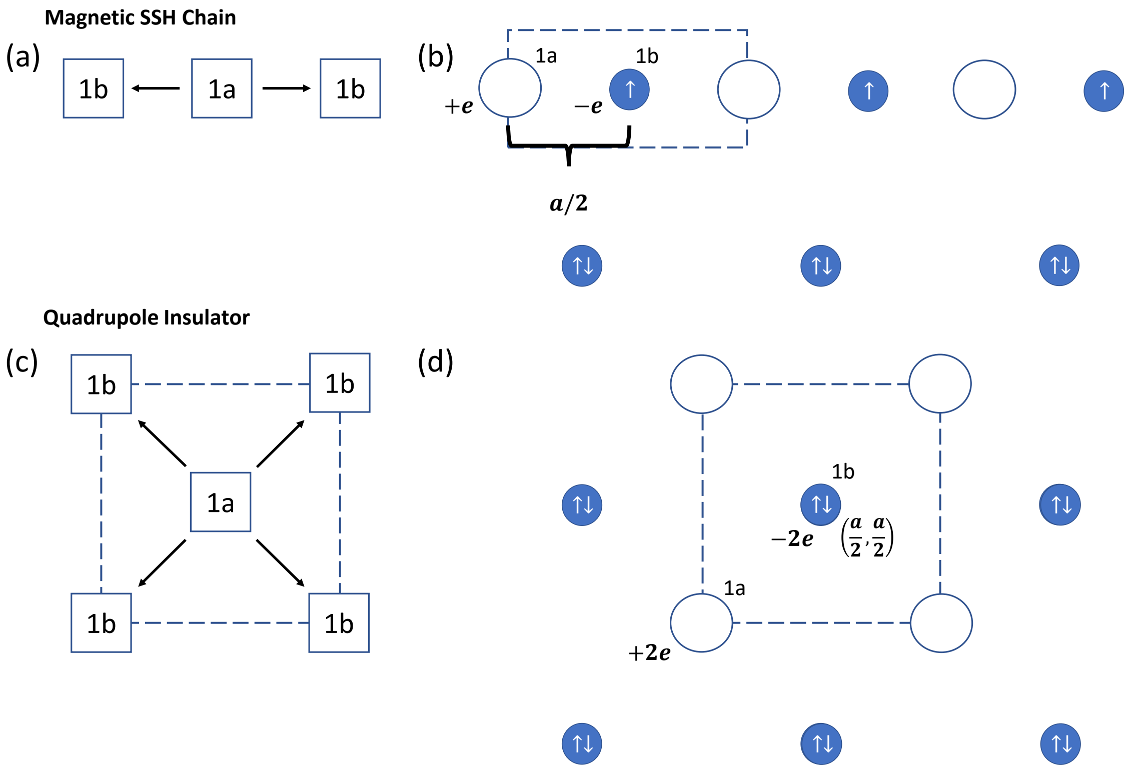

Supplementary Figure 2. Obstructed atomic limits and Wannier center homotopy in 1D and 2D. (a) The Wannier center homotopy for the spinful, $\mathcal{T}$-broken (magnetic) SSH chain[21,22] in magnetic rod group $(p\bar{1})_{\mathrm{RG}}$ (Supplementary References (SRefs.) 20 and 23 and Supplementary Note (SN) 12). In the trivial phase, one Wannier orbital at the $1a$ position is occupied. If a gap closes between two bands from spin-up s and p orbitals at $1a$, then there are sufficient Wannier orbitals to "slide" them out along the general position (x) to the $1b$ position in an $\mathcal{I}$-symmetric manner; the location x of the two Wannier centers is a gauge-dependent quantity[24,25]. (b) When the two orbitals are slid to the $1b$ position and the bulk gap is reopened, a Wannier description of just the lower band is again allowed. When the lower band is again occupied, the resulting state now exhibits a dipole moment (SEq. (47)) of $e/2$ (modulo e) per unit cell (dashed rectangle in (b)). (c) The Wannier center homotopy for a QI in magnetic wallpaper group $p4m$. In the trivial phase, two Wannier orbitals at the $1a$ position are occupied. If a gap closes between four bands from pairs of spinful s and d orbitals at the $1a$ position, then there are sufficient Wannier orbitals to "slide" them out along along the $4d$ position (Fig. 2(a) of the main text) to the $1b$ position in a $p4m$-invariant manner. When the gap is closed, the location of the four Wannier centers at $4d$ (x, x) again becomes a gauge-dependent quantity[24,25]. (d) When the four orbitals are slid to the $1b$ position and the gap between the lower two and upper two bands is reopened, a Wannier description of just the lower two bands is again allowed. When these lower two bands are occupied, the resulting state exhibits trivial x- and y-directed dipole moments (modulo e) (SEq. (50)), and a nontrivial xy quadrupole moment (SEq. (52)) of $e/2$ (modulo e) per unit cell (dashed square in (d)), in agreement with the value obtained for the spinless, flux-threaded QI introduced in SRef. 26.

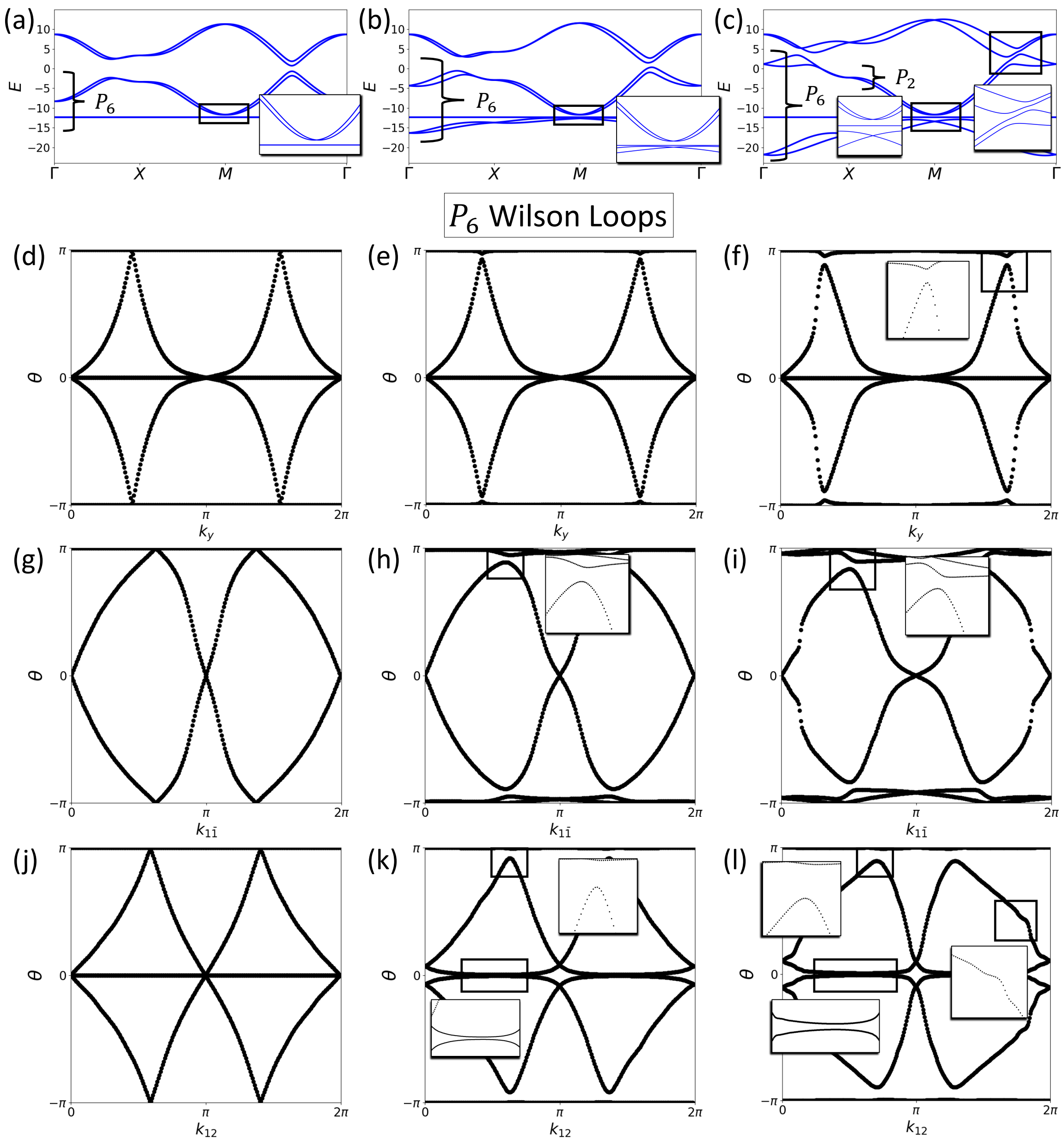

Supplementary Figure 3. Trivialized fragile-phase Wilson loops. (a-c) The bulk bands and (d-f) x-directed Wilson loop, (g-i) $(x+y)$-directed Wilson loop, and (j-l) $(2x+y)$-directed Wilson loop over the lower six bands (P_6) of $H_F(\mathbf{k})$ in SEq. (68) with additional Kramers pairs of s orbitals placed at the $2c$ position of wallpaper group $p4m1'$ (Fig. 2(a) of the main text) and coupled through SEq. (69). Figures are plotted for $v_C = 0, 4$, and 8 in SEq. (69), respectively. In the inset panels, we show narrowly avoided crossings in the energy and Wilson spectra. These three Wilson loops span the set of symmetry-inequivalent Wilson loops (where only one Wilson loop without edge-projecting symmetries[27], the $(2x+y)$-directed Wilson loop, is plotted for simplicity). We observe that the addition of $2c$ orbitals trivializes all of the six-band Wilson loops of this model, even though the bands from the $2c$ orbitals remain separated from the original two occupied bands (P_2 in (c)) by an energy gap, which is explicitly shown along high-symmetry lines in (b) and (c), and was additionally numerically confirmed throughout the BZ interior.

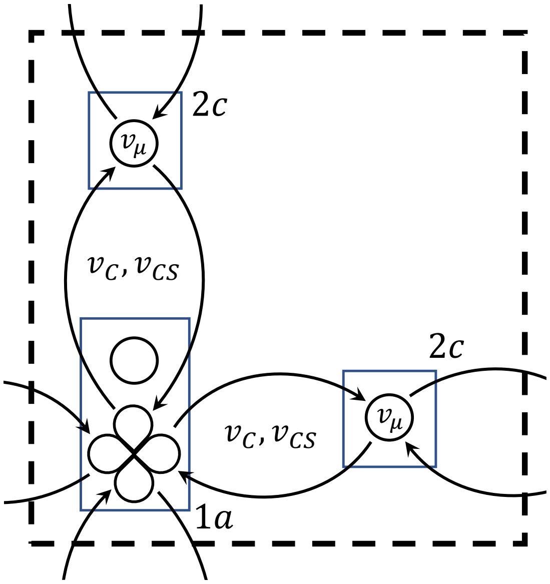

Supplementary Figure 4. Schematic of the hopping terms added in $V_C(\mathbf{k})$ (SEq. (69)) to trivialize the fragile valence bands F of SEq. (68) (SFig. 3). The symbols at each Wyckoff position of $\mathcal{T}$-symmetric wallpaper group $p4m1'$ represent Kramers pairs of spinful orbitals; there are two s and two d orbitals at $1a$, and two s orbitals at each site of the $2c$ position. The hopping terms v_C and v_{CS} in SEq. (69) couple the d orbitals at $1a$ to the s orbitals at $2c$; there are no terms that couple the s orbitals at $1a$ to the s orbitals at $2c$.

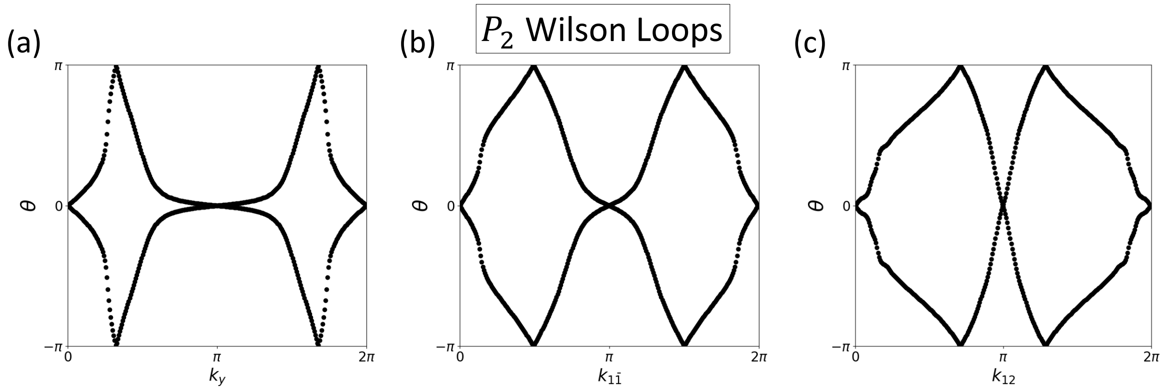

Supplementary Figure 5. Wilson loops of isolated fragile bands. (a) The x-directed, (b) $(x+y)$-directed, and (c) $(2x+y)$-directed Wilson loops over the fragile valence bands of SEq. (68) (P_2 in SFig. 3(c)), which remain separated from additional bands from the $2c$ position by an energy gap (this is shown along high-symmetry lines in SFig. 3(c), and was additionally numerically confirmed throughout the BZ interior). Figures are plotted using the same parameters as SFig. 3(c). All three Wilson loops (a-c) still wind[5], even though the Wilson loops over the lower 6 bands (P_6) have become trivialized by the additional trivial bands from the $2c$ position (SFig. 3(f,i,l)).

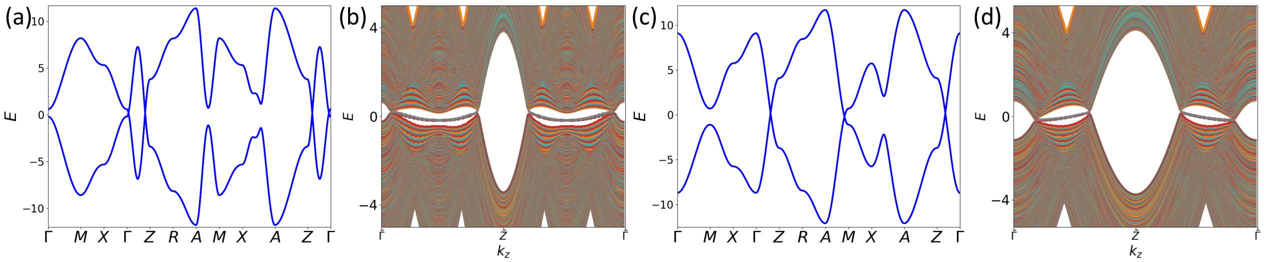

Supplementary Figure 6. Time-reversal-symmetric Dirac semimetals in tetragonal space group 123 $P4/mmm1'$ with four Dirac points. The transition between 2D trivial insulating and QI phases can be driven by band inversion at either $k_x = k_y = 0$ or at $k_x = k_y = \pi$ (SN 3 and SRefs. 26 and 28). Therefore, in a 3D system with BZ planes invariant under type-I magnetic wallpaper group $p4m$ or its supergroup $p4/m'mm$, such as a crystal in SG 123 $P4/mmm1'$, 3D Dirac points along *both* ΓZ and MA represent quadrupole transitions. Therefore, a z-directed rod (Fig. 1(a) of the main text) of either (a) a Dirac semimetal with two Dirac points along ΓZ (and two related by $\mathcal{T}$ symmetry along $Z\Gamma$) or of (c) a Dirac semimetal with one Dirac point along ΓZ and one along MA (in addition to their time-reversal partners), will exhibit (b,d) HOFAs spanning the projections of the 3D bulk Dirac points as its only boundary modes.

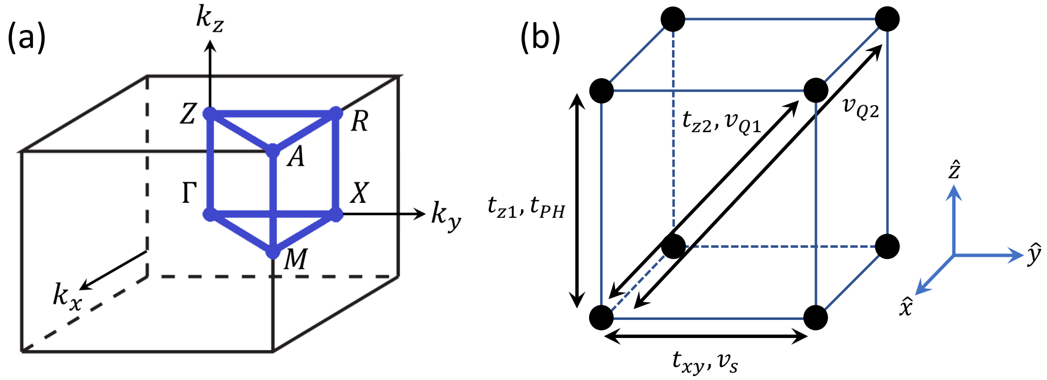

Supplementary Figure 7. BZ and hopping schematic for SEq. (84). (a) The BZ[29–31] of SG 123 $P4/mmm1'$. (b) Schematic of the hoppings of the model of a $p-d$-hybridized HOFA semimetal in SEq. (84). Only one set of hoppings is shown per term in SEq. (84); the full set of hoppings is generated by transforming the arrows in (b) under the symmetries of SG 123 $P4/mmm1'$.

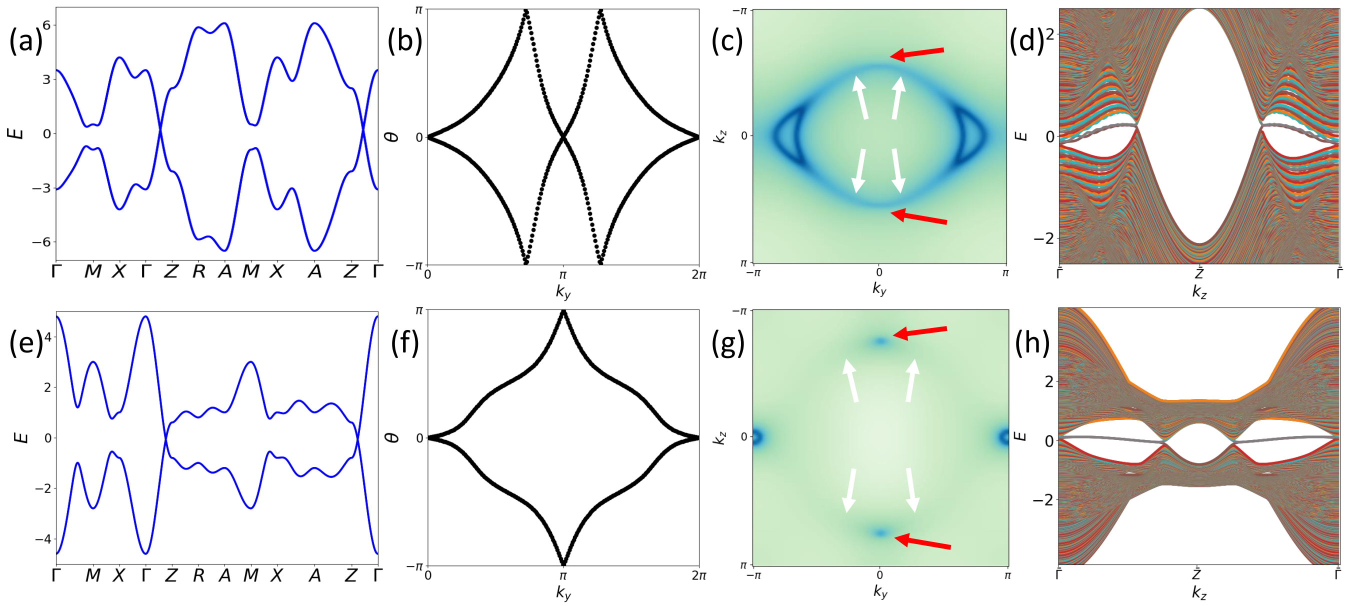

Supplementary Figure 8. Bulk, surface, and hinge states in $s-d$- and $p-d$-hybridized HOFA Dirac semimetals in SG 123 $P4/mmm1'$. (a) Bulk bands of the $s-d_{x^2-y^2}$-hybridized, $\mathcal{I}$-, M_z-, and $\mathcal{T}$-symmetric Dirac semimetal described by $\mathcal{H}_{H2}(\mathbf{k})$ in SEq. (6) and highlighted in the main text, plotted using the parameters in Supplementary Table 2. Here, all of the occupied bands at $k_z = 0$ have the same inversion eigenvalues, and this plane is QSH-trivial. However, because spin-1/2 corepresentations and spin-3/2 corepresentations with the same inversion eigenvalues and opposite pairs of C_{4z} eigenvalues ($\bar{\rho}^+_{6,7}$ in SEqs. (82) and (83)) are inverted at Γ relative to those at M, the Hamiltonian of the $k_z = 0$ plane is equivalent to a mirror TCI with mirror Chern number[32,33] $C_{M_z} = 2$ (Supplementary Table 5), as indicated by (b) the x-directed Wilson spectrum and (c) (100)-surface states. In the surface spectrum in (c), the only surface-localized states are a time-reversed pair of TCI cones at $k_z = 0$; the remains of four Fermi arcs (and their time-reversal partners), which appear in four tightly grouped pairs (white arrows), can be seen connecting the TCI cones at $k_z = 0$ to the projections of the bulk Dirac points (red arrows). As there is no topological invariant that requires these surface Fermi arcs to cross the Fermi energy[34–37], they can be gapped out; here that is accomplished by the bulk quadrupolar SOC term $U(\mathbf{k})\sin(k_z)$ in SEq. (6). (d) z-directed rod bands of the $s-d$-hybridized semimetal; HOFA states are clearly visible connecting the hinge projections of the bulk Dirac points to the projections of the topological surface cones. (e) Bulk bands of the $p_z - d_{x^2-y^2}$-hybridized, $\mathcal{I}$-, M_z-, and $\mathcal{T}$-symmetric Dirac semimetal described by SEq. (84). Here, states with opposite inversion eigenvalues ($\bar{\rho}^-_7$ and $\bar{\rho}^+_6$ in SEqs. (82) and (83)) are inverted at Γ relative to those at M (SEq. (91)), and therefore by the Fu-Kane parity criterion[38], the Hamiltonian of the $k_z = 0$ plane is equivalent to a 2D TI (Supplementary Table 4), as indicated by its (f) x-directed Wilson spectrum and (g) (100)-surface states. In the surface spectrum in (g), the only surface-localized state is a TI cone at $k_z = 0$, $k_y = \pi$; the (extremely) faint remains of two Fermi arcs (and their time-reversal partners) (white arrows) can be seen connecting the TI cones to the projections of the bulk Dirac points (red arrows). These surface Fermi arcs have been almost completely gapped out by bulk quadrupolar SOC ($v_{Q1,2}$ in SEq. (84)). (h) z-directed rod bands of the $p-d$-hybridized semimetal; HOFA states are clearly visible connecting the hinge projections of the bulk Dirac points to the projections of the topological surface cones, as they were in (d).

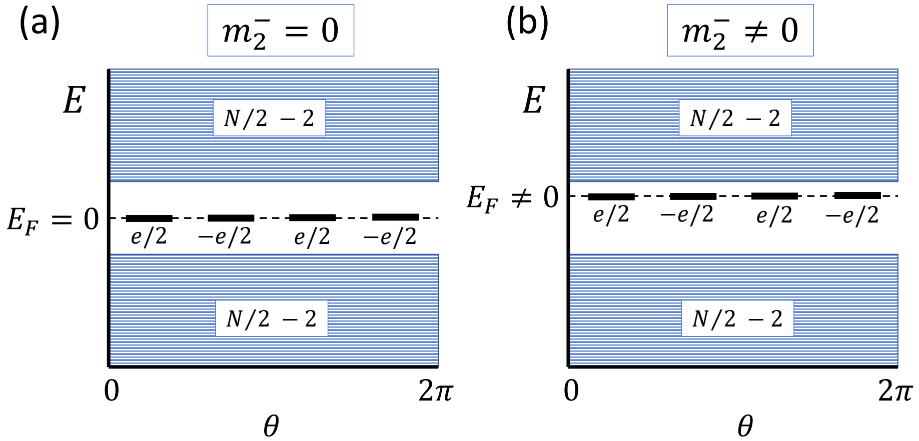

Supplementary Figure 9. Schematic energy spectra of QIs with and without particle-hole symmetry and open boundary conditions (OBC). (a) The corner modes of the QI can be considered two Jackiw-Rebbi solitons and two antisolitions in the particle-hole-symmetric limit that m_2^+ is the only nonzero mass in SEq. (135). In this limit, there are therefore $(N/2)-2$ states in the valence and conduction manifolds, where N is the total number of states. When the four zero modes are half-filled, they exhibit a charge distribution (SEq. (155)) with an $e/2$ quadrupole moment about $r=0$ (SEq. (159)). (b) When one of the m_{2+4a}^- terms in SEq. (135) is perturbatively introduced, particle-hole symmetry $\Pi(\theta)$ (SEq. (123)) is broken and all four 0D modes are uniformly raised in energy (SEq. (164)); this is the same effect as introducing an identity term in the basis of the four 0D modes. However, if this pattern of boundary modes remains half-filled throughout the breaking of particle-hole symmetry, then the four modes still exhibit an $e/2$ quadrupole moment; only the chemical potential has shifted such that $E_F \neq 0$. The QI phase can thus be identified in a $\mathcal{T}$-broken, spinful, $p4m$-symmetric insulator by drawing a line across a gap in the energy spectrum calculated with OBC and counting the number of states below the gap[39], and then by comparing that number to the number of states below the same gap calculated with periodic boundary conditions (PBC). If the difference in the number of states below the gap is $2+4a$, where $a \in \mathbb{Z}$, then the system is a QI (SEq. (159)). In (a) and (b), we depict schematic OBC spectra of a QI; in both cases, the number of states below the four corner modes is $(N/2)-2$, whereas the number of states below the gap in the PBC spectrum is implied to be $N/2$ in both (a) and (b). The difference of 2, combined with the presence of spinful $4mm$ symmetry, indicates that the bulk is a QI.

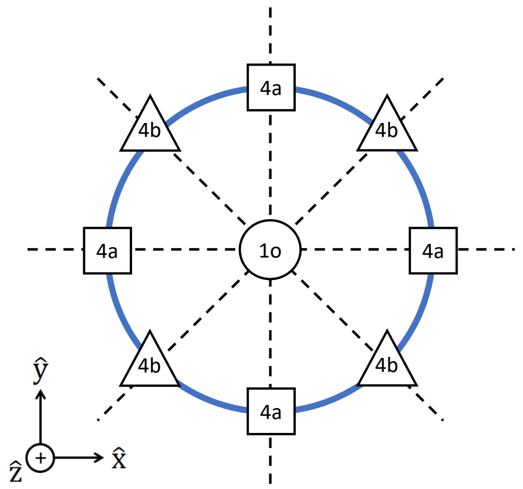

Supplementary Figure 10. The Wyckoff positions of point group[40] $4mm$, generated by $M_{x,y}$ and C_{4z}. Mirrors are indicated by dashed lines. In point groups, the first Wyckoff position is labeled $1o$, to highlight that the origin is a special fixed point in polar (cylindrical) and spherical coordinates. The general position, $8c$, is not pictured. In $4mm$, there is only one maximal Wyckoff position[24]: the $1o$ position (site-symmetry group $4mm$); the $4a$ and $4b$ positions (site-symmetry group m) are non-maximal, because m is a subgroup of $4mm$. This is different than in wallpaper group[20,27] $p4m$, which is generated by adding 2D translations to $4mm$, in which there are two maximal Wyckoff positions with site-symmetry group $4mm$ ($1a$ and $1b$ in Fig. 2(a) of the main text).

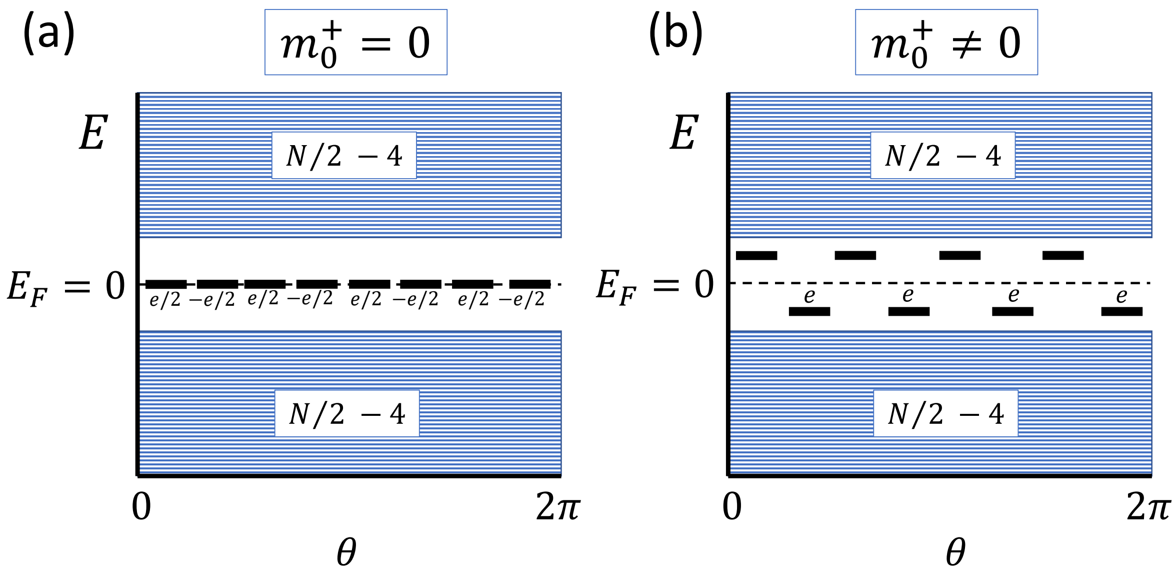

Supplementary Figure 11. Schematic energy spectra of magnetically gapped, $p4m$-symmetric, $s-p_z$-hybridized 2D TIs with and without particle-hole symmetry and open boundary conditions (OBC). (a) For an $s-p_z$-hybridized 2D TI with $p4m$ symmetry, quadrupolar magnetism can generically result in the presence of $8a, a \in \mathbb{Z}$ (SEq. (190)) edge solitons (including zero in the case where m_0^+ is the only nonzero mass term in SEq. (189)). All of these symmetry-allowed soliton configurations exhibit the same, trivial (net-zero) xy and x^2-y^2 quadrupole moments (SEqs. (199) and (200)). (a) As an example, consider the case in which m_4^- is, at first, the only nonzero mass term in SEq. (189). This results in *eight* Jackiw-Rebbi 0D bound states at $\theta_n = n\pi/4$ (SEq. (193)), in the presence of particle-hole symmetry (SEq. (184)). However, when particle-hole symmetry is relaxed as other symmetry-allowed mass terms are reintroduced, half of the modes can float up and half can float down in energy in a $4mm$-symmetric manner (SEq. (201)). In both (a) and (b), the occupied 0D modes do not exhibit a topological quadrupole moment (SEqs. (199) and (200)).

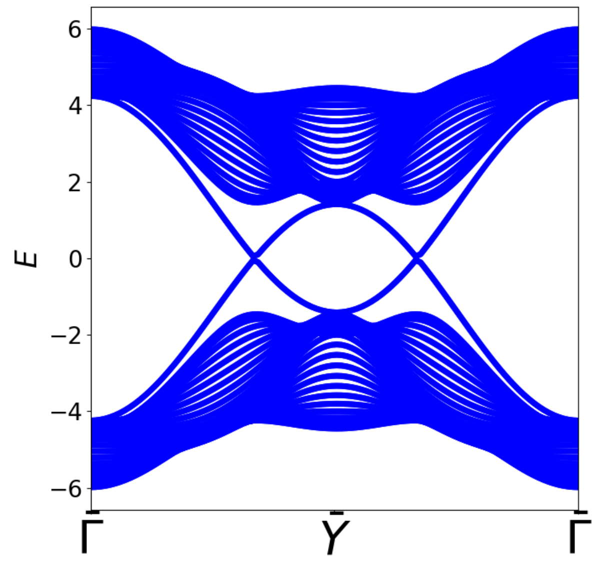

Supplementary Figure 12. The x-directed ribbon bands of the $s - d_{x^2-y^2}$ 2D TCI described by SEq. (1), plotted[41] in the limit that $t_{PH} = 0$, $t_1 = v_m = -t_2 = v_s/2 = 2$. As indicated by the ribbon spectrum, the two occupied bands of this TCI exhibits a mirror Chern number $C_{M_z} = 2$, which can also be determined by direct computation using SEq. (94). Unlike in the (diagonal-mirror-symmetric BZ plane of the) experimentally confirmed TCI phase in SnTe[42], the TCI phase of SEq. (1) is driven by a band inversion between states at $k_x = k_y = 0$ with the same parity eigenvalues and different C_{4z} eigenvalues. Conversely, in SnTe (both in monolayer form[43] and in the diagonal-mirror-symmetric BZ plane of a 3D crystal[42,44]), the $C_{M_z} = 2$ phase is instead driven by band inversion at $(k_x, k_y) = (\pi, 0)$ and $(0, \pi)$ between bands with opposite parity eigenvalues. Therefore, as shown in SN 9, even though the two $C_{M_z} = 2$ TCI phases are topologically equivalent, they will gap into different corner-mode phases when M_z and $\mathcal{I}$ are relaxed, because they exhibit different C_{4z} eigenvalues.

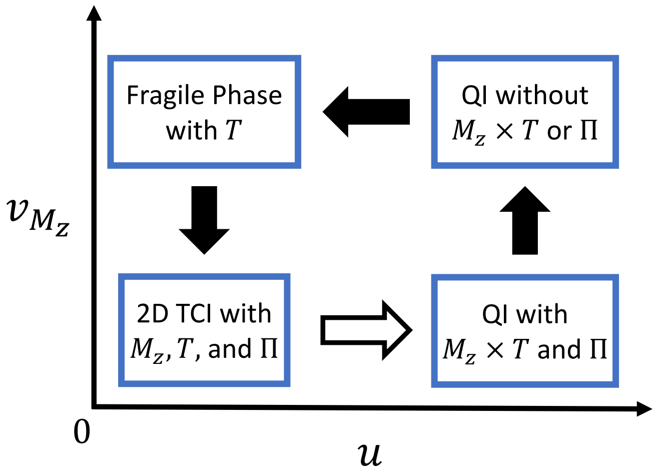

Supplementary Figure 13. Schematic for our analysis of the bulk, edge, and corner spectra of related QI, fragile, and $s - d$-hybridized TCI phases (SN 1). Even though the 2D $C_{M_z} = 2$ TCI in $p4/mmm1'$ examined in SN 9 originates from $s - d$ hybridization (SEqs. (203) through (206)), when the symmetries are lowered to $p4/m'mm$, it transitions into a particle-hole-(Π-) symmetric QI whose linear $k \cdot p$ theory (SEq. (213)) can be mapped to the $k \cdot p$ theory of a (magnetically gapped) $p_z - d_{x^2-y^2}$ TI (SEq. (95)), which we previously analyzed in SN 7. We can therefore schematically work backwards from this Π-symmetric QI phase (black arrows) to avoid performing the more complicated analysis of the edge and corner modes of the quadratic $k \cdot p$ theory of a Π-symmetric $C_{M_z} = 2$ TCI (SEq. (203) and SFig. 12). This allows us to exploit the previous analytic expressions for the boundary (corner) modes and quadrupole moments of a Π-symmetric QI in SN 7 to infer the evolution of the edge and corner spectra of our 2D models in SN 1 through the direct transition from the Π-symmetric TCI to the Π-symmetric QI (white arrow), as well as through the transition from a Π-broken QI to a $\mathcal{T}$-symmetric fragile TI in $p4m1'$ with fractionally charged corner modes (SFig. 14).

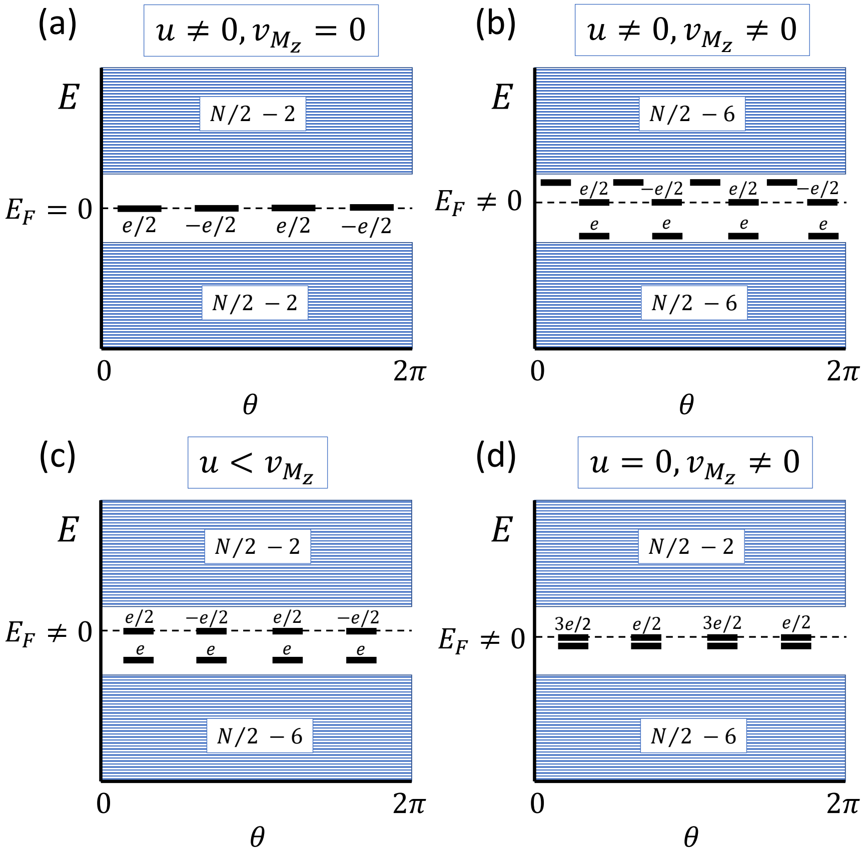

Supplementary Figure 14. Schematic open-boundary-condition (OBC) energy spectra for the transition from a $p4m$- and particle-hole- (Π-) symmetric QI to a $p4m$- and $\mathcal{T}$-symmetric fragile phase with broken Π symmetry. (a) The half-filled corner modes of a Π-symmetric QI, characterized by $u \neq 0$ and $v_{M_z} = 0$ in SEqs. (1), (3), and (4) (SFig. 9(a)). In terms of M_z and $\mathcal{T}$ symmetries, both u and v_{M_z} break M_z, whereas u (v_{M_z}) breaks (respects) $\mathcal{T}$ symmetry; both u and M_z respect the symmetries of $p4m$ (SEq. (2)). (b) Keeping $u \neq 0$, we can introduce eight more corner modes by breaking Π-symmetry through the introduction of nonzero v_{M_z} in SEq. (3). These eight modes are equivalent to the QI-trivial corner states of the 2D $s - p_z$-hybridized TI gapped with $p4m$-symmetric magnetism shown in SFig. 11(b); as shown in the text surrounding SEq. (201), the eight trivial modes generically appear in two energetically-split sets of four $4mm$-symmetry-related states. (c) Further breaking Π symmetry and reducing the strength of u in SEq. (4), we return the four empty, QI-trivial corner modes to the conduction manifold, and push the four occupied trivial corner modes closer to the half-filled (QI-nontrivial) modes in energy. We numerically observe this process in the rod bands near $k_z = 0$ in Fig. 3(h) of the main text, in which a trivial set of HOFAs from the valence manifold begins to approach the HOFAs at the spectral center. (d) Turning u completely to zero while keeping $v_{M_z} \neq 0$, we keep the bulk and edge gap open and restore $\mathcal{T}$ symmetry, resulting in the fragile phase detailed in SN 4. The restoration of $\mathcal{T}$ symmetry forces the corner modes to become doubly degenerate, as they characterize spinful electrons (Fig. 3(h) of the main text). However, as we have not closed a bulk or edge gap, the filling of the corner modes persists from the Π-symmetric QI phase in (a), resulting in quarter-filled (and quarter-empty) corner modes that exhibit the same xy quadrupole moment, taken modulo e, as the corner modes of a magnetic QI in $p4m$ (SN 7). This is consistent with our determination in SEq. (208) that the specific $s - d$-hybridized TCI phase of SEq. (1) exhibits the same nontrivial ($e/2$) quadrupole moment as a $p - d$-hybridized 2D TI when the edge states of the two insulators are respectively gapped. The eight corner modes in (d) modes are not pinned to $E = 0$, as Π is strongly broken. Nevertheless, as there are eight corner modes and the valence and conduction manifolds differ by four states, acting on the modes with a chemical potential that pushes them into one of the bulk manifolds while preserving the bulk and edge gaps will not resolve the (anomalous[17,45]) mismatch between the number of states in the valence and conduction manifolds.

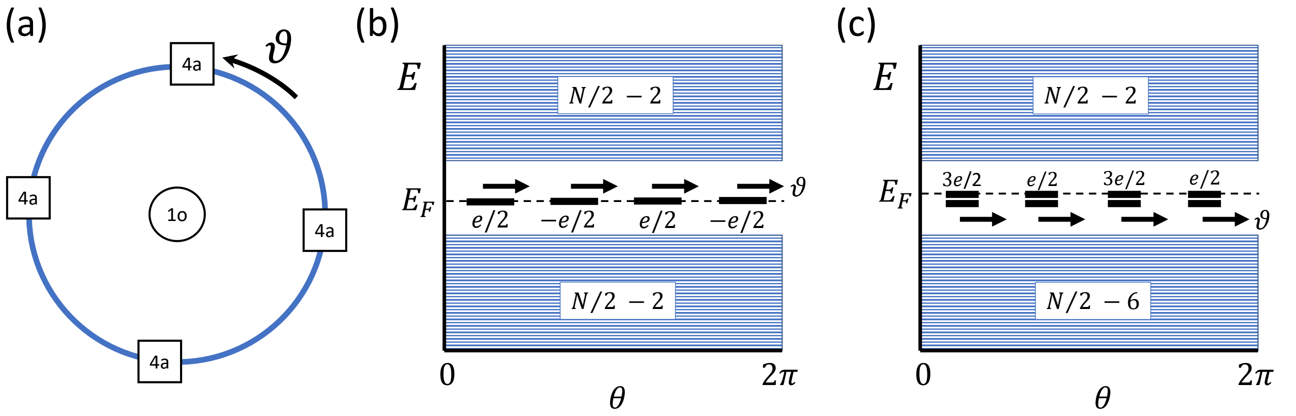

Supplementary Figure 15. (a) The Wyckoff positions of point group[40] 4, generated by C_{4z}. The $4a$ position characterizes four points at C_{4z}-related angles with an overall free angular parameter ϑ. (b) Breaking $M_{x,y}$ while preserving C_{4z} for the QI (SFig. 9), allows the four corner modes to rotate freely as a set; however, as long as the bulk and edge gaps remain open and C_{4z} is preserved, there will remain an anomalous absence of $L_z^{QI} = 2 + 4n$, $n \in \mathbb{Z}$ (SEq. (139)) states from the valence manifold of the open-boundary-condition (OBC) spectrum relative to the spectrum calculated with periodic boundary conditions (PBC). (c) Breaking $M_{x,y}$ while preserving C_{4z} and $\mathcal{T}$ for the fragile phase in $p4m1'$ described in SN 9 similarly allows the four, three-quarters-filled (or quarter-filled) Kramers pairs of corner states to freely rotate as a set; however, if a bulk or edge gap is not closed and C_{4z} and $\mathcal{T}$ are preserved, there will remain an anomalous absence of $6 + 8n$ (or $2 + 8n$) states from the valence manifold of the OBC spectrum relative to the PBC spectrum.

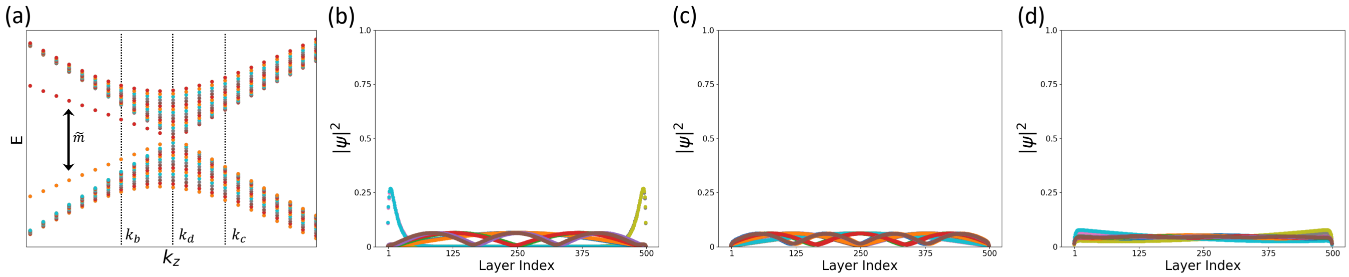

Supplementary Figure 16. Numerical demonstration of the absence of additional surface states in HOFA Dirac semimetals. (a) Energy spectrum of the $\mathcal{T}$-broken Dirac semimetal in SEq. (5), plotted as a function of k_z in the vicinity of k_d for a Dirac point located at $k_x = k_y = 0$, $k_z = k_d$, and restricting to the 64 states closest to the center of the spectrum $E = E_d$. Above and below E_d, the spectrum at each k point exhibits $N/2$ states, where N is the total number of states in a slab with $N/4$ layers, where the factor of 4 originates from the two spin-1/2 s and two spin-1/2 d orbitals in each unit cell. We take $k_y = 0$ for all calculations. Two pairs of gapped surface Fermi arc states, whose splitting $\tilde{m}$ in energy (a) scales as $u\sin(k_d - k_z)$ in SEq. (5), can be observed at $k_z < k_d$. At fixed values of k_z less than k_d (but still closer to k_d than to $k_z = 0$), the surface Fermi arc states also exhibit an increasing energy gap with increasing $|k_y|$ (not pictured). (b) Orbital-summed wavefunction magnitude $\sum_{s,d,\sigma} |\psi|^2$ of states in (a) at k_b, plotted as a function of layer index along x, the finite direction of the slab; exactly four states can be observed localized on the two $\pm x$-normal boundaries, corresponding to the gapped Fermi arc states in (a). (c) Orbital-summed wavefunction magnitude of the states at k_c in (a); all of these states are localized in the bulk; *i.e.* they are centered about layer index 250 and decay as layer index approaches the boundary values of 1 and 500. (d) Orbital-summed wavefunction magnitude of the states exactly at the bulk Dirac point at k_d. All of the system states are perfectly delocalized; we do not detect additional surface states.

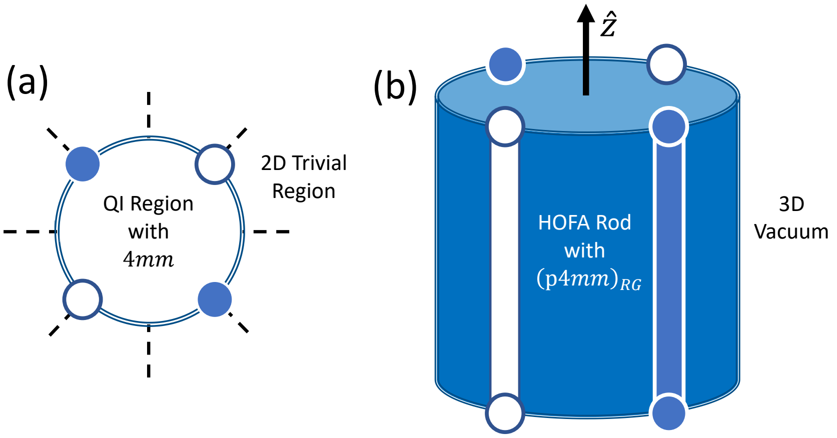

Supplementary Figure 17. The boundary states of finite-sized QI discs and HOFA Dirac semimetal rods. (a) In 2D, a finite-sized region in position space with the bulk topology of a QI will exhibit corner states bound to reflection-fixed angles (*i.e.* $\theta_n^{4a,4b}$ in SEqs. (227) and (228) and SFig. 10) if the point group of the QI region has $4mm$ (or higher) symmetry (SN 6). (b) Extrapolating to 3D, a Dirac semimetal with bulk $4mm$ (or higher) symmetry will exhibit HOFA states that are fixed to $\theta = \theta_n^{4a,4b}$ if, in position space, it is cut into a rod that has a fourfold axis (either a rotation or a screw axis) and two perpendicular reflections (either mirrors or glides whose fractional translations are parallel to the fourfold axis). This implies that the rod is symmetric under the action of a rod group[23] that is a supergroup of either $(p4mm)_{RG}$, $(p4_2cm)_{RG}$, $(p4_2mc)_{RG}$, or $(p4cc)_{RG}$. The $\mathcal{T}$-symmetric rod supergroups of these (magnetic[20]) rod groups are provided in Supplementary Table 6. Note that unlike a nanorod of a higher-order topological insulator[11,27,28,39,46–50], the HOFA semimetal nanorod in (b) is gapless in its interior (and possibly also on its faces (SN 5)), because of its bulk (and possible surface) nodal points. Nevertheless, in the limit that translation in the z direction is still approximately preserved, the HOFA states depicted in (b) may still be detected through momentum-resolved probes along the hinges of the rod.

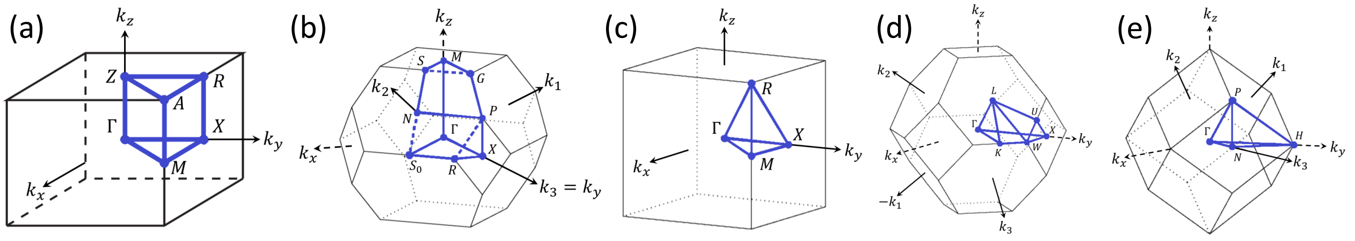

Supplementary Figure 18. The bulk Brillouin zones (BZs), highlighting the k-paths from SRef. 29 and labeling according to the BCS convention[30,31], of the space groups that support Dirac semimetals with HOFA states (Supplementary Table 7). The lattices of these space groups are (a) primitive tetragonal, (b) body-centered tetragonal, (c) primitive cubic, (d) face-centered cubic, and (e) body-centered cubic.

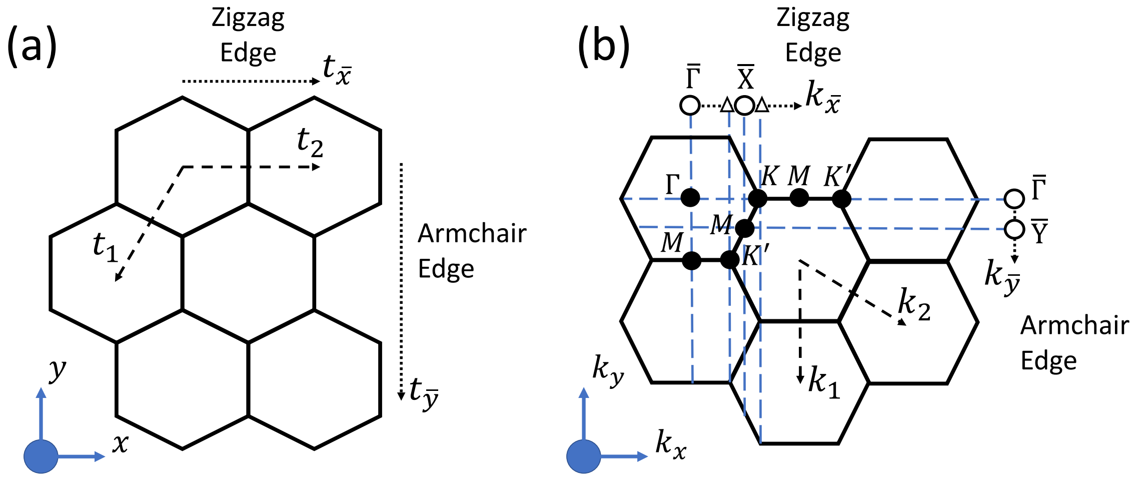

Supplementary Figure 19. Termination dependence of the edge BZ and Fermi arcs in graphene. (a) The position-space 2D honeycomb lattice of graphene, which can be terminated with either zigzag or armchair edges[51]. For the zigzag termination of graphene, the edge lattice vector $t_{\bar{x}}$ is the same as the bulk lattice vector t_2. However, for armchair-terminated graphene, the edge lattice vector $t_{\bar{y}}$ is equal to a linear combination of bulk lattice vectors $2t_1 + t_2$. (b) The 2D bulk and edge Brillouin Zones (BZs) of graphene. The termination dependence of the edge lattice vector in graphene is reflected in where the bulk high-symmetry BZ points project in the edge BZ. For a zigzag-terminated edge, the Dirac points at K and K' project to different points (triangles) on the 1D edge BZ. These projections are spanned by boundary polarization modes, *i.e.*, arc-like flat-band edge states[52–55], that are the "first-order" analogs of the HOFA states analyzed in this work. However, on armchair-terminated edges, the K and K' points both project to the same surface TRIM point $\bar{\Gamma}$, and therefore, as there is no region between the projections of the bulk Dirac points in the 1D armchair edge BZ, there are no edge Fermi arcs[55].

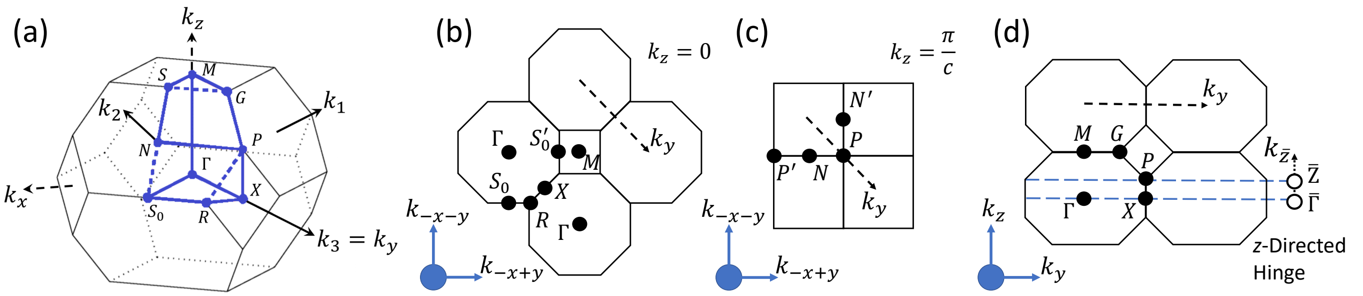

Supplementary Figure 20. Hinge projection of the bulk BZ in body-centered tetragonal crystals. (a) The full 3D bulk BZ[29–31], (b) the bulk BZ slice at $k_z = 0$, and (c) the bulk BZ slice at $k_z = \pi/c$ of a body-centered tetragonal crystal, where c is the lattice periodicity in the z direction (though, as shown in SEq. (233), $k_z = 2\pi/c$ is not a reciprocal lattice vector). (d) In the 1D hinge BZ of a z-directed rod, all of the points in the planes at $k_z = 0, 2\pi/c$ project to the hinge TRIM point $\bar{\Gamma}$, and all of the points at $k_z = \pm\pi/c$ project to the hinge TRIM point $\bar{Z}$. As long as Dirac points along a fourfold axis in the BZ do not lie exactly in the $k_z = \pm\pi/c$ planes, there will be a finite distance between their projections in the 1D hinge BZ, allowing for the presence of HOFA states under the symmetry conditions used to obtain Supplementary Table 8..

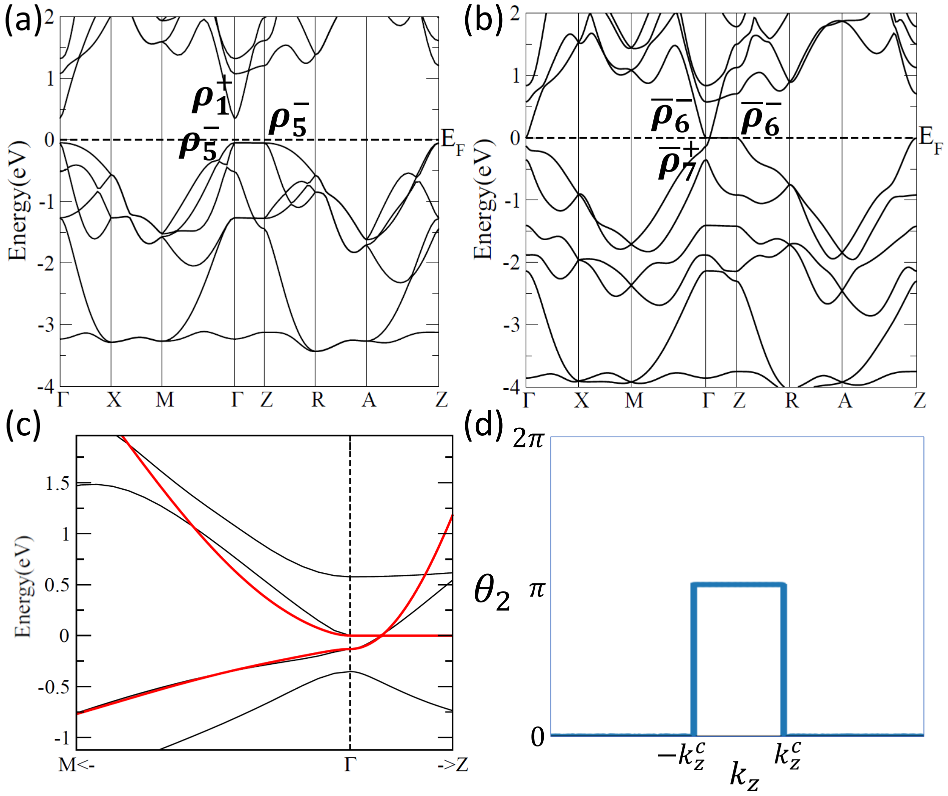

Supplementary Figure 21. The electronic structure of KMgBi in SG 129 $P4/nmm1'$ as calculated from first principles. Bands (a) without and (b) with spin-orbit coupling (SOC), labeled by the single- and double-valued corepresentations, respectively, of point group $4/mmm1'$ (D_{4h}), to which the little co-groups at Γ and Z are isomorphic. In (a), the highest valence band comes from an antibonding combination of Bi p orbitals, and the lowest conduction band comes from a K s orbital. In (b), the effects of SOC drive a band inversion along ΓZ, resulting in a time-reversed pair of symmetry-stabilized Dirac points at $k_x = k_y = 0$, $k_z = \pm k_z^c$, where $k_z^c = 0.06085$ $(2\pi/c)$. (c) Bands fitted from the $k \cdot p$ Hamiltonian in SEq. (237) (red) to the bands obtained from first-principles calculations (black). (d) The nested Wilson loop of the $k \cdot p$ Hamiltonian in SEq. (237) with the parameters in Supplementary Table 9. To obtain the nested Wilson phase $\theta_2(k_z)$ in (d), we first calculate the x-directed Wilson loop $W_1(k_y, k_z)$ over the lower two bulk bands, and then calculate the y-directed nested Wilson loop[17,26,28] over the lower Wilson band of $W_1(k_y, k_z)$. The resulting nested Wilson loop $W_2(k_z)$ has only a single eigenvalue $\theta_2(k_z)$ at each value of k_z, and indicates a nontrivial nested Berry phase of π for the values of k_z with HOFA states in Fig. 4(d) of the main text ($0 < |k_z| < k_z^c$).

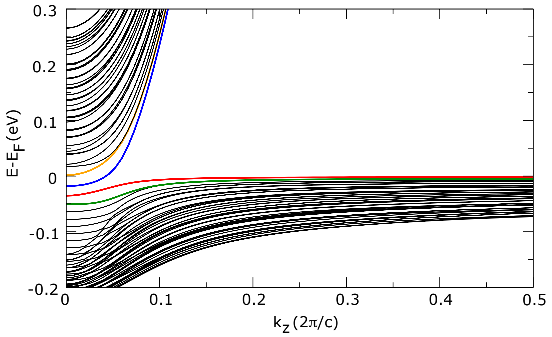

Supplementary Figure 22. The band structure of a square, z-directed rod of a tight-binding model obtained from the $k \cdot p$ theory in SEq. (237) and fit to the first-principles electronic structure of KMgBi in the vicinity of the Γ point (SFig. 21(c)) using the parameters in Supplementary Table 9. Because the rod preserves spinful $\mathcal{I} \times \mathcal{T}$ symmetry (or alternatively, because the rod preserves the spinful x and y reflection symmetries of SEq. (237), whose representations anticommute), the bands are doubly degenerate[19,56]. Fixing the rod filling to that of the bulk Dirac points, we label the two highest valence (lowest conduction) bands in red (blue). Though the hinge projection of the bulk Dirac point at $k_z^c = 0.06085$ $(2\pi/c)$ is split by finite-size effects, we still observe four, half-filled HOFA states at $0 < |k_z| < k_z^c$ (which are split into two occupied (red) and two unoccupied (blue) HOFA states by finite-size effects).

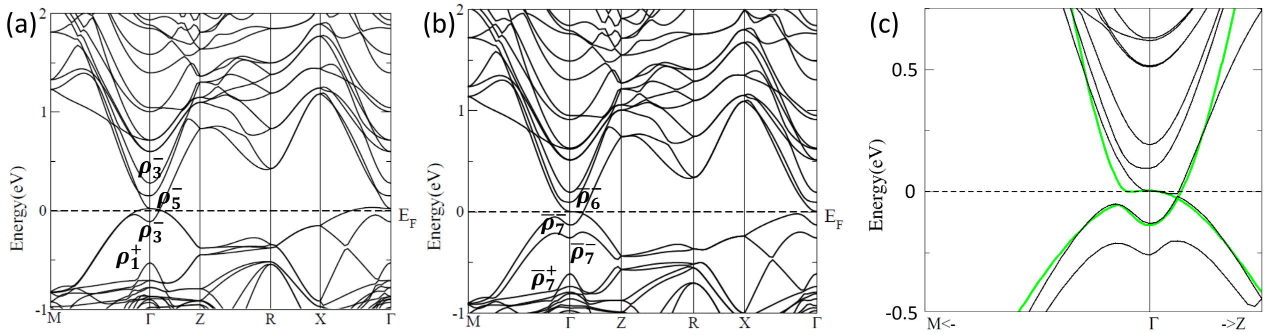

Supplementary Figure 23. The electronic structure of α''-Cd_3As_2 in SG 137 $P4_2/nmc1'$ calculated from first principles. Bands (a) without and (b) with spin-orbit coupling, labeled by the single- and double-valued corepresentations, respectively, of point group $4/mmm1'$ (D_{4h}), to which the little co-group at Γ is isomorphic. (c) Bands fitted from the $k \cdot p$ Hamiltonian in SEq. (237) (green) to the bands obtained from first-principles calculations (black).

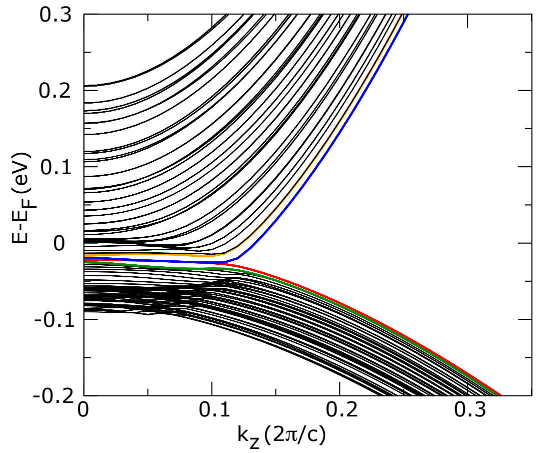

Supplementary Figure 24. The band structure of a square, z-directed rod of a tight-binding model obtained from the $k \cdot p$ theory in SEq. (237) and fit to the first-principles electronic structure of α''-Cd_3As_2 in the vicinity of the Γ point (SFig. 23(c)) using the parameters in Supplementary Table 10. Because the rod preserves spinful $\mathcal{I} \times \mathcal{T}$ symmetry (or alternatively, because the rod preserves the spinful x and y reflection symmetries of SEq. (237), whose representations anticommute), the bands are doubly degenerate[19,56]. Fixing the rod filling to that of the bulk Dirac points at $k_z = \pm k_z^c$, where $k_z^c = 0.125$ $(2\pi/c)$, we label the two highest valence (lowest conduction) bands in red (blue). We observe four, half-filled HOFA states connecting the hinge projection of the surface TI cone at $k_z = 0$ to hinge projection of a bulk Dirac point.

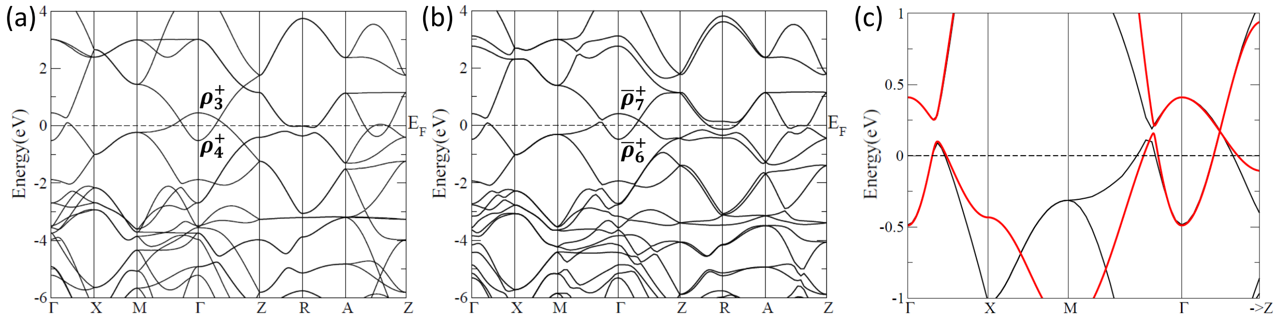

Supplementary Figure 25. The electronic structure of β'-PtO_2 in SG 136 $P4_2/mnm1'$. Bands (a) without and (b) with spin-orbit coupling, labeled by the single- and double-valued corepresentations, respectively, of point group $4/mmm1'$ (D_{4h}), to which the little co-group at Γ is isomorphic. (c) Bands fitted from the $k \cdot p$ Hamiltonian in SEq. (243) (red) to the bands obtained from first-principles calculations (black). The Dirac points in β'-PtO_2 lie along ΓZ in (b,c).

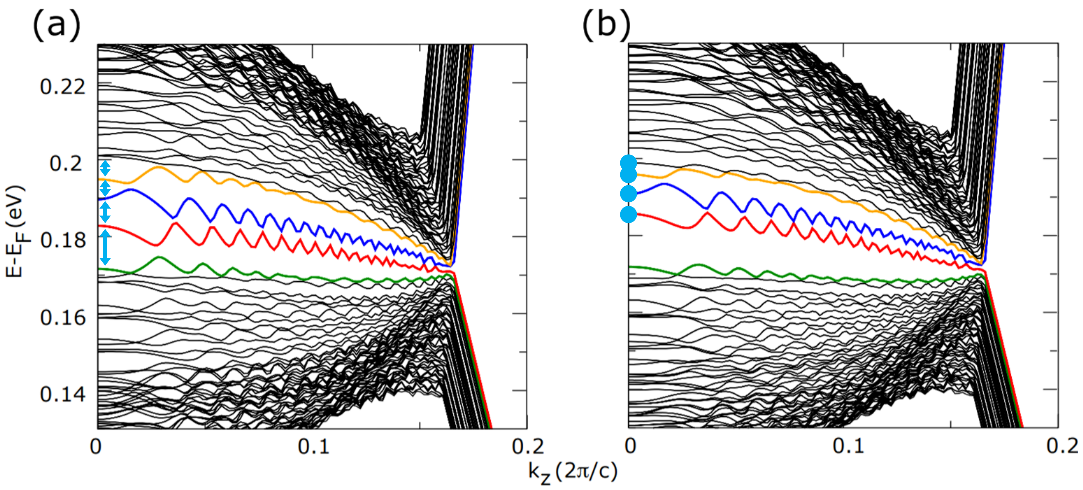

Supplementary Figure 26. The band structures of square, z-directed rods of tight-binding models obtained from the $k \cdot p$ theory in SEq. (243) and fit to the first-principles electronic structure of β'-PtO_2 in the vicinity of the Γ point (SFig. 25(c)) using the parameters in Supplementary Table 11. The bands in (a,b) were respectively calculated in the absence and presence of an external electric field modeled by setting $D = 0.04$ (eV Å^{-1}) in SEq. (245). Because the rod in (a) preserves spinful $\mathcal{I} \times \mathcal{T}$ symmetry (or alternatively, because the rod in (a) preserves the spinful x and y reflection symmetries of SEq. (243), whose representations anticommute), the bands in (a) are doubly degenerate[19,56]; in (b), even though $\mathcal{I} \times \mathcal{T}$ is broken by the external electric field, the anticommuting x and y reflections are still preserved, and the rod bands remain doubly degenerate. In (a,b), fixing the rod filling to that of the bulk Dirac points, we label the two highest valence (lowest conduction) bands in red (blue), and label the next two highest valence (lowest conduction) bands in green (orange). In (a), the TCI surface states at $k_z = 0$ are split by finite-size effects (blue arrows in (a) at $k_z = 0$), but we still observe eight hinge-localized surface states spanning from $k_z = 0$ to the hinge projection of the bulk Dirac point at $k_z^c = 0.1663$ $(2\pi/c)$; these eight bands correspond to the bands labeled in red, blue, and orange, as well as the black pair of conduction bands immediately above the orange pair of bands. Of these eight states, only two (the red bands) are filled. When the effects of an external field are incorporated in (b), the TCI surface states split in the hinge spectrum calculated through hinge Green's functions (Fig. 5(c) of the main text), but the rod spectrum remains nearly identical to (a) ((a) and (b) are only distinguished by the growing gap at $k_z = 0$ between the red and green pairs of bands, and by the shrinking gap at $k_z = 0$ between the orange pair of bands and the lowest black pair of conduction bands). In (b), we interpret the eight HOFA states identified in (a) as connecting to four, quarter-filled Kramers pairs of states at $k_z = 0$ (blue circles in (b) at $k_z = 0$) whose eightfold degneracy is split in the rod spectrum by finite-size effects, and which correspond to the Kramers pairs of hinge states at $k_z = 0$ in the hinge Green's function shown in Fig. 5(c) of the main text.

SUPPLEMENTARY TABLES

Parameters for the 2D Models in Fig. 2 of the Main Text									
Phase	Panels	Equations	t_1	t_2	v_m	t_{PH}	v_s	v_{M_z}	u
Trivial Insulator	(c-e)	(1)	2	1.5	-5	0.1	1.3	0	0
M_z- and $\mathcal{T}$-Symmetric $C_{M_z} = 2$ TCI	(f-h)	(1)	2	1.5	-1.5	0.1	1.3	0	0
$\mathcal{T}$-Symmetric, M_z-Broken Fragile TI	(i-k)	(1) and (3)	2	1.5	-1.5	0.1	1.3	0.4	0
$M_z \times \mathcal{T}$-Symmetric Quadrupole	(l-n)	(1) and (4)	2	1.5	-1.5	0.1	1.3	0	0.5

Supplementary Table 1. Parameters used in SEqs. (1), (3), and (4) to realize the 2D insulating phases shown in Supplementary Figure (SFig.) 1 and in Fig. 2 of the main text.

Parameters for the 3D Models in Fig. 3 of the Main Text											
Phase	Panels	Equations	t_1	t_2	v_m	t_{PH}	v_s	t_H	u ($\mathcal{H}_{H1}$)	u ($\mathcal{H}_{H2}$)	v_{M_z}
$M_z \times \mathcal{T}$-Symmetric Magnetic HOFA Dirac	(c,f)	(5)	1	2	-1.5	0.1	1.3	2.8	0.5	0	0
M_z- and $\mathcal{T}$-Symmetric HOFA Dirac	(d,g)	(6)	1	2	-1.5	0.1	1.3	2.8	0	0.5	0
$\mathcal{T}$-Symmetric HOFA Dirac without 2D Surface States	(e,h)	(6) and (3)	1.5	0.4	-1.5	0.1	1.3	2.3	0	0.5	0.3

Supplementary Table 2. Parameters used in SEqs. (3), (4), (5), and (6) to realize the 3D semimetallic phases shown in SFig. 1 and in Fig. 3 of the main text.

Spinful Atomic Orbitals	Site-Symmetry Representation of $4mm$	Wyckoff Position of $p4m$	Representation Subduced at Γ	Representation Subduced at M
s	$\bar{E}_1$	$1a$	$\bar{\rho}_7$	$\bar{\rho}_7$
$d_{x^2-y^2}$	$\bar{E}_2$	$1a$	$\bar{\rho}_6$	$\bar{\rho}_6$
s	$\bar{E}_1$	$1b$	$\bar{\rho}_7$	$\bar{\rho}_6$
$d_{x^2-y^2}$	$\bar{E}_2$	$1b$	$\bar{\rho}_6$	$\bar{\rho}_7$

Supplementary Table 3. The elementary band representations (EBRs) induced into type-I magnetic wallpaper group $p4m$ from the double-valued site symmetry representations that transform as spinful s and $d_{x^2-y^2}$ orbitals at the $1a$ and $1b$ Wyckoff positions (Fig. 2(a) of the main text), and the resulting subduced little group representations at Γ and M (Fig. 2(b) of the main text), employing the notation in SEqs. (31) and (32). Band inversion at Γ or M between irreducible representations $\bar{\rho}_6$ and $\bar{\rho}_7$ for bands induced from orbitals at the $1a$ position results in a set of occupied bands with the same symmetry eigenvalues as an EBR induced from the $1b$ position. All representations were obtained using the point group[40] and BANDREP[24,57–59] tools on the Bilbao Crystallographic Server (BCS).

Number of Each Occupied Corepresentation in SEq. (94) for the $p-d$-Hybridized HOFA Dirac Semimetal in SEq. (84)							
$k_z=0$	$n_{0,0,0}(\bar{\rho}_7^-)$	$n_{0,0,0}(\bar{\rho}_6^+)$	$n_{0,0,0}(\bar{\rho}_6^-)$	$n_{\pi,\pi,0}(\bar{\rho}_7^-)$	$n_{\pi,\pi,0}(\bar{\rho}_6^+)$	$n_{\pi,\pi,0}(\bar{\rho}_6^-)$	$n_{\pi,0,0}(\bar{\varrho}_5^-)$
Number	1	0	0	0	1	0	1
$C_{M_z}(0)$ mod $4=1$, $z_2(0)=1$							
$k_z=\pi$	$n_{0,0,\pi}(\bar{\rho}_7^-)$	$n_{0,0,\pi}(\bar{\rho}_6^+)$	$n_{0,0,\pi}(\bar{\rho}_6^-)$	$n_{\pi,\pi,\pi}(\bar{\rho}_7^-)$	$n_{\pi,\pi,\pi}(\bar{\rho}_6^+)$	$n_{\pi,\pi,\pi}(\bar{\rho}_6^-)$	$n_{\pi,0,\pi}(\bar{\varrho}_5^-)$
Number	0	1	0	0	1	0	0
$C_{M_z}(\pi)$ mod $4=0$, $z_2(\pi)=0$							

Supplementary Table 4. The number of occupied corepresentations, mirror Chern numbers $C_{M_z}(k_z)$ (modulo 4)[33,60] (SEq. (94)), and 2D TI indices[38] $z_2(k_z)$ (C_{M_z} mod 2) for the $p-d$-hybridized HOFA Dirac semimetal in SEq. (84) with the parameters in SEq. (88). The Hamiltonian of the $k_z=0$ plane is equivalent to a 2D TI, whereas the Hamiltonian of the $k_z=\pi$ plane exhibits the same symmetry eigenvalues as a trivial insulator[33,49,60–62].

Number of Each Occupied Corepresentation in SEq. (94) for the $s-d$-Hybridized HOFA Dirac Semimetal in the Main Text (SEq. (6))							
$k_z=0$	$n_{0,0,0}(\bar{\rho}_7^-)$	$n_{0,0,0}(\bar{\rho}_6^+)$	$n_{0,0,0}(\bar{\rho}_6^-)$	$n_{\pi,\pi,0}(\bar{\rho}_7^-)$	$n_{\pi,\pi,0}(\bar{\rho}_6^+)$	$n_{\pi,\pi,0}(\bar{\rho}_6^-)$	$n_{\pi,0,0}(\bar{\varrho}_5^-)$
Number	0	1	0	0	0	0	0
$C_{M_z}(0)$ mod $4=2$, $z_2(0)=0$							
$k_z=\pi$	$n_{0,0,\pi}(\bar{\rho}_7^-)$	$n_{0,0,\pi}(\bar{\rho}_6^+)$	$n_{0,0,\pi}(\bar{\rho}_6^-)$	$n_{\pi,\pi,\pi}(\bar{\rho}_7^-)$	$n_{\pi,\pi,\pi}(\bar{\rho}_6^+)$	$n_{\pi,\pi,\pi}(\bar{\rho}_6^-)$	$n_{\pi,0,\pi}(\bar{\varrho}_5^-)$
Number	0	0	0	0	0	0	0
$C_{M_z}(\pi)$ mod $4=0$, $z_2(\pi)=0$							

Supplementary Table 5. The number of occupied corepresentations, mirror Chern numbers $C_{M_z}(k_z)$ (modulo 4)[33,60] (SEq. (94)), and 2D TI indices[38] $z_2(k_z)$ (C_{M_z} mod 2) for the $s-d$-hybridized HOFA Dirac semimetal highlighted in the main text (SEq. (6) with the parameters in Supplementary Table 2). The Hamiltonian of the $k_z=0$ plane is equivalent to a 2D TCI with C_{M_z} mod $4=2$, whereas the Hamiltonian of the $k_z=\pi$ plane exhibits the same symmetry eigenvalues as a trivial insulator[33,49,60–62].

$\mathcal{T}$-Symmetric Rod Groups with Point Group $4mm1'$ or $4/mmm1'$						
RG Symbol	RG Number	Isomorphic SG Number		RG Symbol	RG Number	Isomorphic SG Number
$(p4mm1')_{\text{RG}}$	34	99		$(p4/mmm1')_{\text{RG}}$	39	123
$(p4_2cm1')_{\text{RG}}$	35	101		$(p4_2/mmc1')_{\text{RG}}$	38	131
$(p4_2mc1')_{\text{RG}}$		105		$(p4_2/mcm1')_{\text{RG}}$		132
$(p4cc1')_{\text{RG}}$	36	103		$(p4/mcc1')_{\text{RG}}$	40	124

Supplementary Table 6. Crystallographic rod groups[23] with $\mathcal{T}$ symmetry and whose point groups contain $4mm$. We also list the numbers of their isomorphic space groups under the addition of in-plane lattice translations T_x and T_y (SEq. (226)). Because the rod groups are subperiodic groups that are finite in all directions in the xy-plane and infinite in the z (c) direction[23], they do not distinguish between symmetries such as the glide reflections $g_x=\{M_x|00\frac{1}{2}\}$ and $g_{x+y}=\{M_{x+y}|00\frac{1}{2}\}$. For example, rod group 35 can either characterize a rod with $g_x=\{M_x|00\frac{1}{2}\}$ symmetry ($(p4_2cm1')_{RG}$) or a rod with $g_x=\{M_x|00\frac{1}{2}\}$ symmetry ($(p4_2mc1')_{RG}$), which are listed under different "settings" of rod group 35 on the BCS[30,31]. However, when in-plane lattice translations are added to a rod group to convert it into a space group, g_x and g_{x+y} are no longer related by a translation-preserving unitary transformation. Therefore, one rod group can become two different space groups depending on the orientation of its in-plane crystal symmetries[23,30,31] relative to the added in-plane lattice translations. In all of the nonsymmorphic rod groups in this table (*i.e.* those whose symbols contain the letter c), the glide reflections and screw symmetries only contain fractional translations in the z direction (along the rod axis), because translations in the xy-plane are not symmetries of z-directed rods.

Space Groups Admitting Dirac Points with Reflection-Fixed HOFA States							
SG Symbol	SG Number		SG Symbol	SG Number		SG Symbol	SG Number
$P4mm1'$	$99^{\dagger}$		$P4/mcc1'$	$124^{\dagger}$		$I4/mmm1'$	139
$P4_2cm1'$	$101^{\dagger}$		$P4/nmm1'$	129		$I4/mcm1'$	140
$P4cc1'$	$103^{\dagger}$		$P4/ncc1'$	130		$Pm\bar{3}m1'$	$221^{\ddagger}$
$P4_2mc1'$	$105^{\dagger}$		$P4_2/mmc1'$	$131^{\dagger}$		$Pm\bar{3}n1'$	$223^{\ddagger}$
$I4mm1'$	107		$P4_2/mcm1'$	$132^{\dagger}$		$Fm\bar{3}m1'$	225
$I4cm1'$	108		$P4_2/nmc1'$	137		$Fm\bar{3}c1'$	226
$P4/mmm1'$	$123^{\dagger}$		$P4_2/ncm1'$	138		$Im\bar{3}m1'$	229

Supplementary Table 7. Space groups that admit Dirac points with HOFA states derived from the QI introduced in Supplementary Reference (SRef.) 26 and pinned to reflection-fixed rod hinges (*i.e* to $\theta_n^{4a,4b}$ in SEqs. (227) and (228) and in SFig. 10). These groups comprise the supergroups of the isomorphic space groups listed in Supplementary Table 6, obtained using MINSUP on the BCS[30,31,63]. For all of these SGs, HOFA Dirac points are always allowed to form along ΓZ in tetragonal SGs (ΓM if body-centered) and ΓX in cubic SGs (ΓH if face-centered) (SFig. 18). In a subset of the primitive tetragonal groups, denoted with †, HOFA Dirac points may also form along MA. In a subset of the primitive cubic groups, denoted with ‡, HOFA Dirac points may also form along MR. Quadrupolar HOFA states are also supported in Dirac semimetals in additional SGs, if we relax that constraint that the HOFA states at each QI-nontrivial rod k point are bound to $\theta_n^{4a,4b}$ (SEqs. (227) and (228)); a complete tabulation of all SGs supporting Dirac semimetals with free-angle HOFA states is provided in Supplementary Table 8 and is reproduced in Table 2 of the main text.

Space Groups Admitting Dirac Points with HOFA States		
Point Group Name	Point Group Symbol	SG Numbers
C_{4v}	$4mm1'$	99 – 110
D_{4h}	$4/mmm1'$	123 – 142
O_h	$m\bar{3}m1'$	221 – 230

Supplementary Table 8. Space groups that admit Dirac points with HOFA states derived from the QI introduced in SRef. 26. This list includes all of the SGs listed in Supplementary Table 7, which contains the more restrictive set of SGs for which Dirac semimetals can be cut into rods that exhibit HOFA states pinned to the high-symmetry rod hinges at $\theta = \theta_n^{4a,4b}$ in SEqs. (227) and (228) and SFig. 10. In addition to those SGs, this table also includes SGs whose reflections contain in-plane lattice translations, such that they are either glide reflections with in-plane fractional lattice translations (*e.g.*, $g_x = \{M_x|\frac{1}{2}\frac{1}{2}0\}$ in SG 100 $P4bm1'$) or mirror reflections that do not coincide with the bulk fourfold axes (*e.g.*, $M_{x+y} = \{M_{x+y}|\frac{1}{2}\frac{1}{2}0\}$ and $C_{4z} = \{C_{4z}|000\}$ in SG 100 $P4bm1'$), neither of which can be preserved in a rod geometry that also preserves a fourfold axis. For all of the SGs in this table, semimetals with Dirac points along lines with $4mm$ or $4/m'mm$ symmetry will exhibit intrinsic HOFA states when cut into nanorods that preserve fourfold axes and are thick compared to the in-plane lattice spacing. This list is reproduced in Table 2 of the main text.

Supplementary Table 9. The parameters used to fit the bands of the $k \cdot p$ theory in SEq. (237) to the first-principles electronic structure of KMgBi in the vicinity of the Γ point (SFig. 21(c)).

C_0 (eV)	C_1 (eV Å^{-2})	C_2 (eV Å^{-2})	A (eV Å^{-1})	M_0 (eV)	M_1 (eV Å^{-2})	M_2 (eV Å^{-2})	B_1 (eV Å^{-3})	B_2 (eV Å^{-3})
-0.06595	31.58273	7.89568	2.51327	-0.06595	-31.58273	-13.42266	-124.0251	0

Supplementary Table 10. The parameters used to fit the bands of the $k \cdot p$ theory in SEq. (237) to the first-principles electronic structure of α''-Cd_3As_2 in the vicinity of the Γ point (SFig. 23(c)).

C_0 (eV)	C_1 (eV Å^{-2})	C_2 (eV Å^{-2})	A (eV Å^{-1})	M_0 (eV)	M_1 (eV Å^{-2})	M_2 (eV Å^{-2})	B_1 (eV Å^{-3})	B_2 (eV Å^{-3})
-0.066	9.8696	12.23831	0.62832	-0.07	-18.16007	-18.94964	124.025106	0

Supplementary Table 11. The parameters used to fit the bands of the $k \cdot p$ theory in SEq. (243) to the first-principles electronic structure of β'-PtO_2 in the vicinity of the Γ point (SFig. 25(c)).

ϵ_0 (eV)	ϵ_1 (eV Å^{-2})	ϵ_2 (eV Å^{-2})	M_0 (eV)	M_1 (eV Å^{-2})	M_2 (eV Å^{-2})	A (eV Å^{-1})	B (eV Å^{-1})	C (eV Å^{-1})
-0.04	2.3	4.023	0.45	-4.90	-8.046	1.6	1.3	0.4

SUPPLEMENTARY NOTES

1. Tight-Binding Parameters for Figures 2 and 3 of the Main Text

Here, we list the specific model parameters used to generate the figures in the main text. All plots shown in this paper were generated using the tight-binding, slab (ribbon), and Wilson loop functionality of the PythTB package[41]. The nested Wilson loop shown in Fig. 3(b) of the main text was calculated by modifying the PythTB functions relating to Wannier centers and Berry phase. For convenience, we reproduce in this supplement the Hamiltonians and potentials introduced in]red the main text of this work. First, in the main text, we formulated a 2D time-reversal- ($\mathcal{T}$-) symmetric Hamiltonian (Eq. (1) of the main text):

$$\begin{aligned}\mathcal{H}(\mathbf{k}) = t_1\tau^z[\cos(k_x)+\cos(k_y)] + t_2\tau^x[\cos(k_x)-\cos(k_y)] + v_m\tau^z \\ + t_{PH}\mathbb{1}_{\tau\sigma}[\cos(k_x)+\cos(k_y)] + v_s\tau^y\sigma^z\sin(k_x)\sin(k_y),\end{aligned} \tag{1}$$

that was invariant under layer group[20,23,27,56,64] $p4/mmm1'$, whose generating symmetries are represented by:

$$\begin{aligned}&M_x\mathcal{H}(k_x,k_y)M_x^{-1} = \sigma^x\mathcal{H}(-k_x,k_y)\sigma^x,\ M_z\mathcal{H}(k_x,k_y)M_z^{-1} = \sigma^z\mathcal{H}(k_x,k_y)\sigma^z, \\ &C_{4z}\mathcal{H}(k_x,k_y)C_{4z}^{-1} = \tau^z\left(\frac{\mathbb{1}_\sigma - i\sigma^z}{\sqrt{2}}\right)\mathcal{H}(k_y,-k_x)\tau^z\left(\frac{\mathbb{1}_\sigma + i\sigma^z}{\sqrt{2}}\right),\ \mathcal{T}\mathcal{H}(k_x,k_y)\mathcal{T}^{-1} = \sigma^y\mathcal{H}^*(-k_x,-k_y)\sigma^y.\end{aligned} \tag{2}$$

We also introduced the M_z- (and $\mathcal{I}$-) breaking, $\mathcal{T}$-symmetric potential $V_{M_z}(\mathbf{k})$ (Eq. (3) of the main text),

$$V_{M_z}(\mathbf{k}) = v_{M_z}\left[\tau^z\sigma^y\sin(k_x) - \tau^z\sigma^x\sin(k_y)\right], \tag{3}$$

and the M_z-, $\mathcal{I}$-, and $\mathcal{T}$-breaking, $M_z\times\mathcal{T}$- (and $\mathcal{I}\times\mathcal{T}$-) symmetric quadrupolar magnetic potential $U(\mathbf{k})$ (Eq. (4) of the main text):

$$U(\mathbf{k}) = u[\tau^y\sigma^y\sin(k_x) + \tau^y\sigma^x\sin(k_y)]. \tag{4}$$

In Supplementary Figure (SFig). 1, we show the relationships between the 2D insulating phases highlighted in Fig. 2 of the main text, which are realized using the parameters listed in Supplementary Table 1. Beginning with the trivial (uninverted) phase of the M_z- and $\mathcal{T}$-symmetric 2D Hamiltonian in Supplementary Equation (SEq.) (1), we tune v_m to invert bands with different C_{4z} eigenvalues and the same parity eigenvalues at Γ, while keeping the bulk bands uninverted at the other TRIM points. This induces a 2D spinful topological crystalline insulator (TCI)[32,44] with mirror Chern number $C_{M_z}=2$. This TCI phase can be reduced to an insulating phase with "fragile" topology[1–18] through the introduction of nonzero v_{M_z} in the M_z- and $\mathcal{I}$-breaking, $\mathcal{T}$-symmetric potential $V_{M_z}(\mathbf{k})$ in SEq. (3). The $C_{M_z}=2$ TCI phase can also be reduced to a $\mathcal{T}$-broken quadrupole insulating (QI) phase[26,28,39,65–73] through the introduction of nonzero u in the $\mathcal{T}$-, M_z-, and $\mathcal{I}$-breaking potential $U(\mathbf{k})$ in SEq. (4). Crucially, although these fragile and QI phases are topologically distinct, we show in Supplementary Note (SN) 9 that they both exhibit quadrupolar 0D corner modes with charges $\pm e/2 \mod e$ when half filled, *even though the occupied bands of the fragile phase are not Wannierizable,* i.e., *they do not admit a description in terms of symmetric, exponentially localized Wannier functions*[5,24,74,75]. By introducing the magnetic potential $U(\mathbf{k})$ into the fragile topological phase, or by introducing the $M_z\times\mathcal{T}$-breaking potential $V_{M_z}(\mathbf{k})$ into the QI in $p4/m'mm$, a QI can be realized in the type-I magnetic group[19,20] $p4mm$. We do not explicitly show an $\mathcal{I}\times\mathcal{T}$-broken QI phase in 2D, but we do present in Fig. 3(e,h) of the main text a 3D model of a Dirac semimetal in which an $\mathcal{I}\times\mathcal{T}$-broken QI phase occurs in 2D Brillouin zone (BZ) planes indexed by $k_z\neq 0,\pi$.

For the 3D semimetallic phases shown in Fig. 3 of the main text, we used the parameters listed in Supplementary Table 2 in the 3D Hamiltonians:

$$\mathcal{H}_{H1}(\mathbf{k}) = \mathcal{H}(\mathbf{k}) + U(\mathbf{k}) + t_H\tau^z\cos(k_z) \tag{5}$$

$$\mathcal{H}_{H2}(\mathbf{k}) = \mathcal{H}(\mathbf{k}) + U(\mathbf{k})\sin(k_z) + t_H\tau^z\cos(k_z). \tag{6}$$

SEq. (6) is invariant under the symmetries of space group (SG) 123 $P4/mmm1'$, whose generating symmetries are represented by:

$$\begin{aligned}&M_x\mathcal{H}(k_x,k_y,k_z)M_x^{-1} = \sigma^x\mathcal{H}(-k_x,k_y,k_z)\sigma^x,\ M_z\mathcal{H}(k_x,k_y,k_z)M_z^{-1} = \sigma^z\mathcal{H}(k_x,k_y,-k_z)\sigma^z, \\ &C_{4z}\mathcal{H}(k_x,k_y,k_z)C_{4z}^{-1} = \tau^z\left(\frac{\mathbb{1}_\sigma - i\sigma^z}{\sqrt{2}}\right)\mathcal{H}(k_y,-k_x,k_z)\tau^z\left(\frac{\mathbb{1}_\sigma + i\sigma^z}{\sqrt{2}}\right),\ \mathcal{T}\mathcal{H}(k_x,k_y,k_z)\mathcal{T}^{-1} = \sigma^y\mathcal{H}^*(-k_x,-k_y,-k_z)\sigma^y,\end{aligned} \tag{7}$$

whereas SEq. (5) is only invariant under magnetic SG $P4/m'mm$ (123.341 in the BNS notation[20]), whose generating symmetries are M_x, C_{4z}, and $M_z \times \mathcal{T}$ as represented in SEq. (7). Additionally, in Fig. 3(e,h) of the main text, we show a noncentrosymmetric Dirac semimetal in SG 99 $P4mm1'$ that results from breaking M_z (and $\mathcal{I}$) symmetry while keeping the other symmetries of SG 123 in $P4/mmm1'$ through the addition of SEq. (3) to SEq. (6).

All three of the Dirac semimetal phases listed in Supplementary Table 2 exhibit higher-order Fermi arcs (HOFAs) along their 1D hinges. The $M_z \times \mathcal{T}$-symmetric magnetic Dirac semimetal ($\mathcal{H}_{H1}(\mathbf{k})$ in SEq. (5)) and the M_z- and $\mathcal{T}$-symmetric Dirac semimetal ($\mathcal{H}_{H2}(\mathbf{k})$ in SEq. (6)) both exhibit twofold band degeneracies at generic k points due the presence of the combined antiunitary symmetry $\mathcal{I} \times \mathcal{T}$. Of these, the $\mathcal{T}$-symmetric semimetal also exhibits a topologically nontrivial plane at $k_z = 0$, for which the 2D mirror Chern number $C_{M_z} = 2$. Finally, by relaxing M_z (and $\mathcal{I}$) symmetry while keeping $\mathcal{T}$ by adding SEq. (3) to SEq. (6), we realize a previously uncharacterized variant of topological Dirac semimetal[32] without topological surface states, but with topological hinge states connected to the 0D corner states of a 2D fragile phase (Fig. 3(h) of the main text). Specifically, for the noncentrosymmetric Dirac semimetal phase of SEqs. (3) and (6), the Hamiltonian of the $k_z = 0$ plane exhibits fragile topology and Kramers pairs of corner modes, and the k_z planes between $k_z = 0$ and the Dirac points are equivalent to $M_z \times \mathcal{T}$-broken QIs in wallpaper group $p4m$ (layer group $p4mm$). This "fragile" topological Dirac semimetal therefore represents a gapless tuning cycle between a 2D fragile phase and a trivial insulator, similar to the gapped pumping cycles between 2D fragile and trivial phases that characterize higher-order TIs (HOTIs)[17]. Like with the (magnetic) HOTIs analyzed in Supplementary Reference (SRef.) 17, when trivial bands are added to the fragile topological Dirac semimetal phase of SEqs. (3) and (6), the Hamiltonian of the $k_z = 0$ plane no longer describes a fragile phase, but instead characterizes an obstructed atomic limit with the same corner modes as the fragile phase from which it originated (SN 4 and SRefs. 11 and 17).

2. Topological Equivalence of the Spinful s-d Hybridized Model and Spinless Flux-Threaded Model of Quadrupole Insulators

Here, we show that the spinful $s - d_{x^2-y^2}$-hybridized model of a QI introduced in this paper (Eqs. (1) and (4) of the main text, reproduced in SEqs. (1) and (4), respectively) is topologically equivalent to the spinless QI model with threaded flux introduced in SRef. 26. We begin with the $\mathcal{T}$-broken Hamiltonian formed by adding SEqs. (1) and (4):

$$\mathcal{H}_M(\mathbf{k}) = \mathcal{H}(\mathbf{k}) + U(\mathbf{k}). \tag{8}$$

SEq. (8) is invariant under the action of the type-III magnetic layer group $p4/m'mm$, which is generated by:

$$\{C_{4z}|00\},\ \{M_x|00\},\ \{M_z \times \mathcal{T}|00\}, \tag{9}$$

as well as the 2D lattice translations $T_{x,y}$. Throughout this work, we have employed the magnetic group labeling convention of SRefs. 19 and 20, for which the combined operation of mirror and time-reversal (for this layer group, the operation $M_z \times \mathcal{T}$) is denoted as m'. The dispersion relation of SEq. (8) can be calculated explicitly and compared to that of the quadrupole model in SRef. 26 (Eq. (6) in SRef. 26). Because the QI model introduced in SRef. 26 is particle-hole symmetric, we begin by tuning the quadrupole model introduced in this work (SEqs. (1) and (4)) to the particle-hole symmetric limit in which $t_{PH} = v_s = 0$, while keeping all of the other symmetries of $p4/m'mm$. In this limit, the spectrum of SEqs. (1) and (4) is given by:

$$\begin{aligned} E^2(\mathbf{k}) = t_1^2 \left[\cos^2(k_x) + \cos^2(k_y) + 2\cos(k_x)\cos(k_y)\right] + v_m^2 + t_2^2 \left[\cos^2(k_x) + \cos^2(k_y) - 2\cos(k_x)\cos(k_y)\right] \\ + 2t_1 v_m \left[\cos(k_x) + \cos(k_y)\right] + u^2 \left[\sin^2(k_x) + \sin^2(k_y)\right], \end{aligned} \tag{10}$$

where bands are doubly degenerate due to the presence of the combined antiunitary symmetry $\mathcal{I}\times\mathcal{T} = M_x M_y (M_z \times \mathcal{T})$. In the limit that:

$$t_1 = t_2 = \frac{1}{\sqrt{2}} u = t, \tag{11}$$

SEq. (10) reduces to:

$$E^2(\mathbf{k}) = 4t^2 + v_m^2 + 2t v_m \left[\cos(k_x) + \cos(k_y)\right]. \tag{12}$$

We can perform the substitution:

$$t = \frac{\lambda}{\sqrt{2}},\ v_m = \gamma\sqrt{2}, \tag{13}$$

under which the dispersion relation of this spinful $s-d$-hybridized model (SEq. (12)) becomes exactly equal to that of the spinless, flux-threaded model of a QI in SRef. 26 (Eq. (6) in SRef. 26). As with the earlier QI model in Eq. (6) in SRef. 26, our model displays gap closures at Γ for $\gamma/\lambda = -1$ and at M for $\gamma/\lambda = 1$. Calculating the nested Wilson loop of the lower two bands of SEq. (8), we confirm that they exhibit a nested Berry phase of 0 for $|\gamma/\lambda| > 1$ and a nested Berry phase of π for $|\gamma/\lambda| < 1$. This nested Berry phase is also symmetry-indicated by the eigenvalues of C_{4z} of the occupied corepresentations at Γ and M[28]. In both models, the representations of the mirror symmetries M_x and M_y anticommute at all four TRIM points, such that states at the TRIM points are twofold degenerate despite the absence of time-reversal symmetry. In the trivial insulating phases of both models, the occupied bands at both C_{4z}-invariant TRIM points have the same C_{4z} eigenvalues: $\lambda_{C_{4z}}(\Gamma) = \lambda_{C_{4z}}(M) = (1 \pm i)/\sqrt{2}$ or $\lambda_{C_{4z}}(\Gamma) = \lambda_{C_{4z}}(M) = -(1 \pm i)/\sqrt{2}$. In the QI phases of both models, either Γ or M has an occupied pair of states with C_{4z} eigenvalues $\lambda_{C_{4z}} = (1 \pm i)/\sqrt{2}$, whereas the other C_{4z}-invariant TRIM point has an occupied pair with $\lambda_{C_{4z}} = -(1 \pm i)/\sqrt{2}$. This can also be understood from the elementary band representations (EBRs) induced from the site-symmetry representations of spinful s and d orbitals[2–6,24,28,57–60,62,76–78] (see SN 3).

Furthermore, we can show that within each of the two phases of SEq. (8), there exist points in parameter space at which both models are unitarily related through a k-independent transformation. As the QI phase is distinguished from a trivial insulator with Wannier orbitals located on the unit-cell atoms (*i.e.* an unobstructed atomic limit[24]) by a $\mathbb{Z}_2$ topological invariant[26,28], it follows that all points in parameter space with the same symmetry are in topologically equivalent phases as long as one can tune between them without closing a gap[21,22,24,74,75,79–86]. Therefore, to demonstrate the overall topological equivalence between the two models, we only need to demonstrate the topological equivalence between them at one point in parameter space within each of the two gapped phases of the different models.

We begin in the limit that both models have the same dispersion relations. The model of a QI introduced in Eq. (6) of SRef. 26 can be written as:

$$\mathcal{H}'(\mathbf{k}) = (\gamma + \lambda\cos(k_x))\Gamma_1 + (\gamma + \lambda\cos(k_y))\Gamma_2 + \lambda\sin(k_x)\Gamma_3 + \lambda\sin(k_y)\Gamma_4, \tag{14}$$

whereas the $\mathcal{T}$-broken $s-d$-hybridized model in SEq. (8) with the substitutions in SEqs. (11) and (13) can be written as

$$\mathcal{H}_M(\mathbf{k}) = \left(\gamma\sqrt{2} + \frac{\lambda}{\sqrt{2}}[\cos(k_x) + \cos(k_y)]\right)\tilde{\Gamma}_1 + \frac{\lambda}{\sqrt{2}}\left(\cos(k_x) - \cos(k_y)\right)\tilde{\Gamma}_2 + \lambda\sin(k_x)\tilde{\Gamma}_3 + \lambda\sin(k_y)\tilde{\Gamma}_4, \tag{15}$$

where $\{\Gamma_i\}$ and $\{\tilde{\Gamma}_i\}$ are sets of four-component Dirac matrices that each separately form a Clifford algebra. Without loss of generality, we choose the matrix representations:

$$\Gamma_i = \tilde{\Gamma}_i, \text{ for } i = 1 \text{ to } 5, \tag{16}$$

and develop for each phase an exact transformation matrix at one point in parameter space. We note that, because the coefficients of $\Gamma_{3,4}$ and $\tilde{\Gamma}_{3,4}$ in SEqs. (14) and (15), respectively, are proportional to the same functions of k, we expect that the k-dependence of the unitary transformation that maps $\mathcal{H}'(\mathbf{k})$ to $\mathcal{H}_M(\mathbf{k})$ will lie in the subspace of $\Gamma_{1,2}$ for generic values of k.

We first consider relating the two models' trivial (normal) insulating (NI) phases, defined as exhibiting a nested Berry phase of 0. The quadrupole moments of the insulating phases of both Hamiltonians are entirely determined by a single parameter: the ratio $|\gamma/\lambda|$. Both models are gapless only when $|\gamma| = |\lambda|$, and both are topologically trivial for $|\gamma| > |\lambda|$. We first consider the limit $\lambda \to 0$, which defines:

$$\mathcal{H}'_{NI}(\mathbf{k}) = (\gamma)\Gamma_1 + (\gamma)\Gamma_2, \tag{17}$$

and

$$\mathcal{H}_{M,NI}(\mathbf{k}) = (\gamma\sqrt{2})\Gamma_1. \tag{18}$$

Expressing the Hamiltonians as vectors of coefficients of the Dirac matrices:

$$\mathcal{H}_M(\mathbf{k}) = \mathbf{v}(\mathbf{k}) \cdot \mathbf{\Gamma},\ \mathcal{H}'(\mathbf{k}) = \mathbf{v}'(\mathbf{k}) \cdot \mathbf{\Gamma}, \tag{19}$$

it is trivial to show that for $v' = \bar{V}v$,

$$\bar{V} = \begin{pmatrix} R\left(\frac{\pi}{4}\right) & \mathbf{0} \\ \mathbf{0} & \mathbb{1} \end{pmatrix}, \tag{20}$$

where $R\left(\frac{\pi}{4}\right)$ is an orthogonal rotation matrix in the 2×2 $\Gamma_{1,2}$ subspace:

$$R\left(\frac{\pi}{4}\right) = \begin{pmatrix} 1/\sqrt{2} & -1/\sqrt{2} \\ 1/\sqrt{2} & 1/\sqrt{2} \end{pmatrix}. \tag{21}$$

As $\bar{V}$ is k-independent, both models' trivial phases are topologically equivalent.

We now relate the QI phases of both models. A characteristic point within this phase can be obtained by choosing $\gamma/\lambda = 0$, which defines:

$$\mathcal{H}'_{QI}(\mathbf{k}) = \lambda\cos(k_x)\Gamma_1 + \lambda\cos(k_y)\Gamma_2 + \lambda\sin(k_x)\Gamma_3 + \lambda\sin(k_y)\Gamma_4 \tag{22}$$

and

$$\mathcal{H}_{M,QI}(\mathbf{k}) = \frac{\lambda}{\sqrt{2}}\left(\cos(k_x)+\cos(k_y)\right)\Gamma_1 + \frac{\lambda}{\sqrt{2}}\left(\cos(k_x)-\cos(k_y)\right)\Gamma_2 + \lambda\sin(k_x)\Gamma_3 + \lambda\sin(k_y)\Gamma_4. \tag{23}$$

Again expressing the transformation matrix $\bar{V}$ in terms of the vectors of the coefficients of the Dirac matrices (SEq. (19)) for each Hamiltonian, the transformation $v' = \bar{V}v$ is satisfied by:

$$\bar{V} = \begin{pmatrix} S\left(\frac{\pi}{4}\right) & \mathbf{0} \\ \mathbf{0} & \mathbb{1} \end{pmatrix}, \tag{24}$$

where $S\left(\frac{\pi}{4}\right)$ is an orthogonal rotoinversion matrix in the 2×2 $\Gamma_{1,2}$ subspace:

$$S\left(\frac{\pi}{4}\right) = \begin{pmatrix} 1/\sqrt{2} & 1/\sqrt{2} \\ 1/\sqrt{2} & -1/\sqrt{2} \end{pmatrix}. \tag{25}$$

As $\bar{V}$ is here also k-independent, both models' QI phases are topologically equivalent.

3. Quadrupole Insulators in Spinful Magnetic Wallpaper Group $p4m$ as Obstructed Atomic Limits

We analyze the EBRs[2,24,57–59,76,77] of the four-band, $\mathcal{T}$-broken model of a $s-d_{x^2-y^2}$-hybridized quadrupole insulator (QI) introduced in this paper (SEqs. (1) and (4)). In this section, we show that the QI phase of this model, and consequently the QI phase of the topologically equivalent model introduced in SRef. 26 (SN 2), is an obstructed atomic limit[24] of a 2D magnetic wallpaper (or layer) group. Our model consists of two spin-1/2 s and two spin-1/2 $d_{x^2-y^2}$ orbitals at the $1a$ Wyckoff position of type-I magnetic wallpaper group $p4m$, which is generated by C_{4z} and M_x:

$$\{C_{4z}|00\},\ \{M_x|00\}, \tag{26}$$

as well as the 2D lattice translations $T_{x,y}$. Our analysis in this section will also apply to the type-III magnetic layer group[20] $p4/m'mm$, which has an additional $\mathcal{I}\times\mathcal{T}$ symmetry:

$$\{\mathcal{I}\mathcal{T}|00\}, \tag{27}$$

such that:

$$p4/m'mm = (E)p4m \cup (\mathcal{I}\times\mathcal{T})p4m, \tag{28}$$

where E is the identity operation. By examining all momentum space (co)representations of these two groups[19,24,57–59], we find that the additional generator that separates them, $\mathcal{I}\times\mathcal{T}$, does not increase the connectivity of any of the double valued EBRs of either 2D magnetic group. More specifically, because the little co-groups of the Γ and M points are isomorphic to $4mm$, and the little co-group of the X point is isomorphic to $mm2$ (Fig. 2(b) of the main text), the double-valued (co)representations at all of the TRIM points are already twofold degenerate whether or not $\mathcal{I}\times\mathcal{T}$ is present; the additional antiunitary symmetry simply serves in $p4/m'mm$ to enforce twofold band degeneracies at all of the other points in the BZ (and to make the Wilson loop particle-hole symmetric at each k point[27,87–90]). Here, and throughout this work, the little co-group is defined as the little group modulo lattice translations[19,24]. We also

note that, in position space, the addition to $p4m$ of $\{\mathcal{I}\mathcal{T}|00\}$ does not change the locations, multiplicities, or group-subgroup relations of the site-symmetry groups of any of the Wyckoff positions. Therefore, we can perform the more general analysis here of the Wannier description of band representations of wallpaper group $p4m$, and conclude that the same analysis applies to its supergroup $p4/m'mm$. As the original model of a QI is topologically equivalent to a spinful model in $p4/m'mm$ (SN 2), and as the models introduced in this work (SN 1) describe QIs in both $p4/m'mm$ (SEqs. (1) and (4)) and $p4m$ ((SEqs. (1), (3), and (4)) our more general analysis in this section of (obstructed) atomic limits in $p4m$ applies to both the original spinless QI model introduced in SRef. 26 as well as to all of the QI models introduced in this work.

To understand the band representations that correspond to the QI phase, we first examine the C_{4z}-invariant, multiplicity-one maximal Wyckoff positions of the type-I magnetic wallpaper group $p4m$ and the irreducible representations of their site-symmetry groups. The site-symmetry groups of the $1a$ and $1b$ Wyckoff positions of $p4m$ are isomorphic to the point group $4mm$, and the site-symmetry group of the $2c$ position is isomorphic to the point group $mm2$ (Fig. 2(a) of the main text). Consulting the point group tables on the Bilbao Crystallographic Server (BCS)[24,40], we find that a spinless s orbital transforms as the 1D single-valued irreducible representation A_1, whereas a spinless $d_{x^2-y^2}$ orbital, whose wavefunction is odd under diagonal mirror $M_{x\pm y}$ and C_{4z}, transforms as the irreducible representation B_1. To add spin, we consult the direct product tables of Altmann and Herzig (Table 52.9 on page 491 of SRef. 91),

$$A_1 \otimes \bar{E}_{1/2} = \bar{E}_{1/2} \equiv \bar{E}_1,\ B_1 \otimes \bar{E}_{1/2} = \bar{E}_{3/2} \equiv \bar{E}_2, \tag{29}$$

where for convenience, we provide double-valued irreducible representations in both the notation of Altmann and Herzig[91] ($\bar{E}_{1/2,3/2}$) and that of the BCS[40,57–59] ($\bar{E}_{1,2}$). The notation of Altmann and Herzig in particular provides some physical intuition for these representations: $\bar{E}_{1/2}$ corresponds to the spin-1/2 representation of a spinful s orbital, and $\bar{E}_{3/2}$ corresponds to the $m_j = \pm 3/2$ piece of the $J = 5/2$ representation of spinful d orbitals[91–95]. Both double-valued irreducible representations $\bar{E}_{1,2}$ are two-dimensional, and their dimension does not double when $\mathcal{T}$ (or $\mathcal{I} \times \mathcal{T}$) is introduced. These representations correspond to doubly-degenerate pairs of spinful atomic orbitals with different complex-conjugate pairs of C_{4z} eigenvalues:

$$\chi_{\bar{E}_1}(C_{4z}) = \frac{1+i}{\sqrt{2}} + \frac{1-i}{\sqrt{2}} = \sqrt{2},\ \chi_{\bar{E}_2}(C_{4z}) = \frac{-1+i}{\sqrt{2}} + \frac{-1-i}{\sqrt{2}} = -\sqrt{2}, \tag{30}$$

where $\chi_\rho(h)$ is the character of the unitary symmetry h in the irreducible representation ρ, and is equal to the sum of the eigenvalues of h in ρ. The twofold degeneracy of states characterized by $\bar{E}_{1,2}$ is here enforced by $\{M_x, M_y\} = 0$, instead of $\mathcal{T}$, which is absent for the calculations in this section (we will later also enforce $\mathcal{T}$ symmetry in SN 4).

Finally, before detailing the EBRs of $p4m$, we establish the set of possible little co-group representations at the high-symmetry points in the BZ (Fig. 2(b) of the main text). Because wallpaper group $p4m$ is symmorphic, it is sufficient to examine the little co-group at each high-symmetry point. The little co-groups of Γ and M (Fig. 2(b) of the main text) are isomorphic to $4mm$, which has two double-valued representations, denoted $\bar{\rho}_{6,7}$. When we are referring to the little co-group representation at Γ, we will write:

$$\bar{\Gamma}_7 \equiv \bar{E}_1 \equiv \bar{\rho}_7,\ \bar{\Gamma}_6 \equiv \bar{E}_2 \equiv \bar{\rho}_6, \tag{31}$$

while at M:

$$\bar{M}_7 \equiv \bar{E}_1 \equiv \bar{\rho}_7,\ \bar{M}_6 \equiv \bar{E}_2 \equiv \bar{\rho}_6, \tag{32}$$

such, that, incorporating SEq. (30), the characters of the little co-group representations $\bar{\rho}_{6,7}$ are:

$$\chi_{\bar{\rho}_6}(C_{4z}) = -\sqrt{2},\ \chi_{\bar{\rho}_7}(C_{4z}) = \sqrt{2}. \tag{33}$$

At the X and X' points, the little co-groups are isomorphic to the double-valued point group $mm2$, which has only a single double-valued irreducible representation $\bar{E}$, which is two-dimensional and corresponds to a pair of states with $M_{x,y}$ eigenvalues $\pm i$. This twofold degeneracy does not require time-reversal symmetry; instead, it is enforced by the anticommutation relation $\{M_x, M_y\} = 0$.

In the four-band quadrupole Hamiltonian (SEqs. (1) and (4)), the $\mathcal{T}$-symmetric spin-orbit coupling term $v_s \tau^y \sigma^z \sin(k_x)\sin(k_y)$ and quadrupolar magnetic term $u[\tau^y \sigma^y \sin(k_x) + \tau^y \sigma^x \sin(k_y)]$ vanish at the Γ ($k_x = k_y = 0$) and M ($k_x = k_y = \pi$) points, and the Hamiltonian at these points takes the simplified form:

$$\mathcal{H}(\Gamma) = (2t_1 + v_m)\tau^z + 2t_{PH}\mathbb{1}_{\tau\sigma},\ \mathcal{H}(M) = (-2t_1 + v_m)\tau^z - 2t_{PH}\mathbb{1}_{\tau\sigma}, \tag{34}$$

where eigenstates can be indexed by their *spinless* fourfold rotation eigenvalues. Most precisely, because none of the terms in SEq. (34) contain σ matrices, SEq. (34) exhibits $SU(2)$ spin rotation symmetry, in addition to the spinful C_{4z} symmetry inherited from SEqs. (1) and (4):

$$C_{4z} = \tau^z \left(\frac{\mathbb{1} - i\sigma^z}{\sqrt{2}} \right). \tag{35}$$

The combination of $SU(2)$ spin rotation symmetry and spinful C_{4z} results in an additional spinless fourfold rotation symmetry, represented at the Γ and M points by:

$$\tilde{C}_{4z} = \tau^z. \tag{36}$$

The states that transform as s (d) orbitals correspond to its positive (negative) eigenvalues. We find that the trivial and QI phases of the $s-d$-hybridized model introduced in this work can be distinguished by the irreducible representations of their occupied bands at Γ and at M, which are determined by the numerical prefactors of τ^z in SEq. (34) using the values in Supplementary Table 1. The trivial phase displays either $\bar{\rho}_7$ or $\bar{\rho}_6$ at both Γ and at M; the QI phase displays $\bar{\rho}_6$ at Γ and $\bar{\rho}_7$ at M, or $\bar{\rho}_7$ at Γ and $\bar{\rho}_6$ at M, in agreement with the results of SRef. 28.

To relate these momentum-space representations to EBRs, we induce the site-symmetry irreducible representations of spinful s and $d_{x^2-y^2}$ orbitals ($\bar{E}_{1,2}$, respectively) at the $1a$ and $1b$ Wyckoff positions into $G = p4m$ and then subduce onto Γ and M. As we have previously shown that the only symmetry indicators in $p4m$ are the pairs of C_{4z} eigenvalues within each occupied irreducible representation at Γ and at M, we can deduce all of the relevant information by calculating the character of C_{4z} in the subduced representations at the Γ and M points following the procedure in SRef. 59. Since the Wyckoff positions are multiplicity-one, the formula simplifies to:

$$\chi_G^{\mathbf{k}}(h) \equiv e^{-i(R\mathbf{k})\cdot\mathbf{t}} \tilde{\chi}[\rho(\{E| - \mathbf{t}\}h)], \tag{37}$$

where:

$$\tilde{\chi}[\rho(g)] = \begin{cases} \chi[\rho(g)] & \text{if } g \in G_{\mathbf{q}} \\ 0 & \text{if } g \notin G_{\mathbf{q}}, \end{cases} \tag{38}$$

and:

$$\mathbf{t} \equiv h\mathbf{q} - \mathbf{q}. \tag{39}$$

In SEqs. (37) – (39), $G_{\mathbf{q}}$ is the site-symmetry group at $\mathbf{q}$, h is an element of the little group at $\mathbf{k}$ and R is the rotational part of h, such that $h = \{R|v\}$. Applying SEq. (39) to $h = \{C_{4z}|00\}$, we find that for the $1a$ position, $\mathbf{t} = h\mathbf{q}_{1a} - \mathbf{q}_{1a} = \mathbf{0}$, while for the $1b$ position, $\mathbf{t} = h\mathbf{q}_{1b} - \mathbf{q}_{1b} = -\mathbf{T}_x$, where $\mathbf{T}_x$ denotes a lattice translation in the x direction. Thus, from SEq. (37), an EBR induced from the $1a$ position has characters:

$$\chi^{\Gamma}(C_{4z}) = \chi^{M}(C_{4z}) = \tilde{\chi}^{1a}(C_{4z}), \tag{40}$$

While for the $1b$ position,

$$\chi^{\Gamma}(C_{4z}) = -\chi^{M}(C_{4z}) = \tilde{\chi}^{1b}(C_{4z}). \tag{41}$$

The two possible EBRs induced from each site are described in Supplementary Table 3; the same information can be found using the BANDREP tool on the BCS[24,57–59]. Each of the EBRs shown in this table is twofold connected, with nondegenerate bands along all lines and the plane of the BZ away from the TRIM points and with twofold degeneracies at the TRIM points where $M_{x,y}$ intersect and anticommute. In $p4/m'mm$, the layer supergroup of $p4m$, all of the EBRs maintain the same connectivity and symmetry eigenvalues. In wallpaper group $p4m$, singly degenerate bands along the four high-symmetry mirror lines $M_{x,y}$ and $M_{x\pm y}$ can be labeled with either $\pm i$ mirror eigenvalues and bands at the TRIM points come in doublets with complex conjugate pairs of mirror eigenvalues. Conversely, in layer group $p4/m'mm$, all of the bands along the mirror lines and at the TRIM points come in doublets with complex conjugate pairs of mirror eigenvalues related by $\mathcal{I} \times \mathcal{T}$ symmetry. Furthermore, in $p4/m'mm$, bands at generic momenta in the 2D BZ also appear in doublets protected by $\mathcal{I} \times \mathcal{T}$ symmetry. Therefore, when bands are inverted at Γ in our four-band model in $p4/m'mm$ (SEqs. (1) and (4)), the model is only gapless at a single point in parameter space at Γ. Conversely, for a four-band model in $p4m$ with two occupied bands, depending on the band dispersion, the model either directly transitions from a trivial phase to a QI (as occurs in the fragile topological Dirac semimetal in Fig. 3(e,h) of the main text), or instead transitions from a trivial insulator (or QI) into a 2D semimetal with $8n$

twofold degenerate nodal points protected by $M_{x,y}$ and $M_{x\pm y}$ symmetries[56]. In this work, we specifically restrict consideration to models in $p4m$ that transition between trivial insulators and QIs when bands are inverted at Γ.

We now show that the QI phase that results from inverting bands originating from spinful s and d orbitals at the $1a$ position of $p4m$ is an obstructed atomic limit with Wannier centers at the $1b$ position. From the contents of Supplementary Table 3, it is clear that when $1a$ bands from s and d orbitals are inverted at Γ in a four-band model, the bottom two bands will have the same symmetry eigenvalues as bands from the $1b$ position ($\bar{\rho}_6$ at Γ and $\bar{\rho}_7$ at M). To formally show that the resulting insulator is an obstructed atomic limit, we look at the common Wyckoff position $4d$ (Fig. 2(a) of the main text), which lies at $(\pm x, \pm x)$, such that:

$$G_{1a} \cap G_{1b} = G_{4d}, \tag{42}$$

where G_{4d} is isomorphic to point group[30,31,40] m, as its only symmetry is $M_{x\pm y}$. For both of the one-dimensional irreducible representations ${}^1\bar{E}$ and ${}^2\bar{E}$ of G_{4d} we find that[19,57]:

$$({}^{1,2}\bar{E})_{4d} \uparrow G_{1a} = (\bar{E}_1)_{1a} \oplus (\bar{E}_2)_{1a},\ \ ({}^{1,2}\bar{E})_{4d} \uparrow G_{1b} = (\bar{E}_1)_{1b} \oplus (\bar{E}_2)_{1b}, \tag{43}$$

where $(\bar{\sigma})_{\mathbf{q}}$ is the EBR induced from the site-symmetry representation $\bar{\sigma}$ of the site-symmetry group of the Wyckoff position at $\mathbf{q}$. From this we can conclude the equivalence[2–6,24,57–59,76,77,96]:

$$[\bar{E}_1 \oplus \bar{E}_2]_{1a} \uparrow G \equiv [\bar{E}_1 \oplus \bar{E}_2]_{1b} \uparrow G, \tag{44}$$

where $G = p4m$. To define just $(\bar{E}_2)_{1b}$ in terms of other EBRs, we introduce the symbol $\ominus$,

$$[\bar{E}_1 \oplus \bar{E}_2]_{1a} \uparrow G \ominus [\bar{E}_1]_{1b} \uparrow G \equiv [\bar{E}_2]_{1b} \uparrow G, \tag{45}$$

where an equivalence formed with $\ominus$ is only defined if a corresponding equivalence with $\oplus$ of the form of SEq. (44) is also defined. From this it is clear that we can realize, via induction of four bands from the $1a$ position and band inversion, an occupied pair of bands that is equivalent to an EBR induced from the $1b$ position, or an obstructed atomic limit[2,24]. This highlights the direct similarity between the QI and the nontrivial (obstructed atomic limit) phase of the SSH model. In the SSH chain, which is a 1D chain with inversion centers at the $1a$ and $1b$ positions, the induction of two bands from the $1a$ position and band inversion analogously gives, at half filling, an occupied band that is equivalent to an EBR induced from the $1b$ position[24] (SFig. 2(a,b)). Furthermore, more physically, the SSH transition can be expressed as $s-p$ hybridization[21,22,24], whereas in this section we have shown that the QI phase results from $s-d$ hybridization.

SEq. (45) shows that the four bands corresponding to spinful s and d orbitals at the $1a$ position of $p4m$ can realize a trivial insulator at half-filling; that is, the valence (and conduction) bands possess localized, symmetric Wannier functions centered at the $1a$ position. When the gap closes and reopens, the valence bands still possess a Wannier description, but one which instead corresponds to s or d orbitals centered at the $1b$ position. In the intermediate gapless regime, it is not well-defined to compute the Wannier functions of only two bands, but one can compute the Wannier functions of *both* the valence and conduction bands and observe that they correspond to orbitals centered at the $4d$ position (x, x), where x is a gauge-dependent quantity[24,25]. We view this entire process as a Wannier center homotopy, where the gap closing and reopening "slides" the Wannier orbitals along the $4d$ position in a $p4m$-symmetric manner (SFig. 2(c)), ultimately realizing an atomic insulator with Wannier centers lying on a different Wyckoff position ($1b$) than the ionic centers ($1a$) (SFig. 2(d)). This corresponds to an obstructed atomic limit in the language of SRefs. 2 and 24.

We emphasize that the transition between a trivial insulator and the QI obstructed atomic limit in $p4m$ can only be realized as a function of a single-parameter in a spinful (double-valued) 2D magnetic group; unlike with the spinful SSH or Rice-Mele chains[21,22], there is no analogous $\mathcal{T}$-symmetric limit of the quadrupole as an obstructed atomic limit (as opposed to a bipartite atomic-limit transition) with the symmetries of $p4m$ and single-parameter phase transitions. Specifically, the singly-degenerate (magnetic spinful or spinless)[21,22] and $\mathcal{T}$-symmetric (spinful, nonmagnetic)[97] formulations of the SSH chain are both Wannierizable[24], and differ from trivial insulators (unobstructed atomic limits) by phase transitions characterized by a single parameter[21,22,97] (analogous to γ/λ in the text following SEq. (13)). Conversely, as we will show in SN 4, the Wannier description of the QI established in this section is no longer valid when $\mathcal{T}$ symmetry is restored; instead the analogous set of occupied bands exhibits fragile topology. Since topological phases that are not Wannierizable do not exist in 1D[87,98], this fragile phase represents one of the simplest topological obstructions to forming an obstructed atomic limit (though another commonly cited example occurs in magnetic layer groups with only $\mathcal{I}$ symmetry[11,12,14,17,18,87]). In fact, the results of SN 4 imply that with both $\mathcal{T}$ symmetry and the symmetries of $p4m$, an obstructed atomic limit from $1a$ to $1b$ can only be realized with a minimum of eight total bands (specifically because Wannier orbitals at the intermediate $4d$ (x, x) position must be doubly degenerate). An example of a closely related obstructed atomic limit with four occupied (and four unoccupied) bands in $p41'$ ($p4m1'$

with broken $M_{x,y}$) was introduced in SRef. 39, and is implied in that work to differ from a trivial insulator through a phase transition that is a function of more than one parameter (when only physical (space/layer group) symmetries are enforced). Furthermore, we can show that eight-band phase transitions between $1a$ trivial insulators and $1b$ obstructed atomic limits in $p4m1'$ must also be functions of more than one parameter. Specifically, because neither wallpaper group $p4m1'$ nor its layer supergroup $p4/mmm1'$ host symmetry-stabilized four-dimensional corepresentations[19,27,56] (though there is a single four-dimensional (including spin) corepresentation when SOC is neglected[19,57]), then all band-inversion transitions in $p4m1'$ and $p4/mmm1'$ involving eight bands must be functions of more than one parameter, and cannot occur simultaneously without fine tuning. Therefore, instead of being driven by a single band inversion, like the QI[26,28], an obstructed atomic limit with four valence and four conduction bands from $1a$ to $1b$ in $p4m1'$ generically represents an example of a 2D corner-mode phase driven by "double band inversion", analogous to the $\mathcal{I}$-symmetric corner-mode phases analyzed in SRefs. 10, 11, 13, 14, and 17.

Finally, we analyze the multipole moments of the QI phase and compare them to the dipole moment of the spinful, $\mathcal{T}$-broken (magnetic) SSH chain[21,22]. The spinful magnetic SSH chain can be considered a spin-polarized half-filled 1D crystal in magnetic rod group $(p\bar{1})_{\text{RG}}$ (SRef. 20 and 23 and SN 12), which is generated by:

$$\{\mathcal{I}|0\},\ \{E|1\}, \tag{46}$$

where E is the identity operation. We consider this chain to have lattice spacing a and two total bands originating from spin-up s and p orbitals at the $1a$ position ($x = 0$) with a gap at half filling; the lower (occupied) and upper (unoccupied) bands each form a Wannier orbital, initially located at the $1a$ position (SFig. 2(a)). When a gap is closed between the occupied and unoccupied bands at a 1D TRIM point, a Wannier description of the valence and conduction bands taken together is still admitted, and the two Wannier orbitals of these bands may then "slide" along the general position $2c$ (x) in an $\mathcal{I}$-symmetric manner. More precisely, the location $\pm x$ of these two Wannier orbitals becomes a gauge-dependent quantity[24,25]. If the two Wannier orbitals remain at the $1a$ position or slide all the way to the $1b$ position ($x = a/2$), a Wannier description of only the lower band in energy is again permitted after a symmetry-preserving gap is reopened, and specifically characterizes an obstructed atomic limit if the Wannier orbital of the occupied band occupies the $1b$ position[24]. In this obstructed atomic limit, the inversion eigenvalues of the occupied band indicate whether it is induced from a spinful s or p orbital at the $1b$ position[24]. To calculate the dipole moment of each unit cell of the spinful magnetic SSH obstructed atomic limit, we use the charge counting depicted in SFig. 2(b) in which an uncovered atom (charge $+e$) lies at $x = 0$ ($1a$) and an electron occupying a Wannier orbital (charge $-e$) lies at $x = a/2$ ($1b$):

$$P^{SSH} = \sum_i q_i x_i = -(e)(a/2) = -ae/2. \tag{47}$$

We confirm that P^{SSH} is nontrivial by calculating:

$$\left(\frac{1}{a}\right) P^{SSH} \bmod \mathrm{e} = e/2, \tag{48}$$

which coincides with the established value for the polarization of the singly-degenerate (magnetic) SSH chain[21,22].

For the QI phase, we now exploit the Wannier center homotopy to perform the analogous analysis. As established in this section and depicted in SFig. 2(d), the QI is an obstructed atomic limit in magnetic wallpaper group $p4m$ formed from ions (charge $+2e$) lying at $(x, y) = (0, 0)$ ($1a$) and two occupied Wannier orbitals (charge $-2e$) lying at $(a/2, a/2)$ ($1b$). First, using SEq. (47), we calculate the x- and y-directed dipole moments of the QI unit cell:

$$P_x^{QI} = \sum_i q_i x_i = -2e(a/2) = -ae,\ P_y^{QI} = \sum_i q_i y_i = -2e(a/2) = -ae. \tag{49}$$

As in SEq. (48), to determine whether $P_{x,y}^{QI}$ are nontrivial, we calculate the dipole moments in the units of a (*i.e.* the dipole densities) modulo e:

$$\left(\frac{1}{a}\right) P_x^{QI} \bmod \mathrm{e} = \left(\frac{1}{a}\right) P_y^{QI} \bmod \mathrm{e} = 0, \tag{50}$$

which reveals that both $P_{x,y}^{QI}$ exhibit the same values as the trivial phase of the spinful magnetic SSH chain (SFig. 2(a,b)). We then calculate the bulk quadrupole moment per unit cell using the standard formulation in SRef. 99:

$$Q^{ab} = \frac{1}{2}\sum_i q_i(3r_i^a r_i^b - |\mathbf{r}_i|^2\delta^{ab}), \tag{51}$$

finding that Q^{xy}, in particular, is nonzero (SFig. 2(d)):

$$Q^{xy,QI} = \frac{3}{2}\sum_i q_i x_i y_i = -\frac{3}{2}\left(\frac{2ea^2}{4}\right) = -\left(\frac{3a^2}{2}\right)\frac{e}{2}. \quad (52)$$

As previously with the dipole moments in SEqs. (48) and (50), Q^{xy} can also be expressed as a multipole (quadrupole) density with the units of charge[26]. Analogously to the simplest dipole, which is a rod centered at the origin with length a and alternating charges $\pm e$ on its ends, the simplest xy-quadrupole is a square centered at the origin with side-length a and alternating charges $\pm e$ on its corners[99], for which $Q^{xy} = 3a^2/2$ (SEq. (52)). We therefore express $Q^{xy,QI}$ in the reduced units of $3a^2/2$ and confirm that it is nontrivial by calculating its value modulo[26] e:

$$\left(\frac{2}{3a^2}\right) Q^{xy,QI} \text{ mod e} = e/2, \quad (53)$$

which agrees with the value obtained for the spinless, flux-threaded QI model in SRef. 26.

4. Fragile Topology in Wallpaper Group $p4m1'$

In this section, we explore the consequences of restoring $\mathcal{T}$-symmetry to wallpaper group $p4m$ (SN 3). We therefore analyze the EBRs of the $\mathcal{T}$-symmetric wallpaper group $p4m1'$, and apply the group theory of band representations[2–6,24,28,57–60,62,76–78] to a $\mathcal{T}$-symmetric, $\mathcal{M}_z$-broken limit of the 2D model introduced in this work (SEqs. (1) and (3)). We show that, despite respecting the same unitary symmetries and exhibiting the same eigenvalues as a QI (SN 3), this model instead hosts two valence and two conduction bands that exhibit "fragile," topology[1–18] as a consequence of the additional presence of $\mathcal{T}$ symmetry.

We again consider spinful s and d orbitals at the $1a$ position of a wallpaper group with C_{4z} and $M_{x,y}$. However, we now also impose $\mathcal{T}$ symmetry, such that the symmetry group is instead the wallpaper group:

$$G = p4m1', \quad (54)$$

the type-II nonmagnetic[20] ($\mathcal{T}$-symmetric) supergroup of the QI wallpaper group $p4m$ (SN 3). The site-symmetry groups of the $1a$ and $1b$ positions of G are isomorphic to $4mm1'$. The corepresentations of $4mm1'$ have the same symmetry eigenvalues and dimensions as the irreducible representations of its unitary subgroup $4mm$, namely $\bar{E}_{1,2}$, whose C_{4z} eigenvalues are given in SEq. (33). Therefore, the EBRs induced from these corepresentations display the same symmetry eigenvalues as those in Supplementary Table 3.

However, we observe an important difference between the $\mathcal{T}$-broken case (SN 3) and the $\mathcal{T}$-symmetric case. If we start with a four-band, $\mathcal{T}$-symmetric model whose two valence (conduction) bands are equivalent to EBRs induced from s (d) orbitals on the $1a$ position and then invert bands at Γ, the resulting valence (conduction) bands have the same little group corepresentations at Γ and at M as the EBRs induced from d (s) orbitals at the $1b$ position. Therefore, they display the same little group corepresentations as the valence bands of a $\mathcal{T}$-broken QI in $p4m$. However, when we calculate the x-directed Wilson loop of the $\mathcal{T}$-symmetric valence bands (Fig. 2(k) of the main text), it now *winds*. This winding indicates that the valence bands are not Wannierizable[24,74,75,83], and therefore reveals that they do not characterize an obstructed atomic limit like the QI phase previously analyzed in SN 3. Instead, as we will show in this section, the presence of $\mathcal{T}$ symmetry enforces a Kramers doubling at the $4d$ Wyckoff position of $p4m1'$ that obstructs the formation of the Wannier center homotopy of the QI (SFig. 2(c)). This obstruction prevents the two occupied bands from being smoothly deformed into EBRs induced from the $1b$ position. Specifically, unlike previously in SN 3, the two occupied bands are no longer homotopically equivalent to a $1b$ atomic limit in the presence of $\mathcal{T}$ symmetry[24,59].

We formally reveal the obstruction by again attempting to form a homotopy through the $4d$ position at (x, x), which was previously accomplished successfully in SN 3 for the type-I magnetic wallpaper group $p4m$. We again look at the $4d$ Wyckoff position, whose site-symmetry group satisfies:

$$G_{1a} \cap G_{1b} = G_{4d}. \quad (55)$$

In the presence of $\mathcal{T}$ symmetry, G_{4d} is isomorphic to $m1'$. Therefore, unlike in SN 3 where the $4d$ position admitted two one-dimensional irreducible representations ${}^{1,2}\bar{E}$ distinguished by their mirror eigenvalues $\pm i$, here $\mathcal{T}$ symmetry enforces that G_{4d} has only a single, *two-dimensional* corepresentation[19,57] ${}^1\bar{E}{}^2\bar{E}$. When induced into its site-symmetry supergroups G_{1a} and G_{1b}, ${}^1\bar{E}{}^2\bar{E}$ crucially results in a sum of *twice* as many EBRs as previously (SEq. (43)):

$$({}^1\bar{E}{}^2\bar{E})_{4d} \uparrow G_{1a} = (2\bar{E}_1)_{1a} \oplus (2\bar{E}_2)_{1a}, \;\; ({}^1\bar{E}{}^2\bar{E})_{4d} \uparrow G_{1b} = (2\bar{E}_1)_{1b} \oplus (2\bar{E}_2)_{1b}, \quad (56)$$

where $(\bar{\sigma})_{\mathbf{q}}$ is the EBR induced from the site-symmetry representation $\bar{\sigma}$ of the Wyckoff position at $\mathbf{q}$, and where:

$$(2\bar{E}_i)_{\mathbf{q}} = (\bar{E}_i)_{\mathbf{q}} \oplus (\bar{E}_i)_{\mathbf{q}}. \tag{57}$$

From this we can conclude the equivalence of band representations:

$$[2\bar{E}_1 \oplus 2\bar{E}_2]_{1a} \uparrow G \equiv [2\bar{E}_1 \oplus 2\bar{E}_2]_{1b} \uparrow G, \tag{58}$$

SEq. (58) shows that the set of eight bands consisting of EBRs induced from two Kramers pairs of spinful s orbitals and two Kramers pairs of spinful d orbitals at the $1a$ position is identical to the eight bands consisting of two Kramers pairs of spinful s orbitals and two Kramers pairs of spinful d orbitals on the $1b$ position. However, no such statement can be made for four bands induced from just one Kramers pair of s orbitals plus one Kramers pair of d orbitals. Therefore, in $p4m1'$, if four bands are induced from one Kramers pair of spinful s and one Kramers pair of spinful d orbitals at the $1a$ position, and then a gap is closed and reopened such that the C_{4z} eigenvalues of the lower two bands, which we denote as F, are no longer the same at Γ and M (which is always generically admitted because there are no connectivity-4 representation-enforced semimetals[24,56–58,100] in $p4m1'$), neither the two valence nor the two conduction bands will be equivalent to an atomic insulator. This inequivalence holds even though these bands carry the *same* symmetry eigenvalues as bands induced from Kramers pairs of s or d orbitals at the $1b$ position (Supplementary Table 3).

Formally, this can be understood as a failure of the Wannier center homotopy depicted in SFig. 2(c,d) in the presence of $\mathcal{T}$ symmetry. When $\mathcal{T}$ symmetry is absent, four Wannier orbitals may slide from the $1a$ to the $1b$ position along the $4d$ position in a manner that respects the symmetries of type-I magnetic wallpaper group $p4m$. However, when $\mathcal{T}$-symmetry is present, four orbitals are insufficient to satisfy the analogous process in $p4m1'$, as Kramers' theorem mandates that spinful Wannier orbitals be twofold degenerate at each of the four sites of the $4d$ Wyckoff position. As shown in numerous works[1–18], if a set of bands fails to satisfy a Wannier center homotopy because of an insufficient number of occupied Wannier orbitals, then those bands exhibit "fragile" topology. Therefore, the two bands denoted as F in the previous paragraph represent a fragile topological phase in $p4m1'$ that exhibits the same symmetry eigenvalues as a QI obstructed atomic limit in $p4m$ (SEq. (45)), which itself exhibits the same symmetry eigenvalues as an EBR induced from a Kramers pair of spinful d orbitals the $1b$ position of $p4m1'$ (Supplementary Table 3):

$$F \overset{I}{\equiv} [\bar{E}_1 \oplus \bar{E}_2]_{1a} \uparrow G \ominus [\bar{E}_1]_{1b} \uparrow G \overset{I}{\equiv} [\bar{E}_2]_{1b}, \tag{59}$$

where $G = p4m1'$ (unlike previously in SEq. (45)). The symbol $\overset{I}{\equiv}$ in SEq. (59) denotes an "irreducible-representation equivalence" – a weaker form of equivalence than previously employed in SN 3 in which bands simply exhibit the same set of (co)representations[2,3,5,9,17,18]. A more detailed discussion of these contrasting notions of equivalence will appear in SRef. 3.

Crucially, unlike the fragile bands previously analyzed in SRefs. 1, 2, 6–8, 14, 15, and 17, F is *already* irreducible-representation-equivalent to a linear combination of EBRs with only positive coefficients (as opposed to the $\ominus$ in SEq. (59)), which was previously determined in SRef. 2 to be a necessary (but not sufficient) condition for a set of bands to be Wannierizable. We are only able to deduce that F is not Wannierizable by attempting (and failing) to derive a four-band Wannier center homotopy between $1a$ and $1b$ in $p4m1'$ (SEqs. (54) through (58)). F therefore represents a previously unidentified variant of fragile phase that eludes the symmetry-eigenvalue-based diagnosis schemes previously employed in SRefs. 1, 2, 6–8, 14, 15, and 17.

Before analyzing F with tight-binding models, we will demonstrate that when an EBR from the $2c$ position of $p4m1'$, which lies at $(1/2, 0)$, $(0, 1/2)$, is added to F, the resulting set of bands remains irreducible-representation-equivalent to a sum of EBRs with only positive coefficients, as well as (when the two fragile conduction bands $\tilde{F}$ are taken into account) contains the same C_{4z} symmetry eigenvalues as bands that satisfy the eight-band Wannier center homotopy from $1a$ to $1b$ in $p4m1'$ derived in SEqs. (54) through (58). First, we note that SEq. (58) implies that:

$$[\bar{E}_1 \oplus \bar{E}_2]_{1a} \uparrow G \overset{I}{\equiv} [\bar{E}_1 \oplus \bar{E}_2]_{1b} \uparrow G. \tag{60}$$

Therefore, if F characterizes the (fragile) valence bands of a four-band model in $p4m1'$ originating from spinful s and d orbitals at the $1a$ position, then the conduction bands of this model $\tilde{F}$, which are also fragile (SEqs. (54) through (58)), satisfy:

$$\tilde{F} \overset{I}{\equiv} [\bar{E}_1 \oplus \bar{E}_2]_{1a} \uparrow G \ominus F \overset{I}{\equiv} [\bar{E}_1]_{1b}. \tag{61}$$

Next, we form an equivalence between EBRs induced from the $1a$ position of $p4m1'$ and EBRs induced from the $2c$ position. The $2c$ position is invariant under site-symmetry group $mm21'$ and has a unique two-dimensional

corepresentation[19,57] $\bar{E}$ due to the anticommutation relation $\{M_x, M_y\} = 0$. We first examine the $4e$ position at $(x, 0)$, $(0, x)$, which interpolates between $1a$ and $2c$ (Fig. 2(a) of the main text and SRef. 19). The site-symmetry group G_{4e} of the $4e$ Wyckoff position is isomorphic to point group[40] $m1'$, and therefore, like the $4d$ position, has a single corepresentation ${}^1\bar{E}^2\bar{E}$. G_{4e} satisfies:

$$G_{1a} \cap G_{2c} = G_{4e}, \tag{62}$$

where:

$$|G_{1a}|/|G_{4e}| = 4,\ |G_{2c}|/|G_{4e}| = 2, \tag{63}$$

where $|G|$ is the number of elements in G; thus, $|G|/|H| = N$ indicates that $|H|$ is an index-N subgroup of G. We also compute:

$$({}^1\bar{E}^2\bar{E})_{4e} \uparrow G_{1a} = (2\bar{E}_1)_{1a} \oplus (2\bar{E}_2)_{1a},\ ({}^1\bar{E}^2\bar{E})_{4e} \uparrow G_{2c} = (2\bar{E})_{2c}, \tag{64}$$

which implies that:

$$[2\bar{E}_1 \oplus 2\bar{E}_2]_{1a} \uparrow G \equiv [2\bar{E}]_{2c} \uparrow G. \tag{65}$$

SEq. (65) displays a similar issue as in SEq. (45): we are unable to define an equivalence between bands induced from $2c$ and single copies of bands induced from the $1a$ position. However, we can still use SEq. (65) to define an irreducible-representation equivalence:

$$[\bar{E}_1 \oplus \bar{E}_2]_{1a} \uparrow G \overset{I}{\equiv} [\bar{E}]_{2c} \uparrow G. \tag{66}$$

We then combine SEqs. (59), (60), (61), and (66) to deduce that:

$$F \oplus \tilde{F} \oplus [\bar{E}]_{2c} \uparrow G \overset{I}{\equiv} [2\bar{E}_1 \oplus 2\bar{E}_2]_{1b} \uparrow G. \tag{67}$$

To conclude, SEq. (67) implies that the fragile valence and conduction bands F and $\tilde{F}$, which originated from spinful s and d orbitals at the $1a$ position of $p4m1'$, along with bands induced from spinful s orbitals at the $2c$ position of $p4m1'$, exhibit the same symmetry eigenvalues as eight bands induced from the $1b$ position. As shown in SEq. (58), this implies that if those eight bands could also be formed into maximally localized, symmetric Wannier functions at $1b$, then they could also be freely "slid" to $1a$ while respecting the symmetries of $p4m1'$.

Though SEq. (67) does not on its own additionally imply that $F \oplus [\bar{E}]_{2c} \uparrow G$ is Wannierizable, we will also numerically demonstrate in this section that a tight-binding model in $p4m1'$ with the occupied bands $F \oplus [\bar{E}]_{2c} \uparrow G$ exhibits trivial winding in all symmetry-inequivalent Wilson loops. Here, we define symmetry-inequivalent Wilson loops as sets of Wilson loops that that are not related to each other by bulk crystal symmetries, and where in this section, for convenience, we only sample one Wilson loop (the $(2x + y)$-directed Wilson loop) that does not preserve *any* edge-projecting crystal symmetry[27] (aside from perpendicular lattice translations).

To numerically demonstrate the removal of Wilson loop winding, which we here refer to as the "trivialization" of the Wilson loop, we begin by reproducing the tight-binding model that realizes the two sets of bands with fragile topology shown in Fig. 2(i-k) of the main text:

$$\begin{aligned}\mathcal{H}_F(\mathbf{k}) &= t_1\tau^z[\cos(k_x) + \cos(k_y)] + t_2\tau^x[\cos(k_x) - \cos(k_y)] + v_m\tau^z \\ &\quad + t_{PH}\mathbb{1}_{\tau\sigma}[\cos(k_x) + \cos(k_y)] + v_s\tau^y\sigma^z\sin(k_x)\sin(k_y) \\ &\quad + v_{M_z}\left[\tau^z\sigma^y\sin(k_x) - \tau^z\sigma^x\sin(k_y)\right].\end{aligned} \tag{68}$$

$\mathcal{H}_F(\mathbf{k})$ is invariant under wallpaper group $p4m1'$. To tune SEq. (68) into a fragile phase with the occupied bands F (SEq. (59)), we choose the parameters $t_1 = 5$, $t_2 = 1.5$, $v_m = -1.5$, $t_{PH} = 0.1, v_s = 1.3$, and $v_{M_z} = 0.4$. In its fragile phase, the spectrum of $\mathcal{H}_F(\mathbf{k})$ exhibits two sets of bands separated by an energy gap at half filling. When placed on a ribbon geometry, the fragile phase of SEq. (68) exhibits no edge modes (Fig. 2(j) of the main text), but when placed on a finite-sized square, it exhibits four Kramers pairs of corner modes (SN 9) that, depending on the energetics (here, the value of t_2), can "float" into the gap, as seen at $\bar{\Gamma}$ in Fig. 3(h) of the main text. As shown in SN 9, even when the corner modes lie within the bulk manifolds, the fragile phase can still be distinguished from a trivial insulator by counting the number of states above and below the gap in the energy spectrum calculated with square (open) boundary conditions.

The x-directed Wilson loop over the lower two bands (or alternatively over the upper two bands) of the fragile phase of SEq. (68) exhibits the same winding as would a TCI with mirror Chern number $C_{M_z} = 2$, even though M_z is

broken when $v_{M_z} \neq 0$ (conversely, when v_{M_z} is tuned to zero without closing a bulk gap, an edge gap closes and the lower two bands actually do characterize a $C_{M_z} = 2$ TCI (Fig. 2(f-h) of the main text)). Unlike in a 2D TCI phase, the x-directed Wilson loop over the lower two bands of the fragile phase of SEq. (68) (Fig. 2(k) of the main text) exhibits crossings that are not protected by the corepresentations of the (10)-edge line group[20,23,27] $pm1'$. Instead, the Wilson crossings in Fig. 2(k) of the main text are only protected by *bulk* symmetries (specifically $C_{2z} \times \mathcal{T}$); more specific details regarding $C_{2z} \times \mathcal{T}$-protected Wilson crossings are discussed in SRefs. 5 and 6. In this work, we demonstrate, through the addition of spinful s orbitals at the $2c$ position, that the winding Wilson spectrum of the valence bands of SEq. (68) becomes trivialized, a hallmark of "fragile," topology[1–18]. We choose to place the additional orbitals at the $2c$ position, rather than at the $1a$ position, which we have already demonstrated can relieve the obstruction to forming an obstructed atomic limit (SEq. (58)), because the particular couplings that trivialize the set of Wilson spectra in this model are considerably easier to deduce using bands from $2c$, and because bands from $2c$ can be irreducible-representation-equivalent to bands from $1a$ (SEq. (66)).

To demonstrate how the winding of the Wilson spectrum of the lower two bands of SEq. (68) is fragile, we introduce spinful s orbitals at the $2c$ position (SFig. 4) through the addition of the terms:

$$V_C(\mathbf{k}) = v_\mu P_{\mu^s} + v_C \left[\mu_1^x \cos\left(\frac{k_x}{2}\right) + \mu_2^x \cos\left(\frac{k_y}{2}\right)\right] + v_{CS}\left[\mu_1^x \sigma^y \sin\left(\frac{k_x}{2}\right) + \mu_2^x \sigma^x \sin\left(\frac{k_y}{2}\right)\right], \quad (69)$$

where μ_1^x (μ_2^x) represents sublattice hopping between the $d_{x^2-y^2}$ orbitals at the $1a$ position and the s orbitals at the $x = 1/2,\ y = 0$ ($x = 0,\ y = 1/2$) site of the $2c$ position, and where P_{μ^s} is a projection matrix into the $2c$ s-orbital subspace of the eight-band Hamiltonian $\mathcal{H}_F(\mathbf{k}) + V_C(\mathbf{k})$. As shown in SFig. 4, v_C (v_{CS}) represents (spin-) orbital coupling between Kramers pairs of spinful s orbitals at the $2c$ position and spinful $d_{x^2-y^2}$ orbitals at the $1a$ position, and v_μ represents a chemical potential on the $2c$ s orbitals in $\mathcal{H}_F(\mathbf{k}) + V_C(\mathbf{k})$ (SEqs. (68) and (69)); there is no hopping between the spinful s orbitals at $1a$ and the s orbitals at $2c$. We then calculate the bulk band structure and x-, $(x + y)$-, and $(2x + y)$-directed Wilson loops over the lower six bands of the eight-band Hamiltonian using the parameters $v_\mu = 8.25 v_m$; $v_C = 0, 4$, and 8; and $v_{CS} = 0.45 v_C$ (SFig. 3). As the bulk BZ (Fig. 2(b) of the main text) has independent mirror lines along $k_{x,y} = 0, \pi$ and $k_x = \pm k_y$, these three Wilson loops comprise the set of symmetry-inequivalent Wilson loops that pass through the Γ point. Aside from the $(2x + y)$-directed Wilson loop, there are of course other, low-symmetry Wilson loops, but they will exhibit spectra adiabatically related to that of the $(2x+y)$-directed loop. As can be observed in SFig. 3(f,i,l), all possible Wilson spectra over the lower six bands of this model (P_6 in SFig. 3(a-c)) become trivialized when the two fragile bands are coupled to four bands from spinful s orbitals at the $2c$ position, *even though the bands from these s orbitals are separated from the bands with fragile topology by an energy gap* (which is explicitly shown along high-symmetry lines in SFig. 3(b,c), and was additionally numerically confirmed throughout the BZ interior). However, if the Wilson projector for the same system is chosen over just the two fragile valence bands (P_2 in SFig. 3(c)), then the Wilson loop still winds (SFig. 5), *despite the presence of the additional bands.* This is further explored in SRefs. 5 and 17.

5. Alternative Realizations of $\mathcal{T}$-Symmetric HOFA Semimetals

In the main text, we presented two realizations of $\mathcal{T}$-symmetric Dirac semimetals with HOFAs: in Fig. 3(d,g) of the main text, we showed a tetragonal Dirac semimetal characterized by a nontrivial plane with mirror Chern number $C_{M_z} = 2$ at $k_z = 0$, and in Fig. 3(e,h) of the main text, we showed a tetragonal Dirac semimetal with broken M_z and $\mathcal{I}$ symmetries, for which the $k_z = 0$ plane is in the fragile topological phase examined in SN 4. Here, we present two additional realizations of $\mathcal{T}$-symmetric Dirac semimetals with HOFA states. There are likely many more semimetallic systems with other variants of HOFAs, including both those with $4mm$-symmetric Dirac points representative of the quantum critical point of the QI phase[26], and also additional HOFA semimetals with nodal features equivalent to other 2D atomic limit or fragile transitions[11,39,78]. For simplicity, we will focus in this section on HOFA semimetals within the same space group (SG 123 $P4/mmm1'$) that vary by the locations and multiplicities of their Dirac points and by their bulk symmetry (parity) eigenvalues, and leave for future works the enumeration of all possible 2D one-parameter atomic-limit and fragile-phase transitions (SN 4), and their corresponding 3D HOFA semimetals.

HOFA Semimetals with Band Inversion on a Line – One of the simplest realizations of a $\mathcal{T}$-symmetric Dirac semimetal for which the only boundary modes are hinge-localized Fermi arcs occurs when there are multiple inversions between bands with distinct C_{4z} eigenvalues along $4mm$-invariant lines in a HOFA-supporting SG (SN 12). Though we do not present material candidates in this work with this band inversion structure, we do formulate in this section tight-binding models with multiple band inversions, and show that they exhibit HOFAs as their only boundary (surface and hinge) modes.

To begin, consider a Dirac semimetal in SG 123 ($P4/mmm1'$) formed from orbitals at the $1a$ Wyckoff position ($\{x, y, z\} = \{0, 0, 0\}$) that exhibits four total Dirac points along the $k_x = k_y = 0$ line (SFig. 6(a)). Each one of

these Dirac points is formed by an inversion of the irreducible corepresentations $\bar{\rho}_{6,7}$ of the little group of this BZ line (SN 3). The Hamiltonian of each 2D plane of the BZ indexed by k_z ($\mathcal{H}(k_z)$) is therefore characterized by a $\mathbb{Z}_2$ quantity dictating whether it is equivalent to a 2D trivial insulator or to a QI with corner modes. This $\mathbb{Z}_2$ number is determined at each k_z by the C_{4z} eigenvalues of the occupied corepresentations along the two BZ lines with $4mm$ symmetry: ΓZ ($k_x = k_y = 0$) and MA ($k_x = k_y = \pi$). Specifically, using the results of SN 3, along ΓZ and MA, the occupied bands at a fixed value of k_z exhibit the following set of corepresentations:

$$\{\bar{\rho}\}_{0,\pi}(k_z) = a(k_z)_{0,\pi}\bar{\rho}_7 \oplus b(k_z)_{0,\pi}\bar{\rho}_6, \tag{70}$$

where $a(k_z)_{0,\pi}$ and $b(k_z)_{0,\pi}$ are integers indicating the multiplicities of corepresentations $\bar{\rho}_{7,6}$ in the set $\{\bar{\rho}\}$ and where:

$$a_{0,\pi}(k_z) + b_{0,\pi}(k_z) = \nu/2, \tag{71}$$

where $\nu/2$ is equal to half the number of occupied bands, as $\dim\bar{\rho}_6 = \dim\bar{\rho}_7 = 2$, and where $0,\pi$ indicate BZ lines at $k_x = k_y = 0,\pi$, respectively. When $a_0(k_z) = a_\pi(k_z)$ (and hence $b_0(k_z) = b_\pi(k_z)$), Supplementary Table 3 implies that the symmetry eigenvalues of this BZ plane indexed by k_z match those of a 2D spinful magnetic atomic insulator with $p4m$ symmetry and $\nu/2$ pairs of spinful orbitals at the $1a$ position (though again here, as in SN 3, the spinful orbitals are paired by $M_{x,y}$, and not by $\mathcal{T}$). Conversely if $a_0(k_z) \neq a_\pi(k_z)$ (and hence $b_0(k_z) \neq b_\pi(k_z)$), the symmetry eigenvalues of the bands in the BZ plane at k_z match those of a 2D spinful magnetic atomic insulator with $\nu/2 - |a_0(k_z) - a_\pi(k_z)|$ pairs of spinful atomic orbitals at $1a$ and $|a_0(k_z) - a_\pi(k_z)|$ pairs at $1b$.

Continuing, we consider advancing k_z until we encounter a gapless (Dirac) point along either ΓZ or MA. A Dirac point along ΓZ changes $a_0(k_z)$ and $b_0(k_z)$ by one; similarly a Dirac point along MA changes $a_\pi(k_z)$ and $b_\pi(k_z)$ by one. Thus, the symmetry eigenvalues of the occupied bands in the 2D planes above or below a Dirac point are the same as those of 2D atomic insulators which differ by one in their number of Wannier centers at the $1a$ position. Consequently, if there is a Dirac point along ΓZ that lies above k_{z1} and below k_{z2}, then:

$$a_0(k_{z2}) = a_0(k_{z1}) \pm 1,\ b_0(k_{z2}) = b_0(k_{z1}) \mp 1,\ a_\pi(k_{z2}) = a_\pi(k_{z1}),\ b_\pi(k_{z2}) = b_\pi(k_{z1}), \tag{72}$$

and thus $\mathcal{H}(k_{z2})$ is equivalent to a 2D insulator with one fewer pair of Wannier orbitals at $1a$ and one more pair at $1b$. Because the values of $a_{0,\pi}(k_z)$ and $b_{0,\pi}(k_z)$ are not independent (SEq. (71)), we can therefore restrict our discussion to changes in $a_{0,\pi}(k_z)$, which combined with SEq. (71), is sufficient information to determine the (polarization) topology of k_z-indexed planes in this semimetal. If we now assume that $a_0(k_{z1}) = a_\pi(k_{z1})$, then $\mathcal{H}(k_{z2})$ will therefore be nontrivial and exhibit QI corner states. If there is then a second Dirac point along ΓZ lying above k_{z2} and below k_{z3}, then:

$$a_0(k_{z3}) = a_0(k_{z2}) \pm 1,\ a_\pi(k_{z3}) = a_\pi(k_{z2}), \tag{73}$$

leading to an overall relation:

$$a_0(k_{z3}) = a_0(k_{z1}) \text{ or } a_0(k_{z3}) = a_0(k_{z1}) \pm 2. \tag{74}$$

Therefore, $\mathcal{H}(k_{z3})$ is equivalent to a 2D insulator with *either* the same number of Wannier orbitals at $1a$ ($a_0(k_{z3}) = a_0(k_{z1}) = a_\pi(k_{z3})$) as $\mathcal{H}(k_{z1})$, or to one with two pairs of Wannier orbitals at $1b$ ($a_0(k_{z3}) = a_0(k_{z1}) \pm 2 = a_\pi(k_{z3}) \pm 2$). However, keeping only the symmetries of magnetic wallpaper group $p4m$ or its layer supergroup $p4/m'mm$ (SN 3), a set of bands containing only trivial bands and two copies of the QI exhibits a net-trivial quadrupole moment[28] of e mod $e = 0$. Therefore, both of the possibilities in SEq. (74) imply the absence of protected QI corner states at k_{z3}. We can realize this semimetal by tuning parameters to place two, time-reversed pairs of QI-nontrivial regions at k_z values away from the TRIM points (SFig. 6(b)). If the symmetry indicators at the $k_z = 0,\pi$ planes are otherwise trivial (*e.g.* the symmetry eigenvalues do not indicate 2D quantum spin Hall or mirror Chern $C_{M_z} = 2$ phases[32,38,80,101]), and if all the non-symmetry-indicated Wilson loops are trivial (ruling out the case of $C_{M_z} = 4n$ where n is a nonzero integer[33]), then this semimetal will not exhibit any topological surface states on its 2D faces[32], and will only exhibit HOFAs connecting the hinge projections of its four bulk 3D Dirac points. We realize a Dirac semimetal in this phase (SFig. 6(a,b)) by introducing the term:

$$V_{H2}(\mathbf{k}) = t_{H2}\cos(2k_z), \tag{75}$$

to $\mathcal{H}_{H2}(\mathbf{k})$ in SEq. (6), and choosing the parameters:

$$t_1 = 2,\ t_2 = 1.5,\ v_m = -1.5,\ t_{PH} = 0.1,\ v_s = 1.3,\ v_{M_z} = 0,\ t_H = 1.6,\ u(\mathcal{H}_{H2}) = 0.5, t_{H2} = -4.5. \tag{76}$$

A similar configuration of HOFAs can also be achieved in a Dirac semimetal in SG 123 ($P4/mmm1'$) that is formed from orbitals at the $1a$ Wyckoff position and exhibits a time-reversed pair of Dirac points along $k_{x,y} = 0$ and a

time-reversed pair along $k_{x,y} = \pi$ (SFig. 6(c)). To explain this, we follow the same logic as previously and place a Dirac point along ΓZ at a value of k_z between $k_{z1,2}$ where $k_{z2} > k_{z1} > 0$. The Dirac point between k_{z1} and k_{z2} has a time-reversal partner with $k_z < 0$, to which the following arguments also apply. At k_{z2},

$$a_0(k_{z2}) = a_0(k_{z1}) \pm 1,\ a_\pi(k_{z2}) = a_\pi(k_{z1}), \tag{77}$$

again indicating that $\mathcal{H}(k_{z2})$ is equivalent to a 2D insulator with one fewer pair of Wannier orbitals at $1a$ and one pair at $1b$, and specifically is equivalent to a QI if $a_0(k_{z1}) = a_\pi(k_{z1})$. We then place a second Dirac crossing instead *along* MA ($k_x = k_y = \pi$) above k_{z2} and below a third momentum k_{z3} for which $k_{z3} > k_{z2}$, such that:

$$a_0(k_{z3}) = a_0(k_{z2}),\ a_\pi(k_{z3}) = a_\pi(k_{z2}) \pm 1, \tag{78}$$

which indicates that $\mathcal{H}(k_{z3})$ is equivalent to a 2D insulator with *either* the same number of Wannier orbitals at $1a$ ($a_0(k_{z3}) = a_\pi(k_{z3})$) as $\mathcal{H}(k_{z1})$, or to one with two pairs of Wannier orbitals at $1b$ ($a_0(k_{z3}) = a_\pi(k_{z3}) \pm 2$), both of which correspondingly carry a trivial quadrupole moment of $e \mod e = 0$ and do not exhibit corner states. The Dirac point between k_{z2} and k_{z3} also has a time-reversal partner with $k_z < 0$, to which these arguments also apply.

We realize a semimetal in SG 123 $P4/mmm1'$ with this configuration of Dirac points (SFig. 6(c)) by choosing in SEqs. (6) and (75) the parameters:

$$t_1 = 2,\ t_2 = 1.5,\ v_m = -1.5,\ t_{PH} = 0.1,\ v_s = 1.3,\ v_{M_z} = 0,\ t_H = 6.4,\ u(\mathcal{H}_{H2}) = 0.5, t_{H2} = 0. \tag{79}$$

Plotting the bands of SEqs. (6) and (75) with the parameters in SEq. (79) on a z-directed rod (SFig. 6(d)), it is clear that HOFA states connect the projections of the bulk Dirac points along ΓZ to those along MA, and that there are no other boundary modes. Specifically, at $k_z = 0, \pi$, the C_{4z} eigenvalues of the occupied bands are the same at $k_x = k_y = 0, \pi$, and match those of either a trivial insulator or a TCI with[33] $C_{M_z} \mod 4 = 0$. Through calculations of the x-directed Wilson loop and the (100)-surface states (the results of which are implicit in the rod bands shown in SFig. 6(d)), we confirm that the Hamiltonians of the $k_z = 0, \pi$ planes are topologically trivial ($C_{M_z} = 0$).

Finally, for completeness, we note that depending on the values of $a_{0,\pi}(k_z)$ and $b_{0,\pi}(k_z)$, sets of four occupied bands can exhibit the same symmetry eigenvalues as *either* superposed atomic limits at the $1a$ and $1b$ position of $p4m$, or a single, four-band atomic limit at the $2c$ position (SEqs. (60) and (66)). Specifically, because placing pairs of spinful s orbitals at the $2c$ position of $p4m$ (Fig. 2(a) of the main text) induces four bands with the combined symmetry eigenvalues of bands from s orbitals at the $1a$ and $1b$ positions (SN 3 and SRefs. 24, 57–59), then, for generic numbers of occupied bands with different complex-conjugate pairs of C_{4z} eigenvalues, a change of $a_{0,\pi}(k_z)$ indicates *either* a shift of two occupied (and two unoccupied) Wannier orbitals from $1a$ to $1b$ or a shift of four occupied (and four unoccupied) Wannier orbitals from $1a$ and $1b$ to $2c$. We emphasize this point because the irreducible-representation equivalence between EBRs from $1a$ plus $1b$ and EBRs from $2c$ in $p4m$ is key to understanding atomic-limit transitions in *nonsymmorphic* 2D wallpaper groups[20,27], such as $p4g$, which, while largely beyond the scope of this work, can also exhibit corner modes[102] that are equivalent to the HOFA states of $4mm$-symmetric *nonsymmorphic* 3D Dirac semimetals (SN 12).

$p_z - d_{x^2-y^2}$-Hybridized HOFA Dirac Semimetals – We can also realize a $\mathcal{T}$-symmetric semimetal with HOFA states in SG 123 $P4/mmm1'$ by substituting the s orbitals used to form $\mathcal{H}_{H2}(\mathbf{k})$ in SEq. (6) with p_z orbitals, still at the $1a$ position. We first discuss how the representations of crystalline symmetries are different under this orbital substitution than in the $s-d$-hybridized cases highlighted in the main text, and then demonstrate how this difference affects the topology in k_z-indexed BZ planes.

The site-symmetry group of the $1a$ position of SG 123 is $4/mmm1'$ (D_{4h}), which is an index-2 supergroup of $4mm1'$:

$$4/mmm1' = (E)4mm1' \cup (\mathcal{I})4mm1'. \tag{80}$$

As $\mathcal{I}h = h\mathcal{I}$ for all $h \in 4mm1'$, then there are simply twice as many corepresentations of $4/mmm1'$ as there are of $4mm1'$, each of which takes the form $\bar{E}_{1,2/g,u}$ where g (u) indicates a two-dimensional corepresentation with positive (negative) inversion eigenvalues[19,40]. Specifically, the p_z orbitals transform as $\bar{E}_{1,u}$ and the $d_{x^2-y^2}$ orbitals transform as $\bar{E}_{2,g}$. When these corepresentations are induced into the space group $G = P4/mmm1'$ and then subduced onto the TRIM points at $k_x = k_y = 0, \pi$ (SFig. 7(a)), the resulting little co-group corepresentations are[24,57–59]:

$$\begin{aligned}(\bar{E}_{1u} \uparrow G) \downarrow \Gamma \equiv (\bar{E}_{1u} \uparrow G) \downarrow Z \equiv (\bar{E}_{1u} \uparrow G) \downarrow M \equiv (\bar{E}_{1u} \uparrow G) \downarrow A \equiv \bar{\rho}_7^-,\\ (\bar{E}_{2g} \uparrow G) \downarrow \Gamma \equiv (\bar{E}_{2g} \uparrow G) \downarrow Z \equiv (\bar{E}_{2g} \uparrow G) \downarrow M \equiv (\bar{E}_{2g} \uparrow G) \downarrow A \equiv \bar{\rho}_6^+,\end{aligned} \tag{81}$$

where placing $\mathcal{I}$-odd ($\bar{E}_{1,2u}$) (-even ($\bar{E}_{1,2g}$)) orbitals at $1a$ results in all little co-group corepresentations having negative (positive) inversion eigenvalues, whose sum is given by the characters:

$$\chi_{\bar{\rho}^+_{6,7}}(\mathcal{I}) = +2,\ \chi_{\bar{\rho}^-_{6,7}}(\mathcal{I}) = -2. \tag{82}$$

The little co-group corepresentations $\bar{\rho}^{\pm}_{6,7}$ also inherit the C_{4z} characters of the corepresentations $\bar{\rho}_{6,7}$ of their $\mathcal{I}$-broken subgroups $4mm$ and $4/m'mm$ (SEq. (33)):

$$\chi_{\bar{\rho}^{\pm}_{6}}(C_{4z}) = -\sqrt{2},\ \chi_{\bar{\rho}^{\pm}_{7}}(C_{4z}) = \sqrt{2}. \tag{83}$$

Crucially, BZ planes indexed by $k_z \neq 0, \pi$ are only invariant under magnetic layer group $p4/m'mm$, and thus, as discussed in SN 3 and 5, their Hamiltonians are equivalent to 2D trivial insulators or QIs as indicated only by the number of occupied bands labeled by $\bar{\rho}_6$ at $k_x = k_y = 0, \pi$ (discussed in the text following SEq. (74)). Therefore, whether all of the valence bands of a Dirac semimetal in SG 123 $P4/mmm1'$ have the same inversion eigenvalues at the C_{4z}-invariant TRIM points ($k_x = k_y = 0, \pi$, $k_z = 0, \pi$), or if some of the inversion eigenvalues are different, the semimetal will still exhibit HOFA states, because the (obstructed-atomic-limit, specifically QI) topology of the k_z-indexed planes away from the TRIM points is unaffected by the topology in high-symmetry planes elsewhere in the BZ. Consequently, a Dirac semimetal in SG 123 $P4/mmm1'$ should exhibit HOFA states whether it is formed from hybridized s and $d_{x^2-y^2}$ orbitals or from p_z and $d_{x^2-y^2}$ orbitals.

To demonstrate this, we form a tight-binding model with p_z and $d_{x^2-y^2}$ orbitals placed at the $1a$ position of SG 123 $P4/mmm1'$:

$$\begin{aligned}\mathcal{H}(\mathbf{k}) &= \left[t_{z1}\cos(k_z) + t_{z2}\cos(k_z)\times(\cos(k_x)+\cos(k_y))\right]\tau^z + t_{xy}\left[\cos(k_x)+\cos(k_y)\right]\tau^z \\ &+ t_{PH}\cos(k_z)\mathbb{1}_{\tau\sigma} + v_s\left[\sin(k_x)\tau^x\sigma^y + \sin(k_y)\tau^x\sigma^x\right] + v_{Q1}\tau^y\sin(k_z)\left[\cos(k_x)-\cos(k_y)\right] \\ &+ v_{Q2}\tau^x\sigma^z\sin(k_x)\sin(k_y)\sin(k_z),\end{aligned} \tag{84}$$

where τ indicates the orbital degree of freedom, σ indicates the $\mathcal{T}$-odd spin degree of freedom, and where $\mathbb{1}_{\tau\sigma}$ is the 4×4 identity. We note that after the submission of this work, it was recognized that SEq. (84) contains the same terms as the simplified Dirac semimetal model employed in SRef. 103 (as well as additional terms that respect the symmetries of SG 123 $P4/mmm1'$). In SEq. (84), all of the terms correspond to nearest-neighbor hopping, except for the quadrupolar SOC terms v_{Q1} and v_{Q2}, which correspond to second-neighbor hopping in the xy-plane and third-neighbor hopping along the xyz diagonal, respectively (SFig. 7(b)). These terms, like $U(\mathbf{k})\sin(k_z)$ in SEqs. (4) and (6) for the $s-d$-hybridized Dirac semimetal, are equivalent to the term employed in SRef. 34 to disconnect the surface Fermi arcs of Dirac semimetals. Specifically, $v_{Q1,2}$ enforce the breaking of $\mathcal{T}$- and M_z symmetries in k_z-indexed BZ planes away from $k_z = 0, \pi$, and gap the surface states at generic values of $k_z \neq 0, \pi$. When $v_{Q1,2}$ in SEq. (84) (and u in SEqs. (4) and (6)) are strong, this causes the surface states to close off into rings with a narrow width in k_z, as opposed to connecting all the way to the surface projections of the bulk Dirac points (SFig. 8(c,g)). This is allowed because unlike in Weyl semimetals, the surface Fermi arcs in Dirac semimetals are not protected by a robust bulk topological invariant, and are rather just a consequence of the continuity of surface states from topological surface TI and TCI cones to trivial surface states in the vicinities of the projections of bulk Dirac points that can be pushed away from the Fermi energy by bulk SOC[34–37]. This causes the surface states of Dirac semimetals to either appear in closed loops disconnected from the projections of the bulk Dirac points (SFig. 8(c,g) and SRefs. 34, 35, and 37), or allows them to be completely absent, depending on the topology in high-symmetry BZ planes[32,36].

The generating symmetries of the $p-d$-hybridized model in SEq. (84) are represented at the eight TRIM points by:

$$\mathcal{T} = i\sigma^y K,\ \mathcal{I} = \tau^z,\ M_{x,y} = -i\sigma^{x,y}, \tag{85}$$

and by an additional C_{4z} symmetry at the four C_{4z}-invariant TRIM points:

$$C_{4z} = \tau^z\left(\frac{\mathbb{1} - i\sigma^z}{\sqrt{2}}\right), \tag{86}$$

where the prefactor of τ^z in SEq. (86) reflects that p_z ($d_{x^2-y^2}$) orbitals are even (odd) under C_{4z}. SEqs. (85) and (86) also imply the presence of C_{2z} and M_z symmetries, which are represented at each TRIM point by:

$$C_{2z} = (C_{4z})^2 = -i\sigma^z,\ M_z = \mathcal{I}C_{2z} = -i\tau^z\sigma^z. \tag{87}$$

To form a semimetal with broken particle-hole symmetry and Dirac points along ΓZ, we choose in SEq. (84) the parameters:

$$t_{z1} = 0.9,\ t_{z2} = 0.9,\ t_{xy} = 1,\ t_{PH} = 0.1,\ v_s = 0.8,\ v_{Q1} = 0.6,\ v_{Q2} = 0.25. \tag{88}$$

In SFig. 8, we plot the bulk bands, x-directed Wilson bands at $k_z = 0$, (100)-surface states, and z-directed rod bands of (a-d) the $s-d$-hybridized Dirac semimetal highlighted in the main text (SEq. (6)) and of (e-h) the $p-d$-hybridized

Dirac semimetal described by SEq. (84). Along the four C_{2z}-invariant lines of the BZ at $k_{x,y}=0,\pi$, SEq. (84) takes the simplified form:

$$\begin{aligned}\mathcal{H}(0,0,k_z) &= [(t_{z1}+2t_{z2})\cos(k_z)+2t_{xy}]\tau_z + a\cos(k_z)\mathbb{1}_{\tau\sigma},\\ \mathcal{H}(\pi,\pi,k_z) &= [(t_{z1}-2t_{z2})\cos(k_z)-2t_{xy}]\tau_z + a\cos(k_z)\mathbb{1}_{\tau\sigma},\\ \mathcal{H}(0,\pi,k_z) &= \mathcal{H}(\pi,0,k_z) = t_{z1}\cos(k_z)\tau^z + a\cos(k_z)\mathbb{1}_{\tau\sigma},\end{aligned} \tag{89}$$

which, as,

$$[\tau^z,\mathcal{H}(0,0,k_z)] = [\tau^z,\mathcal{H}(\pi,\pi,k_z)] = [\tau^z,\mathcal{H}(0,\pi,k_z)] = 0, \tag{90}$$

indicates that the inversion and C_{4z} eigenvalues of the occupied states at the TRIM points at half filling in SEq. (84) in its Dirac semimetallic phase are entirely determined by the signs of the prefactors of τ^z in SEq. (89). Defining $\bar{\sigma}(\mathbf{k})$ as the corepresentation of the two occupied bands at $\mathbf{k}$, we find that:

$$\bar{\sigma}(\Gamma)=\bar{\rho}_7^-,\ \bar{\sigma}(Z)=\bar{\sigma}(M)=\bar{\sigma}(A)=\bar{\rho}_6^+,\ \bar{\sigma}(X)=\bar{\sigma}(X')=\bar{\varrho}_5^-,\ \bar{\sigma}(R)=\bar{\sigma}(R')=\bar{\varrho}_5^+, \tag{91}$$

where the C_{4z}-symmetric points Γ, Z, M, and A have little co-groups isomorphic to $4/mmm1'$ and therefore the corepresentations in SEq. (81). Conversely, the X and R (and X' and R') points are not C_{4z} symmetric, and therefore they have little co-groups isomorphic to $mmm1'$, which has two, two-dimensional corepresentations[19,40,57–59]:

$$(\bar{E}_{1,2/g,u}\uparrow G)\downarrow X \equiv (\bar{E}_{1,2/g,u}\uparrow G)\downarrow R \equiv \bar{\varrho}_5^{+,-}, \tag{92}$$

with inversion characters:

$$\chi_{\bar{\varrho}_5^{\pm}}(\mathcal{I}) = \pm 2. \tag{93}$$

We observe that the only TRIM points with negative inversion eigenvalues are Γ, X, and X', which all lie in the $k_z=0$ plane. Using the results of SRefs. 33 and 60, we can express the mirror Chern numbers $C_{M_z}(0,\pi)$ of the Hamiltonians of the $k_z=0,\pi$ planes in terms of the occupied corepresentations:

$$\begin{aligned}C_{M_z}(k_z=0,\pi)\text{ mod }4 = \Bigg(&\left[n_{0,0,k_z}(\bar{\rho}_7^-)+n_{\pi,\pi,k_z}(\bar{\rho}_7^-)\right] + 2\left[n_{0,0,k_z}(\bar{\rho}_6^+)+n_{\pi,\pi,k_z}(\bar{\rho}_6^+)\right]\\ &+3\left[n_{0,0,k_z}(\bar{\rho}_6^-)+n_{\pi,\pi,k_z}(\bar{\rho}_6^-)\right] + 2n_{\pi,0,k_z}(\bar{\varrho}_5^-)\Bigg)\text{ mod }4,\end{aligned} \tag{94}$$

where $n_{k_x,k_y,k_z}(\bar{\sigma})$ is equal to the number of copies of the corepresentation $\bar{\sigma}$ that appear in the valence manifold[33,49,60–62] at $\mathbf{k}=(k_x,k_y,k_z)$, where we restrict $k_z=0,\pi$, and where C_{M_z} mod 2 is equivalent to the Fu-Kane parity index z_2 for a 2D TI[38]. For the $p-d$-hybridized semimetal described by SEq. (84) with the parameters in SEq. (88), SEq. (91) implies the values of $n_{k_x,k_y,k_z}(\bar{\sigma})$ shown in Supplementary Table 4. As shown in Supplementary Table 4, for the $p-d$-hybridized HOFA Dirac semimetal in SEq. (84), the Hamiltonian of the $k_z=0$ plane is equivalent to a 2D TI, whereas the Hamiltonian of the $k_z=\pi$ plane exhibits the same symmetry eigenvalues as a trivial insulator[33,49,60–62]. This is the same high-symmetry-plane topology that occurs in many previously identified centrosymmetric 3D Dirac semimetals[32,34], such as Na_3Bi[104–106] and two of the candidate HOFA-semimetals highlighted in this work (Fig. 4 of the main text and SN 13): KMgBi[107–109] and Cd_3As_2[110,111]. In SFig. 8(f,g), we show the x-directed Wilson loop at $k_z=0$ and the (100)-surface states of SEq. (84), respectively. The Wilson loop exhibits the characteristic winding of a 2D TI, and the surface spectrum correspondingly consists of a single twofold-degenerate cone at $k_z=0$, $k_y=\pi$.

Conversely, in the $s-d$-hybridized HOFA Dirac semimetal highlighted in the main text (SEq. (6)), neither of the high-symmetry BZ planes indexed by k_z is topologically equivalent to a 2D TI. Instead, as shown in Supplementary Table 5, in the $s-d$-hybridized HOFA Dirac semimetal in SEq. (6) with the parameters in Supplementary Table 2, the Hamiltonian of the $k_z=0$ plane is equivalent to a 2D TCI[32] (C_{M_z} mod $4=2$). This occurs because, unlike in the $p-d$-hybridized semimetal (SEq. (91)), all of the bulk corepresentations $\bar{\sigma}$ in the $s-d$-hybridized semimetal have positive inversion characters $\chi_{\bar{\sigma}}(\mathcal{I})$. In the surface states of both the $s-d$-hybridized semimetal (SFig. 8(c)) and SEq. (6)) and the $p-d$-hybridized semimetal (SFig. 8(g) and SEq. (84)), only the gapped remnants of surface Fermi arcs (white arrows) are visible connecting the TI cones at $k_z=0$ (TCI cones of the $s-d$-hybridized model in (c)) to the projections of the bulk Dirac points (red arrows). In both models, bulk quadrupolar SOC ($U(\mathbf{k})\sin(k_z)$ in SEq. (6) and the $v_{Q1,2}$ terms in SEq. (84)) has gapped the surface Fermi arc states and pushed them away from the Fermi energy, as described in SRefs. 34–37. Calculating the bands of a z-directed rod of the $p-d$-hybridized semimetal described by SEq. (84) (SFig. 8(h)), we observe four HOFA states connecting the projections of the bulk 3D Dirac points to those of the 2D face cones at $k_z=0$, the same number observed in the rod bands of the $s-d$-hybridized semimetal (SFig. 8(d)).

6. Summary of Supplementary Notes Detailing the Evolution of the 1D Edge States of 2D TIs to the Corner Modes of QIs and Fragile Topological Phases

In the next supplementary notes, we will use low-energy field theory to track the evolution of the 1D edge modes of a 2D TI and TCI in the presence of symmetry-breaking potentials. Specifically, we will show that the presence of quadrupolar magnetism (*i.e.*, magnetism that preserves type-I wallpaper group[20] $p4m$) can gap the edge states of a 2D TI and TCI and leave behind zero-dimensional corner modes. In a 3D crystal, the Hamiltonians of 2D planes of the BZ indexed by k_z can characterize 2D TIs and TCIs[32] at the $\mathcal{T}$- (or M_z-) invariant values of k_z. Thus, in a space group with BZ planes that preserve the crystal symmetries of the QI phase (SN 3), one can consider the Hamiltonian of a BZ plane indexed by $k_z \neq 0, \pi$ as equivalent to a 2D $\mathcal{T}$-broken insulator with bulk quadrupolar magnetism. In this description, the low-energy theory derived here tracks the evolution in k_z of the 2D TI and TCI surface states of a 3D topological Dirac semimetal into the HOFA states on its 1D hinges as the strength of the effective magnetism in each 2D plane grows with increasing k_z. During the final stages of preparing this complete work, the low-energy $k \cdot p$ theory of a related QI was also analyzed in SRef. 112, though that work did not relate their $k \cdot p$ theory to TIs, TCIs, HOFA states, and fragile topology, as we do in this section.

However, it is important to note that *not every* 2D TI and TCI can be gapped into a QI; only topological (crystalline) insulators with the same symmetries and occupied bulk C_{4z} eigenvalues as those of the QI obstructed atomic limit (SN 3 and 5) can transition into QIs when gapped with quadrupolar magnetism, although other symmetries can also realize 2D insulators with topological corner states[11,39,65–73,78]. We will show in SN 7 that a 2D TI formed of hybridized p_z and $d_{x^2-y^2}$ orbitals at the $1a$ position of wallpaper group $p4m1'$ or layer group $p4/mmm1'$ (SN 3) gaps into a QI under $p4m$-preserving magnetism. We will then show in SN 8 that a very similar 2D TI, formed instead of s and p_z orbitals, gaps into a trivial insulator under $p4m$-preserving magnetism. Then, in SN 9, we will show that the edge modes of the 2D TCI phase of SEq. (1) can evolve into two different kinds of 0D corner states, depending on the symmetries that are broken. Specifically, depending on whether $\mathcal{T}$ or M_z are broken while preserving $p4m$, the resulting 2D phase is either a QI or a fragile TI with the same corner charges (modulo e) and symmetry eigenvalues (SEq. (59)) as a QI. If we break $\mathcal{T}$, the resulting phase is a QI, whether or not $M_z \times \mathcal{T}$ is preserved (SN 3), whereas if we keep $\mathcal{T}$ and break M_z, the resulting phase is fragile (SN 4). We will first show that if M_z- and $\mathcal{T}$- symmetries are broken while $p4m$ is preserved, the edge states of this 2D TCI evolve into the singly degenerate spinful corner states of a $M_z \times \mathcal{T}$-broken 2D QI in $p4m$ (SN 1). This $M_z \times \mathcal{T}$-broken QI phase appears in the $k_z \neq 0$ planes with HOFAs in the 3D Dirac semimetal in Fig. 3(e,h) of the main text. Returning to the 2D TCI phase of SEq. (1), we will then show that if just M_z symmetry is broken while preserving $p4m1'$ (and without closing the bulk gap), the 1D TCI edge states evolve into the quarter-filled Kramers pairs of corner modes[39,45] of the fragile topological phase[1–18] discussed in SN 4 (equivalent to the Hamiltonian of the $k_z = 0$ plane of the fragile topological Dirac semimetal in Fig. 3(e,h) of the main text). Finally, in SN 10, we will show that the topological corner modes of a $4mm$-symmetric QI or fragile phase remain anomalous when the system is cut into in a geometry that breaks $M_{x,y}$ while preserving C_{4z}. This is a necessary intermediate step in demonstrating that topological HOFA states are still present in nonsymmorphic Dirac semimetals (such as the archetypal Dirac semimetal α-Cd_3As_2 in SG 142 ($I4_1/acd1'$)[32,110,113,114]) whose glide reflections are formed from the combination of $M_{x,y}$ and lattice translations in the xy-plane, which cannot be preserved in z- (fourfold-axis-) directed nanorod geometries (SN 12).

We will also numerically calculate in SN 11 the position-space localization of the bulk and surface states of a HOFA Dirac semimetal terminated in a slab geometry. We will show that 2D BZ planes with Hamiltonians equivalent to QIs bind gapped edge Fermi arc-like states[115], and that 2D planes equivalent to trivial insulators generically have no edge states. Taking planes at successive values of k_z passing through one of the bulk Dirac points, we will demonstrate that the localization lengths of all surface (and hinge) states diverge exactly at the Dirac point. We therefore find no evidence supporting the presence of the additional surface cones bound to the 2D face projections of the bulk Dirac points.

7. Gapping the Edge Modes of a 2D Topological Insulator with Quadrupolar Magnetism

We will first show that a 2D TI in layer group $p4/mmm1'$ (equivalent to the Hamiltonian of the $k_z = 0$ plane of SEq. (84)), formed of p_z and $d_{x^2-y^2}$ orbitals at the $1a$ position (Fig. 2(a) of the main text), gaps into a 2D QI in the presence of magnetism that preserves the symmetries of 2D point group $4mm$. We begin with this $p-d$-hybridized 2D TI, instead of the $s-d$-hybridized 2D TCI (Fig. 2(f-h) of the main text and SEq. (1)) that is more closely related to the original formulation of the QI[26], because the bulk $k \cdot p$ theory of a $p-d$-hybridized TI is simpler to analyze due to its linear dispersion. In SN 9, we perform the analogous analysis of the quadratically dispersing $s-d$-hybridized TCI. In four-band models of both $p_z - d_{x^2-y^2}$-hybridized 2D TIs and $s - d_{x^2-y^2}$-hybridized 2D TCIs, the bands of the two models exhibit the same C_{4z} eigenvalues (SN 5). Instead, as shown in Supplementary Tables 4 and 5, the

valence and conduction bands of the two insulators are distinguished by their parity eigenvalues. As we will see in this section and in SN 9, when $\mathcal{I}$, M_z, and $\mathcal{T}$ symmetries are broken while $p4m$ is preserved, both $p_z - d_{x^2-y^2}$-hybridized 2D TIs and $s - d_{x^2-y^2}$-hybridized 2D TCIs (formed from orbitals at the $1a$ position of $p4/mmm1'$ as shown in SN 3 and 5) evolve into QIs.

To demonstrate the presence of the 0D boundary modes in the QI phase that results from gapping a $p-d$-hybridized 2D TI in layer group $p4/mmm1'$, we will first derive the low-energy continuum $k \cdot p$ theory for the bulk of a $p-d$-hybridized 2D TI. We will then use this $k \cdot p$ theory to solve a nested pair of Jackiw-Rebbi domain wall problems[116], *i.e.* one for the (gapped) 1D edge states and another for the 0D corner states.

To form the bulk $k \cdot p$ Hamiltonian of a 2D $p-d$-hybridized TI[80], we begin by expanding SEq. (84) about the Γ point to linear order and fixing $k_z = 0$:

$$\mathcal{H}_\Gamma(\mathbf{k}) = m\tau^z + vk_x\tau^x\sigma^y + vk_y\tau^x\sigma^x, \tag{95}$$

where we have taken $t_{PH} \to 0$, have combined all of the terms proportional to τ^z into a single coefficient m, and have relabeled $v_s \to v$. We also note that in SEq. (84), we have suppressed factors of the lattice constants $a_{x,y} = a$; it will be useful for future approximations to highlight that most precisely, $v = v_s a$, where we have specialized to units where $a = 1$. SEq. (95) has the symmetries of the little co-group of the Γ point, which is isomorphic to point group $4/mmm1'$. The Hamiltonian transforms for each symmetry $g \in 4/mmm1'$ under:

$$\mathcal{H}_\Gamma(k_x, k_y) \to g\mathcal{H}_\Gamma(gk_xg^{-1}, gk_yg^{-1})g^{-1}, \tag{96}$$

given in the notation of SRefs. 27, 87, 89, 90, 98, 117, and 118. We summarize this transformation as:

$$g:\ g\mathcal{H}_\Gamma(gk_xg^{-1}, gk_yg^{-1})g^{-1}. \tag{97}$$

In the notation of SEq. (97), $\mathcal{H}_\Gamma(k_x, k_y)$ transforms in the symmetry representation given by:

$$\begin{aligned}
&\mathcal{T}:\ \sigma^y\mathcal{H}^*_\Gamma(-k_x,-k_y)\sigma^y,\ M_z:\ \tau^z\sigma^z\mathcal{H}_\Gamma(k_x,k_y)\tau^z\sigma^z,\ M_x:\ \sigma^x\mathcal{H}_\Gamma(-k_x,k_y)\sigma^x,\\
&M_y:\ \sigma^y\mathcal{H}_\Gamma(k_x,-k_y)\sigma^y,\ C_{4z}:\ \tau^z\left(\frac{\mathbb{1}_\sigma - i\sigma^z}{\sqrt{2}}\right)\mathcal{H}_\Gamma(k_y,-k_x)\tau^z\left(\frac{\mathbb{1}_\sigma + i\sigma^z}{\sqrt{2}}\right),
\end{aligned} \tag{98}$$

where $\mathbb{1}_\sigma$ is the 2×2 identity in σ space. SEq. (98) implies an inversion symmetry $\mathcal{I} = M_xM_yM_z$ that transforms $\mathcal{H}_\Gamma(k_x, k_y)$ under the representation:

$$\mathcal{I}:\ \tau^z\mathcal{H}_\Gamma(-k_x,-k_y)\tau^z. \tag{99}$$

The τ^z contributions to M_z, $\mathcal{I}$, and C_{4z} in SEqs. (98) and (99) reflect that $\mathcal{H}_\Gamma(\mathbf{k})$ describes a 2D TI formed of hybridized p_z and $d_{x^2-y^2}$ orbitals, as p_z ($d_{x^2-y^2}$) orbitals are odd (even) under M_z and $\mathcal{I}$ and even (odd) under C_{4z}. $\mathcal{H}_\Gamma(\mathbf{k})$ also exhibits a unitary particle-hole symmetry:

$$\{\mathcal{H}_\Gamma(\mathbf{k}), \Pi\} = 0,\ \Pi = \tau^x\sigma^z, \tag{100}$$

which we will relax in future steps in this calculation. SEq. (95) also respects a second unitary particle-hole symmetry:

$$\{\mathcal{H}_\Gamma(\mathbf{k}), \tilde{\Pi}\} = 0,\ \tilde{\Pi} = \tau^y. \tag{101}$$

We then Fourier transform $\mathcal{H}_\Gamma(\mathbf{k})$ such that $k_{x,y} \to -i\partial_{x,y}$, and take m to have a spatial dependence $m \to m(x,y)$. Specifically, we choose the bulk gap $m(x,y)$ to be strongly negative within a region bounded by a circle of radius $R \gg a$, and strongly positive for values outside of this circle; we also take $m(x,y)$ to be isotropic in $\theta = \tan^{-1}(y/x)$. This distribution of m suggests that the position-space Hamiltonian is more naturally described in polar coordinates, and therefore we transform:

$$\begin{aligned}
\partial_x &= \cos(\theta)\partial_r - \frac{1}{r}\sin(\theta)\partial_\theta,\\
\partial_y &= \sin(\theta)\partial_r + \frac{1}{r}\cos(\theta)\partial_\theta,
\end{aligned} \tag{102}$$

such that the Hamiltonian in SEq. (95) now takes the form:

$$\mathcal{H}_\Gamma(r,\theta) = m(r)\tau^z - iv\tau^x\left[\sigma^1(\theta)\partial_r + \frac{1}{r}\sigma^2(\theta)\partial_\theta\right], \tag{103}$$

where, through a canonical transformation,

$$\sigma^1(\theta) = \sin(\theta)\sigma^x + \cos(\theta)\sigma^y = \begin{pmatrix} 0 & -ie^{i\theta} \\ ie^{-i\theta} & 0 \end{pmatrix},$$
$$\sigma^2(\theta) = \cos(\theta)\sigma^x - \sin(\theta)\sigma^y = \begin{pmatrix} 0 & e^{i\theta} \\ e^{-i\theta} & 0 \end{pmatrix},$$
$$\{\sigma^1(\theta), \sigma^2(\theta)\} = 0,\ \sigma^1(\theta)\sigma^2(\theta) = -i\sigma^z. \tag{104}$$

In this section, we employ a similar notation for the position-space, polar-coordinate forms of the symmetries of $\mathcal{H}_\Gamma(r,\theta)$ as we previously employed in SEq. (97); for each symmetry g, the Hamiltonian transforms under:

$$\mathcal{H}_\Gamma(r,\theta) \to g\mathcal{H}_\Gamma(grg^{-1}, g\theta g^{-1})g^{-1}. \tag{105}$$

We summarize this transformation as:

$$g:\ g\mathcal{H}_\Gamma(grg^{-1}, g\theta g^{-1})g^{-1}. \tag{106}$$

In the notation of SEq. (106), $\mathcal{H}_\Gamma(r,\theta)$ transforms in the symmetry representation given by:

$$\mathcal{T}:\ \sigma^y\mathcal{H}^*_\Gamma(r,\theta)\sigma^y,\ M_z:\ \tau^z\sigma^z\mathcal{H}_\Gamma(r,\theta)\tau^z\sigma^z,\ M_x:\ \sigma^x\mathcal{H}_\Gamma(r,\pi-\theta)\sigma^x,$$
$$M_y:\ \sigma^y\mathcal{H}_\Gamma(r,-\theta)\sigma^y,\ C_{4z}:\ \tau^z\left(\frac{\mathbb{1}_\sigma - i\sigma^z}{\sqrt{2}}\right)\mathcal{H}_\Gamma(r,\theta+\pi/2)\tau^z\left(\frac{\mathbb{1}_\sigma + i\sigma^z}{\sqrt{2}}\right),$$
$$\mathcal{I}:\ \tau^z\mathcal{H}_\Gamma(r,\theta+\pi)\tau^z, \tag{107}$$

and both particle-hole symmetries remain in the same form as previously in SEqs. (100) and (101):

$$\{\mathcal{H}_\Gamma(r,\theta), \Pi\} = 0,\ \Pi = \tau^x\sigma^z, \tag{108}$$

and:

$$\{\mathcal{H}_\Gamma(r,\theta), \tilde{\Pi}\} = 0,\ \tilde{\Pi} = \tau^y. \tag{109}$$

We search for zero-energy bound states of SEq. (103) on a disc geometry:

$$\mathcal{H}_\Gamma(r,\theta)|\psi(r,\theta)\rangle = 0. \tag{110}$$

To solve SEq. (110), we separate variables by left-multiplying by τ^x; after canceling a factor of $-i$:

$$\left[m(r)\tau^y + v\sigma^1(\theta)\partial_r\right]|\psi(r,\theta)\rangle = -\frac{v}{r}\sigma^2(\theta)\partial_\theta|\psi(r,\theta)\rangle. \tag{111}$$

Because we are only interested in solving SEq. (111) in the (linear) $k\cdot p$ regime, we will use a series of approximations to find a diagonal solution; though these approximations are not strictly necessary, they provide considerable convenience in the early stages of this calculation while ultimately not affecting the final (topological) result. First, we recognize that the bound state $|\psi(r,\theta)\rangle$ is almost entirely localized at $r\approx R$, where $m(r)\to 0$. As $R \gg a$, where a is the lattice spacing and $v \propto a$, the right-hand side of SEq. (111) vanishes to leading order:

$$\left[m(r)\tau^y + v\sigma^1(\theta)\partial_r\right]|\psi(r,\theta)\rangle \approx 0. \tag{112}$$

We note that SEq. (111) can still be exactly solved without exploiting this approximation (it is the $k\cdot p$ differential equation for the edge states of a circular 2D TI, whose exact solution is a 1D Dirac fermion subject to the effects of curvature[119,120]). Nevertheless, for the purpose of the explicit proofs in this section, SEq. (112) is advantageous in that it can be diagonalized simply by left-multiplying by τ^y and integrating, which allows us to circumvent at this stage of the calculation some of the complications that arise from the circular geometry (*e.g.* the fact that ∂_θ acts on $\sigma^{1,2}(\theta)$ and the $1/r$ dependence of the right-hand side of SEq. (111)):

$$|\psi_{1,2}(r,\theta)\rangle = \frac{1}{\sqrt{N}}e^{-\frac{1}{v}\int_R^r m(r')dr'}|\tau^y_\pm\sigma^1_\pm(\theta)\rangle = \mathcal{R}(r)|\tau^y_\pm\sigma^1_\pm(\theta)\rangle, \tag{113}$$

where the normalization constant N has the units of length squared, as the radial part of the measure in polar coordinates is rdr:

$$\int_0^\infty rdr|\mathcal{R}(r)|^2 = 1. \tag{114}$$

In SEq. (113):

$$|\tau_\pm^y \sigma_\pm^1(\theta)\rangle = |\tau_\pm^y\rangle \otimes |\sigma_\pm^1(\theta)\rangle, \tag{115}$$

where $|\tau_\pm^i, \sigma_\pm^j\rangle$ are the eigenstates with eigenvalues ± 1 of the 2×2 Pauli matrices τ^i and σ^j. To leading order, SEq. (113) indicates that there are two nondispersing zero modes localized on the boundary of this circle with radius R, or close to the region where $m(r) = 0$. We note that, in the limit that the mass $m(r')$ in SEq. (113) is rapidly changing in the vicinity of $r \approx R$ (*i.e.*, that $\left|\frac{dm(r')}{dr'}\right| \gg \frac{v}{R^2}$), the radial component $\mathcal{R}(r)$ simplifies:

$$|\mathcal{R}(r)|^2 \to \frac{1}{r}\delta(r-R). \tag{116}$$

However, more generally, like with the SSH chain[21,22], the presence or absence of zero modes of the form of SEq. (113) that are localized in the vicinity of $r \approx R$ does not depend on the form of $m(r)$ – it only depends on whether $m(r)$ changes sign at[121] $r = R$.

For subsequent calculations, we will find that the symmetries of the edge Hamiltonian appear in a more familiar form in the rotated basis:

$$|\phi_1(r,\theta)\rangle = \frac{1}{\sqrt{2}}\left(|\psi_1(r,\theta)\rangle + |\psi_2(r,\theta)\rangle\right) = \frac{\mathcal{R}(r)}{\sqrt{2}}\begin{pmatrix} -e^{i\theta} \\ 0 \\ 0 \\ 1 \end{pmatrix} = R(r)|\xi_1(\theta)\rangle$$
$$|\phi_2(r,\theta)\rangle = -\frac{ie^{-i\theta}}{\sqrt{2}}\left(|\psi_1(r,\theta)\rangle - |\psi_2(r,\theta)\rangle\right) = \frac{\mathcal{R}(r)}{\sqrt{2}}\begin{pmatrix} 0 \\ e^{-i\theta} \\ 1 \\ 0 \end{pmatrix} = R(r)|\xi_2(\theta)\rangle, \tag{117}$$

a transformation that we are free to make because $|\phi_{1,2}(\theta)\rangle$ are degenerate (zero modes) at all values of θ at this stage of the calculation.

We now perturbatively restore the angular velocity term from SEq. (103) by projecting it into the basis of the edge states of $|\phi_{1,2}(r,\theta)\rangle$, integrating out r, and exploiting SEq. (116):

$$\begin{aligned}\mathcal{H}^{TI}_{edge,ij}(\theta) &= -iv\langle\phi_i(r,\theta)|\frac{1}{r}\tau^x\sigma^2(\theta)\partial_\theta|\phi_j(r,\theta)\rangle \\ &= -iv\int_0^\infty rdr\left(\frac{|\mathcal{R}(r)|^2}{r}\right)\left[\langle\langle\xi_i(\theta)|\tau^x\sigma^2(\theta)|\partial_\theta\xi_j(\theta)\rangle\rangle + \langle\langle\xi_i(\theta)|\tau^x\sigma^2(\theta)|\xi_j(\theta)\rangle\rangle\partial_\theta\right] \\ \mathcal{H}^{TI}_{edge}(\theta) &= \frac{v}{R}\left(\frac{1}{2}\mathbb{1}_s + is^z\partial_\theta\right),\end{aligned} \tag{118}$$

where s^z is a Pauli matrix and $\mathbb{1}_s$ is the identity matrix in the 2×2 basis of $|\phi_{1,2}(r,\theta)\rangle$, the $\langle\langle$ and $\rangle\rangle$ symbols in the second line indicate θ-independent contractions over 4×4 matrices. The constant term $(v/2R)\mathbb{1}_s$ arises due to the action of ∂_θ on $|\phi_{1,2}(r,\theta)\rangle$ in SEq. (118). The form of this term depends on the choice of gauge in SEq. (117); it will be useful for future calculations to note that the constant term disappears under the anti- (4π-) periodic gauge transformation:

$$|\xi_1(r,\theta)\rangle \to e^{-i\theta/2}|\xi_1(r,\theta)\rangle = |\tilde{\xi}_1(r,\theta)\rangle,\ |\xi_2(r,\theta)\rangle \to e^{i\theta/2}|\xi_2(r,\theta)\rangle = |\tilde{\xi}_2(r,\theta)\rangle, \tag{119}$$

where $|\partial_\theta \tilde{\xi}_{1,2}\rangle$ are the positive and negative eigenstates of $\tau^x\sigma^2(\theta)$:

$$|\tilde{\xi}_1(\theta)\rangle = \frac{1}{\sqrt{2}}\begin{pmatrix} -e^{\frac{i\theta}{2}} \\ 0 \\ 0 \\ e^{-\frac{i\theta}{2}} \end{pmatrix},\ |\partial_\theta\tilde{\xi}_1(\theta)\rangle = \frac{i}{2\sqrt{2}}\begin{pmatrix} -e^{\frac{i\theta}{2}} \\ 0 \\ 0 \\ -e^{-\frac{i\theta}{2}} \end{pmatrix},\ \tau^x\sigma^2(\theta)|\partial_\theta\tilde{\xi}_1(\theta)\rangle = \frac{i}{2\sqrt{2}}\begin{pmatrix} -e^{\frac{i\theta}{2}} \\ 0 \\ 0 \\ -e^{-\frac{i\theta}{2}} \end{pmatrix}$$

$$|\tilde{\xi}_2(\theta)\rangle = \frac{1}{\sqrt{2}}\begin{pmatrix} 0 \\ e^{-\frac{i\theta}{2}} \\ e^{\frac{i\theta}{2}} \\ 0 \end{pmatrix},\ |\partial_\theta\tilde{\xi}_2(\theta)\rangle = \frac{i}{2\sqrt{2}}\begin{pmatrix} 0 \\ -e^{-\frac{i\theta}{2}} \\ e^{\frac{i\theta}{2}} \\ 0 \end{pmatrix},\ \tau^x\sigma^2(\theta)|\partial_\theta\tilde{\xi}_2(\theta)\rangle = \frac{i}{2\sqrt{2}}\begin{pmatrix} 0 \\ e^{-\frac{i\theta}{2}} \\ -e^{\frac{i\theta}{2}} \\ 0 \end{pmatrix}, \tag{120}$$

such that in SEq. (118):

$$\langle\langle\tilde{\xi}_i(\theta)|\tau^x\sigma^2(\theta)|\tilde{\xi}_j(\theta)\rangle\rangle = 0 \text{ for } i,j = 1,2. \tag{121}$$

In the basis of $|\phi_{1,2}(r,\theta)\rangle$, the symmetries from SEq. (107) transform $\mathcal{H}^{TI}_{edge}(\theta)$ under the representation:

$$\begin{aligned}&\mathcal{T}:\ s^y(\mathcal{H}^{TI}_{edge}(\theta))^* s^y,\ M_z:\ s^z\mathcal{H}^{TI}_{edge}(\theta)s^z,\ M_x:\ s^x\mathcal{H}^{TI}_{edge}(\pi-\theta)s^x,\\ &M_y:\ s^y\mathcal{H}^{TI}_{edge}(-\theta)s^y,\ C_{4z}:\ \left(\frac{\mathbb{1}_s - is^z}{\sqrt{2}}\right)\mathcal{H}^{TI}_{edge}(\theta+\pi/2)\left(\frac{\mathbb{1}_s + is^z}{\sqrt{2}}\right),\\ &\mathcal{I}:\ \mathcal{H}^{TI}_{edge}(\theta+\pi),\end{aligned} \tag{122}$$

Crucially, in the basis of $|\phi_{1,2}(r,\theta)\rangle$, the particle-hole symmetry from SEq. (108) takes a θ-dependent form:

$$\Pi(\theta) = s^1(\theta), \tag{123}$$

where:

$$\begin{aligned}s^1(\theta) &= \cos(\theta)s^x - \sin(\theta)s^y = \begin{pmatrix} 0 & e^{i\theta} \\ e^{-i\theta} & 0 \end{pmatrix},\\ s^2(\theta) &= \sin(\theta)s^x + \cos(\theta)s^y = \begin{pmatrix} 0 & -ie^{i\theta} \\ ie^{-i\theta} & 0 \end{pmatrix},\\ \{s^1(\theta), s^2(\theta)\} &= 0,\ s^1(\theta)s^2(\theta) = is^z.\end{aligned} \tag{124}$$

The θ dependence of particle-hole symmetry in SEq. (123) and the presence of the constant term $(v/2R)\mathbb{1}_s$ in SEq. (118) reflect the extrinsic curvature of the circular boundary. Though $(v/2R)\mathbb{1}_s$ moves the center of the spectrum away from $E=0$, the θ-dependent particle-hole symmetry is still preserved, as the Hamiltonian acts on θ:

$$\{\mathcal{H}^{TI}_{edge}(\theta), \Pi(\theta)\} = 0, \tag{125}$$

where specifically:

$$\partial_\theta s^1(\theta) = -s^2(\theta) + s^1(\theta)\partial_\theta. \tag{126}$$

As in SEq. (109), SEq. (118) similarly also respects a second theta-dependent particle-hole symmetry of the form:

$$\tilde{\Pi}(\theta) = s^2(\theta), \tag{127}$$

where:

$$\partial_\theta s^2(\theta) = s^1(\theta) + s^2(\theta)\partial_\theta. \tag{128}$$

Though many previous works have demonstrated the presence of localized 0D modes in systems with sharp corners[26,28,39,65–73], for which the curvature is zero on the edges and singular on the corners, our explicit calculation of the QI boundary states in a geometry with constant curvature (*i.e.*, on a disc) will allow us to separate the extrinsic effects of sharp corners from the intrinsic (higher-order) topological bulk-boundary (-corner) correspondence of QIs. It is also important to note that, as $\mathcal{H}^{TI}_{edge}(\theta)$ describes the edge Hamiltonian at $r \sim R$, it is invariant under fewer

symmetry restrictions than a Hamiltonian localized in a region containing $r = 0$ (*i.e.*, the origin of the symmetry operations of the point group $4/mmm1'$). At generic values of θ, $\mathcal{H}^{TI}_{edge}(\theta)$ (SEq. (118)) is only invariant under the θ-preserving action of $\mathcal{T}$ and M_z in SEq. (122); the other symmetries of $4/mmm1'$ act at generic angles θ to relate $\mathcal{H}^{TI}_{edge}(\theta)$ to its value at another, symmetry-related generic angle θ'. Point group $4/mmm1'$ also has four mirror lines in the xy-plane[40] (Fig. 2(a) of the main text), $M_{x,y}$ and $M_{x\pm y}$ that fix the angles:

$$\tilde{\theta}_n = n\pi/4,\ n \in \mathbb{Z}, \tag{129}$$

such that one of $M_{x,y}$ or $M_{x\pm y}$ is a symmetry of $\mathcal{H}^{TI}_{edge}(\theta)$ at each $\tilde{\theta}_n$:

$$M_{x,y}\mathcal{H}^{TI}_{edge}(\tilde{\theta}_n)M^{-1}_{x,y} \text{ or } M_{x\pm y}\mathcal{H}^{TI}_{edge}(\tilde{\theta}_n)M^{-1}_{x\pm y}. \tag{130}$$

When M_z and $\mathcal{T}$ symmetries are broken to gap the TI edge states, we will see that QI-nontrivial 0D states become bound to the $\tilde{\theta}_n$, *i.e.*, the fixed points (angles) of point group $4mm$.

We now gap $\mathcal{H}^{TI}_{edge}(\theta)$ (SEq. (118)) by introducing quadrupolar ($p4m$-preserving) magnetism. We begin by proposing the most general r-independent bulk potential to add to $\mathcal{H}_\Gamma(r,\theta)$ (SEq. (103)):

$$U(\theta) = \sum_{L_z=0}^{\infty}\sum_{\mu=\pm} m^{\mu}_{L_z}\Gamma^{L_z,\mu}f^{\mu}_{L_z}(\theta), \tag{131}$$

where $\Gamma^{L_z,\mu}$ is a 4×4 matrix in the basis of $\tau\otimes\sigma$ and $f^{\pm}_{L_z}(\theta)$ is a real circular harmonic[99,122–124]:

$$f^{+}_{L_z}(\theta) = \cos(L_z\theta),\ f^{-}_{L_z}(\theta) = \sin(L_z\theta), \tag{132}$$

with angular momentum L_z. The sum in SEq. (131) is taken over all possible products of 4×4 matrices and $f^{\pm}_{L_z}(\theta)$ that respect the symmetries of point group $4mm$ (the point group of $p4m$). In terms of the more familiar spherical harmonics, the functions $f^{\pm}_{L_z}(\theta)$ derive from the set of "cubic harmonics"[124], *i.e.*, the real-valued linear combinations of the spherical harmonics that define the angular dependence of the wavefunctions of the atomic orbitals[123,124]. Specifically, choosing the z-axis to be the plane normal, the real circular harmonics are obtained by taking $z\to 0$ in the subset of cubic harmonics (atomic orbitals) for which the total angular momentum L equals the magnitude of the z-component of the angular momentum L_z, which we refer to as the angular momentum of the circular harmonic[99,122–124] (SEq. (132)).

We next explicitly expand SEq. (131) by choosing all possible mass terms that respect the symmetries of $4mm$ (SEq. (107)) while containing 4×4 matrices $\Gamma^{L_z,\mu}$ that anticommute with the Dirac matrix coefficients of the bulk mass and angular velocity terms in $\mathcal{H}_\Gamma(r,\theta)$ (SEq. (103)): $m(r)\tau^z$ and $-i(v/r)\tau^x\sigma^2(\theta)\partial_\theta$, respectively. This guarantees that the terms in $U(\theta)$, when individually added to $\mathcal{H}_\Gamma(r,\theta)$, strictly enlarge the bulk gap and open an edge gap[50]. Expressing $U(\theta)$ as a sum of terms that respect the symmetries of $4mm$ (C_{4z} and $M_{x,y}$ in SEq. (107)):

$$\begin{aligned} U(\theta) = {}& \tau^x\sigma^z\left[m^-_2\sin(2\theta) + m^-_6\sin(6\theta) + m^-_{10}\sin(10\theta) + \dots\right] \\ &+ \tau^y\left[m^+_2\cos(2\theta) + m^+_6\cos(6\theta) + m^+_{10}\cos(10\theta) + \dots\right] \\ &+ \tau^x\sigma^1(\theta)\left[m^+_0 + m^+_4\cos(4\theta) + m^+_8\cos(8\theta) + \dots\right] \\ &+ \tau^y\sigma^2(\theta)\left[m^-_4\sin(4\theta) + m^-_8\sin(8\theta) + m^-_{12}\sin(12\theta) + \dots\right]. \end{aligned} \tag{133}$$

We observe that the terms in $U(\theta)$ group into circular harmonics of increasing L_z multiplied by one of four 4×4 matrices. We then project $U(\theta)$ into the basis of the edge modes $|\phi_{1,2}(r,\theta)\rangle$, following the procedure in SEq. (118) and SRef. 50:

$$\begin{aligned} U_{edge,ij}(\theta) &= \langle\phi_i(r,\theta)|U(\theta)|\phi_j(r,\theta)\rangle \\ &= \sum_{L_z,\mu} m^{\mu}_{L_z}\langle\phi_i(r,\theta)|\Gamma^{L_z,\mu}|\phi_j(r,\theta)\rangle f^{\mu}_{L_z}(\theta), \end{aligned} \tag{134}$$

$$\begin{aligned} U_{edge}(\theta) &= s^1(\theta)\left[m^-_2\sin(2\theta) + m^-_6\sin(6\theta) + m^-_{10}\sin(10\theta) + \dots\right] \\ &+ s^2(\theta)\left[m^+_2\cos(2\theta) + m^+_6\cos(6\theta) + m^+_{10}\cos(10\theta) + \dots\right]. \end{aligned} \tag{135}$$

We observe that the terms in $U(\theta)$ that commute with $\tau^y\sigma^1(\theta)$ ($m^{\pm}_{2+4a}$) have nonzero edge projections, whereas the terms that anticommute with $\tau^y\sigma^1(\theta)$ ($m^{\pm}_{4a}$) project to zero in $U_{edge}(\theta)$ and hence do not open an edge gap. The nonzero terms in $U_{edge}(\theta)$ break $\mathcal{I}$, M_z, and $\mathcal{T}$ symmetries in the bulk and on the edge, while respecting the combined

magnetic symmetries $\mathcal{I}\times\mathcal{T}$ and $M_z\times\mathcal{T}$ (SEq. (122)). To understand this result, we form the expression for the projector into the positive eigenspace of $\tau^y\sigma^1(\theta)$, *i.e.* the space of eigenvectors with eigenvalues $\lambda_{\tau^y\sigma^1(\theta)}=1$:

$$P_{\lambda_{\tau^y\sigma^1(\theta)}=1}=\frac{\mathbb{1}+\tau^y\sigma^1(\theta)}{2}. \tag{136}$$

In order for a generic 4×4 matrix $\Gamma^{L_z,\mu}$ to have a nonzero projection into the basis of $|\phi_{1,2}(r,\theta)\rangle$, it must satisfy:

$$P_{\lambda_{\tau^y\sigma^1(\theta)}=1}\Gamma^{L_z,\mu}P_{\lambda_{\tau^y\sigma^1(\theta)}=1}\neq 0. \tag{137}$$

SEq. (137) can only be satisfied if:

$$\{\tau^y\sigma^1(\theta),\Gamma^{L_z,\mu}\}\neq 0. \tag{138}$$

As the basis of edge states $|\phi_{1,2}(r,\theta)\rangle$ is formed from linear combinations of the positive eigenstates of $\tau^y\sigma^1(\theta)$ (SEq. (117)), then SEqs. (136), (137), and (138) imply that the $m^{\pm}_{4a}$ terms in $U(\theta)$ (SEq. (133)) project to zero in $U_{edge}(\theta)$ (SEq. (135)). This indicates that, for the $p-d$-hybridized TI in this section, bulk $p4m$-preserving magnetism can only open an edge gap with:

$$L_z^{QI}=2+4a,\ a\in\mathbb{Z}, \tag{139}$$

where the $m^{\pm}_2$ terms in SEq. (135), in particular, are proportional to the circular harmonics of $d_{x^2-y^2}$ and d_{xy} orbitals, respectively[99,122–124].

We next confirm that $U_{edge}(\theta)$ is proportional to the representations of $M_{x,y}$ and $M_{x\pm y}=C^{\pm 1}_{4z}M_x$ (SEq. (122)) at the first four mirror-invariant points in θ (SEq. (129)):

$$U\left(0\right)\propto s^y,\ U\left(\frac{\pi}{4}\right)\propto(s^x-s^y),\ U\left(\frac{\pi}{2}\right)\propto -s^x,\ U\left(\frac{3\pi}{4}\right)\propto(s^x+s^y), \tag{140}$$

and thus verify that $U_{edge}(\theta)$ respects the mirror symmetries at those points. We define the edge Hamiltonian of the QI to be:

$$\mathcal{H}^{QI}_{edge}(\theta)=\mathcal{H}^{TI}_{edge}(\theta)+U_{edge}(\theta). \tag{141}$$

If we truncate $U_{edge}(\theta)$ (SEq. (135)) to its leading two $L_z=2$ terms, $\mathcal{H}^{QI}_{edge}(\theta)$ exhibits a gap in the long-wavelength limit of:

$$\Delta(\theta)=2\sqrt{(m^-_2)^2\sin^2(2\theta)+(m^+_2)^2\cos^2(2\theta)}. \tag{142}$$

With this formality established, we now show that SEq. (141) exhibits a quantized quadrupole moment. We will first demonstrate this in a particle-hole-symmetric limit, after which we will show that the quadrupole moment of SEq. (141) remains quantized when particle-hole symmetry is relaxed. Particle-hole symmetry as represented in SEq. (123) ($\Pi(\theta)=s^1(\theta)$) is also a symmetry of all of the $m^+_{L_z^{QI}}$ mass terms (but not the $m^-_{L_z^{QI}}$ terms) in SEq. (135) (most generally, as indicated in SEq. (127), there is a also second particle-hole symmetry $\tilde{\Pi}(\theta)=s^2(\theta)$ that is also a symmetry of $\mathcal{H}^{TI}_{edge}$ and only the $m^-_{L_z^{QI}}$ mass terms in SEq. (135)). In the specific particle-hole symmetric limit of SEq. (141) in which the only particle-hole symmetry is $\Pi(\theta)=s^1(\theta)$, we can first choose m^+_2 to be the only nonzero mass term in $U_{edge}(\theta)$. In this limit, $\mathcal{H}^{QI}_{edge}(\theta)$ is gapless at $\theta=\theta_n$, where the first four independent values of θ_n are:

$$\theta_n=\pi/4+n\pi/2,\ n\in\{0,1,2,3\}. \tag{143}$$

We then solve for the zero modes bound at θ_n, or at the values of θ at which $\cos(2\theta)$ changes sign, by formulating a Jackiw-Rebbi problem:

$$\left[\frac{v}{R}\left(\frac{1}{2}\mathbb{1}_s+is^z\partial_\theta\right)+m^+_2\cos(2\theta)s^2(\theta)\right]|\tilde{\Theta}(\theta)\rangle=0. \tag{144}$$

We will solve for the bound states of SEq. (144) in two steps: first we will remove the constant curvature term $(v/2R)\mathbb{1}_s$ in SEq. (144) by transforming $|\tilde{\Theta}(\theta)\rangle$ into the antiperiodic gauge in SEq. (119), which will allow SEq. (144)

to be solved using the same method that we used for SEq. (112). We will then Taylor expand SEq. (144) around θ_n (SEq. (143)) to solve for 0D bound states at each θ_n.

We first explicitly demonstrate that transforming $|\tilde{\Theta}(\theta)\rangle$ into a wavefunction $|\Theta(\theta)\rangle$ in an antiperiodic gauge removes the constant curvature term in SEq. (144). We express the transformation between $|\tilde{\Theta}(\theta)\rangle$ and $|\Theta(\theta)\rangle$ as:

$$|\tilde{\Theta}(\theta)\rangle = U(\theta)|\Theta(\theta)\rangle. \tag{145}$$

In order for the constant term to vanish, $|\tilde{\Theta}(\theta)\rangle$ must satisfy:

$$\left(\frac{1}{2}\mathbb{1}_s + is^z\partial_\theta\right)|\tilde{\Theta}(\theta)\rangle = is^z\partial_\theta|\Theta(\theta)\rangle, \tag{146}$$

such that $|\Theta(\theta)\rangle$ behaves as if it is a θ-independent eigenstate of a linear Hamiltonian without curvature[125]. SEqs. (145) and (146) imply that:

$$is^z\partial_\theta U(\theta) = -\frac{1}{2}U(\theta), \tag{147}$$

which is satisfied by:

$$U(\theta) = \begin{pmatrix} e^{i\theta/2} & 0 \\ 0 & e^{-i\theta/2} \end{pmatrix}. \tag{148}$$

The antiperiodicity of SEq. (148) reflects that curvature in a circular (cylindrical) geometry acts as an effective π flux[120]. While one might be concerned by the antiperiodic boundary conditions of $|\Theta(\theta)\rangle$ in SEqs. (145) and (148), we note that $|\Theta(\theta)\rangle$ will only be used here as the wavefunction of a single 0D bound state that is exponentially localized within a small vicinity of one of the angles θ_n (SEq. (143)). We postulate that, for the wavefunction of each 0D bound state at $\theta = \theta_n$, 2π periodicity can be restored, by adding a θ-dependent local gauge transformation into the gapped region far away from θ_n. For sufficiently large circular boundaries in the thermodynamic limit, these smooth, but highly localized, gauge transformations should have a negligible effect on the (gauge-independent) spectrum[49], because, for each 0D bound state, the 2π-periodicity-restoring gauge transformation can be placed in a region where the bound state wavefunction is nearly zero (*i.e.*, at $\theta_n + \pi$ for each bound state at θ_n). By substituting SEq. (145) into SEq. (144), we remove the constant curvature term:

$$\left[i\frac{v}{R}s^z\partial_\theta + m_2^+\cos(2\theta)s^2(\theta)\right]|\Theta(\theta)\rangle = 0. \tag{149}$$

We next expand SEq. (149) around $\theta = \theta_n + \epsilon$ (SEq. (143)), where ϵ is a small parameter, to form an angular Jackiw-Rebbi problem for the zero-energy normalizable bound state at each θ_n:

$$\left[i\frac{v}{R}s^z\partial_\epsilon - (-1)^n m_\theta\epsilon s^2(\theta_n)\right]|\Theta(\theta_n,\epsilon)\rangle = 0, \tag{150}$$

where θ_n is given in SEq. (143) such that $s^2(\theta_n)$ is the matrix $s^2(\theta)$ in SEq. (124) evaluated at θ_n, and where:

$$m_2^+\cos[2(\theta_n+\epsilon)] \to -2m_2^+\epsilon\,\mathrm{sgn}[\sin(2\theta_n)] = -(-1)^n m_\theta\epsilon, \tag{151}$$

where $m_\theta = 2m_2^+$. The factor of $-(-1)^n$ in SEq. (150) enforces that, for increasing ϵ, the domain-wall mass $m_2^+\cos(2\theta) \to -(-1)^n m_\theta\epsilon$ in SEq. (151) exhibits a derivative with the respective signs $\{-,+,-,+\}$ at $\theta_n = \{\pi/4, 3\pi/4, 5\pi/4, 7\pi/4\}$. Next, we solve SEq. (151) for all values of θ_n by left-multiplying by $s^2(\theta_n)$ (exploiting that SEq. (124) can be rearranged to obtain $s^z s^2(\theta_n) = -is^1(\theta_n)$), and then integrating (exploiting that $\epsilon^2/2 = \int_0^\epsilon \epsilon' d\epsilon'$). We find that, over the circumference of the circle, there are four bound states of the form:

$$\begin{aligned}|\Theta(\theta_n,\epsilon)\rangle &= \frac{1}{\sqrt{N}}e^{-\lambda(\theta_n)(-1)^n\frac{m_\theta R}{2v}\epsilon^2}|s^1(\theta_n)\rangle_{\lambda(\theta_n)}, \\ &= \frac{1}{\sqrt{N}}e^{-\frac{m_\theta R}{2v}\epsilon^2}|s^1(\theta_n)\rangle_{\lambda(\theta_n)},\end{aligned} \tag{152}$$

where we have simplified by exploiting that $|s^1(\theta_n)\rangle_{\lambda(\theta_n)}$ is the eigenstate of $s^1(\theta_n)$ with eigenvalue:

$$\lambda(\theta_n) = (-1)^n. \tag{153}$$

We therefore find that when m_2^+ is the only nonzero mass term in SEq. (135), there are Jackiw-Rebbi zero modes[116] localized to the zeroes θ_n of $\cos(2\theta)$:

$$|\Theta(\pi/4,\epsilon)\rangle \propto |s^1(\pi/4)\rangle_+,\ |\Theta(3\pi/4,\epsilon)\rangle \propto |s^1(3\pi/4)\rangle_-,\ |\Theta(5\pi/4,\epsilon)\rangle \propto |s^1(5\pi/4)\rangle_+,\ |\Theta(7\pi/4,\epsilon)\rangle \propto |s^1(7\pi/4)\rangle_-, \tag{154}$$

where all of the coefficients in the proportionalities are real and of the form of the Gaussian exponential in SEq. (152). As $\{C_{4z}, s^1(\theta)\} = 0$ ($\{M_{x,y}, s^1(\theta)\} = 0$), acting with C_{4z} ($M_{x,y}$) on a positive eigenstate of $s^1(\theta_n)$ transforms it to a *negative* eigenstate of $s^1(C_{4z}\theta_n C_{4z}^{-1})$ ($s^1(M_{x,y}\theta_n M_{x,y}^{-1})$) (SEq. (106)). The set of four states in SEq. (154) is left invariant under C_{4z} and $M_{x,y}$, and thus the four zero modes as a set respect the symmetries of point group $4mm$.

By observing the profile of $m_2^+\cos(2\theta)$ and choosing the convention in which the $n=0$ corner mode is positively charged, we determine that the zero modes in SEq. (154) are (anti-) solitons at $\theta = \pi/4, 5\pi/4$ ($3\pi/4, 7\pi/4$) which acquire a charge[21,22,126,127] $+e/2$ ($-e/2$) when C_{4z} is "softly" broken[26] to C_{2z} (SFig. 9(a)). Specifically, in this work, we use the convention in which valence states, when occupied, carry a charge e, and conduction states, when occupied, carry a charge $-e$. The four zero modes in SEq. (154) are formed from a $4mm$-symmetry related set of two valence and two conduction states, each of which is half filled on the average, and can therefore be expressed as fully filled or empty linear combinations of valence (electron) and conduction (hole) states (*i.e.*, solitons and antisolitons) with fractional charge[21,22,127]. Each 0D bound state then individually carries a charge $\pm e/2$, depending on whether its wavefunction is an even (soliton) or an odd (antisoliton) linear combination of a valence and a conduction state. However, whereas the energy spectrum of the disc still respects the full point group $4mm$, the charge assignment of the bound states (corner modes) only respects C_{2z}; like in the SSH chain[127], we take this symmetry breaking to be "soft" in the sense that the number of electrons (*i.e.*, the filling of the bound states) does not affect the energy spectrum itself. This charge distribution can be summarized as:

$$q(\theta_n) = q_n = \frac{e}{2}(-1)^n. \tag{155}$$

The zero modes occupy the $4b$ Wyckoff positions ($\pm x, \pm x$) of point group[40] $4mm$ (SFig. 10). As $s^1(\theta_n) \propto s^x - (+)\ s^y$ for even (odd) n (SEq. (124)), these four zero modes are eigenstates of the diagonal mirrors $M_{x\mp y}$ (SEq. (140)). In every direction, this distribution of charge (SEq. (155)) has a zero dipole moment (SEq. (47)). To determine the xy-quadrupole moment, we reexpress SEq. (52) as the sum of contributions from n charged particles confined to a ring of radius R:

$$Q^{xy} = \frac{3}{2}\sum_n q_n x_n y_n = \frac{3R^2}{4}\sum_n q_n \sin(2\theta_n), \tag{156}$$

where q_n is defined in SEq. (155). For the four 0D modes in our calculation, which have fractional charges given by SEq. (155) and lie at the zeroes of $\cos(2\theta)$, SEq. (156) indicates that $Q^{xy} = (3R^2)e/2$. To compare this with the value obtained through the Wannier description of the QI in SN 3 of $Q^{xy} = e/2$ per square unit cell in the units of $3a^2/2$, where a is the lattice spacing, we can imagine that the four 0D states in SFig. 9 occupy the corners of a 2D square crystal with N unit cells and an overall diagonal length of $2R$. For this square, the quadrupole moment of the corner modes is $Q^{xy} = e/2$ in the units of $3a^2N/2$, or $3a^2/2$ per unit cell, in agreement with the bulk quadrupole moment obtained in SN 3.

To see that this $e/2$ quadrupole moment is a general bulk property, and not merely a unique feature of the m_2^+ term in SEq. (135), we perform two more analyses: we first consider setting $m_2^+ \to 0$ in SEq. (135) and instead tuning one of the other mass terms away from zero, and we then demonstrate that the quadrupole moment survives under the relaxation of particle-hole symmetry via the introduction of multiple nonzero mass terms in SEq. (135).

Before breaking particle-hole symmetry, we first consider how the previous analysis in SEqs. (143) to (156) is modified by instead taking $m_6^+\cos(6\theta)s^2(\theta)$ to be the only nonzero term in SEq. (135). The $L_z = 6$ circular harmonic $\cos(6\theta)$ has 12 zeroes on a circle, which occur at $\pi/12 + n\pi/6$ where n is an integer between 0 and 11. More generally, we can state that for a general circular harmonic $\cos(L_z\theta)$, it will have $2L_z$ zeroes located at:

$$\theta_n = \pi/(2L_z) + n\pi/L_z, n \in \{0, 1, \ldots, 2L_z - 1\}. \tag{157}$$

For the specific case of $L_z = L_z^{QI} = 2 + 4a$ (SEq. (139)) for the $m^{\pm}_{L_z^{QI}}$ mass terms in SEq. (135), SEq. (157) implies that each mass term will individually contribute $2L_z = 4 + 8a$ 0D zero modes. As the first derivative of *any* circular harmonic is at an extremum at a zero of that harmonic and alternates in sign at each zero in increasing θ (SEq. (132)), then SEqs. (150) and (155) also apply here, *without further modification.* We can therefore conclude that introducing m_6^+ to the $p-d$-hybridized TI in this section as the only mass term results in a circular boundary with 6 solitons and 6 antisolitions, *i.e.* 0D modes with alternating charge $\pm e/2$ localized at the θ_n in SEq. (157) for $L_z = 6$. This charge

distribution is still characterized by SEq. (155), with n taken most generally over the range 0 to $2L_z - 1$, and thus here specifically from $n = 0$ to 11. Four of these charges, those with:

$$n_{4b} = \{0, 3, 6, 9\} \tag{158}$$

lie at the same locations, *i.e.*, the $4b$ position of $4mm$ (SFig. 10), as did the four charges with $Q^{xy} = e/2$ for the $L_z = 2$ case in SFig. 9. The remaining 8 charges occupy the general position ($8c$) (SFig. 10). We use SEq. (156) to express the quadrupole moment of these 12 charges (SEq. (155)) as a sum over the contributions from those at $4b$ and those at $8c$:

$$Q^{xy} = \frac{3R^2}{4}\left(\sum_{n\in n_{4b}} q_n \sin(2\theta_n) + \sum_{n\notin n_{4b}} q_n \sin(2\theta_n)\right) \tag{159}$$

where by direct computation we confirm that the charges occupying $4b$ contribute again contribute a Q^{xy} of $e/2$ in the units of $3R^2$, as they did previously for the m_2^+ mass term (see the text following SEq. (156)), and the charges at $8c$ (*i.e.* those not at $4b$) contribute a *net-zero* Q^{xy}. This is because, within each quadrant, there are two sites in the $8c$ position with opposite charges $\pm e/2$ but with the *same* value for $\sin(2\theta_n)$, because the charges are related by $M_{x\pm y}$, which takes $\theta \rightarrow \pm\pi/2 - \theta$. This implies that *any* set of 0D modes of alternating charge placed at the $8c$ position of $4mm$ will have a net-zero quadrupole moment. We therefore conclude that the m_6^+ term also only provides an $e/2$ quadrupole moment if it is the only nonzero term in $U_{edge}(\theta)$. Furthermore, as all of the terms proportional to $s^2(\theta)$ in SEq. (135) are circular harmonics of the form $\cos(L_z\theta)$ where $L_z = L_z^{QI}$ (SEq. (139)), then we can conclude from SEqs. (157) and (159) that introducing *any* term proportional to $s^2(\theta)$ in $U_{edge}(\theta)$ results in a collection of 0D bound states that decomposes into four modes at the $4b$ position and a remaining multiple of 8 modes at the general position, for which the overall quadrupole moment $Q^{xy} = e/2$.

We also note the previous analysis of the quadrupole moments of the $m_{2,6}^+$ mass terms can also, with minor modifications, be applied to any of the terms in $U_{edge}(\theta)$ proportional to $m^-_{L_z^{QI}}$. Using the transformation $s^1(\theta) \leftrightarrow s^2(\theta)$ in SEqs. (123) to SEq. (150), we can conclude that if the only nonzero mass term in $U_{edge}(\theta)$ is proportional to $s^1(\theta)$, it will be proportional to a circular harmonic of the form $\sin(L_z\theta)$ where $L_z = L_z^{QI}$ (SEq. (139)), and will therefore exhibit a configuration of $2L_z$ 0D states of alternating charges $\pm e/2$ localized on the $r = R$ boundary at:

$$\theta_n^s = n\pi/L_z, n \in \{0, 1, \ldots, 2L_z - 1\}. \tag{160}$$

These states will instead decompose into 4 modes of alternating charges occupying the $4a$ position $(\pm x, 0)$; $(0, \pm x)$ of $4mm$[40] (SFig. 10) and multiples of 8 states of alternating charge occupying the general position. Plugging the coordinates and charges (SEq. (155)) of the (anti)solitions at $4a$, as well as those at $8c$, into SEq. (156):

$$Q^{xy} = \frac{3R^2}{4}\left(\sum_{n\in n_{4a}} q_n \sin(2\theta_n) + \sum_{n\notin n_{4a}} q_n \sin(2\theta_n)\right) = 3R^2\,(0+0)\,. \tag{161}$$

However, we can also define an $x^2 - y^2$ quadrupole moment[99] by rotating SEq. (156) by $\pi/4$:

$$Q^{x^2-y^2} = \frac{3R^2}{4}\sum_n q_n \cos(2\theta_n), \tag{162}$$

for which, using the (anti)soliton locations θ_n^s in SEq. (160) and charge assignments in SEq. (155):

$$Q^{x^2-y^2} = \frac{3R^2}{4}\left(\sum_{n\in n_{4a}} q_n \cos(2\theta_n) + \sum_{n\notin n_{4a}} q_n \cos(2\theta_n)\right) = 3R^2\,(e/2+0)\,. \tag{163}$$

Therefore, as long as there exists a particle-hole symmetry of the form of *either* $s^1(\theta)$ or $s^2(\theta)$, the introduction to a 2D TI of a term in $U_{edge}(\theta)$ proportional to a circular harmonic with $L_z = L_z^{QI} = 2 + 4a$ will result in a distribution of boundary zero modes with an $e/2$ quadrupole moment, be it $Q^{xy} = e/2$ or $Q^{x^2-y^2} = e/2$.

Finally, to demonstrate the robustness of this result, we show that the corner-mode quadrupole moment persists in the presence of multiple nonzero mass terms (both $m^\pm_{2+4a}$) in $U_{edge}(\theta)$, which generically results in the relaxation of all particle-hole symmetries. To accomplish this, we begin by keeping $m_2^+ \neq 0$ and $m^-_{2+4a} = 0$ and perturbatively introduce one of the other m^+_{2+4a} terms in $U_{edge}(\theta)$ (SEq. (135)). The added term, which is proportional to

$m^+_{2+4a}\cos[(2+4a)\theta]s^2(\theta)$, is equal to zero at the four values of θ_n in SEq. (143) where $\cos(2\theta)$ is zero (*i.e.*, the $4b$ Wyckoff position in SFig. 10), as well as at $8a$ additional values of θ that correspond to the $8c$ Wyckoff position in $4mm$. As shown in SEqs. (159) and (163), respectively, any set of alternating solitons and antisolitons occupying the $8c$ position of $4mm$ contribute net-zero Q^{xy} and $Q^{x^2-y^2}$ quadrupole moments. Therefore, the additional m^+_{2+4a} mass term does not affect the existing $e/2$ xy quadrupole moment from the m^+_2 term.

We next, setting all of the m^+_{2+4a} mass terms in SEq. (135) back to zero except for m^+_2, consider the effect of adding one of the terms proportional to $m^-_{2+4a}\sin[(2+4a)\theta]s^1(\theta)$. Adding one of these m^-_{2+4a} terms explicitly breaks particle-hole symmetry (SEq. (123)). At each θ_n in SEq. (143), we calculate the first-order energy correction:

$$\begin{aligned}\Delta_E(\theta_n) &= m^-_{2+4a}\sin[(2+4a)\theta_n]\langle s^1(\theta_n)|_{\lambda(\theta_n)}s^1(\theta_n)|s^1(\theta_n)\rangle_{\lambda(\theta_n)} \\ &= m^-_{2+4a}\lambda(\theta_n)\sin[(2+4a)\theta_n] \\ &= m^-_{2+4a}(-1)^n(-1)^n(-1)^a \\ &= m^-_{2+4a}(-1)^a,\end{aligned} \tag{164}$$

where we have exploited that $\lambda(\theta_n) = (-1)^n$ (SEq. (153)) and that:

$$\sin[(2+4a)\theta_n] = (-1)^n(-1)^a, \tag{165}$$

because $\sin(L^{QI}_z\theta)$ (SEq. (139)) is always at an extremum at a zero θ_n of $\cos 2\theta$, which are coincident with the zeroes of $\cos(L^{QI}_z\theta)$. SEq. (164) indicates that, beginning with m^+_2 as the only nonzero mass term in SEq. (135), adding any of the m^-_{2+4a} mass terms causes all four (anti)solitons at θ_n to shift together in energy in a $4mm$-symmetric manner, such that the spectrum no longer exhibits zero modes (SFig. 9(b)). Nevertheless, if the system is half-filled, then within a perturbative range in m^-_{2+4a}, half of the 0D modes will still be occupied under softly broken C_{4z} symmetry, resulting in a charge distribution with an $e/2$ quadrupole moment (SEq. (156)). We therefore conclude that the $4mm$-symmetric $p-d$-hybridized TI highlighted in this section can gap into a QI because it admits the presence of a bulk magnetic term with a nonzero edge projection and which is proportional to a circular harmonic with $L_z = L^{QI}_z$ (SEq. (139)). The preservation of the quantized quadrupole moment away from the particle-hole-symmetric limit is in agreement with the conclusions of Wilczek in SRef. 128, in which it is stated that when the masses at Jackiw-Rebbi domain walls are made complex (*i.e.* more than one Pauli matrix is present), there will no longer generically be zero-energy bound states, but the overall distribution of bound charge will remain preserved and reflect the accumulated phase in the complex mass over the domain wall. This can also be understood from a field-theory perspective by considering the Goldstone-Wilczek formulation[127,129].

8. Gapping the Edge Modes of a 2D TI into an Magnetic Insulator with Zero Quadrupole Moment

We will now show that a 2D TI in layer group $p4/mmm1'$, formed of s and p_z orbitals at the $1a$ position (Fig. 2(a) of the main text), gaps trivially in the presence of magnetism that preserves the symmetries of wallpaper group $p4m$, unlike the previous $p-d$-hybridized TI in SN 7. We will see that this difference arises because, unlike in the previous $p-d$-hybridized TI (SEq. (95)), the bulk C_{4z} eigenvalues of the occupied bands of an $s-p$-hybridized TI do not match those of a QI (SN 3 and 5). The $k\cdot p$ Hamiltonian of a 2D $s-p$-hybridized TI is:

$$\mathcal{H}_\Gamma(\mathbf{k}) = m\tau^z + vk_x\tau^x\sigma^y - vk_y\tau^x\sigma^x, \tag{166}$$

where we have again suppressed factors of the lattice constants $a_{x,y} = a$. We note that SEq. (95) is almost identical to the previous $k\cdot p$ Hamiltonian of a $p-d$-hybridized TI (SEq. (95)); the only difference between the two equations is the minus sign on the k_y velocity term. In the notation of SEq. (97), $\mathcal{H}_\Gamma(k_x,k_y)$ (SEq. (166)) transforms in the symmetry representation given by:

$$\begin{aligned}&\mathcal{T}:\ \sigma^y\mathcal{H}^*_\Gamma(-k_x,-k_y)\sigma^y,\ M_z:\ \tau^z\sigma^z\mathcal{H}_\Gamma(k_x,k_y)\tau^z\sigma^z,\ M_x:\ \sigma^x\mathcal{H}_\Gamma(-k_x,k_y)\sigma^x, \\ &M_y:\ \sigma^y\mathcal{H}_\Gamma(k_x,-k_y)\sigma^y,\ C_{4z}:\ \left(\frac{\mathbb{1}_\sigma - i\sigma^z}{\sqrt{2}}\right)\mathcal{H}_\Gamma(k_y,-k_x)\left(\frac{\mathbb{1}_\sigma + i\sigma^z}{\sqrt{2}}\right),\end{aligned} \tag{167}$$

where $\mathbb{1}_\sigma$ is the 2×2 identity in σ space. SEq. (167) implies an inversion symmetry $\mathcal{I} = M_xM_yM_z$ that transforms $\mathcal{H}_\Gamma(k_x,k_y)$ under the representation:

$$\mathcal{I}:\ \tau^z\mathcal{H}_\Gamma(-k_x,-k_y)\tau^z. \tag{168}$$

The presence of τ^z in M_z and $\mathcal{I}$, and the absence of τ^z in C_{4z} in SEqs. (167) and (168) reflect that $\mathcal{H}_\Gamma(\mathbf{k})$ describes a 2D TI formed of hybridized s and p_z orbitals, as s (p_z) orbitals are even (odd) under M_z and $\mathcal{I}$ and even under C_{4z}. Specifically, unlike previously in the symmetry representation of the $p-d$-hybridized TI in SEq. (98) (and like in the symmetry representation of the $k\cdot p$ theory of the $s-d$-hybridized TCI phase of SEq. (1) that will be analyzed in SN 9), there is no prefactor of τ^z in the representation of C_{4z} in SEq. (167), because the valence and conduction bands of an $s-p_z$-hybridized TI have the same C_{4z} eigenvalues (though they have different parity eigenvalues). $\mathcal{H}_\Gamma(\mathbf{k})$ also exhibits the same pair of unitary particle-hole symmetries as previously in SEqs. (100) and (101):

$$\{\mathcal{H}_\Gamma(\mathbf{k}),\Pi\}=0,\ \Pi=\tau^y, \tag{169}$$

and:

$$\{\mathcal{H}_\Gamma(\mathbf{k}),\tilde{\Pi}\}=0,\ \tilde{\Pi}=\tau^x\sigma^z, \tag{170}$$

which we will again relax in future steps in this calculation, as we did previously in in SN 7. We note that the choice of which particle-hole representation to label with a tilde is arbitrary; the choice of $\Pi=\tau^y$ in SEq. (169) is only distinct from the previous choice in SEq. (100) to simplify notation in expressions that will arise later in this section.

As mentioned previously, SEq. (166), is nearly identical to the $k\cdot p$ Hamiltonian of a $p_z-d_{x^2-y^2}$-hybridized TI analyzed in SN 7 (it only differs from SEq. (95) by the minus sign of $vk_y\tau^x\sigma^x$). Nevertheless, the two Hamiltonians cannot be transformed into each other by a unitary transformation that preserves the handedness of the spin and momentum sectors of rotations about the z axis. Specifically, if we require that C_{4z} is defined as the transformation:

$$k_x\rightarrow k_y,\ k_y\rightarrow -k_x,\ \sigma^x\rightarrow\sigma^y,\ \sigma^y\rightarrow -\sigma^x, \tag{171}$$

then the unitary transformation $U=\sigma^y$ that converts SEq. (166) into SEq. (95) also changes the sign of σ^z in C_{4z} in SEq. (167). Under this transformation, C_{4z} would continue to rotate momentum counterclockwise as specified in SEq. (171), however it would now rotate the spins *clockwise* about σ^z. Therefore we cannot simultaneously transform SEq. (166) into SEq. (95) while preserving a physical definition of C_{4z}. This can also be understood by recognizing that in SEq. (166), unlike in SEq. (95), both the valence and conduction bands have the same complex-conjugate pairs of C_{4z} eigenvalues (they can still be inverted because they possess different inversion eigenvalues[117]). We will show in this section that, unlike previously in SN 7, because the valence and conduction bands of the $s-p_z$-hybridized TI in SEq. (166) exhibit the same C_{4z} eigenvalues, then, when SEq. (166) is terminated in a disc geometry and its edge states are gapped with $p4m$-symmetric magnetism, the resulting 0D bound states exhibit a topologically trivial quadrupole moment of $Q^{xy}\bmod e=Q^{x^2-y^2}\bmod e=0$.

We again Fourier transform $k_{x,y}\rightarrow -i\partial_{x,y}$ and search for Jackiw-Rebbi bound states on the boundary of a circular region of a large radius $R\gg a$ for which $\text{sgn}[m(r)]=\text{sgn}(r-R)$. We note that, because SEqs. (95) and (166) both describe 2D TIs, then the first (bulk-to-edge) Jackiw-Rebbi calculation performed to obtain the edge zero modes will be identical in this section to the previous calculation performed SN 7; spectral differences between the two TIs will only begin to manifest when we gap the edge spectrum with $p4m$-symmetric magnetism.

The Hamiltonian in SEq. (166), when converted to polar coordinates using SEq. (102), takes the same form as SEq. (103):

$$\mathcal{H}_\Gamma(r,\theta)=m(r)\tau^z-iv\tau^x\left[\bar{\sigma}^1(\theta)\partial_r+\frac{1}{r}\bar{\sigma}^2(\theta)\partial_\theta\right], \tag{172}$$

but through a different the canonical transformation than SEq. (104):

$$\begin{gathered}\bar{\sigma}^1(\theta)=\sin(\theta)\sigma^x-\cos(\theta)\sigma^y=\begin{pmatrix}0 & ie^{-i\theta}\\ -ie^{i\theta} & 0\end{pmatrix},\\ \bar{\sigma}^2(\theta)=\cos(\theta)\sigma^x+\sin(\theta)\sigma^y=\begin{pmatrix}0 & e^{-i\theta}\\ e^{i\theta} & 0\end{pmatrix},\\ \{\bar{\sigma}^1(\theta),\bar{\sigma}^2(\theta)\}=0,\ \bar{\sigma}^1(\theta)\bar{\sigma}^2(\theta)=i\sigma^z.\end{gathered} \tag{173}$$

In the notation of SEq. (106), $\mathcal{H}_\Gamma(r,\theta)$ transforms in the symmetry representation given by:

$$\begin{gathered}\mathcal{T}:\ \sigma^y\mathcal{H}^*_\Gamma(r,\theta)\sigma^y,\ M_z:\ \tau^z\sigma^z\mathcal{H}_\Gamma(r,\theta)\tau^z\sigma^z,\ M_x:\ \sigma^x\mathcal{H}_\Gamma(r,\pi-\theta)\sigma^x,\\ M_y:\ \sigma^y\mathcal{H}_\Gamma(r,-\theta)\sigma^y,\ C_{4z}:\ \left(\frac{\mathbb{1}_\sigma-i\sigma^z}{\sqrt{2}}\right)\mathcal{H}_\Gamma(r,\theta+\pi/2)\left(\frac{\mathbb{1}_\sigma+i\sigma^z}{\sqrt{2}}\right),\\ \mathcal{I}:\ \tau^z\mathcal{H}_\Gamma(r,\theta+\pi)\tau^z,\end{gathered} \tag{174}$$

and the particle-hole symmetries remain the same as previously in SEqs. (169) and (170):

$$\{\mathcal{H}_\Gamma(r,\theta),\Pi\}=0,\ \Pi=\tau^y. \tag{175}$$

and:

$$\{\mathcal{H}_\Gamma(r,\theta),\tilde{\Pi}\}=0,\ \tilde{\Pi}=\tau^x\sigma^z. \tag{176}$$

Once again, we form a Jackiw-Rebbi problem for the bound states of SEq. (172):

$$\mathcal{H}_\Gamma(r,\theta)|\psi(r,\theta)\rangle=0, \tag{177}$$

which, following the procedure in SEqs. (110), (111), and (112), we simplify to:

$$\left[m(r)\tau^y+v\bar{\sigma}^1(\theta)\partial_r\right]|\psi(r,\theta)\rangle\approx 0, \tag{178}$$

by left-multiplying by τ^x, canceling a factor of $-i$, and recognizing that $(1/r)\sim(1/R)\to 0$ for a bound state localized at the radius R of a large circle with $R\gg a$ where a is the lattice spacing. We solve SEq. (178) by left-multiplying by τ^y and integrating:

$$|\psi_{1,2}(r,\theta)\rangle=\frac{1}{\sqrt{N}}e^{-\frac{1}{v}\int_R^r m(r')dr'}|\tau^y_\pm\bar{\sigma}^1_\pm(\theta)\rangle=\mathcal{R}(r)|\tau^y_\pm\bar{\sigma}^1_\pm(\theta)\rangle, \tag{179}$$

where:

$$|\tau^y_\pm\bar{\sigma}^1_\pm(\theta)\rangle=|\tau^y_\pm\rangle\otimes|\bar{\sigma}^1_\pm(\theta)\rangle, \tag{180}$$

where $|\tau^i_\pm,\sigma^j_\pm\rangle$ are the eigenstates with eigenvalues ±1 of the 2×2 Pauli matrices τ^i and σ^j.

As in SN 7, we will find that the symmetries of the edge Hamiltonian appear in a more familiar form in the rotated basis:

$$|\bar{\phi}_1(r,\theta)\rangle=\frac{-ie^{i\theta}}{\sqrt{2}}\left(|\psi_1(r,\theta)\rangle-|\psi_2(r,\theta)\rangle\right)=\frac{\mathcal{R}(r)}{\sqrt{2}}\begin{pmatrix}0\\-e^{i\theta}\\1\\0\end{pmatrix}$$

$$|\bar{\phi}_2(r,\theta)\rangle=\frac{1}{\sqrt{2}}\left(|\psi_1(r,\theta)\rangle+|\psi_2(r,\theta)\rangle\right)=\frac{\mathcal{R}(r)}{\sqrt{2}}\begin{pmatrix}e^{-i\theta}\\0\\0\\1\end{pmatrix}, \tag{181}$$

where, as previously in SEq. (117), we are free to make this transformation because $|\bar{\phi}_{1,2}(\theta)\rangle$ are degenerate (zero modes) at all values of θ at this stage of the calculation.

To leading order, SEq. (179) indicates that there are two nondispersing zero modes localized on the boundary of the circular domain wall with radius R, or close to the region where $m(r)=0$. Again, perturbatively restoring the angular velocity term $-i(v/r)\tau^x\bar{\sigma}^2(\theta)\partial_\theta$ and projecting into the basis of the edge states $|\bar{\phi}_{1,2}(r,\theta)\rangle$ using SEq. (118), we realize the edge Hamiltonian:

$$\mathcal{H}^{TI}_{edge}(\theta)=-\frac{v}{R}\left(\frac{1}{2}\mathbb{1}_s+is^z\partial_\theta\right), \tag{182}$$

where s^z is a Pauli matrix and $\mathbb{1}_s$ is the identity matrix in the 2×2 basis of $|\bar{\phi}_{1,2}(r,\theta)\rangle$, and where, as previously in SEq. (118), the action of ∂_θ on the edge states $|\bar{\phi}_{1,2}(r,\theta)\rangle$ results in the presence of a gauge-dependent constant term, which is $-(v/2R)\mathbb{1}_s$ for the choice of gauge in SEq. (181). SEq. (182), the edge Hamiltonian of a $s-p$-hybridized TI, is identical to SEq. (118), the previous edge Hamiltonian of a $p-d$-hybridized TI (up to an overall minus sign).

In the basis of $|\bar{\phi}_{1,2}(r,\theta)\rangle$, the symmetries from SEq. (174) transform $\mathcal{H}^{TI}_{edge}(\theta)$ under the representation:

$$\begin{aligned}&\mathcal{T}:\ s^y(\mathcal{H}^{TI}_{edge}(\theta))^*s^y,\ M_z:\ s^z\mathcal{H}^{TI}_{edge}(\theta)s^z,\ M_x:\ s^x\mathcal{H}^{TI}_{edge}(\pi-\theta)s^x,\\&M_y:\ s^y\mathcal{H}^{TI}_{edge}(-\theta)s^y,\ C_{4z}:\ \left(\frac{\mathbb{1}_s-is^z}{\sqrt{2}}\right)\mathcal{H}^{TI}_{edge}(\theta+\pi/2)\left(\frac{\mathbb{1}_s+is^z}{\sqrt{2}}\right),\\&\mathcal{I}:\ \mathcal{H}^{TI}_{edge}(\theta+\pi),\end{aligned} \tag{183}$$

which is *identical* to the previous edge symmetry representation in SEq. (122). However, because the bulk representations of C_{4z} are different in SEqs. (107) and (174), we will see that when $p4m$-symmetric magnetic masses are added to gap the edge states (SEq. (182)), a different configuration of (quadrupole-trivial) zero modes emerges in the $s-p$-hybridized case than previously appeared for the $p-d$-hybridized 2D TI in SN 7. Specifically, while the *bulk* symmetry representations of C_{4z} are different in the $p-d$-hybridized 2D TI in SEq. (107) and in the $s-p$-hybridized 2D TI in SEq. (174), that difference does not carry over into the symmetry representation of the 2×2 *edge* Hamiltonians of the two TIs (SEqs. (122) and (183), respectively). This occurs because the edge Hamiltonian of any 2D TI characterizes a twofold, spin-1/2 fermion in 1D[119], and because there is, up to unitarily equivalent expressions, only one way to represent spinful C_{4z} symmetry in the 2×2 basis of spin-1/2 Pauli matrices σ^i (as opposed to in the 4×4 basis of orbital τ^i and spin σ^i matrices of the bulk Hamiltonians in SEqs. (95) and (166), in which there are two inequivalent ways to represent C_{4z}). Nevertheless and crucially, as we will shortly see, the corner spectrum of a 2D TI gapped with $p4m$-symmetric magnetism (*i.e.* the number and quadrupole moment of the 0D modes on its boundary), depends on the *bulk* representation of C_{4z}, and not the edge representation, and thus still distinguishes between $p-d$-hybridized TIs and $s-p$-hybridized TIs.

As previously (SEq. (123)), in the basis of $|\bar{\phi}_{1,2}(r,\theta)\rangle$, the particle-hole symmetries from SEqs. (175) and (176) take θ-dependent forms:

$$\Pi(\theta) = \bar{s}^2(\theta),\ \tilde{\Pi}(\theta) = \bar{s}^1(\theta), \tag{184}$$

where:

$$\begin{aligned}
\bar{s}^1(\theta) &= \cos(\theta)s^x + \sin(\theta)s^y = \begin{pmatrix} 0 & e^{-i\theta} \\ e^{i\theta} & 0 \end{pmatrix}, \\
\bar{s}^2(\theta) &= \sin(\theta)s^x - \cos(\theta)s^y = \begin{pmatrix} 0 & ie^{-i\theta} \\ -ie^{i\theta} & 0 \end{pmatrix}, \\
\{\bar{s}^1(\theta), \bar{s}^2(\theta)\} &= 0,\ \bar{s}^1(\theta)\bar{s}^2(\theta) = -is^z,
\end{aligned} \tag{185}$$

and as previously in SEqs. (125) through (128), the θ dependence of $\Pi(\theta)$ and $\tilde{\Pi}(\theta)$ in SEq. (184) maintain both representations of particle-hole symmetry through the relations:

$$\partial_\theta \bar{s}^1(\theta) = -\bar{s}^2(\theta) + \bar{s}^1(\theta)\partial_\theta,\ \partial_\theta \bar{s}^2(\theta) = \bar{s}^1(\theta) + \bar{s}^2(\theta)\partial_\theta. \tag{186}$$

We now add magnetic terms that preserve the symmetries of $p4m$. Here, there are manifest differences between these terms and the corresponding terms in SN 7. We again work in the long-wavelength limit and propose the most general r-independent bulk mass term $U(\theta)$ in the form of SEq. (131), whose terms individually anticommute with the Dirac matrix coefficients of the mass ($m(r)\tau^z$) and angular velocity ($-i(v/r)\tau^x\bar{\sigma}^2(\theta)\partial_\theta$) terms in SEq. (172) while respecting the bulk representations of $M_{x,y}$ and C_{4z} in SEq. (174). We express $U(\theta)$ as a sum of terms proportional to circular harmonics of increasing L_z (SEq. (132)):

$$\begin{aligned}
U(\theta) = \tau^x\sigma^z &\left[m_4^- \sin(4\theta) + m_8^- \sin(8\theta) + m_{12}^- \sin(12\theta) + \dots\right] \\
&+ \tau^y \left[m_0^+ + m_4^+\cos(4\theta) + m_8^+\cos(8\theta) + \dots\right] \\
&+ \tau^x\bar{\sigma}^1(\theta)\left[\tilde{m}_0^+ + \tilde{m}_4^+\cos(4\theta) + \tilde{m}_8^+\cos(8\theta) + \dots\right] \\
&+ \tau^y\bar{\sigma}^2(\theta)\left[\tilde{m}_4^-\sin(4\theta) + \tilde{m}_8^-\sin(8\theta) + \tilde{m}_{12}^-\sin(12\theta) + \dots\right],
\end{aligned} \tag{187}$$

and observe that the terms group into circular harmonics with $\Delta L_z = 4$ multiplied by one of four 4×4 matrices. We then, as was done to generate SEq. (135), project $U(\theta)$ into the basis of the edge modes $|\bar{\phi}_{1,2}(r,\theta)\rangle$:

$$\begin{aligned}
U_{edge,ij}(\theta) &= \langle\bar{\phi}_i(r,\theta)|U(\theta)|\bar{\phi}_j(r,\theta)\rangle \\
&= \sum_{L_z,\mu} m^\mu_{L_z}\langle\bar{\phi}_i(r,\theta)|\Gamma^{L_z,\mu}|\bar{\phi}_j(r,\theta)\rangle f^\mu_{L_z}(\theta),
\end{aligned} \tag{188}$$

$$\begin{aligned}
U_{edge}(\theta) = \bar{s}^1(\theta)&\left[m_4^-\sin(4\theta) + m_8^-\sin(8\theta) + m_{12}^-\sin(12\theta) + \dots\right] \\
&+ \bar{s}^2(\theta)\left[m_0^+ + m_4^+\cos(4\theta) + m_8^+\cos(8\theta) + \dots\right],
\end{aligned} \tag{189}$$

where, in agreement with SEqs. (136), (137), and (138), the terms in $U(\theta)$ that commute with $\tau^y\bar{\sigma}^1(\theta)$ ($m^\pm_{4a}$) have nonzero edge projections, whereas the terms that anticommute with $\tau^y\bar{\sigma}^1(\theta)$ ($\tilde{m}^\pm_{4a}$) project to zero in $U_{edge}(\theta)$. The nonzero terms in $U_{edge}(\theta)$ break $\mathcal{I}$, M_z, and $\mathcal{T}$ symmetries, while respecting the combined magnetic symmetries $\mathcal{I}\times\mathcal{T}$

and $M_z \times \mathcal{T}$ (SEq. (183)) (though, as shown in SN 3, the antiunitary magnetic symmetries $\mathcal{I} \times \mathcal{T}$ and $M_z \times \mathcal{T}$, while symmetries of the original QI model in SRef. 26, are not necessary to protect the QI phase). As previously in SN. 7, half of the mass terms in SEq. (189) only respect one of the particle-hole symmetries in SEq. (184) (the $m^-_{L_z}$ terms and $\Pi(\theta) = \bar{s}^2(\theta)$) and the other terms only respect the other particle-hole symmetry (the $m^+_{L_z}$ terms and $\tilde{\Pi}(\theta) = \bar{s}^1(\theta)$). Therefore, when mass terms from both the $m^{\pm}_{L_z}$ sets are nonzero, particle-hole symmetry is broken.

Unlike previously in SN 7, SEq. (189) indicates that, for the $s-p$-hybridized TI in this section, bulk $p4m$-preserving magnetism can only open an edge gap with:

$$L_z^{NI} = 4a, \;\; a \in \mathbb{Z}, \tag{190}$$

where the m^-_4 and m^+_0 terms in SEq. (189), in particular, are proportional to the circular harmonics of $g_{xy(x^2-y^2)}$ and s orbitals, respectively[99,122–124]. This result is markedly different than the previous conclusion in SN 7 that, for a $p-d$-hybridized TI gapped with $p4m$-preserving magnetism, $L_z^{QI} = 2 + 4a$ (SEq. (139)). We label SEq. (190) with the typical abbreviation for a normal (trivial) insulator (NI) because, as we will shortly demonstrate, it implies that $p4m$-preserving magnetism can only gap an $s-p_z$-hybridized TI into a magnetic insulator with a trivial quadrupole moment.

We define the edge Hamiltonian to be:

$$\mathcal{H}^{NI}_{edge}(\theta) = \mathcal{H}^{TI}_{edge}(\theta) + U_{edge}(\theta). \tag{191}$$

If we truncate $U_{edge}(\theta)$ (SEq. (189)) to its leading m^-_4 and m^+_0 terms, $\mathcal{H}^{NI}_{edge}(\theta)$ exhibits a gap in the long-wavelength limit of:

$$\Delta(\theta) = 2\sqrt{(m^-_4)^2 \sin^2(4\theta) + (m^+_0)^2}. \tag{192}$$

We can understand why $\mathcal{H}_{edge}(\theta)$ does not exhibit a topological quadrupole moment from several perspectives. First, we can begin in the limit where m^+_0 is the only nonzero mass term in SEq. (189). In this limit, $\mathcal{H}_{edge}(\theta)$ is simply gapped at all values of θ, as opposed to SEq. (142), which binds 0D modes when either $m^{\pm}_{L_z}$ is nonzero. As shown previously in SN 7, an edge (and bulk) quadrupole moment is only topological if its value remains fixed (modulo e and up to a choice of orientation, as shown in the text surrounding SEq. (162)) in the presence of any linear combination of symmetry-allowed mass terms. If there are no edge solitons at all, then $Q^{xy} = Q^{x^2-y^2} = 0$ trivially.

We can further understand the absence of a topological quadrupole moment by next beginning in the limit that m^-_4 is the only nonzero mass in SEq. (189), and then perturbatively reintroducing other mass terms. When m^-_4 is the only nonzero mass, the system is particle-hole symmetric (SEq. (184)) and the spectrum is gapless at eight values of θ:

$$\theta_n = n\pi/4, \;\text{ for } n \in \{0, 2, \ldots, 7\}. \tag{193}$$

We search for zero-energy bound states at each of the θ_n by forming the Jackiw-Rebbi problem:

$$\left[-\frac{v}{R}\left(\frac{1}{2}\mathbb{1}_s + is^z\partial_\theta\right) + m^-_4 \sin(4\theta)\bar{s}^1(\theta)\right]|\tilde{\Theta}(\theta)\rangle = 0, \tag{194}$$

which we simplify by using the identical procedure in SEqs. (144) to (150) to remove the constant curvature term $-(v/2R)\mathbb{1}_s$ and then expanding θ in a small range ϵ around each angle θ_n in SEq. (193):

$$\left[-i\frac{v}{R}s^z\partial_\epsilon + (-1)^n m_\theta \epsilon \bar{s}^1(\theta_n)\right]|\Theta(\theta_n, \epsilon)\rangle = 0, \tag{195}$$

where $m_\theta = 4m^-_4$. We solve SEq. (195) by left-multiplying by $\bar{s}^1(\theta)$ and integrating:

$$|\Theta(\theta_n, \epsilon)\rangle = \frac{1}{\sqrt{N}}e^{-\frac{m_\theta R}{2v}\epsilon^2}|\bar{s}^2(\theta_n)\rangle_{\lambda(\theta_n)}, \tag{196}$$

where we have simplified by exploiting that $|\bar{s}^2(\theta_n)\rangle_{\lambda(\theta_n)}$ is the eigenstate of $\bar{s}^2(\theta_n)$ with eigenvalue:

$$\lambda(\theta_n) = (-1)^n. \tag{197}$$

We therefore find that when m^-_4 is the only nonzero mass, there are eight Jackiw-Rebbi zero modes[116] bound to zeroes of $\sin(4\theta)$, in agreement with the result obtained in SEq. (160) for a general edge mass term proportional to a circular harmonic $\sin(L_z\theta)$. These eight modes are alternately, in increasing θ, solitons with charge $e/2$ and antisolitons with

charge[21,22,126,127] $-e/2$, a charge distribution that can still be summarized using SEq. (155) (up to an overall sign reflecting an offset in indexing between the (anti)solitons in this and the previous problem (SN 7)). The four solitons (antisolitons) therefore occupy the $4a$ ($4b$) Wyckoff position of point group[40] $4mm$ (SFig. 10). For this arrangement of charges,

$$n_{4a} = \{0, 2, 4, 6\},\ n_{4b} = \{1, 3, 5, 7\}, \tag{198}$$

in SEq. (193). Using SEqs. (155), (156), (162) to calculate the quadrupole moments of these (anti)solitions, both:

$$Q^{xy} = \frac{3R^2e}{8}\left(\sum_{n\in n_{4a}} \sin(2\theta_n) - \sum_{n\in n_{4b}} \sin(2\theta_n)\right) = 0, \tag{199}$$

and:

$$Q^{x^2-y^2} = \frac{3R^2e}{8}\left(\sum_{n\in n_{4a}} \cos(2\theta_n) - \sum_{n\in n_{4b}} \cos(2\theta_n)\right) = 0. \tag{200}$$

The overall quadrupole moment is therefore topologically trivial.

Furthermore, and crucially, we can also show that any linear combination of nonzero mass terms in SEq. (189) contributes a trivial quadrupole moment (modulo e). First, using the results of SEqs. (160) through (163), we deduce that if *any single* term in $U_{edge}(\theta)$ is nonzero while the other terms are zero, the resulting configuration of zero modes will consist of (up to the overall sign of the charge) four solitions at $4a$, four antisolitons at $4b$, and multiples of eight charges at the general position $8c$ (SFig. 10). As SEqs. (159) and (163) show that any set of charges of alternating sign occupying the general position has $Q^{xy} = Q^{x^2-y^2} = 0$, then this, along with SEqs. (199) and (200) implies, that *all* of the terms in $U_{edge}(\theta)$ (SEq. (189)) individually lead to a configuration of zero modes with trivial quadrupole moments.

We can now explore the effects of adding other mass terms in $U_{edge}(\theta)$ (SEq. (189)) while keeping $m_4^- \neq 0$. First, we consider turning on one of the other mass terms proportional to $m_{4a}^- \sin[(4a)\theta]\bar{s}^1(\theta)$. This term will be zero at all of the θ_n in SEq. (193) where $\sin(4\theta)$ is zero, as well as at $8(a-1)$ additional values of θ that correspond to the $8c$ Wyckoff position in $4mm$ (SFig. 10). Unlike in the previous discussion in the text following SEq. (159), in which the 0D modes at $8c$ had alternating charges, in this case, the $8(a-1)$ modes at $8c$ will either *all* be solitons with charge $e/2$ or antisolitons with charge $-e/2$. However, by direct computation using SEqs. (159) and (163), we confirm that any set of solitons or antisolitons occupying the $8c$ position of $4mm$ contributes net-zero Q^{xy} and $Q^{x^2-y^2}$ quadrupole moments, and therefore the overall quadrupole moment remains trivial.

We next consider, beginning in the limit that m_4^- is the only nonzero mass term in SEq. (189), the effect of perturbatively introducing a term proportional to $m_{4a}^+ \cos[(4a)\theta]\bar{s}^2(\theta)$. Adding one of these m_{4a}^+ terms explicitly breaks particle-hole symmetry (SEq. (184)). At each θ_n in SEq. (193), we calculate the first-order energy correction:

$$\begin{aligned}\Delta_E(\theta_n) &= m_{4a}^+ \cos[(4a)\theta_n]\langle \bar{s}^2(\theta_n)|_{\lambda(\theta_n)}\bar{s}^2(\theta_n)|\bar{s}^2(\theta_n)\rangle_{\lambda(\theta_n)} \\ &= m_{4a}^+\lambda(\theta_n)\cos[(4a)\theta_n] \\ &= m_{4a}^+(-1)^n(-1)^{na} \\ &= m_{4a}^+[(-1)^n]^{(a+1)},\end{aligned} \tag{201}$$

where we have exploited that $\lambda(\theta_n) = (-1)^n$ (SEq. (197)) and that:

$$\cos[(4a)\theta_n] = (-1)^{na}, \tag{202}$$

because $\cos[(4a)\theta]$ is always at an extremum at a zero θ_n of $\sin(4\theta)$, which are coincident with the zeroes of $\sin(L_z\theta)$ for $L_z = L_z^{NI}$ (SEq. (190)). SEq. (201) implies that if a is even, then the eight 0D states in SFig. 11(a) will split into two sets of four states in a $4mm$-symmetric manner, and that if a is odd, then all eight states will shift together in energy by the same amount. For example, if $a = 0$ in SEq. (201), then the sign of $\Delta_E(\theta_n)$ will alternate with increasing θ, such that four of the modes move down in energy and four of the modes move up in energy while the Fermi level stays in the center (SFig. 11). As this leads to all four of the lower (upper) 0D modes at either the $4a$ or $4b$ ($4b$ or $4a$) position being occupied (unoccupied) (SFig. 10), the particle-hole-broken system continues to exhibit a trivial quadrupole moment at half filling (SEqs. (199) and (200)). Because mass terms proportional to m_{4a}^+ in SEq. (189) with both odd and even values of a are allowed by symmetry, then generically, the eight corner states in SFig. 11(a) will always appear split into groups of four states with trivial quadrupole moments.

We therefore conclude that the $p4m$-symmetric $s-p_z$-hybridized TI highlighted in this section *cannot* gap into a QI because it *does not* admit a bulk magnetic mass term that has a nonzero edge projection and is proportional

to a circular harmonic with $L_z = L_z^{QI} = 2 + 4a$ (SEq. (139)). This occurs precisely because the valence and conduction bands of the $k\cdot p$ Hamiltonian (SEq. (166)) have the same complex-conjugate pairs of C_{4z} eigenvalues, which forces all possible $4mm$-symmetric magnetic mass terms (SEq. (187)) to be proportional to circular harmonics with $L_z = L_z^{NI} = 4a$ (SEq. (190)), which we have shown to generate (anti)solition configurations with trivial quadrupole moments (SEqs. (199) and (200)).

9. Gapping the Edge Modes of a 2D TCI with $C_{M_z} = 2$ with Quadrupolar Magnetism

In this section, we show that the specific 2D TCI[32,44] with mirror Chern number $C_{M_z} = 2$ in layer group $p4/mmm1'$ highlighted in this work (SEq. (1)), equivalent to the Hamiltonian of the $k_z = 0$ plane of $\mathcal{H}_{H2}(\mathbf{k})$ in SEq. (6), gaps into a QI in the presence of magnetism that preserves wallpaper group $p4m$ while breaking M_z and $\mathcal{T}$. We further schematically and numerically show that this TCI gaps into a fragile phase with fractionally charged, quarter-filled (or quarter-empty) Kramers pairs of corner modes under a $p4m$-preserving potential that breaks M_z while preserving $\mathcal{T}$. It is important to note that *not every* 2D TCI can gap to realize fractionally charged 0D states; only TCIs with occupied bands with the same bulk C_{4z} eigenvalues as those of a QI-nontrivial obstructed atomic limit (SN 3 and 5) can gap into a QI or fragile TI that exhibits the same corner charges (modulo e) as the QI model introduced in SRef. 26. We also again emphasize that, as stated in the text before SEq. (172), Q^{xy} and $Q^{x^2-y^2}$ are only strictly calculable through SEqs. (156) and (162) for a $C_{M_z} = 2$ TCI when M_z symmetry is relaxed. Therefore, as was done previously for the TIs in SN 7 and 8, we will show in this section that by counting the C_{4z} eigenvalues of the occupied bands of a 2D TCI, we can still determine the quadrupole moment that it will exhibit (modulo e) when M_z is relaxed.

First, we note that the 2D TCI phase highlighted in this work is in this sense distinct from other previous examples of spinful TCIs[42,44]. Whereas the mirror Chern number $C_{M_z} = 2$ in many previously highlighted TCI phases, such as those in (monolayers[43] and mirror-symmetric planes in 3D crystals[42,44] of) SnTe[42], originated from band-inversion between states with opposite parity eigenvalues at two C_{4z}-related TRIM points (*e.g.* $(k_x, k_y) = (\pi, 0)$ and $(0, \pi)$), in the TCI phase of SEq. (1), the bulk topology instead originates from band inversion at a *single*, C_{4z}-invariant TRIM point between states with the same parity eigenvalues and different C_{4z} eigenvalues (SEq. (94)). In both TCIs, the band within each M_z sector undergoes a direct transition from exhibiting Chern number $C = 0$ to $C = \pm 2$. However, in our model (SEq. (1)), this change in Chern number $\Delta C = \pm 2$ is driven by a change in the occupied C_{4z} eigenvalues at Γ ($(k_x, k_y) = (0, 0)$), *without a corresponding change in the occupied parity eigenvalues* at Γ, or at any other TRIM point. This allows our model to realize a transition from a trivial insulator to a $C_{M_z} = 2$ TCI while still exhibing the same C_{4z} eigenvalues as a QI (as well as the same C_{4z} eigenvalues as the 2D $p - d$-hybridized TI highlighted in SN 7). Therefore, like the previous $p - d$-hybridized TI, when we break M_z and $\mathcal{T}$ symmetries while keeping $p4m$, we will see that the 2D TCI phase of SEq. (1) gaps into a QI. However, as we will also see in this section, unlike for the previous $p - d$-hybridized TI in SN 7, we can also realize a corner-mode phase with a nontrivial quadrupole moment *without* breaking $\mathcal{T}$. Specifically, we can gap the edge states of the TCI phase of SEq. (1) by breaking M_z while keeping $\mathcal{T}$ symmetry, which we will see results in an insulator (which is fragile, and not Wannierizable like the QI (SN 3 and 4)) that also exhibits corner modes for which Q^{xy} mod $e = e/2$ or $Q^{x^2-y^2}$ mod $e = e/2$.

We begin by expanding SEq. (1) about the Γ point to quadratic order in $\mathbf{k}$:

$$\mathcal{H}_\Gamma(k_x, k_y) = [v_m + t(2 - \frac{1}{2}(k_x^2 + k_y^2))]\tau^z + C_1(k_x^2 - k_y^2)\tau^x + 2C_2 k_x k_y \tau^y \sigma^z, \tag{203}$$

where we have set $t_{PH} = 0$ and:

$$t = t_1,\ C_1 = -t_2/2,\ C_2 = v_s/2. \tag{204}$$

In the notation of SEq. (97), $\mathcal{H}_\Gamma(k_x, k_y)$ (SEq. (203)) transforms in the symmetry representation given by:

$$\begin{aligned} &\mathcal{T}:\ \sigma^y \mathcal{H}_\Gamma^*(-k_x, -k_y)\sigma^y,\ M_z:\ \sigma^z \mathcal{H}_\Gamma(k_x, k_y)\sigma^z,\ M_x:\ \sigma^x \mathcal{H}_\Gamma(-k_x, k_y)\sigma^x, \\ &M_y:\ \sigma^y \mathcal{H}_\Gamma(k_x, -k_y)\sigma^y,\ C_{4z}:\ \tau^z\left(\frac{\mathbb{1}_\sigma - i\sigma^z}{\sqrt{2}}\right)\mathcal{H}_\Gamma(k_y, -k_x)\tau^z\left(\frac{\mathbb{1}_\sigma + i\sigma^z}{\sqrt{2}}\right), \end{aligned} \tag{205}$$

where $\mathbb{1}_\sigma$ is the 2×2 identity in σ space. SEq. (205) implies an inversion symmetry $\mathcal{I} = M_x M_y M_z$ that transforms $\mathcal{H}_\Gamma(k_x, k_y)$ under the representation:

$$\mathcal{I}:\ \mathcal{H}_\Gamma(-k_x, -k_y). \tag{206}$$

The τ^z contribution to only C_{4z} in SEqs. (205) and (206) reflects that SEq. (203) describes a 2D TCI formed of hybridized s and $d_{x^2-y^2}$ orbitals, as s ($d_{x^2-y^2}$) orbitals are even under $M_{x,y,z}$ and $\mathcal{I}$ and even (odd) under C_{4z}.

Unlike in SN 7 and 8, SEq. (203) is quadratic, and thus eludes the (relatively) straightforward Jackiw-Rebbi analysis performed in those sections. Therefore, to determine the corner modes that result from gapping the TCI edge states, we will employ two distinct approaches. First, we will use EBRs to show that the particular $s-d$-hybridized TCI described by SEq. (1) exhibits the combined quadrupole moments (modulo e) of the $p-d$- and $s-p$-hybridized 2D TIs analytically examined in SN 7 and 8, respectively. Next, to further draw connection between the fragile phase in $p4m1'$ examined in SN 4 and the QI, we will use symmetry arguments and the results of numerical calculations, in conjunction with the analytic results derived in SN 7 and 8, to determine the corner spectrum and charges of the particle-hole-*asymmetric* fragile phase from SN 4 by smoothly deforming the corner spectrum of the Π-symmetric QI phase of SEqs. (1) and (4). We will find in particular that even though this fragile phase is $\mathcal{T}$-symmetric, it still displays Kramers pairs of corner modes, that, because they originate from an imbalanced number of valence and conduction states (6 and 2), which is allowed because Π symmetry is absent, still exhibit the same quadrupole moment Q^{xy} mod $e=e/2$ or $Q^{x^2-y^2}$ mod $e=e/2$ as the corner modes of a *magnetic* QI in $p4m$ when the overall system is half filled.

The Quadrupole Moment of an $s-d$-Hybridized TCI from EBRs – We begin by introducing the EBRs of layer group[20,23,27,56,64]:

$$G = p4/mmm1', \tag{207}$$

which we will use to determine the quadrupole moment of the $s-d$-hybridized TCI highlighted in this work (SEq. (1)) when its edge states are gapped with M_z-breaking, $p4m$-symmetric magnetism. Using the BANDREP tool on the BCS[24,57–59], we induce the following EBRs from the $1a$ Wyckoff position of G (Fig. 2(a) of the main text):

$$\begin{aligned}
(s)_{1a}\uparrow G &\equiv \left(\bar{\rho}_7^+\right)_\Gamma \oplus \left(\bar{\varrho}_5^+\right)_X \oplus \left(\bar{\rho}_7^+\right)_M \\
(p_z)_{1a}\uparrow G &\equiv \left(\bar{\rho}_7^-\right)_\Gamma \oplus \left(\bar{\varrho}_5^-\right)_X \oplus \left(\bar{\rho}_7^-\right)_M \\
\left(d_{x^2-y^2}\right)_{1a}\uparrow G &\equiv \left(\bar{\rho}_6^+\right)_\Gamma \oplus \left(\bar{\varrho}_5^+\right)_X \oplus \left(\bar{\rho}_6^+\right)_M,
\end{aligned} \tag{208}$$

where we have employed the shorthand of SRef. 17 in which, in the left-hand side of SEq. (208), $(\bar{\sigma})_{\mathbf{q}}$ is the EBR induced from the corepresentation of the site-symmetry group of the Wyckoff position at $\mathbf{q}$ that transforms as the (Kramers pair of) atomic orbitals $\bar{\sigma}$, and where, in the right-hand side of SEq. (208), $(\bar{\rho})_{\mathbf{k}}$ is the corepresentation subduced at the TRIM point $\mathbf{k}$. In SEq. (209), all of the position- and momentum-space corepresentations are two-dimensional, and we do not show corepresentations at the X' point ($(k_x,k_y)=(0,\pi)$), because they are fixed to be the same as the corepresentations at the X point ($(k_x,k_y)=(\pi,0)$) by C_{4z} symmetry. In terms of their C_{4z} and parity ($\mathcal{I}$) eigenvalues, as discussed in SN 5:

$$\chi_{\bar{\rho}^{\pm}_{6,7}}(\mathcal{I}) = \pm 2,\ \chi_{\bar{\rho}_6^{\pm}}(C_{4z}) = -\sqrt{2},\ \chi_{\bar{\rho}_7^{\pm}}(C_{4z}) = \sqrt{2}, \chi_{\bar{\varrho}_5^{\pm}}(\mathcal{I}) = \pm 2, \tag{209}$$

where $\chi_{\bar{\rho}}(h)$ is the character of the unitary symmetry h in the corepresentation $\bar{\rho}$, and is equal to the sum of the eigenvalues of h in $\bar{\rho}$, and where $\chi_{\bar{\varrho}_5^{\pm}}(C_{4z})$ does not appear because the X point in $p4/mmm1'$ is not invariant under C_{4z}.

Next, to form the topological valence bands of the 2D phases analyzed in this section and in SN 7 and 8, we invert bands at the Γ point between pairs of EBRs from SEq. (208):

$$\begin{aligned}
[\text{2D TI}]_{p_z-d_{x^2-y^2}} &\equiv \left(\bar{\rho}_7^-\right)_\Gamma \oplus \left(\bar{\varrho}_5^+\right)_X \oplus \left(\bar{\rho}_6^+\right)_M \\
[\text{2D TI}]_{s-p_z} &\equiv \left(\bar{\rho}_7^+\right)_\Gamma \oplus \left(\bar{\varrho}_5^-\right)_X \oplus \left(\bar{\rho}_7^-\right)_M \\
[\text{2D TCI}]_{s-d_{x^2-y^2}} &\equiv \left(\bar{\rho}_7^+\right)_\Gamma \oplus \left(\bar{\varrho}_5^+\right)_X \oplus \left(\bar{\rho}_6^+\right)_M,
\end{aligned} \tag{210}$$

for which, in the convention of SEq. (94), we calculate the mirror Chern numbers C_{M_z} and $\mathbb{Z}_2$ TI indices z_2 to be:

$$\begin{aligned}
C_{M_z}\left([\text{2D TI}]_{p_z-d_{x^2-y^2}}\right) \text{ mod } 4 = -1,\ C_{M_z}\left([\text{2D TI}]_{s-p_z}\right) \text{ mod } 4 = -1,\ C_{M_z}\left([\text{2D TCI}]_{s-d_{x^2-y^2}}\right) \text{ mod } 4 = 2, \\
z_2\left([\text{2D TI}]_{p_z-d_{x^2-y^2}}\right) = 1,\ z_2\left([\text{2D TI}]_{s-p_z}\right) = 1,\ z_2\left([\text{2D TCI}]_{s-d_{x^2-y^2}}\right) = 0.
\end{aligned} \tag{211}$$

Crucially, SEqs. (208) and (210) imply the equivalence:

$$[\text{2D TI}]_{p_z-d_{x^2-y^2}} \oplus [\text{2D TI}]_{s-p_z} \equiv [\text{2D TCI}]_{s-d_{x^2-y^2}} \oplus (p_z)_{1a}\uparrow G. \tag{212}$$

In SN 7 (8) we showed that 2D TIs formed from $p-d$-hybridization ($s-p_z$-hybridization) transition into QIs (trivial insulators) upon gapping their edge states with $p4m$-symmetric magnetism. This implies that when $p4m$-symmetric

magnetism is applied to an insulator whose occupied bands are given by the left-hand side of SEq. (212), then this insulator will transition into a QI (*i.e.* an insulator whose bulk bands and corner modes exhibit Q^{xy} mod $e = e/2$ or $Q^{x^2-y^2}$ mod $e = e/2$). Furthermore, because $(p_z)_{1a} \uparrow G$ is an unobstructed (trivial) atomic limit at the $1a$ position, then it will not exhibit a quadrupole moment under the application of $p4m$-symmetric magnetism (SN 3). Therefore, because the left- and right-hand sides of SEq. (212) must characterize equivalent, QI-nontrivial insulators in the presence of $p4m$-symmetric magnetism, we conclude that the $s-d$-hybridized TCI phase of SEq. (1) will transition into a QI under M_z-breaking, $p4m$-symmetric magnetism.

Fractionally Charged Corner Modes in a Fragile Phase in $p4m1'$ – In this section, we will use symmetry arguments bolstered by numerical observations to show that the QI phase of SEqs. (1) and (4) can transition into a fragile TI in $p4m1'$ (SN 4) that exhibits the same corner charges as a QI (taken modulo e). Because we previously showed that the specific $s-d$-hybridized TCI in SEq. (203) can transition into a QI under breaking M_z and $\mathcal{T}$, then this will allow us to conclude that the same TCI can transition into a fragile phase with corner charges when M_z is broken while preserving $\mathcal{T}$ (SFig. 13). We begin by expanding SEqs. (1) and (4) to linear order about the Γ point:

$$\mathcal{H}_Q(\mathbf{k}) = m\tau^z + u\tau^y(\sigma^y k_x + \sigma^x k_y), \tag{213}$$

in the limit that $t_{PH} = 0$ and where $m = 2t_1 + v_m$. SEq. (213) is the bulk $k \cdot p$ theory of a QI with $M_z \times \mathcal{T}$ and Π symmetries (SN 1); it is specifically invariant under C_{4z}, $M_{x,y}$, $M_z \times \mathcal{T}$, and $\mathcal{I} \times \mathcal{T}$ in the symmetry representation in SEqs. (205) and (206). We note that, because SEq. (213) originates from SEqs. (1) and (4), then, in the limit that $u \to 0$, SEq. (213) reduces to the linear $k \cdot p$ Hamiltonian of the 2D TCI phase of SEq. (1), which is just given by the $m\tau^z$ terms in SEqs. (203) and (213). As $\mathcal{H}_Q(\mathbf{k})$ does not contain terms proportional to τ^x or $\tau^y\sigma^z$, which only arise in a quadratic-order expansion of SEq. (1), then it is slightly underconstrained, and exhibits additional, artificial symmetries:

$$\tilde{\mathcal{T}}:\ \tau^z\sigma^y\mathcal{H}^*_Q(-\mathbf{k})\tau^z\sigma^y,\ \tilde{\mathcal{I}}:\ \tau^z\mathcal{H}_Q(-\mathbf{k})\tau^z, \tag{214}$$

in the notation of SEq. (97). Notably, under a unitary transformation:

$$U = \frac{1}{\sqrt{2}}\left(\mathbb{1}_{\tau\sigma} + i\tau^z\right), \tag{215}$$

which rotates $\tau^{x,y}$ while preserving τ^z and all of the spin matrices σ^i:

$$\tau^y \to \tau^x,\ \tau^x \to -\tau^y,\ \tau^z \to \tau^z, \tag{216}$$

SEq. (213) transforms into:

$$\tilde{\mathcal{H}}_Q(\mathbf{k}) = U\tilde{\mathcal{H}}_Q(\mathbf{k})U^\dagger = m\tau^z + u\tau^x(\sigma^y k_x + \sigma^x k_y), \tag{217}$$

and the symmetries as represented in SEqs. (205) and (214) transform into:

$$\begin{aligned}
&\tilde{\mathcal{T}}:\ \sigma^y\tilde{\mathcal{H}}^*_Q(-k_x,-k_y)\sigma^y,\ \tilde{\mathcal{I}}:\ \tau^z\tilde{\mathcal{H}}_Q(-k_x,-k_y)\tau^z,\ M_x:\ \sigma^x\tilde{\mathcal{H}}_Q(-k_x,k_y)\sigma^x, \\
&M_y:\ \sigma^y\tilde{\mathcal{H}}_Q(k_x,-k_y)\sigma^y,\ C_{4z}:\ \tau^z\left(\frac{\mathbb{1}_\sigma - i\sigma^z}{\sqrt{2}}\right)\tilde{\mathcal{H}}_Q(k_y,-k_x)\tau^z\left(\frac{\mathbb{1}_\sigma + i\sigma^z}{\sqrt{2}}\right), \\
&\tilde{M}_z:\ \tau^z\sigma^z\tilde{\mathcal{H}}_Q(k_x,k_y)\tau^z\sigma^z,
\end{aligned} \tag{218}$$

where $\tilde{M}_z = \tilde{\mathcal{I}}M_x^{-1}M_y^{-1}$. SEqs. (217) and (218) are exactly equal to, respectively, the $k\cdot p$ Hamiltonian (SEq. (95)) and the symmetry representation (SEq. (98)) of the 2D $p_z - d_{x^2-y^2}$-hybridized TI previously analyzed in SN 7. Therefore, we conclude that when Fourier transformed and placed on a disc geometry, SEq. (217) will only admit bulk, edge-projecting mass terms with $L_z^{QI} = 2 + 4a$ (SEq. (139)), and will therefore, exhibit a configuration of 0D (anti)solitons on its boundary with a nontrivial quadrupole moment Q^{xy} mod $e = e/2$ (SEq. (156)) or $Q^{x^2-y^2}$ mod $e = e/2$ (SEq. (163)), as depicted in SFig. 14(a). Because SEq. (213) (and hence SEq. (217)) originated from adding SEq. (4) to SEq. (1) to gap its TCI edge states without closing a bulk gap, then this implies that the 2D $s-d$-hybridized TCI phase of SEq. (1) gaps into *the same* Π-symmetric QI as does a $p-d$-hybridized 2D TI (SN 7) when their edge states are gapped by breaking M_z and $\mathcal{T}$ while preserving $p4m$, in agreement with the result obtained from EBRs (SEq. (208)).

Next, we will exploit this result to demonstrate that a $\mathcal{T}$-symmetric fragile phase in $p4m1'$ that is connected to a ($\mathcal{T}$-broken) $p4m$-symmetric QI without closing a bulk or edge gap also exhibits fractionally charged corner modes. We will first use symmetry arguments (bolstered by explicit numerical calculations) to track the effects of introducing

SEq. (3) and tuning v_{M_z} to be nonzero. The QI phase of SEq. (1) and (4) is invariant under $p4m$ and $M_z \times \mathcal{T}$ symmetries (magnetic layer group[20,23] $p4/m'mm$), as well as Π symmetry, where specifically the terms proportional to u in SEq. (4) break $\mathcal{T}$ and M_z symmetries while preserving their product $M_z \times \mathcal{T}$. We begin by assuming that u is large, and that this corresponds to the dominant mass term in SEq. (133) being $m_2^+ \cos(2\theta)$, giving the $L_z = 2$ distribution of corner charges in SFig. 14(a), which we observe in our numerical calculations (*e.g.*, the hinge states in Fig. 3(f-h) of the main text). We next break $M_z \times \mathcal{T}$ and particle-hole symmetries by introducing nonzero v_{M_z} in SEq. (3); particle-hole symmetry is specifically broken because, when v_{M_z} is nonzero, there are no 4×4 matrices that anticommute with the combination of SEqs. (1), (4), and (3). In the bulk spectrum, because $M_{x,y}$ are still enforced, and $\mathcal{I} = M_x M_y M_z$, v_{M_z} also breaks $\mathcal{I}\times\mathcal{T}$ in the bulk, causing bands to become singly degenerate (observable at $k_z \neq 0, \pi$ in the bulk band structure of the fragile topological Dirac semimetal in Fig. 3(e) of the main text). Tuning v_{M_z} away from zero does not change the filling of the four corner modes, but does allow additional, QI-trivial corner modes to float into the gap (SFig. 14(b)). We depict this process in SFig. 14(b) as the donation from the valence and conduction manifolds of eight additional corner charges (four from each manifold), representative of the zero modes of a QI-trivial $L_z = 4$ mass term (SEq. (190)) split with a $p4m$-symmetric, Π-breaking potential (SFig. 11(b)). More generally, as shown in SEq. (189), QI-trivial 0D states (*i.e.* those that do not carry a Q^{xy} or $Q^{x^2-y^2}$ modulo e of $e/2$ (SEqs. (159) and (163), respectively)) can appear in any arrangement consistent with a linear combination of circular harmonics with $L_z = L_z^{NI} = 4a$ (SEq. (190)) (energetically split in a manner respecting the symmetries of $p4m$ (SFig. 11)); we are in this section only choosing the $L_z = 4$ harmonic to be associated with nonzero v_{M_z} because it is the harmonic with the smallest L_z that is consistent with the trivial corner (hinge) states that we observe in our numerics (Fig. 3(h) of the main text). As with all of the $L_z \neq 0$ mass terms in SEq. (189), the $L_z = 4$ mass term creates four (occupied) modes of the same charge (which we here take to be e) at the same angles as the (QI-nontrivial) $m_2^+ \cos(2\theta)$ term in SEq. (135), as well as four (unoccupied) modes with a different charge (here 0) at four angles that are offset from the first four angles by $\pi/4$; the eight total 0D boundary modes originating from the $L_z = 4$ mass term are equivalent to the QI-trivial bound states of an $s-p_z$-hybridized 2D TI gapped with $p4m$-symmetric magnetism (SFig. 11(b) and SEq. (208)). We depict this Π-breaking arrangement of QI-trivial and non-trivial corner modes in SFig. 14(b).

Next, we begin to restore $\mathcal{T}$ symmetry by tuning u towards zero in SEq. (4), while keeping $v_{M_z} \neq 0$ in SEq. (3); in our numerical calculations using the parameters in Supplementary Table 1, this causes the four unoccupied QI-trivial corner modes to return to the conduction manifold and the fully occupied trivial corner modes to approach the nontrivial 0D states in energy (SFig. 14(c)). This process can specifically be observed in the rod bands near $k_z = 0$ of the noncentrosymmetric fragile topological Dirac semimetal shown in Fig. 3(h) of the main text, in which a QI-trivial set of HOFAs from the valence manifold begins to approach the QI-nontrivial HOFAs at the spectral center. Specifically, in Fig. 3(h) of the main text, near $k_z = 0$, k_z acts exactly like u to break M_z and $\mathcal{T}$, because the SOC term in SEq. (6) is proportional to $u\sin(k_z)$. Next, we set $u = 0$ while keeping $v_{M_z} \neq 0$, resulting in the preservation of the bulk and edge gaps and the restoration of $\mathcal{T}$ symmetry (SFig. 13). As shown in SN 4, the resulting insulator has the bulk symmetries of $\mathcal{T}$-symmetric wallpaper group $p4m1'$, and exhibits fragile topology. To satisfy Kramers' theorem in the fragile phase, the trivial corner modes join with the half-filled, nontrivial corner modes to form 3/4-filled Kramers pairs of corner states, which carry alternating charges $3e/2$ and $e/2$ (SFig. 14(d) and $k_z = 0$ in Fig. 3(h) of the main text). Taken modulo e, this is the same charge per corner as the original Π-symmetric QI in SFig. 14(a), indicating that this fragile phase represents a previously unrecognized example of an insulator without maximally localized symmetric Wannier functions[1–6,10,13,17,24,74,75,83], but with a nontrivial multipole moment[26,28]. This is consistent with our determination in SEq. (208) that the specific $s-d$-hybridized TCI phase of SEq. (1) exhibits the same nontrivial ($e/2$) quadrupole moment as a $p-d$-hybridized 2D TI when their edge states are respectively gapped. Because Π-symmetry can be strongly broken in this fragile phase, then these Kramers pairs of corner states may not necessarily appear as midgap modes. Nevertheless, because they are accompanied by an anomalous mismatch in the number of states in the valence and conduction manifolds, their presence in the spectrum can still be detected by counting the number of states above and below the gap in the energy spectrum of a 2D, $p4m1'$-symmetric insulator. Specifically, the corner modes of this fragile phase (or of the obstructed atomic limit that results from adding trivial bands (SN 4)) can be diagnosed by calculating the energy spectrum with open boundary conditions (OBC) and periodic boundary conditions (PBC) and comparing the number of states below an energy gap; if the number of states below the gap in the OBC and PBC spectra differs by $6+8\mathbb{Z}$ (or $2+8\mathbb{Z}$) then an anomalous number of fragile-phase corner modes are present in the spectrum[17,45] (though, depending on energetics, the states themselves may lie within the bulk manifolds) (SFig. 14(d)). Finally, we tune $v_{M_z} \to 0$, which closes the edge gap, restores $\mathcal{T}$, Π, and M_z symmetries, and induces a $C_{M_z} = 2$ TCI phase (Fig. 2(f-h) of the main text). Because the topology of our model is indifferent to the order in which parameters are tuned as long as the bulk gap is not closed and the symmetries of $p4m$ are not broken, then this process must be equivalent to the reverse of the direct transition from the Π-symmetric TCI to the Π-symmetric QI with four 0D corner states (SFig. 13, white arrow).

The 3/4-filled corner modes that appear in the $p4m1'$-symmetric fragile phase (SFig. 14(d)) can also be understood

from the perspective of the two pairs of helical edge states of the parent mirror TCI phase (SFig. 13). Naively, one might expect that for all $C_{M_z} = 2$ TCIs with $p4m$ symmetry, each pair of edge states gaps under a $p4m$-symmetric, M_z-breaking potential to give four $\pm e/2$-charged corner modes, resulting in an overall trivial quadrupole moment (taken modulo e). However, because the C_{4z} eigenvalues of the specific 2D TCI in SEq. (1) match those of a QI (as well as those of a $p-d$-hybridized 2D TI), as shown in SEq. (212), then the we instead observe that the two pairs of helical modes gap differently when M_z is broken. Specifically, one pair of helical modes gaps to give an anomalous configuration of corner charges with:

$$L_{z,1} = L_z^{QI} = 2 + 4a,\ a \in \mathbb{Z}, \tag{219}$$

where L_z^{QI} is derived in the text surrounding SEq. (139), and the other pair gaps to give a non-anomalous configuration of corner charges with:

$$L_{z,2} = L_z^{NI} = 4a,\ a \in \mathbb{Z}, \tag{220}$$

where L_z^{NI} is derived in the text surrounding SEq. (190). When the TCI gaps directly into the QI through the introduction of u, which breaks M_z and $\mathcal{T}$ while preserving $p4m$, then $L_{z,1}$ and $L_{z,2}$ are free to take their minimum values of 2 and 0, respectively. However, when the TCI gaps into a $p4m1'$-symmetric fragile phase, then $\mathcal{T}$ symmetry requires $L_{z,2}$ to be a nonzero multiple of 4 (SEq. (190)), so that there are fully-filled (or empty) modes at the $4b$ position of $4mm$ (SFig. 10) to pair with the existing QI corner states. These quarter-filled (or quarter-empty) Kramers pairs of fragile-phase corner modes are depicted in SFig. 14(d) and appear in our numerical calculations ($k_z = 0$ in Fig. 3(h) of the main text). Our discovery that 2D TCIs with the same number of edge states, depending on their bulk symmetry eigenvalues, can gap to give different corner-mode phases is reminiscent of a similar phenomenon that occurs in 3D TCIs and higher-order topological insulators (HOTIs)[49,60,62]. Specifically, in a 3D TCI, each 2D surface is characterized by pairs of twofold Dirac cones from bulk double band inversion, which can be deformed into unstable fourfold Dirac cones[11,27,130], and can therefore be gapped by breaking crystal symmetries while preserving $\mathcal{T}$[11,46,49,60,62]. Depending on the bulk symmetry eigenvalues, the pairs of twofold Dirac cones gap to provide either an integer (non-anomalous) or a half-integer (anomalous) contribution to the surface quantum spin Hall effect[18,27], which taken over all Dirac cones and surfaces, determines the presence or absence of intrinsic helical hinge modes, and thus whether the 3D TCI has transitioned into a HOTI or a trivial insulator. Because HOTIs can be expressed as pumping cycles of QIs and other 2D corner-mode phases[17,28,39,46] there is likely a more explicit link between the 2D and 3D cases.

Finally, we note that the observation that six (or two) of the eight fragile-phase corner modes (four Kramers pairs) are filled is subtly in agreement with the results of SN 3 and 4. To see this, we first briefly summarize the details of the Wannier center homotopy for a half-filled QI, which we will then adapt to analyze the analogous homotopy of the three-quarters-filled trivialized fragile phase in $p4m1'$ examined in SN 4. In SN 3, we developed a Wannier center homotopy of the QI obstructed atomic limit (SFig. 2(c,d)). In this homotopy, four Wannier centers "slide" from the $1a$ to the $1b$ Wyckoff position of $p4m$ (Fig. 2(a) of the main text), representing the transition between a trivial insulator with two valence and two conduction bands both originating from orbitals at the $1a$ position, and an obstructed atomic limit with two valence and two conduction bands, which can each be formed into maximally localized, symmetric Wannier orbitals at the $1b$ position (SFig. 2(c,d)). When this pattern of Wannier center "sliding" is terminated on the boundary of a finite-sized QI, four of the bulk Wannier orbitals (one per corner) become the 0D corner states of the QI. As there are four Wannier orbitals in the Wannier center homotopy, and two orbitals originate from valence bands if the system remains half-filled, then we conclude that each of the four corner states is half-filled, in agreement with the results of SN 7.

A similar analysis reveals that the 3/4-filled corner modes of the fragile phase analyzed in this section can *also* originate from a Wannier center homotopy, even though they can appear on the corners of an insulator that is not Wannierizable. When our four-band fragile model in $p4m1'$ (SEqs. (1) and (3)) is half-filled, the two valence and two conduction bands each exhibit fragile topology. In SN 4, we proposed through an irreducible-representation equivalence (SEq. (67)) and demonstrated numerically (SFig. 3) that this obstruction to forming a Wannier description can be lifted by introducing four (trivial) valence bands. Taken over the now six total valence bands (two fragile and four trivial) and two fragile conduction bands, a homotopic description of sliding eight Wannier centers from the $1a$ to the $1b$ position of $p4m1'$ can be formed (SEq. (58)). When this eight-band pattern of sliding Wannier orbitals is terminated on the boundary of a finite-sized system, it results in eight corner states (one Kramers pair per corner). Counting the occupancy of the eight sliding Wannier centers, as stated previously, six originate from valence bands and two originate from conduction bands, yielding an overall 3/4-filled, eight-band obstructed atomic limit that carries the same corner degeneracy and charges as the original four-band fragile insulator at half filling (SFig. 14(d)). Specifically, because unobstructed (trivial) atomic limits do not exhibit corner charges along a boundary with a trivial insulator with atoms at the same Wyckoff positions (SN 3), then we conclude that the 3/4-filled Kramers pairs of corner modes

must be a consequence of the (fragile) topology of the two valence bands (and the two conduction bands) of our original four-band model (SEqs. (1) and (3)). This equivalence between the corner charges of a fragile phase (the four-band fragile TI in $p4m1'$ characterized by SEqs. (1) and (3)) at one filling (here 1/2) and those of an obstructed atomic limit at a (sometimes) different filling (here 3/4) is also explored in SRef. 17, and can be inferred from the results of SRef. 69.

10. Relaxation of $M_{x,y}$ Symmetry in QIs and Related Fragile Phases

In this section, we discuss the consequences of relaxing $M_{x,y}$ symmetry while preserving C_{4z} symmetry on the corner spectra of the previous QI and fragile phases (SN 7 and 9, respectively). First, we begin with the $\mathcal{T}$-broken QI phase in $p4m$, and then later, we subsequently reintroduce $\mathcal{T}$ symmetry to analyze the fragile phase in $p4m1'$ discussed in SN 4 and 9.

In SN 7, we showed that the QI with $M_{x,y}$ and C_{4z} symmetries can be diagnosed by observing that $L_z^{QI} = 2 + 4n$, $n \in \mathbb{Z}$ (SEq. (139)) states are missing from the valence (and conduction) manifolds of the spectrum of a $p4m$-symmetric insulator calculated with $4mm$-symmetric open boundary conditions (OBC), relative to the spectrum calculated with periodic boundary conditions (PBC) (SFig. 9). When the L_z^{QI} missing states from the valence manifold appear in the bulk gap, they, along with L_z^{QI} states from the conduction manifold, represent four corner modes localized to either the $4a$ or the $4b$ Wyckoff position of point group $4mm$ (as well as 8n trivial states localized at the general position $8c$ (SFig. 10)). Specifically, because of the in-plane mirrors $M_{x,y}$, if only four states are present at the same energy in the OBC spectrum, then they *must* appear in a finite-sized QI with $4mm$ at the fixed angles of either $M_{x,y}$ ($4a$):

$$\theta_{4a} = n\pi/2,\ n \in \mathbb{Z}, \tag{221}$$

or at the fixed angles of $M_{x\pm y}$ ($4b$):

$$\theta_{4b} = n\pi/2 + \pi/4,\ n \in \mathbb{Z}. \tag{222}$$

Next, we consider introducing a perturbation that breaks $M_{x,y}$ symmetries while preserving C_{4z} and does not close a bulk gap. Using the point group tables on the BCS[40], we determine that this perturbation transforms as the irreducible representation[27,131–133] A_2 of $4mm$, because:

$$\chi_{A_2}(C_{4z}) = \chi_{A_2}(C_{2z}) = 1,\ \chi_{A_2}(M_{x,y}) = -1, \tag{223}$$

where $\chi_\rho(h)$ is the character of the symmetry h in the irreducible representation[40,91]. Applying an A_2 perturbation reduces the point group of the finite-sized QI from $4mm$ to 4. As shown in SFig. 15(a), point group 4 does not distinguish between any points on a circle; instead it only hosts a $4a$ position that labels four C_{4z}-related angles:

$$\tilde{\theta}_{4a} = \theta_{4b} + \vartheta, \tag{224}$$

where θ_{4b} refers to the coordinates of the $4b$ sites of $4mm$ (SEq. (222)) and ϑ is a free angle. Here, we have chosen ϑ with respect to the $4b$ position of $4mm$, rather than the $4a$ position, because the corner modes of previous QI models[26,28] with $4mm$ appeared at $4b$. In terms of the circular harmonics $f^{\pm}_{L_z}(\theta)$ whose zeroes determine the locations of the QI corner modes (SEqs. (131) through (154)), the reduction from $4mm$ to 4 removes the distinction between $f^{+}_{L_z}(\theta)$ and $f^{-}_{L_z}(\theta)$, resulting in a new set of circular harmonics given by:

$$f^{\vartheta}_{L_z}(\theta) = \cos\left[L_z(\theta - \vartheta)\right]. \tag{225}$$

SEq. (225) implies that perturbatively relaxing $M_{x,y}$ unpins the four corner modes from the fixed angles of $4mm$ (SFig. 15(b)), permitting them to be rotated by a symmetry-allowed boundary term that does not change the bulk topology. This allows the quadrupole moment of the corner modes (SEqs. (156) and (162)) to freely rotate between $Q^{xy} = e/2$, $Q^{x^2-y^2} = 0$; $Q^{xy} = 0$, $Q^{x^2-y^2} = e/2$; and all intermediate values with a total quadrupole moment of $e/2$. However, and crucially, because C_{4z} symmetry still relates the four corner modes to each other, a bulk-gap-preserving chemical potential can only move the four corner modes together in energy (and rotate them as a whole about the origin ($1o$ in SFig. 15(a))), but it cannot lift the anomalous absence of L_z^{QI} states from the valence and conduction manifolds in the OBC spectrum. Therefore, if a QI is terminated on a boundary that only preserves C_{4z}, but not $M_{x,y}$, it will still exhibit four anomalous corner modes with an $e/2$ quadrupole moment whose orientation is a free parameter ϑ. This is analogous to the $e/2$ dipole moment of the inversion- ($\mathcal{I}$)- and $C_{2z} \times \mathcal{T}$- protected fragile phases introduced in SRefs. 11 and 17. In the fragile phases in those works, the bulk (fragile or obstructed-atomic-limit) topology guaranteed the presence of $2 + 4a$ corner modes that exhibit a net $e/2$ dipole moment. However, unlike the

symmetries of $4mm$ (SFig. 10) neither $\mathcal{I}$ nor $C_{2z} \times \mathcal{T}$ fixes any points on the boundary of a circle (they only relate pairs of points). Therefore, the direction of the anomalous $e/2$ dipole moment of the corner modes of the fragile phases in SRefs. 11 and 17 is a free parameter, analogous to ϑ in SEq. (225).

We note that, in this section, we only consider the case in which $M_{x,y}$ is perturbatively broken in the bulk. This guarantees that the bulk gap does not close when $M_{x,y}$ is broken, and keeps the bulk bands adiabatically connected to those of a QI. This also allows us to avoid symmetry-allowed intermediate Chern insulating phases between a trivial insulator and a QI in wallpaper group[27,56] $p4$, the $M_{x,y}$-broken subgroup of $p4m$. Specifically, if the bulk in-plane mirrors of $p4m$ are preserved, then bands at Γ and M remain twofold degenerate, and the (C_{4z}) eigenvalues of the occupied bands only distinguish between QI and trivial phases (SN 3). Conversely, if $M_{x,y}$ are broken, then bands at all TRIM points become singly degenerate. With only singly-degenerate bands, then transitions between trivial insulating and QI phases can only be facilitated through multiple, independent band inversions. However, unlike with wallpaper group $p4m$, a system with only C_{4z} symmetry can generically pass through intermediate Chern insulating phases when singly-degenerate bands with different C_{4z} (or C_{2z}) eigenvalues are inverted[33]. Therefore, because the phase boundaries separating trivial, Chern, and quadrupole insulators in $p4$ are considerably more complicated than the simple boundary separating trivial and QI phases in $p4m$ (SN 3), we leave the complete analysis of QI transitions in $p4$ for future works.

Finally, we note that the arguments in this section also apply to the corner modes of the fragile phase in $p4m1'$ examined in SN 9 with minimal modification. As shown in SFig. 14, the eight three-quarters-filled (or quarter-empty) corner modes of a fragile phase in $p4m1'$ are localized at the $4a$ or $4b$ Wyckoff position of $4mm1'$ (SFig. 10), the point group generated by adding $\mathcal{T}$ to $4mm$. Here, the presence of $\mathcal{T}$ symmetry requires that all states are at least twofold degenerate; therefore, the constraints imposed by C_{4z} and $\mathcal{T}$ symmetry require that a bulk-gap-preserving chemical potential moves all eight corner states together in energy. When $M_{x,y}$ are relaxed without breaking C_{4z} or $\mathcal{T}$, then the point group of the finite-sized system is reduced from $4mm1'$ to $41'$, and the eight corner modes can be rotated as a set by a free angle ϑ (SFig. 15(c)). However, as previously with the QI, because a C_{4z}- and $\mathcal{T}$-preserving potential that does not close the bulk or edge gap must move all eight corner modes together in energy, then the valence manifold of the OBC spectrum will still display an anomalous absence of $6+8n$ (or $2+8n$) states when calculated relative to the PBC spectrum. Therefore, if this fragile phase is terminated on a boundary that only preserves C_{4z} and $\mathcal{T}$, but not $M_{x,y}$, it will still exhibit four anomalous Kramers pairs of corner modes with an $e/2$ quadrupole moment whose orientation is a free parameter ϑ (though, due to strongly broken particle-hole symmetry, the corner modes may be buried in the valence or conduction manifolds in the OBC spectrum).

11. Numerical Investigations of the Surface States of HOFA Dirac Points

A Dirac point in a HOFA semimetal represents the bulk quantum critical point between QI and trivial phases (SN 3 and 5). In previous works, QI phase transitions have been shown to be accompanied by changes in the edge polarization that correspond to "edge" quantum critical points[26,28,115,134]. It is therefore natural to ask whether there are additional states in the surface spectrum (besides the projections of the bulk Dirac points) that would be representative of a QI edge gap closure, analogous to the surface states of the higher-order "surface-only" semimetals proposed in SRef. 134, which appeared while this extensive work was in preparation. To accomplish this, we begin with the $\mathcal{T}$-broken model of a HOFA semimetal in SEq. (5), and place it on a slab geometry that is infinite in the y and z directions and finite with 500 layers in the x direction. Here, this semimetallic system, while not particle-hole symmetric, still features bulk Dirac crossings at $k_x = k_y = 0, k_z = k_d^{\pm}$ that lie in the spectral center $N/2$ (Fig. 3(c) of the main text), where $N = 500 \times 4$, with the factor of 4 coming from the two spin-1/2 s and two spin-1/2 d orbitals in each unit cell. This guarantees that, taking E_d to be the energy of the bulk Dirac points at $k_z = k_d$, the spectrum at each k point near k_d exhibits $N/2$ states with energy $E > E_d$ and $N/2$ states with energy $E \leq E_d$.

We plot in SFig. 16(b,c,d) the orbital-summed wavefunction magnitude $\sum_{s,d,\sigma} |\psi|^2$ of the 64 energy eigenstates nearest $E = E_d$ as a function of k_z, taking k_y=0 (SFig. 16(a)). We define k_b (k_c) to be a point at $k_y = 0$ with $k_z <$ ($>$) k_d (SFig. 16(a)). We then define a state to be edge localized if more than 75% of its probability density lies within within the first or the last 100 layers of the slab. If a state exhibits equal probability density on all layers, we consider it to be delocalized. If a state is neither edge localized nor delocalized, then we consider it to be bulk localized. At $k_y = 0$, $k_z = k_b$, all of the modes are bulk localized, except for two pairs of gapped surface Fermi arc states (SFig. 16(a,b)), whose splitting $\tilde{m}$ in energy scales as $u\sin(k_d - k_z)$ in SEq. (5). Remaining at $k_y = 0$ and taking increasing values of k_z that pass through the bulk Dirac point at k_d, all of the states become perfectly delocalized at $k_z = k_d$ (*i.e.*, they display equal probability density on all layers), and then become bulk localized at $k_c > k_d$ (SFig. 16(a,c,d)). It is clear that, aside from the four surface Fermi arc states at $k < k_d$, which separate into groups of two arcs on each of the two slab surfaces, there are no additional bound states on the 2D faces of this system; at $k_z = k_d$, all of the modes in the bulk and on the faces and hinges become delocalized. We therefore do not observe

any signatures of additional surface states bound to the projections of the bulk 3D Dirac points.

12. Space Groups Supporting Dirac Semimetals with Quadrupolar HOFA States

In this section, we deduce the set of 3D space groups (SGs) capable of hosting Dirac semimetal phases with HOFA states. For the purposes of this work, we restrict consideration to Dirac points equivalent to the critical point between 2D QI and trivial phases, as discussed in SN 3 and 5. Other nodal points equivalent to other 2D critical points occur in other SGs (both with and without SOC[11,17]), and if the symmetries that enforce their anomalous corner (hinge) modes can be preserved on a rod, they will also exhibit HOFAs[11]; we leave the complete enumeration of such bulk nodal points and variants of HOFA states for future works. First, we will use the symmetries of quasi-one-dimensional rods, known as the crystallographic "rod groups[23]," to derive a set of SGs in which Dirac semimetals exhibit HOFA states derived from QIs. In real materials, unlike in the models examined in this work, there are generically multiple kinds of atoms, each with different valence atomic orbitals, occupying different Wyckoff positions throughout the unit cell[135]. Therefore, instead of searching for candidate HOFA semimetals by restricting to specific cases of atomic-orbital hybridization (*e.g.*, $s-d_{x^2-y^2}$- or $p_z-d_{x^2-y^2}$-hybridization at the $1a$ position, like the models in SEqs. (6) and (84) respectively), we will exploit the analysis in SN 7 through 10 to perform the more general search for topological semimetals whose low-energy theories bind and position-space symmetries protect QI-nontrivial corner- (hinge-) states. We will find that HOFA states are generically present on the hinges of Dirac semimetals whose SGs have point group[40] $4mm$ (or higher) when they are cut into rods that preserve a fourfold axis. Then, we will demonstrate that body-centered Dirac semimetals can also exhibit HOFA states, even though fourfold axes do not coincide with crystal lattice vectors in body-centered SGs[19]. Specifically, when a body-centered Dirac semimetal is cut into a rod that preserves a fourfold axis, the lattice vectors of the finite-sized rod cannot coincide with the original lattice vectors of the bulk crystal, and so one might be concerned that HOFA states do not appear along the rod, due to the same zone-folding effects that negate the presence of edge states on armchair-terminated graphene[51–55]. However, by explicitly performing the BZ folding from a Dirac semimetal in a body-centered SG to a rod that preserves a fourfold axis, we will show that HOFA states are still generically present on the rod hinges. We find that this occurs because the 3D HOFA Dirac points examined in this work arise from band inversion ("enforced semimetals" in nomenclature of SRef. 136), and are thus free to shift in momentum along high-symmetry BZ lines, whereas, conversely, the Dirac points in graphene are pinned by band connectivity to the high-symmetry BZ points[24,51] K and K' ("enforced semimetal with Fermi degeneracy" in the nomenclature of SRef. 136).

In this work, we define a rod as a 1D crystal that is invariant under 3D symmetry operations; the symmetries of these systems are given by the rod groups[20,23]. For our purposes, we specialize to the crystallographic rod groups, which only contain symmetry elements that are also allowed in the 3D space groups[23]. We introduce the subscript RG to distinguish the symbols for rod groups from those for layer groups[20,27,56,64], as there are rod groups and layer groups whose symbols are otherwise indistinguishable[20,23] (*e.g.*, $(p4mm1')_{RG}$ and $p4mm1'$). Each of the crystallographic rod groups, when in-plane lattice translations $T_{x,y}$ are added to it, is isomorphic to a 3D space group. For example, the $\mathcal{T}$-symmetric rod group $(p4mm1')_{\text{RG}}$ is generated by T_z, C_{4z}, and M_x, and is related to SG 99 $P4mm1'$ by:

$$P4mm1' \equiv E(p4mm1')_{\text{RG}} \cup T_x(p4mm1')_{\text{RG}}, \tag{226}$$

where E is the identity operation and the other in-plane translation T_y is generated by $C_{4z} \times T_x$.

We will shortly specifically show that when Dirac semimetals whose SGs have point group[30,31,40] $4mm$ (or higher) are cut into a rod whose rod group has point group 4 (or higher), the rod will exhibit quadrupolar HOFA states. From an experimental perspective, the theoretical process of "cutting a 3D crystal into a quasi-1D rod with point group 4" is equivalent to growing or cutting a sample into a nanowire whose long axis is coincident with a bulk fourfold axis. It is possible that HOFA states may also be observable through momentum-resolved probes of the interior hinges of the pits of C_4-symmetric arrangements of step edges, as high-symmetry step edge configurations have been shown in experiment to exhibit the same $d-2$-dimensional hinge states as nanowires[50].

Symmetry Conditions for Dirac Points with HOFA States Derived from QIs – We will now derive the symmetry conditions for Dirac semimetals to exhibit anomalous HOFA states originating from QIs. We will divide this process into two steps, based on the analyses performed in SN 7, 5, and 10. First, we will use the results of SN 7 and 5 to show the more narrow result that Dirac semimetals whose SGs have point group $4mm$ or higher will exhibit HOFA states pinned to the fixed angles of $4mm$ (*i.e.*, the $4a$ or $4b$ position in SFig. 10) if they can be cut into rods with $4mm$ (or higher) symmetry. In these Dirac semimetals, the HOFA states are pinned to the same angles (the $4a$ or $4b$ position in SFig. 10) at all values of k along the rod axis (SFig. 17(b)). Then, we will use the results of SN 10 to further extend consideration to tetragonal and cubic SGs with bulk $4mm$ symmetry that *cannot* be cut into rods with point group $4mm$, but which nevertheless support Dirac semimetals with HOFA states. In these Dirac semimetals, the quadrupolar HOFAs generically appear at each rod k point at free angles related by fourfold symmetry (SFig. 15).

The list of SGs supporting HOFA Dirac semimetals obtained here will consequently include the list of SGs supporting Dirac semimetals with fixed-angle HOFA states; we therefore reproduce the more general list of SGs supporting Dirac semimetals with free-angle HOFA states in Table 2 of the main text.

Dirac Semimetals with HOFA States Pinned to Fixed Angles – We begin by determining the SGs in which Dirac semimetals, when cut into $4mm$-symmetric rods, exhibit HOFA states that are pinned at each QI-nontrivial rod k point (SN 5) to high-symmetry angles corresponding to the $4a$ or $4b$ Wyckoff position of point group $4mm$ (SFig. 10). For the $4a$ position, these angles are[30,31,40]:

$$\theta_n^{4a} = \{0, \pi/2, \pi, 3\pi/2\}, \tag{227}$$

and for the $4b$ position, the angles are:

$$\theta_n^{4b} = \{\pi/4, 3\pi/4, 5\pi/4, 7\pi/4\}. \tag{228}$$

We note that, as we will subsequently see, the list of SGs obtained here is *not* the most general list of SGs in which Dirac semimetals exhibit quadrupolar HOFA states. For completeness, however, we will still here complete the more restrictive tabulation of the SGs that support Dirac semimetals with HOFAs pinned to $\theta_n^{4a,4b}$ (SEqs. (227) and (228)) as an intermediate step towards the complete list that will appear later in this section (and is reproduced in Table 2 of the main text) of SGs supporting Dirac semimetals with HOFA states directly derived from the specific QI phase introduced in SRef. 26.

To obtain the relevant SGs, we first analyze the conditions that allowed the low-energy theory of the 2D QI in SN 6 to exhibit corner modes pinned to $\theta_n^{4a,4b}$ in SEqs. (227) and (228). In SN 6, we demonstrated that the presence of four corner modes localized to $\theta_n^{4a,4b}$ in the low-energy theory of a 2D QI occurred under three conditions:

1. The bulk differed from a trivial (unobstructed) atomic limit through a band inversion at a k point whose little co-group had a subgroup (possibly itself) isomorphic to $4mm$.

2. The finite-sized QI region in position space (*i.e.* in SN 6, the circle whose interior was a QI) was invariant under the action of point group $4mm$ (SFig. 17(a)).

3. The valence and conduction bands of the bulk $k{\cdot}p$ theory had different complex-conjugate pairs of C_{4z} eigenvalues (*i.e.*, transformed under different (co)representations of $4mm$ (SN 3)).

These constraints guarantee that the presence of 0D modes, which localize on the corners of a square geometry[26,46,50,131], is a consequence of a topological quadrupole moment $Q^{xy} = e/2$ or $Q^{x^2-y^2} = e/2$ (SEqs. (156) and (162)) that cannot be removed without breaking a symmetry or closing a *bulk* gap (SN 6). If C_{4z} is relaxed, then the boundary mass terms may change locally, allowing for corner modes to be removed by *surface* gap closures[26,28]. In this work, we are only concerned with systems that exhibit boundary states (both edge (surface) and corner (hinge)) as a consequence of their bulk topology. This focus allows us to predict and analyze robust, intrinsic $d-2$-dimensional modes based on bulk topology, as opposed to predicting extrinsic corner (hinge) states whose presence depends on surface physics. We therefore exclude 2D insulators whose anomalous corner modes may be removed by closing an edge (*i.e.* Wilson) gap without closing a bulk gap, such as the QI phases without fourfold rotation symmetry in SRefs. 26 and 28. When these constraints are extended to 3D semimetals, we therefore also exclude the "surface-only" HOFA-semimetal phases introduced in SRef. 134, in which the presence of HOFA states is *entirely* dependent on the details of surface potentials, and is thus not a consequence of the bulk topology and difficult to predict in real materials through density functional theory.

To identify 3D SGs that support Dirac semimetals with HOFA states pinned to $\theta_n^{4a,4b}$ in SEqs. (227) and (228), we therefore require three conditions:

1. There exist lines in the BZ whose little groups contain $4mm$.

2. When a crystal in this SG is cut into a rod that is finite in two dimensions and infinite along the direction of the fourfold axis from condition 1, its rod group has a point group that contains $4mm$. This implies that the finite-sized rod is symmetric under the action of a rod group[23] that is a (possibly $\mathcal{T}$-symmetric) supergroup of one of the type-I magnetic rod groups[20,23,137] $(p4mm)_{RG}$, $(p4_2cm)_{RG}$, $(p4_2mc)_{RG}$, or $(p4cc)_{RG}$, as those are the lowest-symmetry rod groups that contain these symmetries.

3. The BZ line from condition 1 must have at least two distinct two-dimensional (co)representations, characterized by different complex-conjugate pairs of C_{4z} eigenvalues, that can cross to form a symmetry-stabilized Dirac point. As shown in SN 5, this, along with the reflection symmetries from condition 1, guarantees that this Dirac point is equivalent to the critical point between 2D trivial and QI phases. This condition excludes, for example, BZ lines in nonsymmorphic SGs along which additional crystal symmetries beyond $4mm$ act to make corepresentations fourfold degenerate[19,56,57].

In order to cut a 3D crystal into a rod with a point group that contains $4mm$, that crystal must have two reflection planes (mirrors or glides) that intersect on a fourfold axis (rotation or screw); for z-directed rods, this requirement necessarily excludes glide reflections with translations in the xy-plane, which cannot be preserved on a rod. The rod group of this rod is a subgroup of the 3D space group of the crystal; cutting an infinite 3D crystal into a rod only lowers the overall symmetry. Therefore, to identify the relevant SGs, we begin by enumerating the rod groups with point groups that contain $4mm$, of which the nonmagnetic examples are given in Supplementary Table 6.

Three of the rod groups in Supplementary Table 6 are noncentrosymmetric (34 – 36) and three are their centrosymmetric supergroups (38–40). In particular, the three noncentrosymmetric rod groups are isomorphic, under the addition of in-plane lattice translations, to SGs 99, 101, 103, and 105 (both SGs 101 and 105 have the same symmetries as rod group 35 under the addition of in-plane lattice translations (Supplementary Table 6)). Consequently, *all* $\mathcal{T}$-symmetric space groups that characterize crystals that can be cut into rods with point group $4mm1'$ or its supergroup $4/mmm1'$ are necessarily supergroups of these four space groups. For a Dirac semimetal in one of these SGs, if the Dirac points lie along an axis of fourfold rotation, then a rod cut from this crystal along the same axis will exhibit HOFA states, as such a Dirac point is necessarily equivalent to the critical point between 2D trivial and QI phases (SN 5). We obtain these space groups by using MINSUP on the BCS[30,31,63] to find all of the supergroups of the space groups listed in Supplementary Table 6. We then impose conditions 1 and 3 explicitly by using SRefs. 19, 24, 57–59 to identify the BZ lines that admit fourfold Dirac points. Specifically, these lines have little groups with with $4mm$ (or higher) symmetry and have at least two, two-dimensional corepresentations; when bands with different corepresentations cross along these lines, a fourfold Dirac point forms[110]. This excludes, for example, BZ lines in nonsymmorphic SGs along which additional crystal symmetries combine with $4mm$ to make all corepresentations four-dimensional[19,56,57] (*e.g.*, MA in SG 129 $P4/nmm1'$). The full list of space groups and BZ lines is listed in Supplementary Table 7.

We note that the preceding arguments contain a subtlety when applied to the space supergroups of the rod groups in Supplementary Table 6 with fourfold *screw* axes (rod groups 35 and 38). Along momentum-space lines with a fourfold screw axis defined by a $b/4$ fractional lattice translation:

$$s_{4_b z} = C_{4z} T_{b/4}, \tag{229}$$

as well as with two orthogonal reflections (*e.g.*, the line ΓZ in SG 105 $P4_2mc1'$), the symmetries in the basis of the four bands nearest a Dirac point can be represented by[56,130]:

$$s_{4_b z} = \tau^z \left(\frac{\mathbb{1}_\sigma - i\sigma^z}{\sqrt{2}}\right) \Lambda(k_z) = C_{4z}\Lambda(k_z),\ M_{x,y} = i\sigma^{x,y}, \tag{230}$$

where:

$$\Lambda(k_z) = e^{\frac{ibk_z}{4}} = e^{ik_z t_s}. \tag{231}$$

While $t_s = 1/2$ in the units of the z-direction lattice spacing for the 4_2 screw in SG 105, more generally, the values $t_s = 1/4,\ 3/4$ are also allowed in other SGs with fourfold screws[19]. At first, the symmetry representations in SEq. (230) appear distinct from those employed in SN 7 to characterize the corner modes of a QI (SEq. (98)). However, we note that, in the absence of antiunitary symmetries, such as $\mathcal{T}$ or $\mathcal{I}\times\mathcal{T}$ (which are present in $\mathcal{T}$- and centro- ($\mathcal{I}$-) symmetric SGs), we are free to rotate the phases of the representations of crystal symmetries without changing their commutation relations[56,64]. Specifically, here, we are also permitted to employ the symmetry representation:

$$\bar{C}_{4_b z} = s_{4_b z}\Lambda^*(k_z) = \tau^z \left(\frac{\mathbb{1}_\sigma - i\sigma^z}{\sqrt{2}}\right),\ M_{x,y} = i\sigma^{x,y}, \tag{232}$$

where we have labeled $\bar{C}_{4_b z}$ with a bar to emphasize that it is not the representation of a real C_{4z} symmetry, but is rather an alternative representation of the fourfold screw symmetry $s_{4_b z}$. SEq. (232), is *identical* to the representation used in SN 7 to predict QI corner modes (SEq. (98)). Therefore, a Dirac point with the nonsymmorphic symmetry representation in SEq. (230) is described by the *same* $k\cdot p$ Hamiltonian as a Dirac point with the symmorphic symmetry representation in SEq. (232), in agreement with the methods employed in SRef. 110 to characterize the band-inversion Dirac points in the nonsymmorphic Dirac semimetal Cd_3As_2. Furthermore, even though the eigenvalues of screw symmetries, unlike the eigenvalues of rotations, depend on the choice of BZ (*i.e.*, are not 2π-periodic)[27,56,89,90,130], *within* each BZ, two bands can still be unambiguously labeled with distinct pairs of fourfold screw eigenvalues. Crucially, because, within each 2D BZ slice (here indexed by k_z), the presence or absence of QI-nontrivial corner modes (HOFA states) only depends on the *difference* in fourfold rotation eigenvalues between the valence and conduction bands (SN 7 and 8), and because we have already shown that symmorphic Dirac semimetals with $4mm$ (or higher) point-group symmetry exhibit HOFA states when cut into rods with point group $4mm$ (or higher), then SEq. (232)

allows us to conclude that noncentrosymmetric nonsymmorphic Dirac semimetals with point group $4mm$ *also* exhibit HOFA states.

Furthermore, we can show that SEq. (232) also applies in $\mathcal{I}$- (centro-) and $\mathcal{T}$-symmetric nonsymmorphic Dirac semimetals. Specifically, the HOFA Dirac points analyzed in this work occur away from TRIM points, and therefore, in an $\mathcal{I}$- and $\mathcal{T}$- symmetric Dirac semimetal, the Hamiltonian in the vicinity of each Dirac point only respects the combined magnetic symmetry $\mathcal{I}\times\mathcal{T}$ (in addition to $4mm$). We can incorporate $\mathcal{I}\times\mathcal{T}$ into the symmetry representation in SEq. (232) by choosing a representation for $\mathcal{I}\times\mathcal{T}$ that neither commutes nor anticommutes with[64] $\bar{C}_{4z}$. However, because, in the bulk, $\mathcal{I}\times\mathcal{T}$ only serves to make bands doubly degenerate away from[56] $k_{x,y}=0$, and, on the corners, it does not change the anomalous QI state counting in SFig. 9, then we conclude that $\mathcal{I}\times\mathcal{T}$- and fourfold-screw-symmetry-enforced Dirac points also exhibit intrinsic HOFA states. This argument only breaks down when both $\mathcal{I}$ and $\mathcal{T}$ are individually enforced, which can only occur at TRIM points, and thus does not apply to the band-inversion Dirac points discussed in this work. While previous works have also introduced nonsymmorphic-symmetry-enforced fourfold Dirac[56,64,130,138,139] (and eightfold double-Dirac[92,131]) points that are specifically pinned to TRIM points, we will leave a detailed analysis of potential higher-order topology in these *enforced* semimetals for future works, though we do predict that enforced semimetals should also exhibit higher-order topological effects. Additionally, because our arguments here do not depend on the details of the exact phase $\Lambda(k_z)$ in SEq. (230), then they will also apply without further modification to the Dirac semimetals with free-angle HOFA states enforced by 4_1 $(t_s=1/4)$ and 4_3 $(t_s=3/4)$ screw symmetries that will be introduced later in this section.

Finally, we note that, as implied from the discussion in the main text and $\mathcal{H}_{H1}(\mathbf{k})$ (SEq. (5)), HOFA states are also permitted in some magnetic Dirac semimetals. However, as the number of known magnetic structures is small compared to the number of known materials[135,140], it is relatively difficult to identify magnetic materials candidates. Therefore, we leave the complete enumeration of all magnetic space groups that admit Dirac semimetals with HOFA states for future works. However, as examples, by applying the procedure used to generate Supplementary Tables 6 and 7 to the magnetic rod and space groups, we conclude that magnetic Dirac points with HOFAs may form along ΓZ in magnetic SGs $P4/m'mm$ (123.341 in the Belov-Nerenova-Smirnova (BNS) notation[20]) and P_c4/ncc (130.432 in the BNS notation). The first group is that of our model, $\mathcal{H}_{H1}(\mathbf{k})$ in SEq. (5), and the second group characterizes the antiferromagnetic (AFM) Dirac semimetal phase of CeSbTe[141]. As the Dirac points in the AFM phase of CeSbTe lie along lines with $4mm$ symmetry and its magnetic space group contains $P4cc$, CeSbTe will exhibit reflection-fixed HOFA states when cut into a rod with $(p4cc)_{RG}$ (or higher) symmetry (though the HOFA states may be difficult to separate from the hinge projections of bulk and surface states in CeSbTe).

Dirac Semimetals with HOFA States at Free Angles – Building upon the previous discussion, we will here develop the most general list of SGs in which Dirac semimetals exhibit HOFA states derived from the QI model introduced in SRef. 26. Previously, we showed that if both infinite crystals and finite-sized rods of a Dirac semimetal preserve fourfold axes and two in-plane reflection symmetries (*i.e*, have point groups that contain $4mm$), then the semimetal will exhibit HOFA states fixed to the rod hinges at $\theta=\theta_n^{4a,4b}$ in SEqs. (227) and (228) and SFig. 10. However, we also previously showed in SN 10 that if a 2D QI is formed from band inversion about a k point whose little co-group contains $4mm$, then its four corner modes remain anomalous when the system is terminated in an $M_{x,y}$-breaking, C_{4z}-symmetric geometry. In this lower-symmetry geometry with point group[40] 4, the corner modes become unpinned from the fixed angles of $4mm$, and their $e/2$ quantized quadrupole moment becomes free to lie at any intermediate angle between xy and x^2-y^2 (SFig. 15). Exploiting this result, we will determine in this section a list of SGs whose bulk crystals support $4mm$-symmetric HOFA Dirac points, even though they cannot all be cut into rods that simultaneously preserve fourfold axes and in-plane reflections. Specifically, in some of these SGs, the in-plane reflections (mirrors and glides) also contain in-plane translations relative to the fourfold axes; when they are cut into rods that preserve one of their fourfold axes, their in-plane reflections are necessarily broken. The list of SGs that we obtain in this section will contain the SGs previously tabulated in Supplementary Table 7, as well as additional SGs with bulk $4mm$ symmetry.

First, we will explain our restriction in this section to SGs with point group $4mm$ or higher, and not to SGs with point group 4 or higher. In SGs with point group $4mm$, bands along at least one fourfold axis are generically twofold degenerate, because the double-valued (spinful) in-plane reflections of $4mm$ anticommute (SN 3). Therefore, along a line with $4mm$ symmetry, there are two distinct corepresentations with different fourfold rotation eigenvalues, which can cross to form a symmetry-stabilized Dirac point (SN 5). However in lower symmetry SGs with only point group 4, then absent additional symmetries, bands are generically singly degenerate along fourfold axes away from the TRIM points, and can only cross to form Weyl points[95,142]. Additionally, while other SGs with other point groups (such as[30,31,40] $4/m1'$) also exhibit twofold degenerate corepresentations along fourfold axes that can cross to form symmetry-stabilized Dirac points, we have not explicitly analyzed QI-nontrivial obstructed atomic limits and fragile phases in those SGs. We will, for now, consider HOFA states in these SGs beyond the scope of the present work due to our focus on Dirac semimetals directly derived from the QI phase introduced in SRef. 26. Therefore, we will only focus in this work on Dirac semimetals whose infinite crystals have point group $4mm$, even though our results imply

that even more semimetallic phases exist with HOFA states derived from other variants of QIs and 2D corner-mode phases.

To identify 3D SGs that support Dirac semimetals with QI-nontrivial HOFA states at unpinned rod angles, we will find that, unlike earlier in this section, there is only one independent condition. To see how the three previous conditions from earlier in this section reduce here to a single independent condition, we focus on the group-subgroup relations of the rod, point, and space groups.

To begin, we previously established that, in order for 2D BZ planes in the vicinity of a bulk Dirac point to exhibit the same bulk topology as a QI, the little group along the high-symmetry BZ line of the Dirac point must have $4mm$ or higher symmetry (previous condition 1). Therefore, we require that:

1. The SG of the bulk crystal must have point group $4mm$ or higher.

For the purposes of identifying SGs that support Dirac semimetals with HOFA states, this requirement subsumes the previous condition 1 from earlier in this section, because BZ lines cannot have $4mm$ symmetry if the point group of the SG of the crystal does not contain[19] $4mm$. Specifically, because we will ultimately here obtain a much larger list of SGs than previously obtained earlier in this section (Supplementary Table 7), then we will only focus here on whether or not an SG can support *any* Dirac point with HOFA states, and will not further determine the specific BZ lines along which such a Dirac point can form, as we did previously in Supplementary Table 7.

Under this looser restriction, because point groups $4mm$ and $4/m'mm$ both host pairs of two-dimensional (co)representations with different fourfold rotation eigenvalues, then all of the Γ-point-intersecting BZ lines in SGs with point group $4mm$ or higher also satisfy condition 3 from earlier in this section. Specifically, because translations only act as phases in momentum space[19,56,57,130], then *all* SGs with point group $4mm$ or higher contain at least one fourfold axis in momentum space that intersects the Γ point along which k points have little groups that are either isomorphic to $4mm$ or to $4/m'mm$. For example, taking the fourfold axis to lie along the z direction, whether the x and y (in-plane) reflections of an SG with point group $4mm$ are mirrors or glides, they can still be represented the same way along $k_x = k_y = 0$ (*i.e.* along a line intersecting the Γ point ($\mathbf{k} = \mathbf{0}$)) because in-plane translations are represented as $\exp(ik_{x,y}/b)$ where b depends on the specific SG[19], and because translations along the z axis only act as an overall phase that can be removed by a unitary transformation away from the TRIM points (SEq. (232)). Using the TRIM point labeling in SFig. 18, we identify the BZ lines that always support HOFA Dirac points in crystals in these SGs as ΓZ and ΓM in primitive and body-centered tetragonal crystals, respectively, and ΓX, ΓX, and ΓH in primitive, face-centered, and body-centered cubic crystals, respectively. Additionally we note that, as previously discussed in this section, some SGs host more than one line that satisfies the previous conditions 1 and 3 from earlier in this section (such as MA in SG 123 $P4/mmm1'$). However, we also again note that in many nonsymmorphic SGs, such as SG 100 $P4bm1'$, some BZ lines with $4mm$ symmetry instead exhibit a single four-dimensional corepresentation, due to the commutation relations between the nonsymmorphic crystal symmetries[19,27,56]. In summary, both conditions 1 and 3 from earlier in this section are redundant with the requirement that the SG of the bulk crystal must have point group $4mm$ or higher.

Finally, for a Dirac semimetal whose SG contains point group $4mm$ to exhibit HOFA states, we require that when this semimetal is cut into a rod that is finite in two dimensions and infinite along the direction of its fourfold axis, its rod group contains enough symmetries to preserve the nontrivial topology of its intrinsic (anomalous) HOFA states. As discussed earlier in this section, we previously determined in SN 10 that the minimum symmetry requirement for QI-nontrivial 0D states in 2D is fourfold rotation. When this constraint is applied to HOFA states in 3D rods, it is promoted to the constraint that the rod respects a rod group with a fourfold (rotation or screw) axis. This supersedes the more restrictive previous requirement in condition 2 that the rod respects both a fourfold axis as well as perpendicular in-plane reflections. The requirement that a rod respects a fourfold axis can be reexpressed as the statement that the rod group[23] has a point group that contains point group 4. All of the rod groups with point group 4 or higher are necessarily supergroups of one of the type-I magnetic rod groups[20,23,137] $(p4)_{RG}$, $(p4_1)_{RG}$, $(p4_2)_{RG}$, or $(p4_3)_{RG}$, as those are the lowest-symmetry rod groups that contain fourfold axes. Crucially, we find that *all* SGs with point group $4mm$ or higher are supergroups of these four rod groups, and therefore characterize crystals that can be cut into rods with fourfold axes. Therefore, the requirement that a Dirac semimetal can be cut into a rod with a fourfold axis, which was obtained by relaxing the reflection-symmetry requirement in condition 2 in SN 10, is *also* redundant with the simple restriction in this section to SGs with point group $4mm$ or higher. Furthermore, as discussed in the text surrounding SEq. (232), because the representation of fourfold screw symmetry in the vicinity of Dirac points in screw-symmetric Dirac semimetals can be rotated into the same form as the representation of fourfold rotation in symmorphic Dirac semimetals, then we predict the presence of HOFA states in both symmorphic and nonsymmorphic Dirac semimetals whose SGs have point group $4mm$ or higher. Additionally, later in this section, we will show that body-centered Dirac semimetals with $4mm$ symmetry can generically be cut into rods that preserve a fourfold axis, even though they do not preserve the body-centered lattice vectors of the uncut crystal, and that, consequently, they also exhibit HOFA states. We have therefore shown that that *all* semimetals in SGs with point

group $4mm$ or higher hosting Dirac points along BZ lines with little groups that contain point group $4mm$ or $4/m'mm$ can be cut into nanorod geometries that exhibit anomalous (*i.e.* intrinsic) HOFA states. In Supplementary Table 8 we summarize this result and enumerate the SGs with point group $4mm$ or higher that support Dirac semimetals with HOFA states derived from QIs.

Though in some of the SGs in Supplementary Table 8 (*i.e.* those also listed in Supplementary Table 7), the in-plane reflections coincide with the fourfold axes and do not contain in-plane translations, other SGs (such as SG 100 $P4bm1'$) contain in-plane glide reflections with in-plane lattice translations (*i.e.* $\{M_x|\frac{1}{2}\frac{1}{2}0\}$), which cannot be preserved in a fourfold-symmetric rod geometry. Nevertheless, we can take nanorods in these SGs to be sufficiently thick for the glide symmetries to be approximately preserved in the bulk, such that $4mm$-symmetric Dirac points are only weakly split when in-plane lattice translations are broken in the finite-sized rod geometry. Specifically, while the angle ϑ by which the HOFA states are rotated is uncontrollably large (SFig. 15), the weak breaking of glide symmetry still preserves the bulk band ordering, guaranteeing that the HOFA states remain an intrinsic consequence of the bulk Dirac points.

We finally note that, more generally, breaking in-plane reflections in the bulk while preserving a fourfold axis will split a $4mm$-symmetric Dirac point into Weyl points whose arrangement and Chern numbers are restricted by fourfold rotation symmetry[142] (conversely, the Dirac points can be fully gapped by breaking fourfold rotation symmetry in the bulk). Most interestingly, this implies that if a fourfold-symmetric Weyl semimetal can be deformed into a Dirac semimetal with HOFA states, then it will also exhibit intrinsic HOFA states at free angles *coexisting* with topological surface Fermi arcs if the Dirac point was split into a pair of Weyl points along the fourfold axis. Specifically, in this Weyl semimetal, taking k_z to lie along the fourfold axis and considering 2D BZ planes at increasing values of k_z, one will pass first from a trivial insulator into a 2D Chern insulating phase when k_z is increased through the first Weyl point. Then, continuing to increase k_z, when one passes over the second Weyl point, one will pass from the Chern insulating phase into a 2D corner-mode phases that exhibits a free-angle quadrupole moment (SN 10). In the Chern insulating phase, the surface Fermi arcs are equivalent to the chiral edge states of the intermediate Chern insulating phases separating trivial and QI phases in wallpaper group $p4$ discussed in SN 10. Because the band connectivity, topology, and Fermi surfaces of this HOFA *Weyl semimetal* are considerably more complicated than those of the HOFA Dirac semimetals introduced in this work, then we leave the analysis and complete enumeration of HOFA Weyl semimetals for future works.

HOFA States in Body-Centered Dirac Semimetals – When a body-centered Dirac semimetal is cut into a rod that preserves a fourfold axis, as was prescribed in earlier in this section to observe HOFA states, the lattice vector of the finite-sized rod (*i.e.* the direction and length along which it is periodic) cannot coincide with the original lattice vectors of the bulk crystal, because fourfold axes and lattice vectors do not coincide in body-centered SGs[19]. Therefore, one might be concerned that HOFA states do not appear on the rod, due to zone-folding effects similar to those that negate the presence of edge states on armchair-terminated graphene[51–55], which we will review in this section. We make this analogy to graphene, because the flat-band-like Fermi arc states in graphene can be considered the "first-order" analogs of the HOFA states analyzed in this work. However, we will show in this section that body-centered tetragonal and cubic Dirac semimetals still generically exhibit HOFA states, because their Dirac points are free to shift along high-symmetry lines, whereas the Dirac points in graphene are pinned to high-symmetry BZ points.

First, we will review how geometry and periodicity explain the absence of flat-band-like edge Fermi arcs in armchair-terminated graphene. Then, by explicitly performing the BZ folding from a Dirac semimetal in a body-centered SG to a rod that preserves a fourfold axis, we will show that HOFA states are still generically present on the rod hinges.

We begin by reviewing graphene, a two-dimensional honeycomb carbon lattice[51,55]. The electronic structure of graphene is characterized by two spin-degenerate Dirac cones lying at the high-symmetry BZ points[51] K and K' (SFig. 19(b)). Graphene sheets can be terminated with either zigzag or armchair edges (SFig. 19(a)). It has been extensively demonstrated that while zigzag edges exhibit nearly flat surface Fermi arcs, armchair edges do not exhibit low-energy boundary modes[52–55]. To explain this phenomenon, we employ arguments from SRef. 55, and analyze the relationship between edge and bulk periodicity. On zigzag edges, the edge lattice vector ($t_{\bar{x}}$) is the same length and direction as the bulk lattice vector t_2. Consequently, in the edge BZ (SFig. 19(b)), the bulk Dirac points at K and K', which have different k_x momentum coordiantes, project to different points. Due to the presence of bulk mirror and inversion symmetries, their projections are spanned by Fermi arcs "protected" by the dipole moments of the effective 1D Hamiltonians along the BZ lines that project to them[52–55]. Conversely, in armchair-terminated graphene, the edge lattice vector $t_{\bar{y}}$ (SFig. 19(a)) spans multiple unit cells; specifically, $t_{\bar{y}} = 2t_1 + t_2$. Consequently, in the armchair edge BZ (SFig. 19(b)), the edge reciprocal lattice vector $k_{\bar{y}}$ is shorter than the bulk reciprocal lattice vector k_1, and the K and K' points project to the *same* point: the edge TRIM point $\bar{\Gamma}$. Therefore, there is no region in the armchair edge BZ spanning the projections of the bulk Dirac points, and thus armchair terminations do not exhibit edge states at low energies[52–55].

We can extend the same arguments to 3D body-centered crystals to calculate the hinge projections of bulk Dirac points capable of supporting HOFA states. In Supplementary Tables 7 and 8, we show that Dirac points with HOFA

states may only form in body-centered crystals along the fourfold axis ΓZ (ΓM) in tetragonal SGs (SFig. 18(a) and (b), respectively), and along the fourfold axes ΓX (ΓH) in cubic SGs (SFig. 18(c,d) and (e), respectively). Therefore, following the arguments employed earlier in this section, HOFAs may form on the hinges of z-directed rods of semimetals in these SGs (and, up to equivalence, on the hinges of rods oriented along the x, y, and z directions in cubic systems). However, in body-centered tetragonal and cubic SGs, the k_z axis is not parallel to the lattice vectors[19,30,31]. Specifically, in tetragonal SGs, the fourfold axis lies along the z direction, whereas the reciprocal lattice vectors are:

$$\mathbf{k}_1 = \left(0, \frac{2\pi}{a}, \frac{2\pi}{c}\right),\ \mathbf{k}_2 = \left(\frac{2\pi}{a}, 0, \frac{2\pi}{c}\right),\ \mathbf{k}_3 = \left(\frac{2\pi}{a}, \frac{2\pi}{a}, 0\right). \tag{233}$$

Therefore when a crystal with a body-centered tetragonal or cubic SG is cut into a rod that preserves a fourfold axis and exhibits a lattice periodicity of c in the z direction, distinct points within the bulk BZ will be folded onto the same point in the rod BZ, analogous to armchair-terminated graphene (SFig. 19(b)).

We show in SFig. 20(b,c) the bulk BZ planes that project to hinge TRIM points (SFig. 20(d)); note that the Γ and M points in successive BZs lie in the same planes at $k_z = 0, 2\pi/c$, where c is the lattice spacing in the c direction (though in the bulk crystal, $2\pi/c$ is not a reciprocal lattice vector (SEq. (233))). Therefore, all of the points in these planes project to the hinge TRIM point $\bar{\Gamma}$ (SFig. 20(d)). The N and P points and their time-reversal and fourfold rotation partners N' and P' also lie in the same bulk BZ planes at $k_z = \pm\pi/c$ (SFig. 20(c)), and all project to the same hinge TRIM point $\bar{Z}$. Therefore, if a Dirac point along ΓM lies *exactly* at π/c, its time-reversal partner will also lie in the same plane in the next BZ, and both Dirac points will project to $\bar{Z}$. This situation is analogous to the armchair termination of graphene (SFig. 19(b)): because both Dirac points project to the same point in the hinge BZ, there is no region between their projections for HOFA states to span, and so HOFAs will not be present. However, as the SGs in Supplementary Table 8 do not contain additional symmetries that can force Dirac points to lie exactly at $k_z = \pi/c$, it is extremely unlikely for band-inversion-driven[56,110] Dirac points in real materials to lie in these planes. Therefore, HOFAs should still be generic features of fourfold-symmetric rods of body-centered Dirac semimetals with the SGs listed in Supplementary Table 8.

The difference between armchair-terminated graphene and body-centered HOFA Dirac semimetals can be summarized by recognizing that the 3D HOFA Dirac points analyzed in this section arise from band inversion, and are thus free to shift in momentum along high-symmetry BZ lines (they are "enforced semimetals" in nomenclature of SRef. 136), whereas, conversely, the Dirac points in graphene are pinned by band connectivity to the high-symmetry BZ points[24,51] K and K' (graphene is an "enforced semimetal with Fermi degeneracy" in the nomenclature of SRef. 136). For example, if the Dirac points in a body-centered 3D semimetal were instead hypothetically characterized by four-dimensional corepresentations[138] pinned to the P and P' points (SFig. 20(a,c)), then they would lie exactly at $k_z = \pm\pi/c$, and would not exhibit HOFA states (SFig. 20(d)).

13. First-Principles Calculation Details

HOFA States in KMgBi – Among the previously synthesized materials that fulfill the criteria for HOFAs derived in SN 12 (Supplementary Table 8), we identify the candidate HOFA Dirac semimetal KMgBi in SG 129 ($P4/nmm1'$)[107–109] (Inorganic Crystal Structure Database (ICSD)[135] No. 616748, further details available at[24,30,31,136] `https://topologicalquantumchemistry.org/#/detail/616748`). We calculate the electronic structure of KMgBi from first principles with the projector augmented wave (PAW)[143] method as implemented in the VASP package[144,145] (SFig. 21(a,b) and Fig. 4 of the main text). In KMgBi, the Bi atoms occupy the 2c Wyckoff position and each exhibit an oxidation state of 3−; this implies that all 6 occupied bands near the Fermi energy (E_F) arise from Bi p orbitals in the limit of vanishing spin-orbit coupling (SOC) (SFig. 21(a)). As there are two Bi atoms per unit cell, these p-orbitals can form bonding and anti-bonding states. In SG 129, the Γ and Z points have little co-groups isomorphic to point group $4/mmm1'$ (D_{4h}), and points along the line ΓZ have little groups isomorphic to magnetic point group $4/m'mm$ (SN 3). We therefore label bands at Γ and Z using the notation employed in SN 3 (SEqs. (31) and (32)) and SN 5 (SEq. (81)) for the irreducible corepresentations of $4/mmm1'$ (SEqs. (33), (83), and (82)). In this notation, ρ_i indicates the ith irreducible representation of $4mm$, the $\mathcal{I}$- (and $\mathcal{I}\times\mathcal{T}$-) broken unitary subgroup of $4/mmm1'$ (and $4/m'mm$); bars indicate double-valued (co)representations; and the corepresentations of $4/mmm1'$ at Γ and at Z are labeled with additional $\pm$ superscripts to indicate whether they have positive or negative parity ($\mathcal{I}$) eigenvalues. Specifically, the numbering for ρ_i is chosen to match the order of irreducible representations displayed in the REPRESENTATIONS: DBG tool on the BCS[24,57] for the Γ point of the unitary subgroup of SG 99 $P4mm1'$, which is isomorphic to $4mm$.

At Γ and Z, in the absence of SOC, the bonding and antibonding states nearest the Fermi energy are characterized by the single-valued corepresentations ρ_1^+ and ρ_5^-, respectively[40,57,58] (SFig. 21(a)). In terms of the corepresentations

of point group[40] $4/mmm1'$:

$$\rho_1^+ \equiv A_{1g},\ \rho_5^- \equiv E_u. \tag{234}$$

In first-principles calculations incorporating the effects of SOC, SOC drives a band inversion in KMgBi at Γ between states labeled by double-valued corepresentations with opposite inversion characters (parity eigenvalues). Specifically, after this SOC-driven band inversion, the states just above and below E_F at Γ are characterized by $\bar{\rho}_6^-$ and $\bar{\rho}_7^+$, respectively (SFig. 21(b)), whose C_{4z} and $\mathcal{I}$ inversion characters are respectively given by:

$$\chi_{\bar{\rho}_6^-}(C_{4z}) = \frac{-1+i}{\sqrt{2}} + \frac{-1-i}{\sqrt{2}} = -\sqrt{2},\ \chi_{\bar{\rho}_7^+}(C_{4z}) = \frac{1+i}{\sqrt{2}} + \frac{1-i}{\sqrt{2}} = \sqrt{2},\ \chi_{\bar{\rho}_6^-}(\mathcal{I}) = -2,\ \chi_{\bar{\rho}_7^+}(\mathcal{I}) = 2, \tag{235}$$

where $\chi_\rho(g)$ is the character of the unitary symmetry g in the irreducible representation ρ, and is equivalent to the trace of the matrix representation of g (*i.e.*, the sum of the symmetry eigenvalues of g in ρ). In terms of the corepresentations of point group[40] $4/mmm1'$:

$$\bar{\rho}_6^- \equiv \bar{E}_{2u},\ \bar{\rho}_7^+ \equiv \bar{E}_{1g}. \tag{236}$$

Because, SOC drives bands with opposite parity eigenvalues to become inverted at Γ, the Hamiltonian of the $k_z = 0$ plane of KMgBi is topologically equivalent to a 2D TI, as occurs in many other topological semimetals[32], such as Na_3Bi[34,35,104–106,146], both the room- (α) and intermediate-temperature (α'') phases of Cd_3As_2[32,110,111,147,148], and WC[149]. Specifically, incorporating the effects of SOC in KMgBi, the product of the parity eigenvalues per Kramers pair up to the Fermi energy is positive at Γ and negative at X, X', and M. The Hamiltonian of the $k_z = 0$ plane is therefore $\mathbb{Z}_2$-nontrival by the Fu-Kane parity index[38]. As shown in SFig. 21(b), because the bands that cross along ΓZ are labeled by $\bar{\rho}_6^-$ and $\bar{\rho}_7^+$ at the TRIM points, then the Hamiltonian of the $k_z = 0$ plane *also* exhibits the same fourfold rotation eigenvalues as a QI in $p4m$ (SN 3 and 7). This indicates that, at intermediate values of k_z, bands must be labeled by $\bar{\rho}_{6,7}$, and implies that their crossing (Dirac) points must exhibit HOFA states when projected to the hinges of fourfold-symmetric nanorods (SN 5 and 12).

To demonstrate the presence of hinge-localized HOFAs in KMgBi, we form a $p4m$, $\mathcal{I}$-, and $\mathcal{T}$-symmetric, four-band $k \cdot p$ theory near the Γ point. We choose the basis in which bands characterized by $\bar{\rho}_7^+$ are labeled $|s, \uparrow\rangle$ and $|s, \downarrow\rangle$, and bands characterized by $\bar{\rho}_6^-$ are labeled $|p_x + ip_y, \uparrow\rangle$ and $|p_x - ip_y, \downarrow\rangle$. It is important to note that, because we are here using $p_{x,y}$ orbitals, and not p_z orbitals, then the spinful states labeled by $|p_x + ip_y, \uparrow\rangle$ and $|p_x - ip_y, \downarrow\rangle$ exhibit the same fourfold rotation eigenvalues (but not the same parity eigenvalues) as spinful $d_{x^2-y^2}$ orbitals (SN 3, 7, and 8); therefore, as shown in SN 7, we expect SEq. (237) to exhibit the same HOFA states as the $p-d$-hybridized Dirac semimetal in SN 5. Using these four states, we formulate a $k \cdot p$ Hamiltonian that is the same to quadratic order as the one introduced in SRef. 110 for the Dirac points in the centrosymmetric phases of Cd_3As_2 (later in this section, we will detail calculations showing HOFA states in α''-Cd_3As_2):

$$H_\Gamma(\mathbf{k}) = \epsilon_0(\mathbf{k}) + \begin{pmatrix} M(\mathbf{k}) & Ak_+ & 0 & B^*(\mathbf{k}) \\ Ak_- & -M(\mathbf{k}) & B^*(\mathbf{k}) & 0 \\ 0 & B(\mathbf{k}) & M(\mathbf{k}) & -Ak_- \\ B(\mathbf{k}) & 0 & -Ak_+ & -M(\mathbf{k}) \end{pmatrix},$$

where:

$$\begin{aligned} \epsilon_0(\mathbf{k}) &= C_0 + C_1k_z^2 + C_2(k_x^2 + k_y^2),\ k_\pm = k_x \pm ik_y,\ B(\mathbf{k}) = B_1k_z^ck_+^2 + B_2k_z^ck_-^2, \\ M(\mathbf{k}) &= M_0 - M_1k_z^2 - M_2(k_x^2 + k_y^2). \end{aligned} \tag{237}$$

We chose $M_0, M_1, M_2 < 0$ to reproduce the band inversion, and set $\epsilon_0(\mathbf{k}) \to 0$ for simplicity. Using our first-principles calculations, we predict that the two Dirac points in KMgBi are located at $(0, 0, \pm k_z^c)$ with $k_z^c = 0.06085\ (2\pi/c) = 0.046\ \mathring{A}^{-1}$. Using the energy ordering of the irreducible representations $\bar{\rho}_{6,7}$ (SN 3 and 5) and the nested Wilson loop[26,28] (SFig. 21(d)), we deduce that k_z slices with topological quadrupole moments and hinge-localized HOFA states occur between $0 < |k_z| < k_z^c$. Fitting the model in SEq. (237) to the calculated electronic structure (SFig. 21(c)), we obtain the $k \cdot p$ parameters listed in Supplementary Table 9.

We then map this fitted $k \cdot p$ theory to a lattice tight-binding model, following the procedure employed in SRef. 110. Next, we cut the lattice tight-binding model into a fourfold-symmetric (square) rod that is finite in the x and y directions and infinite in the z direction and calculate its bulk, surface, and hinge states (SFig. 22); in particular, we observe that the four hinge states closest to the Fermi energy are half-filled, in agreement with the characterization of the corner modes of a QI in SFig. 9. However, because the spinful rod groups do not host symmetry-stabilized four-dimensional corepresentations away from rod TRIM points[19,57], then there is no crystal symmetry that can force

the bands corresponding to the bulk Dirac points and HOFA hinge states to appear in strict fourfold degeneracies in the rod bands in SFig. 22; instead, their fourfold degeneracy is only restored in the thermodynamic limit that states along the rod do not hybridize. Therefore, because of finite-size effects, the hinge projections of the bulk Dirac points and the HOFA hinge states split into two sets of states in the rod bands in SFig. 22.

Next, to visualize the HOFA state that appears on a single hinge of a macroscopic, z-directed sample of KMgBi, we perform a hinge Green's function calculation, the results of which are shown in Fig. 4(d) of the main text. To isolate the surface and corner (hinge) states, we first form a slab of the tight-binding model in SEq. (237) that is infinite in the z direction, semi-infinite in the x direction, and large (~ 60 unit cells) in the y direction. We then employ *hinge* Green's functions to calculate the hinge states localized on just a single edge of the semi-infinite slab (Fig. 4(d) of the main text). We observe clear HOFA states connecting the hinge projections of the bulk 3D Dirac points to the hinge projections of the 2D topological surface cones of the $\mathbb{Z}_2$-nontrivial bulk plane at $k_z = 0$ (Fig. 4(d) of the main text). Unlike in our previous rod calculation (SFig. 22), the semi-infinite slab used to calculate the hinge Green's function exhibits strongly broken fourfold rotation symmetry, and is therefore not strictly required to exhibit topological HOFA states, which may be removed through surface gap closures, analogous to the corner modes of the C_{4z}-broken QI in SRef. 26. Nevertheless, in the Green's function of a single hinge (Fig. 4(d) of the main text), we still observe the same, isolated, half-filled HOFA state as is present on each of the four hinges in the fourfold-symmetric rod tight-binding calculation (SFig. 22) that we performed to confirm the presence of intrinsic HOFA states in KMgBi. We postulate that this is because the slab still has 90-degree corners, like in the square rod calculation (SFig. 22), and because it is very (infinitely) large in the xy-plane compared to the hoppings in SEq. (237); therefore, at most (if not all) k_z points with HOFA states in the hinge Green's function calculation (Fig. 4(d) of the main text), a very large suface potential may be required to change the sign of the surface (edge) gap from its value in the fourfold-symmetric rod calculation (SFig. 22).

HOFA States in α''-Cd_3As_2 – We also find that the criteria for HOFAs in SN 12 (Supplementary Table 8) are satisfied by the archetypal Dirac semimetal Cd_3As_2 in both its room- (α) and intermediate-temperature (α'') phases (SGs 142 ($I4_1/acd1'$) and 137 ($P4_2/nmc1'$), respectively)[110,111,113,150]. Because of its simple primitive tetragonal Bravais lattice, we here focus on α''-Cd_3As_2, though the calculations performed in this section could also be adapted to characterize the HOFA states in α-Cd_3As_2 after carefully mapping its body-centered lattice to a primitive tetragonal rod (SN 12).

The α'' phase of Cd_3As_2 (ICSD[135] No. 609930) has been extensively studied in theoretical works[110,113,150], and has been stabilized in experiment in single crystalline form at room temperature and below by 2% zinc doping[114]. Furthermore, as Zn is isoelectronic to Cd, this doping should not affect the Fermi level. In SFig. 23(b,a), we show the electronic structure of α''-Cd_3As_2 calculated from first principles with and without incorporating the effects of SOC, respectively, obtained using the same methodology previously employed in this section to calculate the electronic structure of KMgBi. As in KMgBi, the little co-group of the Γ point is isomorphic to point group $4/mmm1'$ (D_{4h}) (though unlike in KMgBi, the little co-group of the Z point is here *not* isomorphic to a point group[19,56,130], because of the projective action of the fractional lattice translation in the 4_2 screw symmetry in SG 137 $P4_2/nmc1'$). The representation labels of the bands in SFig. 23(a,b) were obtained from first principles, are given in the convention previously established earlier in this section for the bands of KMgBi, and agree with the results of previous investigations[110,111,113,150] of α''-Cd_3As_2. In terms of the corepresentations of point group[40] $4/mmm1'$, the single-valued corepresentations in SFig. 23(a) are related by the equivalences:

$$\rho_1^+ \equiv A_{1g},\ \rho_3^- \equiv A_{2u},\ \rho_5^- \equiv E_u. \tag{238}$$

When the effects of SOC are incorporated, the corepresentations at Γ are labeled by $\bar{\rho}_6^-$, $\bar{\rho}_7^+$, where the s_{4_2z} and $\mathcal{I}$ characters of ρ_6^- and $\bar{\rho}_7^+$ were previously given in SEq. (235) (s_{4_2z} exhibits the same set of eigenvalues and commutation relations with other spatial symmetries as C_{4z} at the Γ point of any SG[19]), and where the s_{4_2z} and $\mathcal{I}$ characters of $\bar{\rho}_7^-$ are:

$$\chi_{\bar{\rho}_7^-}(s_{4_2z}) = \frac{1+i}{\sqrt{2}} + \frac{1-i}{\sqrt{2}} = \sqrt{2},\ \chi_{\bar{\rho}_7^-}(\mathcal{I}) = -2. \tag{239}$$

In terms of the double-valued corepresentations of point group[40] $4/mmm1'$:

$$\bar{\rho}_6^- \equiv \bar{E}_{2u},\ \bar{\rho}_7^+ \equiv \bar{E}_{1g},\ \bar{\rho}_7^- \equiv \bar{E}_{1u}. \tag{240}$$

Away from the TRIM points Γ and Z, bands along ΓZ cross to form a time-reversed pair of symmetry-stabilized Dirac points at $k_x = k_y = 0$, $k_z = \pm k_z^c$, where $k_z^c = 0.125\ (2\pi/c)$ (SFig. 23(b)), where the crossed bands are labeled by $\bar{\rho}_{6,7}$ of $4mm$ with an additional $\mathcal{I} \times \mathcal{T}$ symmetry whose symmetry-representation commutation relations contain phases that reflect the 4_2 screw symmetry in SG 137 $P4_2/nmc1'$ (SN 3 and 12). Furthermore, α''-Cd_3As_2 crystals

in SG 137 are theoretically capable of being shaped into nanowires with fourfold axes, as discussed in the caption of and text surrounding Supplementary Table 8 (indeed nanowires[151] and z- (c-axis-) directed samples[152] of the room-temperature (α) phase of Cd_3As_2 in SG 142 ($I4_1/acd1'$) have already been synthesized in experiment). Therefore, as shown in SN 5 and 12, a c-axis-directed, 4_2-screw-symmetric nanowire of α''-Cd_3As_2 should exhibit HOFA states on its 1D hinges.

To demonstrate the presence of HOFA states in α''-Cd_3As_2, we follow the same procedure previously employed for KMgBi. We introduce the $k \cdot p$ Hamiltonian in SEq. (237) to model the electronic structure of α''-Cd_3As_2 calculated from first principles (SFig. 23(b)). Unlike previously with KMgBi, there is an additional subtlety in using SEq. (237) to model the low-energy electronic structure of α''-Cd_3As_2. As shown in SFig. 23(b) and discussed in detail in SRef. 110, the electronic structure of α''-Cd_3As_2 features multiple band inversions and split representations at the Γ point: specifically, after incorporating the effects of SOC, the inverted bands closest to the Fermi energy are labeled by corepresentations with different s_{4_2z} eigenvalues and the same parity eigenvalues ($\bar{\rho}^-_{6,7}$), and there is a second, larger band inversion, also at Γ, between bands with opposite parity eigenvalues (the valence band of which is labeled with $\bar{\rho}^+_7$). To simplify the description of the bands at the Fermi energy, we follow the procedure developed in SRef. 110, and use a four-band tight-binding model in which the valence states at Γ closest to the Fermi energy ($\bar{\rho}^-_7$) in SFig. 23(b) are replaced with states labeled by $\bar{\rho}^+_7$, reflecting the summed parity and fourfold rotation eigenvalues of the entire valence manifold at Γ. As shown in SRef. 110, this simplified description still captures the band inversion in α''-Cd_3As_2 between Kramers pairs of Cd $5s$ orbitals and the $m_j = \pm 3/2$ subset of spinful As $4p_{x,y}$ orbitals, because spinful $m_j = \pm 3/2$ p orbitals (*i.e.* $p_x + ip_y, \uparrow$ and $p_x - ip_y, \downarrow$ orbitals) were previously shown in this section to exhibit the same fourfold rotation eigenvalues (but not the same parity eigenvalues) as spinful $d_{x^2-y^2}$ orbitals (SN 3, 7, and 8).

Next, we fit the bands of the simplified $k \cdot p$ theory in SEq. (237) to the first-principles electronic structure of α''-Cd_3As_2 (SFig. 23), obtaining the fitting parameters listed in Supplementary Table 10. We then map this fitted $k \cdot p$ theory to a lattice tight-binding model, which we place on a fourfold-symmetric rod finite in the x and y directions, and calculate its surface and hinge states (SFig. 24). In α''-Cd_3As_2, it is well documented that the Hamiltonian of the $k_z = 0$ plane is equivalent to a 2D TI[32,111,148], whose edge states correspondingly manifest as rod surface states at $k_z = 0$. In both the complete DFT description of α''-Cd_3As_2 (SFig. 23(b)) and in SEq. (237) with the fitting parameters listed in Supplementary Table 10, the Hamiltonian of the $k_z = 0$ plane *also* exhibits the same fourfold rotation eigenvalues as a QI in $p4m$ (SN 3 and 7). As shown in SN 7, this indicates that the TI surface states at $k_z = 0$ will gap into HOFA hinge states away from $k_z = 0$. In our rod calculation, we also observe four, half-filled hinge states connecting the hinge projections of the bulk 3D Dirac points to the projections of the 2D TI cones at $k_z = 0$. Finally, using the same semi-infinite slab construction previously employed for KMgBi, we use hinge Green's functions to calculate the states on a single hinge of a large, z-directed crystal of α''-Cd_3As_2, the results of which are shown in Fig. 4(c) of the main text. The hinge spectrum exhibits clear HOFA states connecting the hinge projections of the bulk 3D Dirac points to the hinge projections of the 2D topological surface cones of the 2D-TI-equivalent bulk plane at $k_z = 0$.

HOFA States and Fragile Corner Modes in β'-PtO_2 – Finally, we also use first-principles and tight-binding calculations to demonstrate the presence of HOFA states and related fragile-phase corner modes in the candidate Dirac semimetal[153–156] PtO_2 in its rutile-structure (β') phase (SG 136 ($P4_2/mnm1'$), ICSD[135] No. 647316, further details available at[24,30,31,136] `https://topologicalquantumchemistry.org/#/detail/647316`), which satisfies the criteria for HOFA states derived in SN 12. In β'-PtO_2, the Pt atoms occupy the $2a$ Wyckoff position and the O atoms occupy the $4f$ position. In SFig. 25(b,a), we respectively show the electronic structure of β'-PtO_2 with and without incorporating the effects of SOC, calculated using the same methodology previously employed for KMgBi. Even before incorporating the effects of SOC, spin-degenerate bands in β'-PtO_2 are already strongly inverted at Γ. Specifically, the valence and conduction bands in β'-PtO_2 (SFig. 25(a,b)) only cross in the vicinity of inverted bands at the Γ point, though there are also additional electron and hole pockets from bands that do not connect across the gap nearest the Fermi energy. In the absence of SOC, the inverted states at Γ are labeled by the single-valued corepresentations $\rho^+_{3,4}$ of the little co-group of the Γ point of SG 136, which is isomorphic to point group $4/mmm1'$ (D_{4h}), and where corepresentations are labeled using the convention previously established earlier in this section. In terms of the corepresentations of point group[40] $4/mmm1'$, the single-valued corepresentations in SFig. 25(a) are related by the equivalences:

$$\rho^+_3 \equiv A_{2g},\ \rho^+_4 \equiv B_{2g}. \qquad (241)$$

When the effects of SOC are incorporated (SFig. 25(b)), the nodal lines near Γ split into a time-reversed pair of Dirac points located at $\mathbf{k} = (0, 0, \pm k^c_z)$, where $k^c_z = 0.1663\ (2\pi/c) = 0.33386$ Å^{-1}. In the electronic structure of β'-PtO_2 incorporating SOC, the bands closest to the Fermi energy at Γ are labeled by $\bar{\rho}^+_{6,7}$, whose s_{4_2z} and $\mathcal{I}$ characters were previously listed in SEqs. (235) and (239), and which are related to the corepresentations of $4/mmm1'$ through the

equivalences:

$$\bar{\rho}_6^+ \equiv \bar{E}_{2g},\ \bar{\rho}_7^+ \equiv \bar{E}_{1g}, \tag{242}$$

again noting, as we did previously in this section for α''-Cd_3As_2, that s_{4_2z} exhibits the same set of eigenvalues and commutation relations with other spatial symmetries as C_{4z} at the Γ point of any SG[19].

Unlike in the previous HOFA Dirac semimetals analyzed in this section KMgBi and α''-Cd_3As_2, because the Dirac points in β'-PtO_2 originate from a single band inversion between bands with the same parity eigenvalues (SEqs. (235) and (239)), the Hamiltonian of the $k_z = 0$ plane is not equivalent to a 2D TI. Instead, because the inverted bands exhibit different fourfold rotation eigenvalues (and the same parity eigenvalues), the Hamiltonian of the $k_z = 0$ plane of β'-PtO_2 is equivalent to a $C_{M_z} = 2$ mirror TCI[32,33,60,154] (SEq. (94)). As in the model of an $s-d$-hybridized 3D Dirac semimetal introduced in this work (SEqs. (1) and (6)), the nontrivial mirror Chern number $C_{M_z} = 2$ of the Hamiltonian of the $k_z = 0$ plane necessitates the presence of two, twofold degenerate TCI cones at $k_z = 0$ on M_z-preserving surfaces (SFig. 8(b,c)). We note that the Hamiltonian of the $k_z = 0$ plane *also* exhibits the same fourfold rotation eigenvalues as a QI in $p4m$ (SN 3 and 9); as shown in SN 9, this indicates that the TCI surface states at $k_z = 0$ will gap into HOFA hinge states away from $k_z = 0$.

To calculate the HOFA states in β'-PtO_2, we first form a 4×4 $k \cdot p$ Hamiltonian of the bands closest to the Dirac points at the Fermi energy. Because the inverted bands in β'-PtO_2 have different parity and fourfold rotation eigenvalues than in KMgBi and α''-Cd_3As_2, then we cannot employ the previous model of SEq. (237). Instead, we begin by defining a different four-band basis in which the two states labeled with $\bar{\rho}_7^+$ ($\bar{\rho}_6^+$) are denoted as $|\pm\frac{1}{2}\rangle$ ($|\pm\frac{3}{2}\rangle$), which we summarize in a Pauli-matrix notation in which τ indexes orbital components (*i.e.* $J = \frac{1}{2}, \frac{3}{2}$) and σ indexes spin components (*i.e.*, $\mathrm{sgn}(m_j) = \mathrm{sgn}(\pm\frac{1}{2})$ or $\mathrm{sgn}(\pm\frac{3}{2})$). In this basis, the four-band $k \cdot p$ Hamiltonian of the bands closest to the Fermi energy in β'-PtO_2 takes the form:

$$H_\Gamma(\mathbf{k}) = \epsilon(\mathbf{k})\mathbb{1}_{\tau\sigma} + M(\mathbf{k})\tau^z + A(k_x^2 - k_y^2)\tau^x + \tau^y(Bk_xk_y\sigma^z + Ck_yk_z\sigma^x + Ck_xk_z\sigma^y), \tag{243}$$

where $\mathbb{1}_{\tau\sigma}$ is the 4×4 identity and:

$$\epsilon(\mathbf{k}) = \epsilon_0 + \epsilon_1 k_z^2 + \epsilon_2(k_x^2 + k_y^2),\ M(\mathbf{k}) = M_0 + M_1 k_z^2 + M_2(k_x^2 + k_y^2). \tag{244}$$

In particular, SEq. (243) is identical to the $k \cdot p$ Hamiltonian of the Γ point of the four-band model of an $s-d$ hybridized Dirac semimetal in SG 123 $P4/mmm1'$ introduced in this work (SEqs. (1) and (6)), and therefore satisfies the symmetry representation used throughout this work (Table 1 of the main text). To model the electronic structure of β'-PtO_2, we map the $k \cdot p$ theory in SEq. (243) to a lattice tight-binding model, which we then fit to the electronic structure of β'-PtO_2. From this, we obtain the fitting parameters listed in Supplementary Table 11.

Next, to identify and characterize the hinge states in β'-PtO_2, we follow the same procedure employed for the previous HOFA Dirac semimetals analyzed in this section. First, we calculate the bands of a fourfold-symmetric, z-directed rod of the lattice tight-binding model obtained from SEq. (243). In agreement with analysis performed throughout this work, when we fix the system filling to that of the Dirac points, we observe in SFig. 26(a) a single, half-filled set of four HOFA states connecting the hinge projections of the bulk Dirac points to the projections of the 2D TCI cones at $k_z = 0$ (which have been gapped by finite-size effects, as indicated by the blue arrows in SFig. 26(a)), as well as a second set of empty HOFA states above them in energy. As previously discussed earlier in this section, because the spinful rod groups do not host symmetry-stabilized four-dimensional corepresentations away from rod TRIM points[19,57], then there is no crystal symmetry that can force the HOFA hinge states to appear in strict fourfold degeneracies in the rod bands in SFig. 26; instead, their fourfold degeneracy is only restored in the thermodynamic limit that states along the rod do not hybridize. Therefore, because of finite-size effects, the eight HOFA hinge states in SFig. 26(a) split into four sets of two states, which are labeled, in increasing energy, with red, blue, orange, and black.

To isolate the states on a single hinge, we then calculate the hinge Green's function of a semi-infinite slab, which we plot in Fig. 5(b) of the main text. At each hinge k point between $|k_z| = 0, k_z^c$ we observe two narrowly split hinge states with an overall one-quarter filling. This agrees with the analysis performed in SN 9. Specifically, the hinge spectrum at each k point with HOFA states in Fig. 5(b) of the main text represents the particle-hole conjugate of the particle-hole-broken QI corner spectrum in SFig. 14(c).

Crucially, unlike the surface 2D TI cones in KMgBi and α''-Cd_3As_2, the surface 2D TCI cones in β'-PtO_2 are only protected by M_z symmetry, and therefore can be gapped without breaking $\mathcal{T}$ symmetry. As shown in the main text and discussed in detail in SN 4 and 9, breaking M_z while keeping fourfold rotation, in-plane reflection, and $\mathcal{T}$ symmetries gaps the surface cones of a $C_{M_z} = 2$ 2D TCI formed from band inversion at Γ (such as the $k_z = 0$ plane of β'-PtO_2) into the Kramers pairs of corner modes of a fragile topological phase. Because the corner states are a manifestation of an anomalous absence of ($2 + 8n$ or $6 + 8n$) states from the valence manifold in the $k_z = 0$ plane,

and are a property of the inverted (fragile) bands near the Fermi energy (SN 9), then they *remain* present even when trivial bands below the Fermi energy are added to trivialize the fragile valence manifold. To gap the surface TCI cones in our model of β'-PtO_2, we introduce a term that breaks M_z and $\mathcal{I}$ symmetries while preserving fourfold rotation, in-plane reflection, and $\mathcal{T}$ symmetries:

$$H_{\Gamma}^{el}(\mathbf{k}) = H_{\Gamma}(\mathbf{k}) + D\tau^z(\sigma^x k_y - \sigma^y k_x), \tag{245}$$

where $H_{\Gamma}(\mathbf{k})$ is given in SEq. (243). The D term in SEq. (245) can be induced in experiment by directing an external electric field that is spatially constant (or slowly varying on the scale of the lattice spacing) along the z- (c-) axis of a 4_2-screw-symmetric nanorod of β'-PtO_2. To demonstrate the presence of fragile-phase corner charges in β'-PtO_2 in an external field, we set $D = 0.04$ (eV Å^{-1}) in SEq. (245) and again calculate the bands of a fourfold-symmetric rod and the hinge Green's function of a semi-infinite slab. In the rod calculation (SFig. 26(b)), we observe that the electric field term in SEq. (245) shifts in energy the four Kramers pairs of hinge states (blue circles in SFig. 26(b) at $k_z = 0$) that resulted from gapping the TCI surface states at $k_z = 0$ (the surface states were already largely split by finite-size effects in (a)); only two of the eight hinge states at $k_z = 0$ (the red states) are filled when the system filling is fixed to that of the Dirac points. Even though our rod calculation preserves fourfold rotation and $\mathcal{T}$ symmetry, because the spinful rod groups do not host symmetry-stabilized eight-dimensional corepresentations[19,57], then there is no crystal symmetry that can force the bands corresponding to the eight Kramers pairs of fragile-phase corner modes at $k_z = 0$ in SFig. 26(b) to appear in a strict eightfold degeneracy; instead, their eightfold degeneracy is only restored in the thermodynamic limit that states along the rod do not hybridize.

Finally calculating the hinge Green's function of a z-directed macroscopic sample of β'-PtO_2 in the presence of a z-directed electric field (Fig. 5(c) of the main text), we observe well-isolated, quarter-filled fragile-phase corner modes at $k_z = 0$ connected to narrowly split pairs of half-filled and fully unoccupied HOFA states, in agreement with the analysis in SN 9. Specifically, the quarter-filled corner states at $k_z = 0$ in Fig. 5(c) of the main text represent the particle-hole conjugates of the three-quarters-filled fragile-phase corner modes observable at $k_z = 0$ in the rod bands of the model of an $s-d$-hybridized, noncentrosymmetric, fragile topological Dirac semimetal introduced in this work (Fig. 3(h) of the main text and SN 9).

SUPPLEMENTARY REFERENCES

[1] Po, H. C., Watanabe, H. & Vishwanath, A. Fragile topology and wannier obstructions. *Phys. Rev. Lett.* **121**, 126402 (2018). URL https://link.aps.org/doi/10.1103/PhysRevLett.121.126402.

[2] Cano, J. *et al.* Topology of disconnected elementary band representations. *Phys. Rev. Lett.* **120**, 266401 (2018). URL https://link.aps.org/doi/10.1103/PhysRevLett.120.266401.

[3] Cano, J., Bradlyn, B., Elcoro, L., Wang, Z. & Bernevig, B. A. *In preparation.*

[4] Bouhon, A., Black-Schaffer, A. M. & Slager, R.-J. Wilson loop approach to fragile topology of split elementary band representations and topological crystalline insulators with time-reversal symmetry. *Phys. Rev. B* **100**, 195135 (2019). URL https://link.aps.org/doi/10.1103/PhysRevB.100.195135.

[5] Bradlyn, B., Wang, Z., Cano, J. & Bernevig, B. A. Disconnected elementary band representations, fragile topology, and wilson loops as topological indices: An example on the triangular lattice. *Phys. Rev. B* **99**, 045140 (2019). URL https://link.aps.org/doi/10.1103/PhysRevB.99.045140.

[6] Song, Z. *et al.* All magic angles in twisted bilayer graphene are topological. *Phys. Rev. Lett.* **123**, 036401 (2019). URL https://link.aps.org/doi/10.1103/PhysRevLett.123.036401.

[7] Zou, L., Po, H. C., Vishwanath, A. & Senthil, T. Band structure of twisted bilayer graphene: Emergent symmetries, commensurate approximants, and wannier obstructions. *Phys. Rev. B* **98**, 085435 (2018). URL https://link.aps.org/doi/10.1103/PhysRevB.98.085435.

[8] Po, H. C., Zou, L., Senthil, T. & Vishwanath, A. Faithful tight-binding models and fragile topology of magic-angle bilayer graphene. *Phys. Rev. B* **99**, 195455 (2019). URL https://link.aps.org/doi/10.1103/PhysRevB.99.195455.

[9] Liu, S., Vishwanath, A. & Khalaf, E. Shift insulators: Rotation-protected two-dimensional topological crystalline insulators. *Phys. Rev. X* **9**, 031003 (2019). URL https://link.aps.org/doi/10.1103/PhysRevX.9.031003.

[10] Ahn, J., Kim, D., Kim, Y. & Yang, B.-J. Band topology and linking structure of nodal line semimetals with Z_2 monopole charges. *Phys. Rev. Lett.* **121**, 106403 (2018). URL https://link.aps.org/doi/10.1103/PhysRevLett.121.106403.

[11] Wang, Z., Wieder, B. J., Li, J., Yan, B. & Bernevig, B. A. Higher-order topology, monopole nodal lines, and the origin of large fermi arcs in transition metal dichalcogenides $x\mathrm{te}_2$ ($x = \mathrm{Mo}, \mathrm{W}$). *Phys. Rev. Lett.* **123**, 186401 (2019). URL https://link.aps.org/doi/10.1103/PhysRevLett.123.186401.

[12] Else, D. V., Po, H. C. & Watanabe, H. Fragile topological phases in interacting systems. *Phys. Rev. B* **99**, 125122 (2019). URL https://link.aps.org/doi/10.1103/PhysRevB.99.125122.

[13] Ahn, J., Park, S. & Yang, B.-J. Failure of nielsen-ninomiya theorem and fragile topology in two-dimensional systems with space-time inversion symmetry: Application to twisted bilayer graphene at magic angle. *Phys. Rev. X* **9**, 021013 (2019). URL https://link.aps.org/doi/10.1103/PhysRevX.9.021013.

[14] Hwang, Y., Ahn, J. & Yang, B.-J. Fragile topology protected by inversion symmetry: Diagnosis, bulk-boundary correspondence, and wilson loop. *Phys. Rev. B* **100**, 205126 (2019). URL https://link.aps.org/doi/10.1103/PhysRevB.100.205126.

[15] Song, Z., Elcoro, L., Regnault, N. & Bernevig, B. A. Fragile Phases As Affine Monoids: Full Classification and Material Examples. *arXiv e-prints* arXiv:1905.03262 (2019). 1905.03262.

[16] Kooi, S. H., van Miert, G. & Ortix, C. Classification of crystalline insulators without symmetry indicators: Atomic and fragile topological phases in twofold rotation symmetric systems. *Phys. Rev. B* **100**, 115160 (2019). URL https://link.aps.org/doi/10.1103/PhysRevB.100.115160.

[17] Wieder, B. J. & Bernevig, B. A. The Axion Insulator as a Pump of Fragile Topology. *ArXiv e-prints* (2018). 1810.02373.

[18] Schindler, F., Tsirkin, S. S., Neupert, T., Bernevig, B. A. & Wieder, B. J. *In Preparation.*

[19] Bradley, C. J. & Cracknell, A. P. *The Mathematical Theory of Symmetry in Solids* (Clarendon Press Oxford, Oxford, United Kingdom, 1972).

[20] Litvin, D. B. *Magnetic Group Tables* (International Union of Crystallography, 2013).

[21] Su, W. P., Schrieffer, J. R. & Heeger, A. J. Solitons in polyacetylene. *Phys. Rev. Lett.* **42**, 1698–1701 (1979). URL https://link.aps.org/doi/10.1103/PhysRevLett.42.1698.

[22] Rice, M. J. & Mele, E. J. Elementary excitations of a linearly conjugated diatomic polymer. *Phys. Rev. Lett.* **49**, 1455–1459 (1982). URL https://link.aps.org/doi/10.1103/PhysRevLett.49.1455.

[23] Kopsky, V. & Litvin, D. *International Tables for Crystallography, Volume E: Subperiodic Groups.* International Tables for Crystallography (Springer Netherlands, 2002). URL https://books.google.com/books?id=if8nMGopkNgC.

[24] Bradlyn, B. *et al.* Topological quantum chemistry. *Nature* **547**, 298 EP – (2017). URL http://dx.doi.org/10.1038/nature23268. Article.

[25] Marzari, N., Mostofi, A. A., Yates, J. R., Souza, I. & Vanderbilt, D. Maximally localized wannier functions: Theory and applications. *Rev. Mod. Phys.* **84**, 1419–1475 (2012). URL https://link.aps.org/doi/10.1103/RevModPhys.84.1419.

[26] Benalcazar, W. A., Bernevig, B. A. & Hughes, T. L. Quantized electric multipole insulators. *Science* **357**, 61–66 (2017). URL http://science.sciencemag.org/content/357/6346/61.

[27] Wieder, B. J. *et al.* Wallpaper fermions and the nonsymmorphic dirac insulator. *Science* **361**, 246–251 (2018). URL http://science.sciencemag.org/content/361/6399/246.

[28] Benalcazar, W. A., Bernevig, B. A. & Hughes, T. L. Electric multipole moments, topological multipole moment pumping,

and chiral hinge states in crystalline insulators. *Phys. Rev. B* **96**, 245115 (2017). URL https://link.aps.org/doi/10.1103/PhysRevB.96.245115.

[29] Setyawan, W. & Curtarolo, S. High-throughput electronic band structure calculations: Challenges and tools. *Computational Materials Science* **49**, 299 – 312 (2010). URL http://www.sciencedirect.com/science/article/pii/S0927025610002697.

[30] Ilia, A. M. *et al. zkri*, vol. 221, chap. Bilbao Crystallographic Server: I. Databases and crystallographic computing programs, 15 (2006). URL https://www.degruyter.com/view/j/zkri.2006.221.issue-1/zkri.2006.221.1.15/zkri.2006.221.1.15.xml. 1.

[31] Aroyo, M. I., Kirov, A., Capillas, C., Perez-Mato, J. M. & Wondratschek, H. Bilbao Crystallographic Server. II. Representations of crystallographic point groups and space groups. *Acta Crystallographica Section A* **62**, 115–128 (2006). URL https://doi.org/10.1107/S0108767305040286.

[32] Yang, B.-J. & Nagaosa, N. Classification of stable three-dimensional dirac semimetals with nontrivial topology. *Nat. Comm.* **5**, 4898 (2014).

[33] Fang, C., Gilbert, M. J. & Bernevig, B. A. Bulk topological invariants in noninteracting point group symmetric insulators. *Phys. Rev. B* **86**, 115112 (2012). URL https://link.aps.org/doi/10.1103/PhysRevB.86.115112.

[34] Kargarian, M., Randeria, M. & Lu, Y.-M. Are the surface fermi arcs in dirac semimetals topologically protected? *Proceedings of the National Academy of Sciences* **113**, 8648–8652 (2016). URL http://www.pnas.org/content/113/31/8648.abstract.

[35] Kargarian, M., Lu, Y.-M. & Randeria, M. Deformation and stability of surface states in dirac semimetals. *Phys. Rev. B* **97**, 165129 (2018). URL https://link.aps.org/doi/10.1103/PhysRevB.97.165129.

[36] Le, C. *et al.* Dirac semimetal in -cui without surface fermi arcs. *Proceedings of the National Academy of Sciences* **115**, 8311–8315 (2018). URL http://www.pnas.org/content/115/33/8311.

[37] Wu, Y. *et al.* Fragility of fermi arcs in dirac semimetals. *Phys. Rev. B* **99**, 161113 (2019). URL https://link.aps.org/doi/10.1103/PhysRevB.99.161113.

[38] Fu, L. & Kane, C. L. Topological insulators with inversion symmetry. *Phys. Rev. B* **76**, 045302 (2007). URL https://link.aps.org/doi/10.1103/PhysRevB.76.045302.

[39] Song, Z., Fang, Z. & Fang, C. $(d-2)$-dimensional edge states of rotation symmetry protected topological states. *Phys. Rev. Lett.* **119**, 246402 (2017). URL https://link.aps.org/doi/10.1103/PhysRevLett.119.246402.

[40] Aroyo, M. I., Kirov, A., Capillas, C., Perez-Mato, J. M. & Wondratschek, H. Bilbao Crystallographic Server. II. Representations of crystallographic point groups and space groups. *Acta Crystallographica Section A* **62**, 115–128 (2006). URL https://doi.org/10.1107/S0108767305040286.

[41] Python tight binding open-source package. http://physics.rutgers.edu/pythtb/.

[42] Hsieh, T. H. *et al.* Topological crystalline insulators in the snte material class. *Nature Communications* **3**, 982 EP – (2012). URL http://dx.doi.org/10.1038/ncomms1969. Article.

[43] Liu, J., Qian, X. & Fu, L. Crystal field effect induced topological crystalline insulators in monolayer ivâeur"vi semiconductors. *Nano Letters* **15**, 2657–2661 (2015). URL https://doi.org/10.1021/acs.nanolett.5b00308.

[44] Teo, J. C. Y., Fu, L. & Kane, C. L. Surface states and topological invariants in three-dimensional topological insulators: Application to $\mathrm{bi}_{1-x}\mathrm{sb}_x$. *Phys. Rev. B* **78**, 045426 (2008). URL https://link.aps.org/doi/10.1103/PhysRevB.78.045426.

[45] Fang, C. Private communication.

[46] Schindler, F. *et al.* Higher-order topological insulators. *Science Advances* **4** (2018). URL http://advances.sciencemag.org/content/4/6/eaat0346.

[47] Langbehn, J., Peng, Y., Trifunovic, L., von Oppen, F. & Brouwer, P. W. Reflection-symmetric second-order topological insulators and superconductors. *Phys. Rev. Lett.* **119**, 246401 (2017). URL https://link.aps.org/doi/10.1103/PhysRevLett.119.246401.

[48] Fang, C. & Fu, L. Rotation Anomaly and Topological Crystalline Insulators. *ArXiv e-prints* (2017). 1709.01929.

[49] Khalaf, E., Po, H. C., Vishwanath, A. & Watanabe, H. Symmetry indicators and anomalous surface states of topological crystalline insulators. *Phys. Rev. X* **8**, 031070 (2018). URL https://link.aps.org/doi/10.1103/PhysRevX.8.031070.

[50] Schindler, F. *et al.* Higher-order topology in bismuth. *Nature Physics* **14**, 918–924 (2018). URL https://doi.org/10.1038/s41567-018-0224-7.

[51] Castro Neto, A. H., Guinea, F., Peres, N. M. R., Novoselov, K. S. & Geim, A. K. The electronic properties of graphene. *Rev. Mod. Phys.* **81**, 109–162 (2009). URL https://link.aps.org/doi/10.1103/RevModPhys.81.109.

[52] Fujita, M., Wakabayashi, K., Nakada, K. & Kusakabe, K. Peculiar localized state at zigzag graphite edge. *Journal of the Physical Society of Japan* **65**, 1920–1923 (1996). URL http://dx.doi.org/10.1143/JPSJ.65.1920. http://dx.doi.org/10.1143/JPSJ.65.1920.

[53] Nakada, K., Fujita, M., Dresselhaus, G. & Dresselhaus, M. S. Edge state in graphene ribbons: Nanometer size effect and edge shape dependence. *Phys. Rev. B* **54**, 17954–17961 (1996). URL http://link.aps.org/doi/10.1103/PhysRevB.54.17954.

[54] Ruffieux, P. *et al.* On-surface synthesis of graphene nanoribbons with zigzag edge topology. *Nature* **531**, 489 EP – (2016). URL http://dx.doi.org/10.1038/nature17151.

[55] Zhang, F. Spontaneous chiral symmetry breaking in bilayer graphene. *Synthetic Metals* **210**, 9 – 18 (2015). URL http://www.sciencedirect.com/science/article/pii/S0379677915300369. Reviews of Current Advances in Graphene Science and Technology.

[56] Wieder, B. J. & Kane, C. L. Spin-orbit semimetals in the layer groups. *Phys. Rev. B* **94**, 155108 (2016). URL http://link.aps.org/doi/10.1103/PhysRevB.94.155108.

[57] Elcoro, L. *et al.* Double crystallographic groups and their representations on the Bilbao Crystallographic Server. *Journal of Applied Crystallography* **50**, 1457–1477 (2017). URL https://doi.org/10.1107/S1600576717011712.

[58] Vergniory, M. G. *et al.* Graph theory data for topological quantum chemistry. *Phys. Rev. E* **96**, 023310 (2017). URL https://link.aps.org/doi/10.1103/PhysRevE.96.023310.

[59] Cano, J. *et al.* Building blocks of topological quantum chemistry: Elementary band representations. *Phys. Rev. B* **97**, 035139 (2018). URL https://link.aps.org/doi/10.1103/PhysRevB.97.035139.

[60] Song, Z., Zhang, T., Fang, Z. & Fang, C. Quantitative mappings between symmetry and topology in solids. *Nature Communications* **9**, 3530 (2018). URL https://doi.org/10.1038/s41467-018-06010-w.

[61] Kruthoff, J., de Boer, J., van Wezel, J., Kane, C. L. & Slager, R.-J. Topological classification of crystalline insulators through band structure combinatorics. *Phys. Rev. X* **7**, 041069 (2017). URL https://link.aps.org/doi/10.1103/PhysRevX.7.041069.

[62] Po, H. C., Vishwanath, A. & Watanabe, H. Symmetry-based indicators of band topology in the 230 space groups. *Nature Communications* **8**, 50 (2017). URL https://doi.org/10.1038/s41467-017-00133-2.

[63] Ivantchev, S. *et al.* *SUPERGROUPS* – a computer program for the determination of the supergroups of the space groups. *Journal of Applied Crystallography* **35**, 511–512 (2002). URL https://doi.org/10.1107/S002188980200732X.

[64] Young, S. M. & Wieder, B. J. Filling-enforced magnetic dirac semimetals in two dimensions. *Phys. Rev. Lett.* **118**, 186401 (2017). URL https://link.aps.org/doi/10.1103/PhysRevLett.118.186401.

[65] Liu, F. & Wakabayashi, K. Novel topological phase with a zero berry curvature. *Phys. Rev. Lett.* **118**, 076803 (2017). URL https://link.aps.org/doi/10.1103/PhysRevLett.118.076803.

[66] Franca, S., van den Brink, J. & Fulga, I. C. An anomalous higher-order topological insulator. *Phys. Rev. B* **98**, 201114 (2018). URL https://link.aps.org/doi/10.1103/PhysRevB.98.201114.

[67] Kunst, F. K., van Miert, G. & Bergholtz, E. J. Lattice models with exactly solvable topological hinge and corner states. *Phys. Rev. B* **97**, 241405 (2018). URL https://link.aps.org/doi/10.1103/PhysRevB.97.241405.

[68] Noh, J. *et al.* Topological protection of photonic mid-gap defect modes. *Nature Photonics* **12**, 408–415 (2018). URL https://doi.org/10.1038/s41566-018-0179-3.

[69] Benalcazar, W. A., Li, T. & Hughes, T. L. Quantization of fractional corner charge in C_n-symmetric higher-order topological crystalline insulators. *Phys. Rev. B* **99**, 245151 (2019). URL https://link.aps.org/doi/10.1103/PhysRevB.99.245151.

[70] Schindler, F. *et al.* Fractional corner charges in spin-orbit coupled crystals. *Phys. Rev. Research* **1**, 033074 (2019). URL https://link.aps.org/doi/10.1103/PhysRevResearch.1.033074.

[71] Ezawa, M. Higher-order topological insulators and semimetals on the breathing kagome and pyrochlore lattices. *Phys. Rev. Lett.* **120**, 026801 (2018). URL https://link.aps.org/doi/10.1103/PhysRevLett.120.026801.

[72] Benalcazar, W. A., Teo, J. C. Y. & Hughes, T. L. Classification of two-dimensional topological crystalline superconductors and majorana bound states at disclinations. *Phys. Rev. B* **89**, 224503 (2014). URL https://link.aps.org/doi/10.1103/PhysRevB.89.224503.

[73] Park, M. J., Kim, Y., Cho, G. Y. & Lee, S. Higher-order topological insulator in twisted bilayer graphene. *Phys. Rev. Lett.* **123**, 216803 (2019). URL https://link.aps.org/doi/10.1103/PhysRevLett.123.216803.

[74] Thouless, D. J. Wannier functions for magnetic sub-bands. *Journal of Physics C: Solid State Physics* **17**, L325 (1984). URL http://stacks.iop.org/0022-3719/17/i=12/a=003.

[75] Soluyanov, A. A. & Vanderbilt, D. Smooth gauge for topological insulators. *Phys. Rev. B* **85**, 115415 (2012). URL https://link.aps.org/doi/10.1103/PhysRevB.85.115415.

[76] Zak, J. Band representations and symmetry types of bands in solids. *Phys. Rev. B* **23**, 2824–2835 (1981). URL https://link.aps.org/doi/10.1103/PhysRevB.23.2824.

[77] Zak, J. Band representations of space groups. *Phys. Rev. B* **26**, 3010–3023 (1982). URL https://link.aps.org/doi/10.1103/PhysRevB.26.3010.

[78] Huang, S.-J., Song, H., Huang, Y.-P. & Hermele, M. Building crystalline topological phases from lower-dimensional states. *Phys. Rev. B* **96**, 205106 (2017). URL https://link.aps.org/doi/10.1103/PhysRevB.96.205106.

[79] Thouless, D. J., Kohmoto, M., Nightingale, M. P. & den Nijs, M. Quantized hall conductance in a two-dimensional periodic potential. *Phys. Rev. Lett.* **49**, 405–408 (1982). URL https://link.aps.org/doi/10.1103/PhysRevLett.49.405.

[80] Bernevig, B. A., Hughes, T. L. & Zhang, S.-C. Quantum spin hall effect and topological phase transition in hgte quantum wells. *Science* **314**, 1757–1761 (2006). URL http://science.sciencemag.org/content/314/5806/1757.

[81] Kane, C. L. & Mele, E. J. Quantum spin hall effect in graphene. *Phys. Rev. Lett.* **95**, 226801 (2005). URL https://link.aps.org/doi/10.1103/PhysRevLett.95.226801.

[82] Qi, X.-L., Hughes, T. L. & Zhang, S.-C. Topological field theory of time-reversal invariant insulators. *Phys. Rev. B* **78**, 195424 (2008). URL https://link.aps.org/doi/10.1103/PhysRevB.78.195424.

[83] Yu, R., Qi, X. L., Bernevig, A., Fang, Z. & Dai, X. Equivalent expression of $\mathbb{Z}_2$ topological invariant for band insulators using the non-abelian berry connection. *Phys. Rev. B* **84**, 075119 (2011). URL https://link.aps.org/doi/10.1103/PhysRevB.84.075119.

[84] Soluyanov, A. A. & Vanderbilt, D. Wannier representation of $\mathbb{Z}_2$ topological insulators. *Phys. Rev. B* **83**, 035108 (2011). URL https://link.aps.org/doi/10.1103/PhysRevB.83.035108.

[85] Zak, J. Berry's phase for energy bands in solids. *Phys. Rev. Lett.* **62**, 2747–2750 (1989). URL https://link.aps.org/doi/10.1103/PhysRevLett.62.2747.

[86] King-Smith, R. D. & Vanderbilt, D. Theory of polarization of crystalline solids. *Phys. Rev. B* **47**, 1651–1654 (1993). URL https://link.aps.org/doi/10.1103/PhysRevB.47.1651.

[87] Alexandradinata, A., Dai, X. & Bernevig, B. A. Wilson-loop characterization of inversion-symmetric topological insulators. *Physical Review B* **89**, 155114 (2014).

[88] Alexandradinata, A., Fang, C., Gilbert, M. J. & Bernevig, B. A. Spin-orbit-free topological insulators without time-reversal

symmetry. *Phys. Rev. Lett.* **113**, 116403 (2014). URL http://link.aps.org/doi/10.1103/PhysRevLett.113.116403.
[89] Alexandradinata, A., Wang, Z. & Bernevig, B. A. Topological insulators from group cohomology. *Physical Review X* **6**, 021008 (2016).
[90] Wang, Z., Alexandradinata, A., Cava, R. J. & Bernevig, B. A. Hourglass fermions. *Nature* **532**, 189–194 (2016).
[91] Altmann, S. & Herzig, P. *Point-Group Theory Tables* (University of Vienna, 2011), 2 edn. URL http://phaidra.univie.ac.at/o:104731.
[92] Bradlyn, B. *et al.* Beyond dirac and weyl fermions: Unconventional quasiparticles in conventional crystals. *Science* **353** (2016). URL http://science.sciencemag.org/content/353/6299/aaf5037.
[93] Chang, G. *et al.* Unconventional chiral fermions and large topological fermi arcs in rhsi. *Phys. Rev. Lett.* **119**, 206401 (2017). URL https://link.aps.org/doi/10.1103/PhysRevLett.119.206401.
[94] Tang, P., Zhou, Q. & Zhang, S.-C. Multiple types of topological fermions in transition metal silicides. *Phys. Rev. Lett.* **119**, 206402 (2017). URL https://link.aps.org/doi/10.1103/PhysRevLett.119.206402.
[95] Chang, G. *et al.* Topological quantum properties of chiral crystals. *Nature materials* 1 (2018).
[96] Bacry, H., Michel, L. & Zak, J. Symmetry and analyticity of energy bands in solids. *Phys. Rev. Lett.* **61**, 1005–1008 (1988). URL https://link.aps.org/doi/10.1103/PhysRevLett.61.1005.
[97] Fu, L. & Kane, C. L. Time reversal polarization and a Z_2 adiabatic spin pump. *Phys. Rev. B* **74**, 195312 (2006). URL https://link.aps.org/doi/10.1103/PhysRevB.74.195312.
[98] Alexandradinata, A. & Bernevig, B. A. Berry-phase description of topological crystalline insulators. *Phys. Rev. B* **93**, 205104 (2016). URL http://link.aps.org/doi/10.1103/PhysRevB.93.205104.
[99] Jackson, J. *Classical electrodynamics* (Wiley, 1975). URL https://books.google.be/books?id=_7rvAAAAMAAJ.
[100] Watanabe, H., Po, H. C., Vishwanath, A. & Zaletel, M. Filling constraints for spin-orbit coupled insulators in symmorphic and nonsymmorphic crystals. *Proceedings of the National Academy of Sciences* **112**, 14551–14556 (2015). URL http://www.pnas.org/content/112/47/14551.abstract.
[101] Hughes, T. L., Prodan, E. & Bernevig, B. A. Inversion-symmetric topological insulators. *Phys. Rev. B* **83**, 245132 (2011). URL https://link.aps.org/doi/10.1103/PhysRevB.83.245132.
[102] Liu, Y. *et al.* Topological corner modes in a brick lattice with nonsymmorphic symmetry. *arXiv e-prints* arXiv:1812.11846 (2018). 1812.11846.
[103] Bednik, G. Surface states in dirac semimetals and topological crystalline insulators. *Phys. Rev. B* **98**, 045140 (2018). URL https://link.aps.org/doi/10.1103/PhysRevB.98.045140.
[104] Liu, Z. K. *et al.* Discovery of a three-dimensional topological dirac semimetal, na3bi. *Science* **343**, 864–867 (2014). URL http://science.sciencemag.org/content/343/6173/864.
[105] Chiu, C.-K. & Schnyder, A. P. Classification of crystalline topological semimetals with an application to na 3 bi. *Journal of Physics: Conference Series* **603**, 012002 (2015). URL http://stacks.iop.org/1742-6596/603/i=1/a=012002.
[106] Wang, Z. *et al.* Dirac semimetal and topological phase transitions in A_3bi (a = Na, k, rb). *Phys. Rev. B* **85**, 195320 (2012). URL https://link.aps.org/doi/10.1103/PhysRevB.85.195320.
[107] Vogel, R. & Schuster, H.-U. Neue elektrovalente ternre verbindungen des kaliums mit magnesium und elementen der 5. hauptgruppe / new ternary compounds of potassium with magnesium and elements of the 5. main group **34** (1979).
[108] Le, C. *et al.* Three-dimensional topological critical dirac semimetal in aMgBi (a = k, rb, cs). *Phys. Rev. B* **96**, 115121 (2017). URL https://link.aps.org/doi/10.1103/PhysRevB.96.115121.
[109] Zhang, X., Sun, S. & Lei, H. Narrow-gap semiconducting properties of kmgbi with multiband feature. *Phys. Rev. B* **95**, 035209 (2017). URL https://link.aps.org/doi/10.1103/PhysRevB.95.035209.
[110] Wang, Z., Weng, H., Wu, Q., Dai, X. & Fang, Z. Three-dimensional dirac semimetal and quantum transport in cd_3as_2. *Phys. Rev. B* **88**, 125427 (2013). URL http://link.aps.org/doi/10.1103/PhysRevB.88.125427.
[111] Yi, H. *et al.* Evidence of topological surface state in three-dimensional dirac semimetal cd3as2. *Scientific Reports* **4**, 6106 EP – (2014). URL http://dx.doi.org/10.1038/srep06106. Article.
[112] Fukui, T. Dirac fermion model associated with a second-order topological insulator. *Phys. Rev. B* **99**, 165129 (2019). URL https://link.aps.org/doi/10.1103/PhysRevB.99.165129.
[113] Ali, M. N. *et al.* The crystal and electronic structures of cd3as2, the three-dimensional electronic analogue of graphene. *Inorganic Chemistry* **53**, 4062–4067 (2014). URL https://doi.org/10.1021/ic403163d.
[114] Arushanov, E. Crystal growth and characterization of ii3v2 compounds. *Progress in Crystal Growth and Characterization* **3**, 211 – 255 (1980). URL http://www.sciencedirect.com/science/article/pii/0146353580900209.
[115] Peng, Y., Bao, Y. & von Oppen, F. Boundary green functions of topological insulators and superconductors. *Phys. Rev. B* **95**, 235143 (2017). URL https://link.aps.org/doi/10.1103/PhysRevB.95.235143.
[116] Jackiw, R. & Rebbi, C. Solitons with fermion number 1/2. *Phys. Rev. D* **13**, 3398–3409 (1976). URL https://link.aps.org/doi/10.1103/PhysRevD.13.3398.
[117] Bernevig, B. A. & Hughes, T. L. *Topological Insulators and Topological Superconductors* (Princeton University Press, Princeton, NJ, 2013).
[118] Taherinejad, M., Garrity, K. F. & Vanderbilt, D. Wannier center sheets in topological insulators. *Phys. Rev. B* **89**, 115102 (2014). URL https://link.aps.org/doi/10.1103/PhysRevB.89.115102.
[119] Ostrovsky, P. M., Gornyi, I. V. & Mirlin, A. D. Interaction-induced criticality in F_2 topological insulators. *Phys. Rev. Lett.* **105**, 036803 (2010). URL https://link.aps.org/doi/10.1103/PhysRevLett.105.036803.
[120] Rosenberg, G., Guo, H.-M. & Franz, M. Wormhole effect in a strong topological insulator. *Phys. Rev. B* **82**, 041104 (2010). URL https://link.aps.org/doi/10.1103/PhysRevB.82.041104.
[121] Kane, C. Chapter 1 - topological band theory and the 2 invariant. In Franz, M. & Molenkamp, L. (eds.) *Topological*

Insulators, vol. 6 of *Contemporary Concepts of Condensed Matter Science*, 3 – 34 (Elsevier, 2013). URL http://www.sciencedirect.com/science/article/pii/B9780444633149000019.

122 A, M. & D, S. *Physical Chemistry: a Molecular Approach* (Viva Books, 1988). URL https://books.google.fi/books?id=NPqaYgEACAAJ.

123 Blanco, M. A., Flrez, M. & Bermejo, M. Evaluation of the rotation matrices in the basis of real spherical harmonics. *Journal of Molecular Structure: THEOCHEM* **419**, 19 – 27 (1997). URL http://www.sciencedirect.com/science/article/pii/S0166128097001851.

124 Muggli, J. Cubic harmonics as linear combinations of spherical harmonics. *Zeitschrift für angewandte Mathematik und Physik ZAMP* **23**, 311–317 (1972). URL https://doi.org/10.1007/BF01593094.

125 Griffiths, D. *Introduction to Quantum Mechanics* (Cambridge University Press, 2016). URL https://books.google.com/books?id=0h-nDAAAQBAJ.

126 Shockley, W. On the surface states associated with a periodic potential. *Phys. Rev.* **56**, 317–323 (1939). URL https://link.aps.org/doi/10.1103/PhysRev.56.317.

127 Niemi, A. & Semenoff, G. Fermion number fractionization in quantum field theory. *Physics Reports* **135**, 99 – 193 (1986). URL http://www.sciencedirect.com/science/article/pii/0370157386901675.

128 Wilczek, F. Two applications of axion electrodynamics. *Phys. Rev. Lett.* **58**, 1799–1802 (1987). URL https://link.aps.org/doi/10.1103/PhysRevLett.58.1799.

129 Goldstone, J. & Wilczek, F. Fractional quantum numbers on solitons. *Phys. Rev. Lett.* **47**, 986–989 (1981). URL https://link.aps.org/doi/10.1103/PhysRevLett.47.986.

130 Young, S. M. & Kane, C. L. Dirac semimetals in two dimensions. *Phys. Rev. Lett.* **115**, 126803 (2015). URL http://link.aps.org/doi/10.1103/PhysRevLett.115.126803.

131 Wieder, B. J., Kim, Y., Rappe, A. M. & Kane, C. L. Double dirac semimetals in three dimensions. *Phys. Rev. Lett.* **116**, 186402 (2016). URL http://link.aps.org/doi/10.1103/PhysRevLett.116.186402.

132 Cracknell, A. P. The application of landau's theory of continuous phase transitions to magnetic phase transitions. *Journal of Physics C: Solid State Physics* **4**, 2488 (1971). URL http://stacks.iop.org/0022-3719/4/i=16/a=014.

133 Cracknell, A. P. Group theory and magnetic phenomena in solids. *Reports on Progress in Physics* **32**, 633 (1969). URL http://stacks.iop.org/0034-4885/32/i=2/a=305.

134 Lin, M. & Hughes, T. L. Topological quadrupolar semimetals. *Phys. Rev. B* **98**, 241103 (2018). URL https://link.aps.org/doi/10.1103/PhysRevB.98.241103.

135 Inorganic Crystal Structure Database (ICSD) (Fachinformationszentrum Karlsruhe, Karlsruhe, Germany, 2015).

136 Vergniory, M. G. *et al.* A complete catalogue of high-quality topological materials. *Nature* **566**, 480–485 (2019). URL https://doi.org/10.1038/s41586-019-0954-4.

137 Aroyo, M. I. (ed.) *International Tables for Crystallography, Volume A: Space-Group Symmetry*, vol. A (International Union of Crystallography, 2016). URL http://it.iucr.org/A/.

138 Young, S. M. *et al.* Dirac semimetal in three dimensions. *Phys. Rev. Lett.* **108**, 140405 (2012). URL https://link.aps.org/doi/10.1103/PhysRevLett.108.140405.

139 Steinberg, J. A. *et al.* Bulk dirac points in distorted spinels. *Phys. Rev. Lett.* **112**, 036403 (2014). URL https://link.aps.org/doi/10.1103/PhysRevLett.112.036403.

140 Gallego, S. V. *et al. MAGNDATA*: towards a database of magnetic structures. I.The commensurate case. *Journal of Applied Crystallography* **49**, 1750–1776 (2016).

141 Schoop, L. M. *et al.* Tunable weyl and dirac states in the nonsymmorphic compound cesbte. *Science Advances* **4** (2018). URL http://advances.sciencemag.org/content/4/2/eaar2317.

142 Tsirkin, S. S., Souza, I. & Vanderbilt, D. Composite weyl nodes stabilized by screw symmetry with and without time-reversal invariance. *Phys. Rev. B* **96**, 045102 (2017). URL https://link.aps.org/doi/10.1103/PhysRevB.96.045102.

143 Kresse, G. & Joubert, D. From ultrasoft pseudopotentials to the projector augmented-wave method. *Phys. Rev. B* **59**, 1758–1775 (1999). URL https://link.aps.org/doi/10.1103/PhysRevB.59.1758.

144 Kresse, G. & Furthmller, J. Efficiency of ab-initio total energy calculations for metals and semiconductors using a plane-wave basis set. *Computational Materials Science* **6**, 15 – 50 (1996). URL http://www.sciencedirect.com/science/article/pii/0927025696000080.

145 Kresse, G. & Furthmüller, J. Efficient iterative schemes for ab initio total-energy calculations using a plane-wave basis set. *Phys. Rev. B* **54**, 11169–11186 (1996). URL https://link.aps.org/doi/10.1103/PhysRevB.54.11169.

146 Xu, S.-Y. *et al.* Observation of fermi arc surface states in a topological metal. *Science* **347**, 294–298 (2015). URL https://science.sciencemag.org/content/347/6219/294.

147 Armitage, N. P., Mele, E. J. & Vishwanath, A. Weyl and dirac semimetals in three-dimensional solids. *Rev. Mod. Phys.* **90**, 015001 (2018). URL https://link.aps.org/doi/10.1103/RevModPhys.90.015001.

148 Potter, A. C., Kimchi, I. & Vishwanath, A. Quantum oscillations from surface fermi arcs in weyl and dirac semimetals. *Nature Communications* **5**, 5161 EP – (2014). URL http://dx.doi.org/10.1038/ncomms6161. Article.

149 Ma, J.-Z. *et al.* Three-component fermions with surface fermi arcs in tungsten carbide. *Nature Physics* **14**, 349–354 (2018). URL https://doi.org/10.1038/s41567-017-0021-8.

150 Borisenko, S. *et al.* Experimental realization of a three-dimensional dirac semimetal. *Phys. Rev. Lett.* **113**, 027603 (2014). URL https://link.aps.org/doi/10.1103/PhysRevLett.113.027603.

151 Schnherr, P. & Hesjedal, T. Structural properties and growth mechanism of cd3as2 nanowires. *Applied Physics Letters* **106**, 013115 (2015). URL https://doi.org/10.1063/1.4905564. https://doi.org/10.1063/1.4905564.

152 Kealhofer, D. A. *et al.* Basal-plane growth of cadmium arsenide by molecular beam epitaxy. *Phys. Rev. Materials* **3**, 031201

(2019). URL https://link.aps.org/doi/10.1103/PhysRevMaterials.3.031201.

153 Schwartz, K., Gillson, J. & Shannon, R. Crystal growth of cdpt3o6, mnpt3o6, copt3o6 and -pto2. *Journal of Crystal Growth* **60**, 251 – 254 (1982). URL http://www.sciencedirect.com/science/article/pii/0022024882900963.

154 Kim, R., Yang, B.-J. & Kim, C. H. Crystalline topological dirac semimetal phase in rutile structure $\beta^{'}-\mathrm{pto}_2$. *Phys. Rev. B* **99**, 045130 (2019). URL https://link.aps.org/doi/10.1103/PhysRevB.99.045130.

155 Shannon, R. Synthesis and properties of two new members of the rutile family rho2 and pto2. *Solid State Communications* **6**, 139 – 143 (1968). URL http://www.sciencedirect.com/science/article/pii/0038109868900197.

156 Herrero Fernndez, P. & Chamberland, B. A new high pressure form of pto2. *Journal of The Less Common Metals* **99** (1984).
