# Supplementary material for: Strong and fragile topological Dirac semimetals with higher-order Fermi arcs
Source: Nat Commun. 2020 Jan 31;11:627. doi: 10.1038/s41467-020-14443-5 (PMC6994491; doi:10.1038/s41467-020-14443-5)
Supplement: Supplementary file 2 — Peer Review File [file 41467_2020_14443_MOESM2_ESM.pdf]

Editorial Note: This manuscript has been previously reviewed at another journal that is not operating a transparent peer review scheme. The manuscript was considered suitable for publication without further review at Nature Communications .
